# Perturbation of endoplasmic reticulum proteostasis triggers tissue injury in the thyroid gland

**DOI:** 10.1172/jci.insight.169937

**Published:** 2023-06-22

**Authors:** Xiaohan Zhang, Crystal Young, Xiao-Hui Liao, Samuel Refetoff, Mauricio Torres, Yaron Tomer, Mihaela Stefan-Lifshitz, Hao Zhang, Dennis Larkin, Deyu Fang, Ling Qi, Peter Arvan

**Affiliations:** 1 Division of Metabolism, Endocrinology & Diabetes and; 2 Department of Molecular & Integrative Physiology, University of Michigan, Ann Arbor, Michigan, USA.; 3 Department of Medicine,; 4 Department of Pediatrics, and Committee on Genetics, Genomics, and Systems Biology, The University of Chicago, Chicago, Illinois, USA.; 5 Department of Medicine, Fleischer Institute for Diabetes and Metabolism, Albert Einstein College of Medicine, New York, New York, USA.; 6 Department of Pathology, Feinberg School of Medicine, Northwestern Medicine, Chicago, Illinois, USA.

**Keywords:** Cell Biology, Protein misfolding

## Abstract

Defects in endoplasmic reticulum (ER) proteostasis have been linked to diseases in multiple organ systems. Here we examined the impact of perturbation of ER proteostasis in mice bearing thyrocyte-specific knockout of either *HRD1* (to disable ER-associated protein degradation [ERAD]) or *ATG7* (to disable autophagy) in the absence or presence of heterozygous expression of misfolded mutant thyroglobulin (the most highly expressed thyroid gene product, synthesized in the ER). Misfolding-inducing thyroglobulin mutations are common in humans but are said to yield only autosomal-recessive disease — perhaps because misfolded thyroglobulin protein might undergo disposal by ERAD or ER macroautophagy. We find that as single defects, neither ERAD, nor autophagy, nor heterozygous thyroglobulin misfolding altered circulating thyroxine levels, and neither defective ERAD nor defective autophagy caused any gross morphological change in an otherwise WT thyroid gland. However, heterozygous expression of misfolded thyroglobulin itself triggered significant ER stress and individual thyrocyte death while maintaining integrity of the surrounding thyroid epithelium. In this context, deficiency of ERAD (but not autophagy) resulted in patchy whole-follicle death with follicular collapse and degeneration, accompanied by infiltration of bone marrow–derived macrophages. Perturbation of thyrocyte ER proteostasis is thus a risk factor for both cell death and follicular demise.

## Graphical abstract

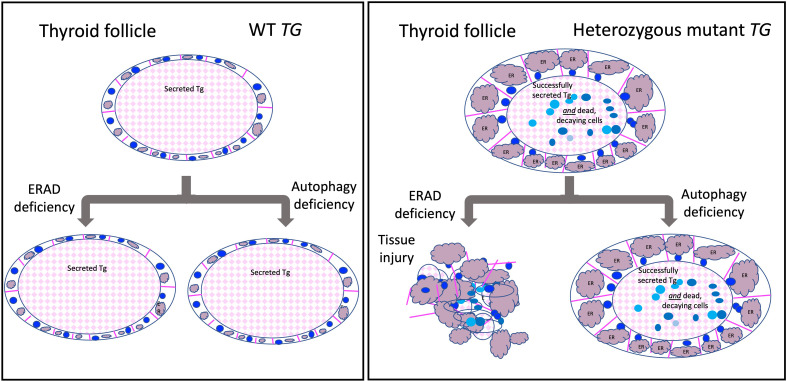

## Introduction

When the requirement for folding of newly synthesized secretory proteins (molecules per second entering the endoplasmic reticulum [ER]) exceeds the protein-folding capacity of the ER organelle, protein misfolding increases, triggering ER stress (1). ER stress itself is potentially proteotoxic (i.e., lethal to cells; ref. 2) and may activate ER stress response signaling (3). Events downstream of such signaling are broad and branching and include critical adaptive responses such as increased ER molecular chaperone expression as well as upregulation of machinery for ER-based protein disposal, e.g., classical Sel1L/Hrd1-mediated ER-associated degradation, or ERAD (4–6), as well as ER autophagy (7, 8). In addition to potential cellular lethality caused directly by accumulation of misfolded ER protein complexes, persistent exuberant ER stress signaling can also trigger cell death (9–11). Nowhere in the body is this seen more clearly than in the thyroid gland (12–14).

The vertebrate thyroid gland uses a mechanism for the biosynthesis of thyroxine (T_4_) that has been conserved in evolution for the past 500 million years. Specifically, thyroid epithelial cells (thyrocytes) form follicles that enclose a central cavity, and each thyrocyte synthesizes and apically secretes a vast quantity of thyroglobulin (Tg, encoded by the *TG* gene) (15, 16). Secreted Tg is iteratively iodinated by machinery localized at the apical plasma membrane, and the iodination of protein within the follicle lumen (17) triggers the formation of T_4_ within Tg itself (18–22) (i.e., a thyroid-specific posttranslational modification; refs. 23, 24). When the thyroid gland is stimulated to release thyroid hormone, internalization of Tg protein via apical endocytosis from the follicle lumen into the endolysosomal system of the surrounding thyrocytes (25) leads to lysosomal digestion of iodinated Tg protein, liberating T_4_ (and some tri-iodothyronine [T_3_]) that can exit lysosomes and cross the basolateral plasma membrane into the systemic circulation (26).

Tg is one of the largest proteins in the vertebrate secretome. Large secretory proteins pose unique demands because of the high energy requirements for biosynthesis, increased opportunities for misfolding, increased difficulty in protein packaging and trafficking, and increased complexity of posttranslational modifications (27). Adding to these issues is the very large number of pathogenic mutations in the *TG* gene that are linked to congenital hypothyroidism (28), including point mutations in the ChEL domain that cause global Tg folding defects (14, 16, 29).

All of the above features represent a major challenge to thyrocyte proteostasis. As best we know, all structurally defective Tg mutants in both humans and animal models are subject to ER quality control and thus are blocked in anterograde export from the ER (29). In the rare homozygous condition, this causes thyroidal ER stress with massive thyrocyte swelling (attributable entirely to expanded ER volume) accompanied by thyroid epithelial cell death (12–14). Interestingly, the prevalence of heterozygously expressed pathogenic variants of Tg is quite common in the human population (30, 31). Nevertheless, because congenital hypothyroidism caused by defective Tg is inherited as an autosomal-recessive disease (32), it has been assumed that thyrocytes can “handle” the gene product derived from a single mutant allele.

In this report we have examined mice bearing 1 of 3 defects: thyrocyte-specific *HRD1^TPO^* mice defective for ERAD, thyrocyte-specific *ATG7^TPO^* mice defective for autophagy, and *TG^+/cog^* heterozygous mice, with 1 allele encoding misfolded mutant Tg (Tg-L2263P, cog Tg) — as well as double mutants of *TG^+/cog^* in a background of *HRD1^TPO^* or *ATG7^TPO^*. Our results shed light on the extent to which each of these components links proteostasis to cell and tissue survival.

## Results

### Tissue-specific deficiency of ERAD, or autophagy, is well tolerated in the thyroid gland.

**Figure 1:**
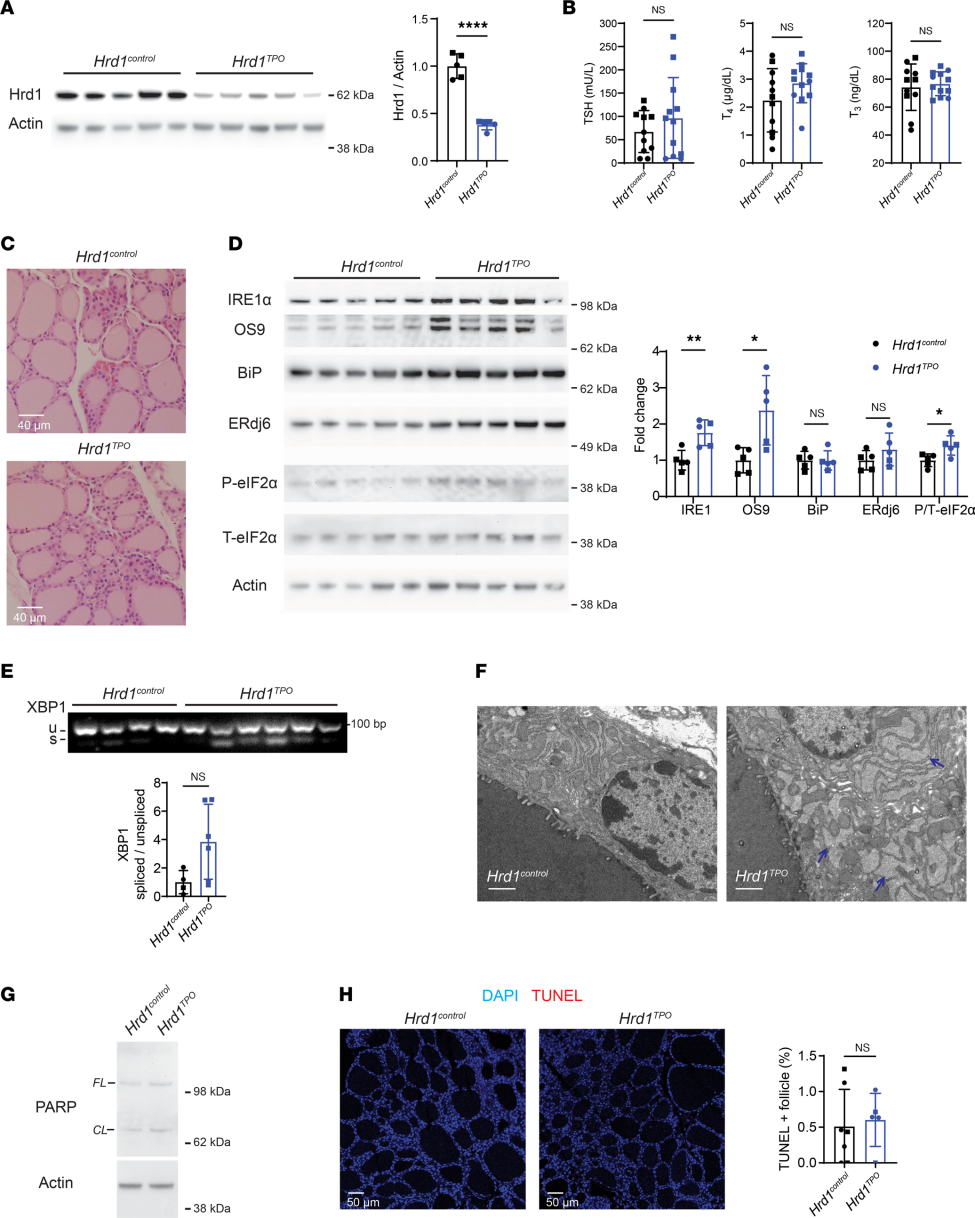
Tissue-specific ERAD deficiency in the thyroid gland. (**A**) Western blotting of Hrd1 and actin (*gels at left*) and quantitation (*right*) in the thyroid glands of *Hrd1^control^* and *Hrd1^TPO^* mice (*n* = 5 mice per group). (**B**) Serum thyroid-stimulating hormone (TSH) and total T_4_ and T_3_ levels of *Hrd1^control^* and *Hrd1^TPO^* mice (*n* = 11–12 mice per group). (**C**) Representative H&E images of thyroid glands from *Hrd1^control^* and *Hrd1^TPO^* mice (*n* = 5 mice per group). (**D**) Western blotting analysis of IRE1α, OS9, BiP, ERdj6, phosphorylated eIF2α (P-eIF2α), and total eIF2α (T-eIF2α) (*gels at left*) and quantification (normalized to actin; except phosphorylated eIF2α normalized to total eIF2α; bar graph *at right*) in the thyroid glands of *Hrd1^control^* and *Hrd1^TPO^* mice (*n* = 5 mice per group). (**E**) PCR revealing spliced and unspliced *XBP1* mRNA as a readout of Ire1 activity (*upper*) and quantitation (*bar graph below*) in the thyroids of *Hrd1^control^* and *Hrd1^TPO^* mice (*n* = 4–6 mice group). (**F**) Transmission electron microscopy of *Hrd1^control^* and *Hrd1^TPO^* thyrocytes (white scale bars = 1 μm; *n* = 1–2 mice per group); the thyroid follicle lumen is seen at the lower left of each image; blue arrows point to distended ER. (**G**) Representative Western blotting analysis of full-length (*FL*) and cleaved (*CL*) PARP in the thyroid glands of *Hrd1^control^* and *Hrd1^TPO^* mice (*n* = 6 mice per group). (**H**) Representative TUNEL labeling (red, with DAPI counterstain in blue, *images at left*) and quantitation of the fraction of TUNEL-positive follicles (as a fraction of total follicles, *right*) shows negligible thyroid cell death in either genotype. Graphs in panels **A**, **B**, and **D**–**F** show mean ± SD; *P < 0.05, **P < 0.01, ****P < 0.0001 (unpaired 2-tailed Student’s *t* test); each dot represents an individual animal (squares = males; circles = females).

**Figure 2:**
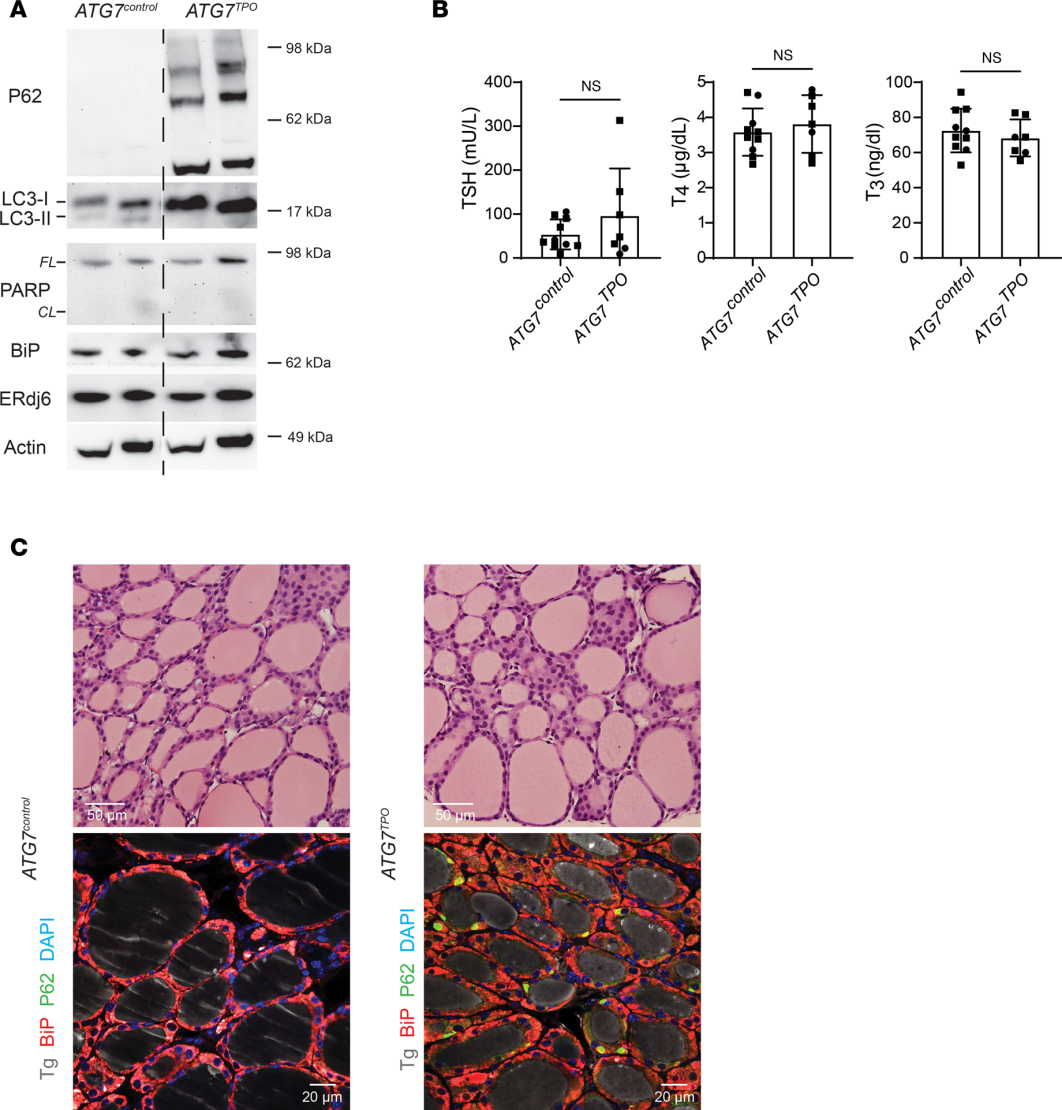
Tissue-specific autophagy deficiency in thyroid glands of mice expressing WT Tg. (**A**) Western blotting analysis of p62, LC3, full-length (FL) and cleaved (*CL*) PARP, BiP, and ERdj6 (*n* = 3 mice per group, 7.2 ± 1.3 mo; 2 animals shown) in *ATG7^control^* and *ATG7^TPO^* mouse thyroid glands. (**B**) Serum TSH and total T_4_ and T_3_ levels of *ATG7^control^* and *ATG7^TPO^* mice (*n* = 7–10 mice per group, 7.4 ± 1.2 mo). The graph shows mean ± SD; unpaired 2-tailed Student’s *t* test; each dot represents an individual animal (squares = males; circles = females). (**C**) Representative H&E images of thyroid glands from *ATG7^control^* and *ATG7^TPO^* mice (*upper images*; *n* = 6–8 mice per group, 7.4 ± 1.0 mo) and immunofluorescence of Tg (gray), p62 (green) and BiP (red) in thyroid glands of *ATG7^control^* and *ATG7^TPO^* mice (*lower images*, *n* = 3–4 mice per group, 6.4 ± 0.1 mo).

We generated mice with thyrocyte-specific deletion of *HRD1* (disabling ERAD; Figure 1) or ATG7 (disabling autophagy; Figure 2). Hrd1 is the E3 ubiquitin ligase that functions, in conjunction with Sel1L, in retrotranslocation for ERAD (33). As thyrocytes comprise approximately 50% of the cells in the mouse thyroid gland (34), a ≥50% decrease in thyroidal Hrd1 protein (Figure 1A, quantified in graph) indicates essentially complete loss of Hrd1 in the thyrocytes of *HRD1^TPO^* mice. Such animals still had normal circulating thyroid hormone and TSH levels (Figure 1B) and normal thyroid histology (Figure 1C) but showed an increase of IRE1α and OS9 (Figure 1D, quantified in graph) that is typical of ERAD deficiency (35), along with a small increase of phosphorylated eIF2α — but without statistical increase of BiP, ERdj6 (Figure 1D), or spliced XBP1 (Figure 1E) — plus moderate swelling of the ER as detected by electron microscopy (Figure 1F). In *HRD1^TPO^* mice, we detected neither an increase in cleaved PARP (Figure 1G) nor thyroid cell death as judged by TUNEL staining (Figure 1H).

*ATG7* is the disease gene encoding an E1-like enzyme that facilitates LC3-I lipidation to form LC3-II, which is required for macroautophagy including ER-phagy (36). Thyroid tissue from *ATG7^TPO^* mice exhibited the expected increase of p62/SQSTM1 oligomers (detected by Western blotting, Figure 2A), as well as accumulation of LC3-I with diminished LC3-II, but with no apparent increase in ER stress markers like BiP or ERdj6 or enhanced PARP cleavage (Figure 2A). Such animals also maintain normal circulating thyroid hormone and TSH levels (Figure 2B). Whereas thyroid histology of *ATG7^TPO^* mice appeared normal (Figure 2C), abnormal accumulation of p62/SQSTM1 puncta was detected in most thyroid follicles (by immunofluorescence, Figure 2C), as has been reported in other autophagy-deficient tissues (37). Thus, the data in Figures 1 and 2 indicate that loss of either *HRD1* or *ATG7* is well tolerated in the thyroid gland in terms of tissue architecture and overall thyroid function.

### Overt cell biological defects in the thyrocytes of heterozygous TG^+/cog^ mice.

**Figure 3:**
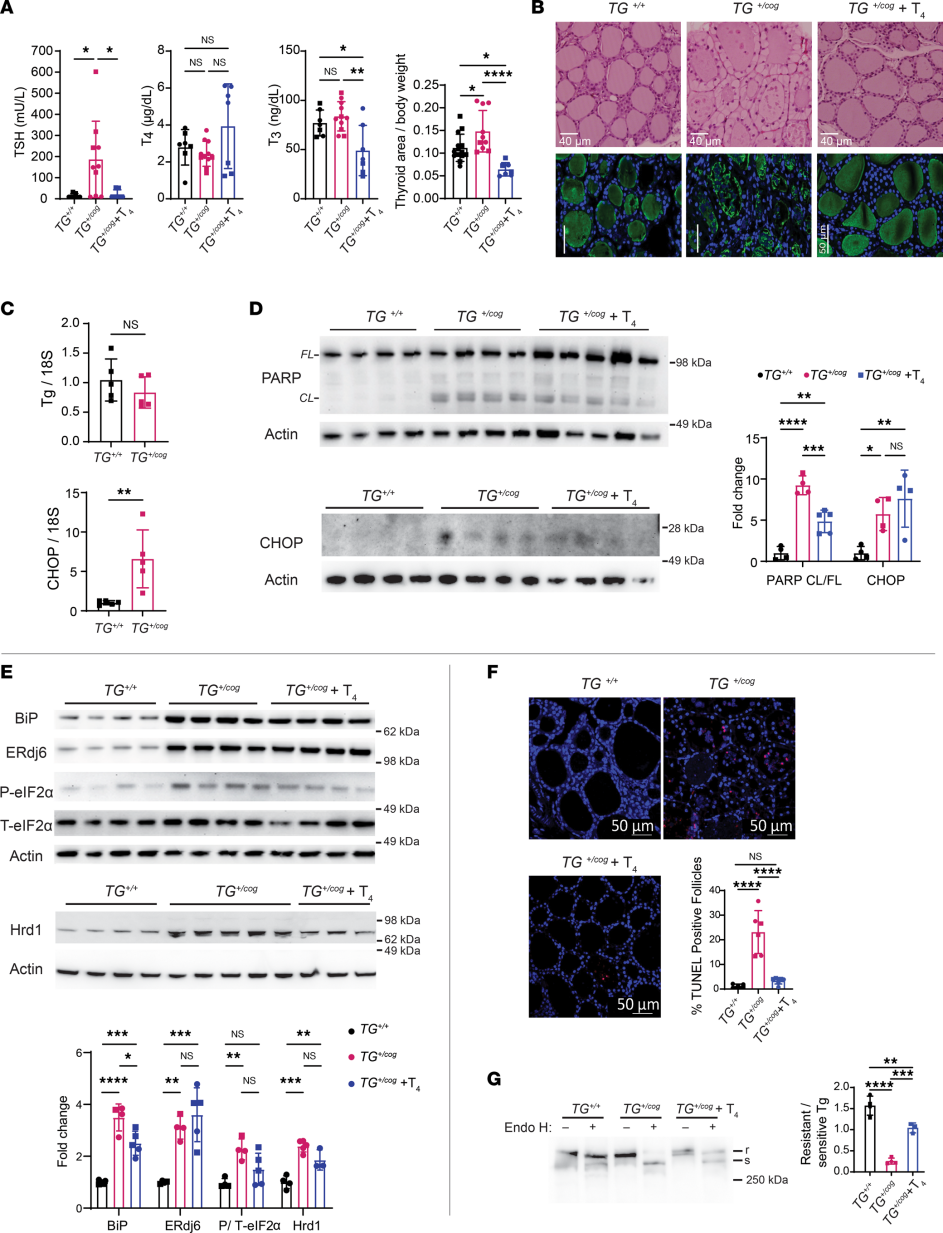
Cell biological defects in the thyrocytes of *TG^+/cog^* mice. (**A**) Serum TSH and total T_4_ and T_3_ levels (*n* = 7–10 mice/group) and thyroid gland size (*graph at right*, area in mm^2^ normalized to body weight; *n* = 7–14 mice/group) of *TG^+/+^*, *TG^+/cog^*, and T_4_-treated *TG^+/cog^* mice. (**B**) Representative H&E images (*upper images*, *n* = 4–8 mice/group; bar = 40 μm) and anti-Tg immunofluorescence (*lower images*, *n* = 2–4 mice/group; bar = 50 μm) of thyroid glands from *TG^+/+^*, *TG^+/cog^*, and T_4_-treated *TG^+/cog^* mice. (**C**) mRNA levels of Tg (*upper graph*) and CHOP (*lower graph*) in the thyroid glands of *TG^+/+^* and *TG^+/cog^* mice (normalized to 18S RNA; *n* = 5 mice/group). (**D**) Western blotting analysis of full-length (*FL*) and cleaved (*CL*) PARP (*upper*) and CHOP (*lower*) and quantitation (*graph at right*, *CL* PARP/*FL* PARP; CHOP protein normalized to actin) in the thyroid glands of *TG^+/+^*, *TG^+/cog^*, and T_4_-treated *TG^+/cog^* mice (*n* = 4–5 mice/group). (**E**) Western blotting analysis of BiP, ERdj6, phosphorylated eIF2α, total eIF2α, and Hrd1 (*blots above, quantitation below*, normalized to actin; except phosphorylated eIF2α normalized to total eIF2α) in the thyroid glands of *TG^+/+^*, *TG^+/cog^*, and T_4_-treated *TG^+/cog^* mice (*n* = 3–5 mice/group). (**F**) Representative TUNEL labeling (red) with DAPI counterstain (blue) and graph of TUNEL-positive follicles as a fraction of total thyroid follicles in thyroid sections of *TG^+/+^*, *TG^+/cog^*, and T_4_-treated *TG^+/cog^* mice (*n* = 5–7 mice/group). (**G**) Mock digest or endoglycosidase H (Endo H) digest followed by Tg Western blotting (*gel at left*) quantified (*bar graph at right*) from thyroid of *TG^+/+^*, *TG^+/cog^*, and T_4_-treated *TG^+/cog^* mice (*n* = 3–4 mice/group). All quantified data are mean ± SD; *P < 0.05, **P < 0.01, ***P < 0.001, ****P < 0.0001 (1-way ANOVA with Tukey’s post hoc test). Each dot represents an individual animal (squares = males; circles = females).

Genetic hypothyroidism caused by defective Tg is inherited in an autosomal-recessive manner (32), suggesting that heterozygosity may be inconsequential, although the homozygous condition leads to profound hypothyroidism with circulating levels of TSH elevated at least 3 orders of magnitude (13). Heterozygous *TG^+/cog^* mice exhibited subclinical hypothyroidism, defined by levels of circulating T_4_ and T_3_ in the normal range (Figure 3A), although the thyroid gland is stimulated by circulating TSH (Figure 3A). TSH elevation also promoted thyrocyte proliferation (Supplemental Figure 1; supplemental material available online with this article; https://doi.org/10.1172/jci.insight.169937DS1) and an increase in thyroid gland size (Figure 3A). This suggests a small but definite impairment in the efficiency of thyroid hormonogenesis in heterozygotes expressing misfolded mutant Tg. Moreover, in *TG^+/cog^* heterozygotes, the thyrocyte cytoplasm was markedly swollen with nuclei subjacent to the apical plasma membrane (Figure 3B). Further, the thyroid follicle lumen was dotted with hematoxylin-staining profiles, and immunostaining of Tg protein was patchy in comparison with the smooth luminal pattern seen in WT thyroid follicles (Figure 3B) — highly suggestive of epithelial cell shedding into the follicle lumen (as reported in *TG^cog/cog^* homozygotes; refs. 12, 13).

Thyroid tissue from heterozygous *TG^+/cog^* mice exhibited obvious stress responses. Despite no significant change in *TG* mRNA, *CHOP* mRNA was notably elevated in *TG^+/cog^* heterozygotes (Figure 3C). Additionally, there was increased PARP cleavage and increased CHOP protein (Figure 3D), along with dramatic elevation of BiP and ERdj6 and a notable increase of phosphorylated eIF2α (Figure 3E). Hrd1 was also elevated in *TG^+/cog^* heterozygotes (Figure 3E), as is known to occur under ER stress conditions (5). These features characterize proteotoxic ER stress with cell death, as demonstrated by a notable increase in thyroid follicles bearing TUNEL-positive cells (Figure 3F).

The fraction of endoglycosidase H–resistant Tg (one indicator of Tg that has advanced in protein trafficking beyond the ER) was distinctly low in *TG^+/cog^* mice (Figure 3G, lanes, quantified in graph). This is consistent with one fraction of Tg molecules comprising misfolded mutant protein that cannot escape the ER, along with a second fraction comprising WT Tg molecules that reach the follicle lumen for iodination followed by turnover as a consequence of TSH-stimulated endocytic Tg retrieval and lysosomal digestion for thyroid hormonogenesis (38). Together, these data indicate that heterozygous expression of misfolded mutant Tg triggers a consequential challenge to metabolic homeostasis as well as to cell survival in the thyroid gland.

Treatment with exogenous thyroid hormone is recommended for some patients with subclinical hypothyroidism (especially those with circulating TSH levels elevated by an order of magnitude; ref. 39). Exogenous T_4_ treatment suppresses TSH (the hormone that stimulates apical re-internalization of Tg from the thyroid follicle lumen; refs. 40–43). We observed that T_4_ treatment of *TG^+/cog^* heterozygotes sufficient to restore TSH to normal levels (Figure 3A, graph) blocked thyrocyte proliferation (Supplemental Figure 1) and led to remarkable improvement of thyroid histology, including smooth eosinophilic staining of the follicle lumen and a return of symmetric immunolabeling of endogenous Tg protein in the follicle lumen (Figure 3B) accompanied by a small decrease of cleaved PARP (Figure 3D, quantified in graph) and a decrease of TUNEL-positive thyrocytes (Figure 3F, quantified in graph). Although T_4_ treatment could not eliminate ER stress (Figure 3E), the data demonstrate that T_4_ treatment improves the morphological phenotype of the disease and additionally diminishes endocytic turnover of Tg, which is seen as an increase in endoglycosidase H–resistant Tg (Figure 3G, quantified in graph).

### Consequences of tissue-specific deficiency of autophagy or ERAD in thyrocytes of heterozygous TG^+/cog^ mice.

**Figure 4:**
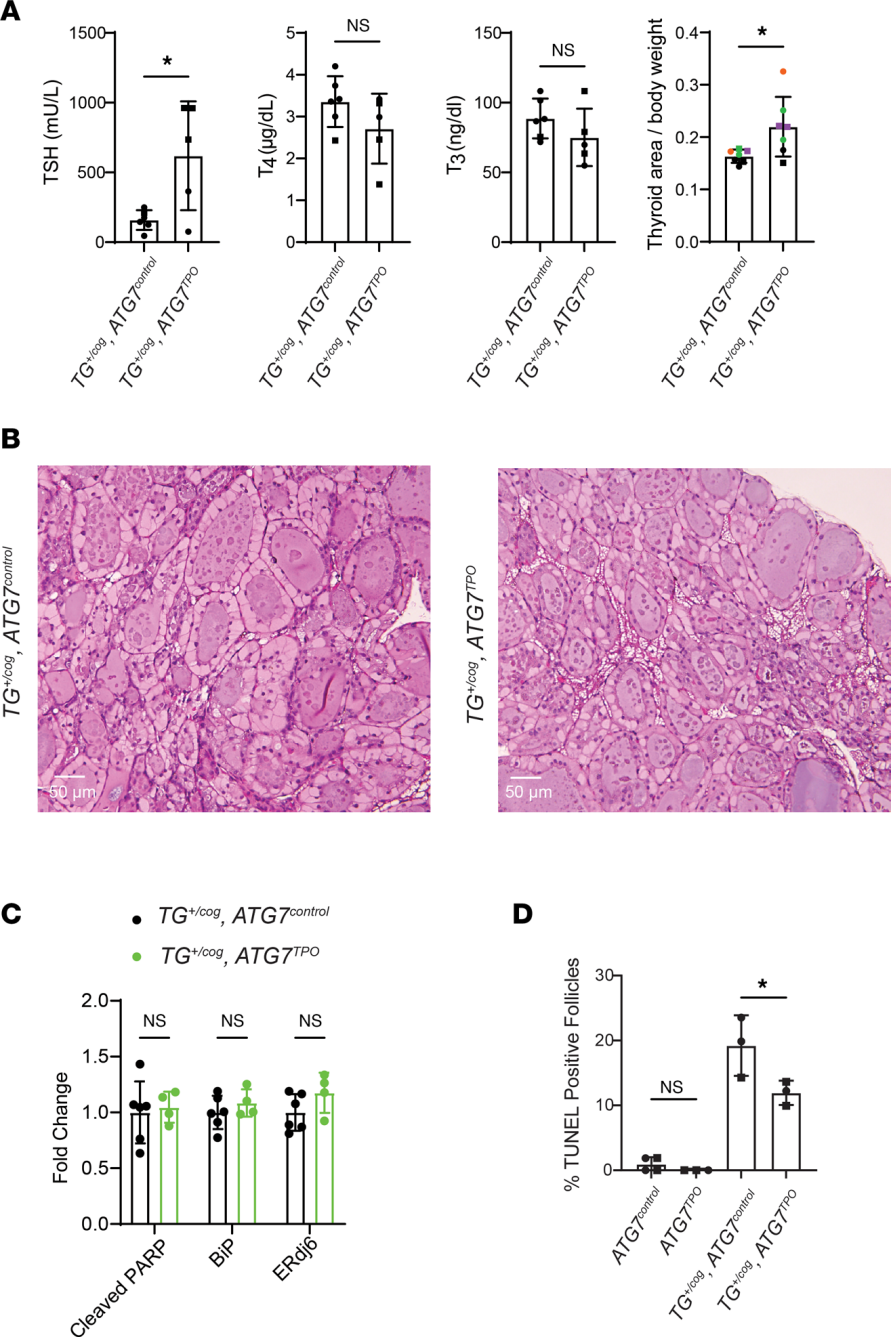
Tissue-specific autophagy deficiency in thyroid glands of mice expressing heterozygous mutant Tg. (**A**) Serum TSH and total T_4_ and T_3_ levels (first 3 graphs) and thyroid gland size (last graph) of *TG^+/cog^* *ATG7^control^* and *TG^+/cog^* *ATG7^TPO^* mice. (**B**) Representative H&E images of thyroid glands from *TG^+/cog^* *ATG7^control^* and *TG^+/cog^* *ATG7^TPO^* mice (*n* = 3 mice per group, 5.7 ± 0.4 mo). (**C**) Quantitation of cleaved PARP, BiP, and ERdj6 in the thyroid glands from *TG^+/cog^* *ATG7^control^* and *TG^+/cog^* *ATG7^TPO^* mice. (**D**) Quantitation of TUNEL-positive follicles (as a fraction of total follicles) in *ATG7^control^*, *ATG7^TPO^*, *TG^+/cog^* *ATG7^control^*, and *TG^+/cog^* *ATG7^TPO^* thyroid glands (*n* = 3–4 mice per group, 6.2 ± 0.5 mo). Graphs show mean ± SD; *P < 0.05 (unpaired 2-tailed Student’s *t* test in **A** and **C**, 1-way ANOVA with Tukey’s post hoc test in **D**); each dot represents an individual animal (squares = males; circles = females).

We proceeded to mate animals bearing *TG^+/cog^* heterozygosity into the *ATG7^TPO^* background. Even in the autophagy-deficient background, *TG^+/cog^* *ATG7^TPO^* mice had no decrease of T_4_ (or T_3_) levels, although they did exhibit an increase of circulating TSH as well as growth of the thyroid gland beyond that seen from *TG^+/cog^* heterozygosity alone (Figure 4A). With or without autophagy deficiency, profiles of dead thyrocytes were abundant in the thyroid follicles of *TG^+/cog^* heterozygotes, and the overall thyroid histology looked very similar from the 2 genotypes (Figure 4B). The thyroid glands of *TG^+/cog^* *ATG7^TPO^* mice showed no incremental change in BiP, ERdj6, or cleaved PARP over that in *TG^+/cog^* *ATG7^control^* animals (Figure 4C), and there was no further increase in TUNEL-positive thyroid follicles (Figure 4D). Thus, the 2 mutations together do not exhibit synthetic/synergistic lethality.

**Figure 5:**
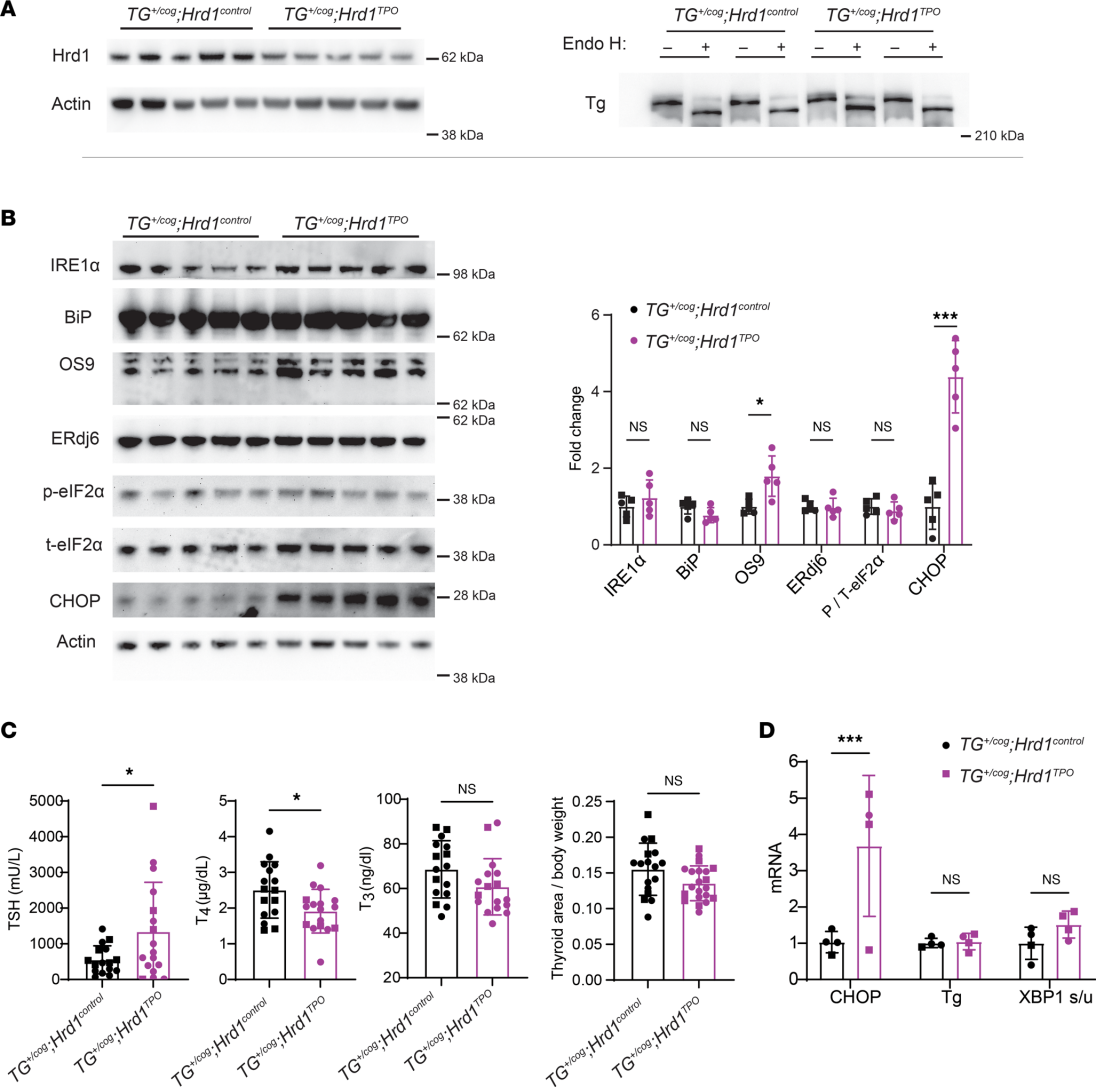
Tissue-specific ERAD deficiency in the thyroid gland of *TG^+/cog^* mice. (**A**) Western blotting analysis of Hrd1 and actin (*n* = 5 mice per group, *gel at left*) and Tg after mock digest or Endo H digest (*n* = 3 per group; 2 from each group in this gel, *right*) from *TG^+/cog^* *Hrd1^control^* and *TG^+/cog^* *Hrd1^TPO^* thyroid glands. (**B**) Western blotting analysis of IRE1α, BiP, OS9, ERdj6, phosphorylated eIF2α (p-eIF2α), total eIF2α (t-eIF2α), and CHOP (*blots at left*) and quantitation (*at right* normalized to actin; except phosphorylated eIF2α normalized to total eIF2α) in *TG^+/cog^* *Hrd1^control^* and *TG^+/cog^* *Hrd1^TPO^* thyroid glands (*n* = 5 mice per group). (**C**) Serum TSH and total T_4_ and T_3_ levels (left 3 panels; *n* = 16–17 mice per group) and thyroid gland size (normalized to body weight, rightmost panel; *n* = 17–21 mice per group) of *TG^+/cog^* *Hrd1^control^* and *TG^+/cog^* *Hrd1^TPO^* mice. (**D**) CHOP and Tg mRNA levels (normalized to 18S RNA) and XBP1 spliced/unspliced mRNA ratio in *TG^+/cog^* *Hrd1^control^* and *TG^+/cog^* *Hrd1^TPO^* thyroid glands (*n* = 4 mice per group). Graphs in **B**–**D** show mean ± SD; *P < 0.05, ***P < 0.001 (unpaired 2-tailed Student’s *t* test); each dot represents an individual animal (squares = males; circles = females).

Intracellular turnover of misfolded mutant Tg has been reported to be sensitive to both kifunensine and MG132 — 2 drugs that can affect early recognition, and ultimate proteolysis, of ERAD substrates (44, 45). Because more recent reports indicate that p97/VCP (which extracts retrotranslocated, ubiquitylated ERAD substrates from the cytosolic face of the ER membrane for delivery to proteasomes) can be selectively inhibited by CB5083 (6, 46, 47), we tested the impact of a 6-hour incubation with CB5083 in cells expressing recombinant mutant (unsecretable) cog Tg and found that the drug increased intracellular cog Tg by approximately 75% (Supplemental Figure 2, A and B). These data are consistent with the hypothesis that at least a portion of misfolded Tg protein may be degraded by ERAD. With this in mind, animals bearing *TG^+/cog^* heterozygosity were mated into the *HRD1^TPO^* background that confers thyroidal ERAD deficiency (Figure 5A). Despite dramatic ER stress responses induced by misfolded mutant Tg alone (Figure 3E), further increases of several ER stress markers were not observed in the double mutant, although CHOP protein was notably increased (Figure 5B, quantified in graph). Moreover, the *HRD1^TPO^* background did not noticeably change that a fraction of Tg was able to acquire endoglycosidase H resistance (Figure 5A). Nevertheless, the combination of these 2 genetic defects led to a small but significant decrease of circulating T_4_ levels (no change in circulating T_3_) accompanied by a further increase in circulating TSH (Figure 5C). Interestingly, however, unlike *TG^+/cog^* heterozygosity alone, the further increase in TSH seen in the double mutant was not accompanied by increased thyrocyte proliferation (Supplemental Figure 3) or by any further enlargement of the thyroid gland (Figure 5C). Consistent with CHOP protein, thyroidal *CHOP* mRNA was also distinctly increased in the double mutants (Figure 5D).

**Figure 6:**
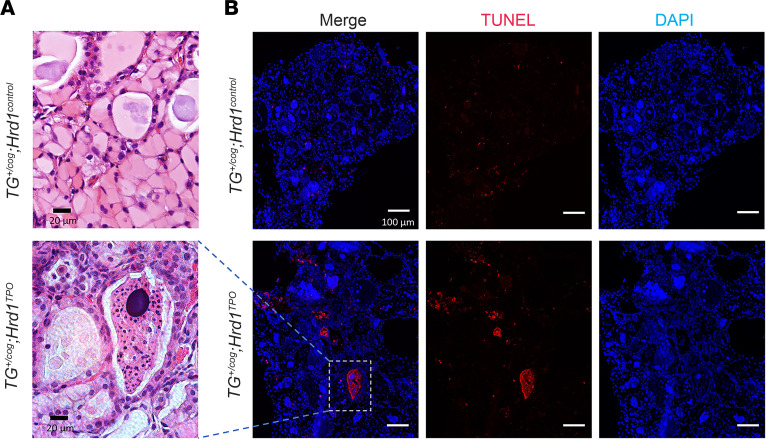
Degenerating thyroid follicles in the thyroid of *TG^+/cog^* *Hrd1^TPO^* mice. (**A**) Representative H&E images from *TG^+/cog^* *Hrd1^control^* thyroid and degenerating thyroid follicle from *TG^+/cog^* *Hrd1^TPO^* mice (*n* = 12–20 mice per group; additional images in Supplemental Figure 4). Black scale bar = 20 μm. (**B**) Representative TUNEL labeling (DAPI counterstain) from *TG^+/cog^* *Hrd1^control^* thyroid tissue showing individual dead cells and *TG^+/cog^* *Hrd1^TPO^* thyroid (*n* = 14–19 mice per group) showing follicle degeneration including the region shown in **A**. White scale bar = 100 μm.

Increased CHOP expression has been linked to tissue injury (such as reported in kidney; ref. 48); thus, we looked more closely at the histological level and discovered several additional thyroid abnormalities caused by defective ERAD in *TG^+/cog^* heterozygotes. Specifically, beyond the thyrocyte swelling observed from the *TG^+/cog^* defect alone (Figure 6A), defective ERAD resulted in patchy swaths of follicle collapse and degeneration (Figure 6A). These degenerating thyroid follicles took on a variety of morphologies, but all lacked preservation of a luminal cavity (Supplemental Figure 4). In the double-mutant genotype, we used serial sections to perform TUNEL staining and verified that the collapsed follicles observed histologically were composed entirely of dead cells with extruded chromatin (Figure 6B).

**Figure 7:**
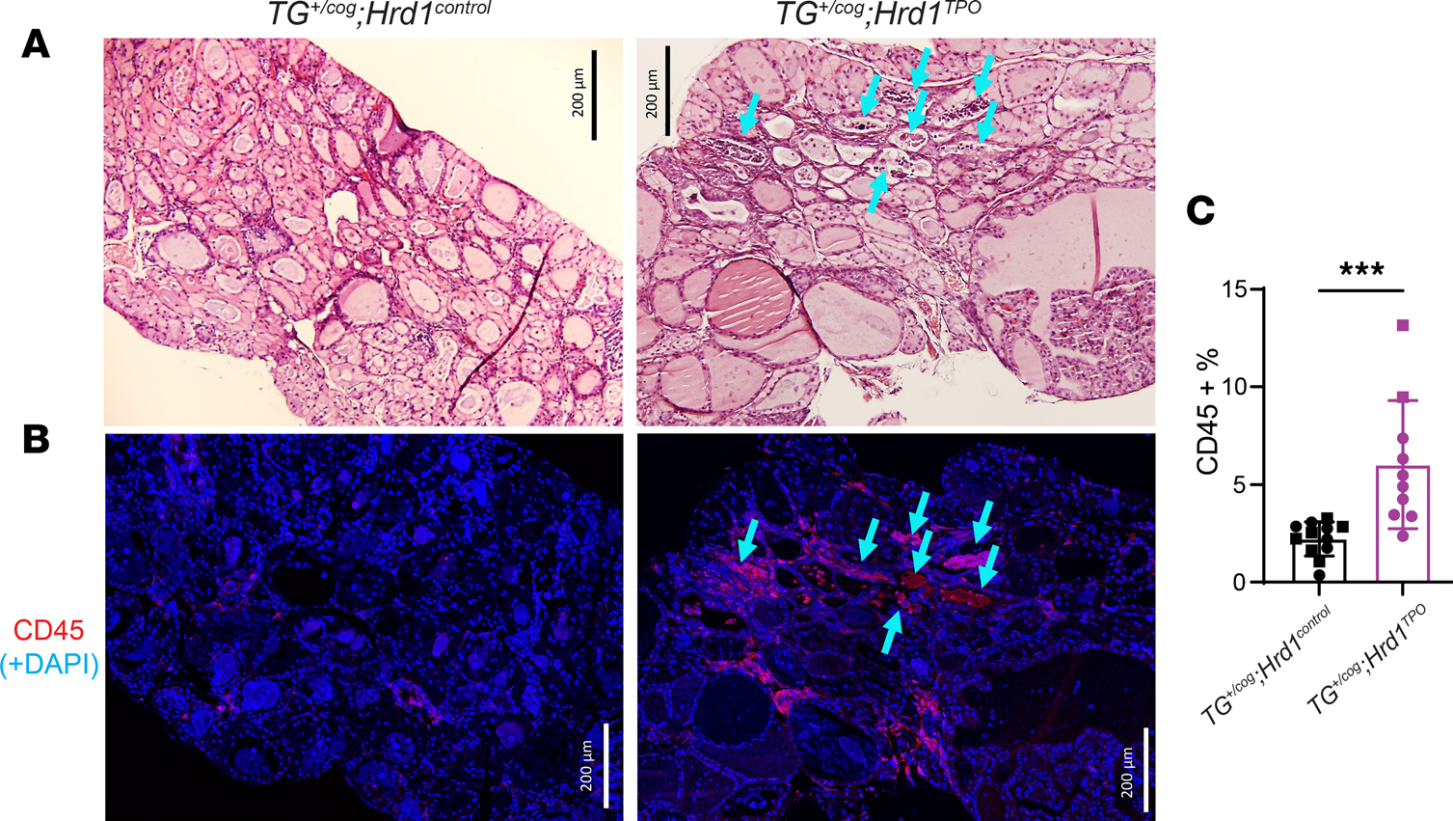
Infiltration of bone marrow–derived CD45^+^ cells in the thyroid glands of *TG^+/cog^* *Hrd1^TPO^* mice. (**A**) Representative H&E images of thyroid glands from *TG^+/cog^* *Hrd1^control^* and *TG^+/cog^* *Hrd1^TPO^* mice (*n* = 12–20 mice per group). (**B**) Representative anti-CD45 immunofluorescence of thyroid glands from *TG^+/cog^* *Hrd1^control^* and *TG^+/cog^* *Hrd1^TPO^* mice (*n* = 10–12 mice per group). The double mutant was stained in serial sections with blue arrows to toggle between histology and CD45 immunostained areas. Size bars are indicated. (**C**) Quantitation of CD45^+^ cells as a fraction of total thyroid gland nuclei (squares = males; circles = females). Data are shown as mean ± SD; ***P < 0.001 (unpaired 2-tailed Student’s *t* test).

**Figure 8:**
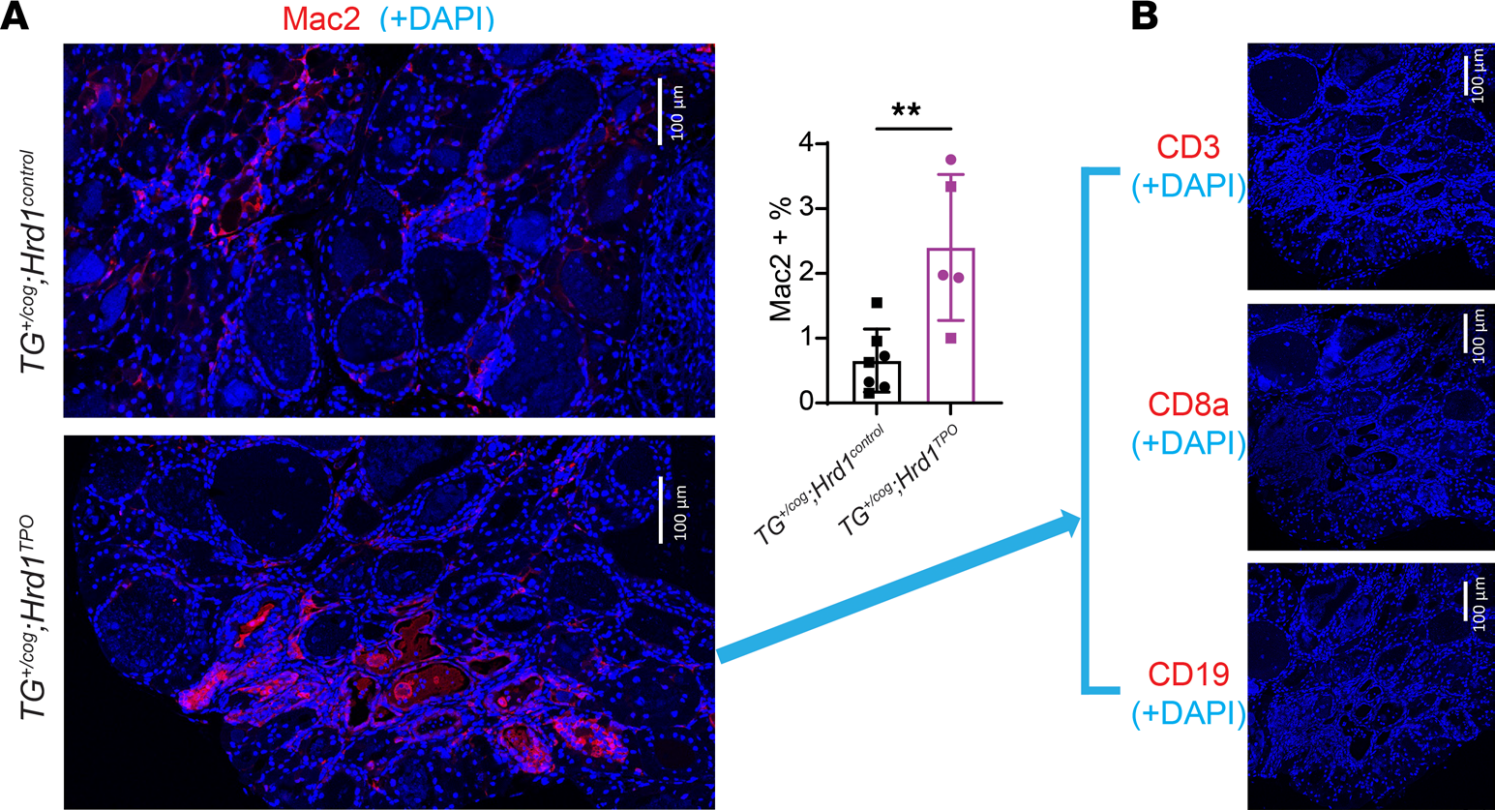
Macrophages positive for Mac2 in the thyroid glands of *TG^+/cog^* *Hrd1^TPO^* mice. (**A**) Representative anti-Mac2 immunofluorescence in *TG^+/cog^* *Hrd1^control^* and *TG^+/cog^* *Hrd1^TPO^* thyroid glands (*n* = 5–7 mice per group; quantified in bar graph at right as Mac2-positive cells as a fraction of total cells per field). (**B**) Representative anti-CD3, anti-CD8a, and anti-CD19 immunofluorescence showing absence of those antigens in the thyroid glands of *TG^+/cog^* *Hrd1^TPO^* mice (*n* = 2–5 animals per group, with spleen tissue used as a positive control as shown in Supplemental Figure 6). Data are shown as mean ± SD; **P < 0.01 (unpaired 2-tailed Student’s *t* test).

Bone marrow–derived CD45^+^ cells are known to represent a small fraction of the cellular composition of the WT thyroid gland (34), and in *TG^+/cog^* thyroid (Figure 7A) such cells could also be detected (Figure 7B). However, in *TG^+/cog^ HRD1^TPO^* double mutants (using serial sections), the thyroid gland attracted an increase of bone marrow–derived CD45^+^ cells (Figure 7B; quantified in Figure 7C) to regions near areas of follicle collapse and degeneration (Figure 7, A and B, arrows) — and this was not noted in thyroid tissue of *TG^+/cog^* *ATG7^TPO^* mice (Supplemental Figure 5). Further thyroid tissue analysis in *TG^+/cog^* *HRD1^TPO^* double mutants suggested that these bone marrow–derived cells were primarily macrophages, which immunostained positively for Mac2 (Figure 8A, quantified in graph) but not for CD3, CD8a, or CD19 (Figure 8B — spleen tissue was used as a positive control to confirm the reactivity of these latter antibodies in Supplemental Figure 6). Additionally, these animals did not develop anti-Tg autoantibodies (Supplemental Figure 7). However, in the histology of some of the thyroid glands of *TG^+/cog^* *HRD1^TPO^* mice at 4.5 months of age, we could detect “holes” representing degenerated regions in place of normal thyroid tissue (Supplemental Figure 8). Together, the data suggest that ERAD (but not autophagy) deficiency confers a synthetic/synergistic lethality in thyrocytes expressing misfolded mutant Tg, which promotes whole-follicle death and tissue injury with an increased number of bone marrow–derived macrophages that are attracted to clean up tissue debris.

## Discussion

The thyroid gland is proving an exceptionally valuable model of cytotoxicity under conditions of perturbed ER proteostasis (12–14). Dead thyroid epithelial cells are shed into the lumen of thyroid follicles (unconnected to any duct system), wherein dead thyrocytes slowly disintegrate over time. Thus, in this tissue it is exceptionally easy to detect cell death. Heterozygous humans bearing mutations encoding misfolded mutant Tg are quite common (30, 31). Although such mutations are not expected to lower circulating thyroid hormone levels (Figure 3), these mutants cause dramatic ER stress and stress response (14, 29), and we suspect that most if not all such mutants trigger extensive (albeit clinically unsuspected) thyrocyte cell death.

Some fraction of WT Tg protein almost certainly misfolds and may need to be proteolytically disposed of; nevertheless, neither tissue-specific genetic deficiency of Hrd1-dependent ERAD function nor loss of ATG7-dependent autophagy function triggers any significant change in either circulating thyroid hormone levels or (the more sensitive) circulating TSH levels — and neither deficiency yields any morphological or biochemical signs of increased thyrocyte cell death (Figures 1 and 2). These findings are also broadly consistent with a previous study of thyroidal *ATG5*-deficient mice that were found to exhibit normal thyroid morphology, thyroid gland weight, and circulating T_4_ and TSH levels (although in a different strain background, such animals were said to exhibit an increased susceptibility to thyroid epithelial cell death) (49). Ongoing ERAD and autophagy, together, comprise essential housekeeping functions in all eukaryotic cells; however, recent evidence suggests that loss of one central proteolytic pathway can be at least partially compensated by presence of the other, such as in the case of ERAD deficiency increasing Ire1α (35) that in turn can stimulate autophagy (8).

Although neither autophagy nor Sel1L/Hrd1-ERAD appear essential to keeping thyroid function operational, heterozygous mutant Tg offers misfolding in the ER in much greater abundance than the smaller amount that occurs for WT Tg. Certainly, in untreated/undertreated humans and animals with biallelic mutant *TG*, ER stress–mediated thyrocyte cell death is dramatic (12, 13). Although this has never previously been examined in heterozygotes to our knowledge, we find that entrapment of mutant Tg in the ER is sufficient to cause marked cell swelling from cytoplasmic ER expansion, diminished thyroid function as demonstrated by an increase of TSH that is required to sustain normal circulating thyroid hormone levels, as well as upregulation of CHOP, cleavage of PARP, and readily detectable TUNEL-positive cells (Figure 3). The overall organismal phenotype can be described as subclinical hypothyroidism, which means that most patients bearing heterozygous *TG* mutations would escape detection by the medical system. However, it is important to note that the elevated TSH of subclinical disease contributes to the tissue pathology, as administration of exogenous thyroid hormone to suppress TSH results in suppression of *TG* gene expression (50) as well as suppression of apical endocytosis (38, 40–43), and these effects contribute to suppressing ER swelling, PARP cleavage, and thyrocyte death, as well as allowing for a homogenous reaccumulation of endoglycosidase H–resistant Tg in the thyroid follicle lumen (Figure 3).

Misfolded mutant Tg might be degraded by ER autophagy (51), thus contributing to the autosomal-recessive inheritance of disease attributed to mutant *TG* (such as by limiting the extent of ER stress, cell death, and thyroid dysfunction). Although plausible, the current analyses offer little support for the hypothesis that ER autophagy effectively prevents accumulation of misfolded Tg or its adverse downstream consequences in vivo. Remarkably, other than a further TSH increase with no change in circulating thyroid hormone levels, autophagy deficiency superimposed upon *TG^+/cog^* heterozygosity has incremental impact neither on thyroid gland histology nor on thyroid cell death (Figure 4).

At present, there is strong albeit circumstantial evidence that mutant Tg protein may be degraded in part by ERAD (44, 45, 52) (Supplemental Figure 2). Interestingly, Hrd1 protein is upregulated in the thyroid glands of animals bearing heterozygous mutant Tg (Figure 3E) as has been reported to occur in other cells under ER stress conditions (5). Moreover, thyroidal ERAD deficiency exacerbates the hypothyroidism of *TG^+/cog^* heterozygotes (circulating T_4_ levels lower and TSH higher), further increases the induction of CHOP, and limits further growth of the thyroid gland (Figure 5) at least in part by blocking incremental thyrocyte proliferation (Supplemental Figure 3) — and most dramatically, yields patchy areas of whole-follicle death and collapse (Figure 6 and Supplemental Figure 4), which leads to infiltration of bone marrow–derived cells (Figure 7) that are predominantly macrophages (Figure 8). The resulting tissue degeneration can ultimately be seen as “holes” in the thyroid gland (Supplemental Figure 8), consistent with the notion that macrophages have entered primarily to clean up debris from dead tissue. These observations include phenotypes that we believe have not been reported previously in other tissues as a consequence of Sel1L/Hrd1 ERAD deficiency — or even in the thyroid gland in which misfolded mutant Tg protein is also expressed. We expect that because of the massive synthesis of misfolded Tg protein, the absence of thyroidal Hrd1-mediated ERAD function converts *TG^+/cog^* heterozygosity into a disease that no longer behaves as a “recessive” condition.

Several possible mechanisms may contribute to whole-follicle cell death seen in the thyroid glands of *TG^+/cog^* *HRD1^TPO^* mice. First, loss of Hrd1 ERAD function is likely to be significantly detrimental to ER homeostasis — even in an otherwise WT thyroid gland, ER swelling is already evident by electron microscopy (Figure 1F). Second, as treatment with T_4_ lowers circulating TSH and thus renders the thyroid gland more quiescent (Figure 3), the converse situation of decreased circulating T_4_ levels with high levels of circulating TSH stimulates the thyroid gland further (Figure 5), which may render active follicles more susceptible to toxicity. Finally, deficiency of Hrd1 ERAD function may result in a failure to dispose of a subset of misfolded Tg molecules that are more highly proteotoxic. Although each of these mechanisms may contribute in part, and more work is needed to distinguish between them, the cumulative effect is that the condition of mutant *TG* heterozygosity depends upon Hrd1-dependent ERAD function to avoid adding insult to injury, limiting follicular demise, and preventing subclinical disease from evolving into frank hypothyroidism.

In summary, our data highlight that heterozygous expression of misfolded Tg, so common in the human population, involves ongoing, large-scale ER stress–mediated cell death. This condition is relatively benign only because of various compensatory responses linked to ER homeostatic function, specifically, Hrd1-mediated ERAD (Figures 5–8), as well as increased circulating TSH (Figure 3) accompanied by stimulated thyrocyte proliferation (Supplemental Figure 1) to help maintain normal thyroid hormone production. Several other tissues may lack a potent feedback control (equivalent to TSH) that can allow for organ regrowth to replace tissue that has been lost by ER stress–mediated cell death. Curiously, however, in the thyroid gland, this feedback stimulation, despite being a necessary compensatory response to maintain tissue mass, actually makes a contribution to the toxic phenotype by stimulating the gland to express more of the misfolded mutant secretory protein. This reflects a pathophysiologic cost-benefit analysis, with cost to individual cells, yet benefit to the entire organism.

## Methods

### Primary antibodies.

We used rabbit anti-Tg (ab156008, Abcam; 365997, Santa Cruz Biotechnology), rabbit anti-PARP (9542, Cell Signaling Technology); mouse anti-Actin (66009-1-Ig, Proteintech); rabbit anti-BiP was previously described (53); rabbit anti-ERdj6 (2940, Cell Signaling Technology); rabbit anti–phospho-eIF2α (Ser51) (9721, Cell Signaling Technology) and total eIF2α (9722, Cell Signaling Technology); rabbit anti-Hrd1 (13473-1-AP, Proteintech); rabbit anti-IRE1 (3294, Cell Signaling Technology); rabbit anti-OS9 (ab109510, Abcam); rabbit anti-CD45 (ab10558, Abcam); rat anti-Mac2 (14-5301-81, Invitrogen); rat anti-CD3 (ab11089, Abcam); rat anti-CD8a (14-0808-82, Invitrogen); rat anti-CD19 (14-0194-82, Invitrogen); rabbit anti-P62 (Enzo, BML-PW9860); guinea pig anti-P62 (MBL, PM066); rabbit anti-LC3 (2775, Cell Signaling Technology); rabbit anti-Ki67 [SP6] (ab16667, Abcam); and rabbit anti-CHOP (sc-575, Santa Cruz Biotechnology).

### Mice.

All mice were in a C57BL6/J background. *TG^cog/cog^* mice (C57BL6/J) were obtained from The Jackson Laboratory. *Hrd1*-floxed mice (54), *Atg7*-floxed mice (55), and *Tpo^Cre^* mice (56, 57) were used as previously described. *Tpo^Cre^* mice were back-bred with *Hrd1^fl/fl^* or *Atg7^fl/fl^* mice to generate thyrocyte-specific *Hrd1^TPO^* and *Atg7^TPO^* mice, respectively. Control littermates were heterozygous deletion of *Hrd1* mice (*Hrd1^TPO/+^*, simply labeled *Hrd1^control^*) for *Hrd1^TPO^* mice and heterozygous deletion of *Atg7* mice (*Atg7^TPO/+^*) plus *Atg7^fl/fl^* mice (both simply labeled *Atg7^control^*) for *Atg7^TPO^* mice. *TG^+/cog^* *Hrd1^TPO^* and *TG^+/cog^* *Atg7^TPO^* animals were generated by additional backcrossing, with control littermates including heterozygous *TG^+/cog^* *Hrd1^TPO/+^* (*TG^+/cog^* *Hrd1^control^*) or heterozygous *TG^+/cog^* *Atg7^TPO/+^* plus *TG^+/cog^* *Atg7^fl/fl^* genotypes (*TG^+/cog^* *Atg7^control^*). All animal experiments performed were approved by the University of Michigan Institutional Animal Care and Use Committee. Both male and female animals were used (males represented as squares and females as circles). Unless otherwise indicated, mice were 4.5–5.5 months old. When used, T_4_ treatment of *TG^+/cog^* mice was performed by supplementation of drinking water with T_4_ (1 μg/mL, T2501, MilliporeSigma) for at least the last 3 months.

### Cell culture and transfection.

293T cells (ATCC CRL-3216, cultured in DMEM plus 10% bovine calf serum) were transiently transfected with plasmid encoding cog-Tg, using Lipofectamine 2000 transfection reagent according to the manufacturer’s instructions. After 18 hours, transfected cells were trypsinized, replated, and treated for 6 hours ± CB5083 (10 μM, Apexbio).

### Serum measurements.

Serum total TSH, T_4_, and T_3_ concentrations were measured using radioimmunoassays as previously described (58, 59). Serum anti-Tg autoantibody was measured using ELISA as previously described (60) (data presented as optical density at 405 nm).

### Thyroid gland size measurement.

Thyroid gland area (mm^2^, normalized to body weight in grams) was measured and quantified as previously described (12).

### Preparation and immunostaining of thyroid sections.

Mouse thyroids were quickly dissected and immersion-fixed in 10% formalin, paraffin-embedded, sectioned, and stained with hematoxylin and eosin (Vector Laboratories). For immunofluorescence, thyroid sections (6 μm) were deparaffinized in Citrisolv, then rehydrated using a graded ethanol series, followed by antigen retrieval in citrate buffer, blocking in 1.5% goat serum, incubation with primary antibodies (overnight, 4°C) and Alexa Fluor–conjugated secondary antibodies (Invitrogen A11073, A11001, A21422, A21428, A21245; Jackson ImmunoResearch 712-606-153; 1 hour, room temperature), counterstaining with ProLong Gold and DAPI (Invitrogen), and imaging with Nikon A1 confocal microscope or Leica STELLARIS 8 FALCON confocal microscope. Quantification of CD45^+^ cells in the proportion of total cells was performed using AIVIA Artificial Intelligence-guided Software. Immunohistochemistry of Ki67 used VECTASTAIN-ABC (Vector Laboratories) with 40× objective image capture (Olympus EX51 Microscope). Quantitation of Ki67-positive nuclei as a fraction of total thyroid nuclei per field, or Mac2-immunostained cells as a fraction of total thyroid nuclei per field, was performed using Imaris software (version 7.7.2).

### PCR.

Total RNA isolation from the mouse thyroids was performed using an RNeasy Plus Kit (QIAGEN), followed by cDNA synthesis using High-Capacity cDNA Reverse Transcription Kits (Applied Biosystems). For real-time PCR, Radiant Green Hi-ROX qPCR Kit (Alkali Scientific) was used on a StepOnePlus PCR system (Thermo Fisher Scientific) or CFX Opus 384 Real-Time PCR System (Bio-Rad). Gene expression was normalized to 18S RNA, with primers as follows: Tg (forward: 5′-TGATCTGATCCACAACTACAACAG-3′; reverse: 5′-ATTCCAGTCCTGTCTCAGCC-3′), CHOP (forward: 5′-CCTGAGGAGAGAGTGTTCCAG-3′; reverse: 5′-GACACCGTCTCCAAGGTGAA-3′), and 18S (forward: 5′-GGCGTCCCCCAACTTCTTA-3′; reverse: 5′-GGGCATCACAGACCTGTTATTC-3′). Spliced and unspliced XBP1 were amplified with a single primer pair (forward: 5′-TGGCCGGGTCTGCTGAGTCCG-3′; reverse: 5′-GTCCATGGGAAGATGTTCTGG-3′) using the GoTaq Green Master Mix Kit (Promega), with products resolved by 3% agarose gel (18S RNA used as a control) and bands quantified using ImageJ (NIH).

### Western blotting.

Mouse thyroid glands or 293T cells were homogenized in RIPA buffer (150 mM NaCl, 25 mM Tris-HCl pH 7.6, 1% NP-40, 1% sodium deoxycholate, 0.1% SDS, Thermo Fisher Scientific) supplemented with protease and phosphatase inhibitor cocktails (Thermo Fisher Scientific), followed by sonication. Protein concentration was measured by BCA assay (Thermo Fisher Scientific). Protein lysates were heated in NuPAGE LDS sample buffer with 50 mM dithiothreitol at 95°C for 5 minutes, resolved by SDS-PAGE, and transferred to nitrocellulose membranes. The membranes were blocked with 5% milk, immunoblotted with the indicated antibodies and appropriate HRP-conjugated secondary antibody (Bio-Rad, 1721019,1706516), and visualized by enhanced chemiluminescence. Bands’ intensities were quantified using ImageJ.

### TUNEL labeling.

The In Situ Cell Death Detection Kit, Fluorescein (Roche), was used for TUNEL staining. Sections were counterstained and mounted with ProLong Gold and DAPI. Follicles bearing TUNEL-positive thyroid cells were quantified as a fraction of total TUNEL-positive follicles in each field (with multiple fields per thyroid gland).

### Endoglycosidase H digest.

Thyroid homogenates were boiled in endo H denaturing buffer (New England Biolabs) at 95°C for 5 minutes, then were either mock-digested or digested with endoglycosidase H (1,000 units) (New England Biolabs) at 37°C for 1 hour.

### Electron microscopy.

Mouse thyroids were quickly dissected and immersion-fixed in 2.5% glutaraldehyde. The tissue was washed in 100 mM Na cacodylate containing 2 mM CaCl_2_, postfixed with 0.25% OsO_4_, washed, stained with 0.5% uranyl acetate, washed and dehydrated in a graded ethanol series, incubated 30 minutes in propylene oxide, and infiltrated and polymerized in Araldite. Sections of 70 nm on Formvar-coated copper grids, poststained with 1% lead citrate, were examined under a JEOL JEM-1400 transmission electron microscope.

### Statistics.

Comparisons between 2 groups were made by unpaired 2-tailed Student’s *t* test. Comparisons of more than 2 groups were made by 1-way ANOVA with Tukey’s post hoc test. All statistical analyses were conducted with GraphPad Prism. Data are represented as mean ± SD; *P* < 0.05 was considered significant.

### Study approval.

All animal experiments performed with mice were in compliance with and approved by the University of Michigan Institutional Animal Care and Use Committee (PRO00009936).

### Data and materials availability.

All data and methods for this manuscript are included directly in the paper and Supplemental Data 1. Materials are freely available upon request.

## Author contributions

XZ and PA designed experiments; XZ, CY, XHL, MT, and MSL performed experiments; assistance was provided by HZ and DL; DF, SR, LQ, and YT provided key reagents; XZ wrote the Methods section; and PA supervised the work. PA and XZ wrote the manuscript; all authors reviewed, edited, and approved the manuscript.

## Supplementary Material

Supplemental data

Supplemental data set 1

## Version 1 06/22/2023

Electronic publication
