# Supplementary material for: Perturbation of endoplasmic reticulum proteostasis triggers tissue injury in the thyroid gland
Source: JCI Insight. 2023 Jun 22;8(12):e169937. doi: 10.1172/jci.insight.169937 (PMC10371246; doi:10.1172/jci.insight.169937)
Supplement: Supplemental data [file jciinsight-8-169937-s016.pdf]

Zhang et al., Supplemental Figure Legends

Supplemental Fig. S1. Thyroid cell proliferation in mice bearing heterozygous *TG* mutation is inhibited by thyroxine treatment. Quantitation Ki67-positive nuclei (as a fraction of total nuclei in the thyroid section) from untreated $TG^{+/cog}$ mice and those treated with T_4 (each color represents a different animal; squares = males, circles = females; each data point represents quantitation from an independent thyroid section). Low-level Ki67-positive nuclei in $TG^{+/+}$ mice is shown as a reference. Data are shown as mean ± s.d.; $^{***}p < 0.001$, $^{****}p < 0.0001$.

Supplemental Fig. S2. Effect of ERAD inhibition on misfolded intracellular Tg. **A**. 293T cells were either untransfected (no plasmid) or transfected to express cog-Tg. At 18 h post-transfection, cells were trypsinized, divided in two equal portions, and replated, and were then either treated with CB5083 (10 µM) for 6 h or vehicle alone (“–"). Cell lysates were collected and analyzed by SDS-PAGE and immunoblotting with anti-Tg. **B**. Quantitation of cog-Tg (normalized to actin). Each dot represents an independent experiment (n = 5) with mean ± s.d.; $^{**}p < 0.01$.

Supplemental Fig. S3. Cell proliferation in the thyroid glands of mice bearing heterozygous *TG* mutation ± thyroid-specific ERAD deficiency. Quantitation of Ki67-positive nuclei (as a fraction of total nuclei in the thyroid section) from untreated from $TG^{+/cog}$;$Hrd1^{control}$ and $TG^{+/cog}$;$Hrd1^{TPO}$ mice (n=3 mice per group; each color represents a single mouse; squares = males; circles = females). Data are shown as mean ± s.d.

Supplemental Fig. S4. H&E images of degenerating thyroid follicles from $TG^{+/cog}$;$Hrd1^{TPO}$ mice. The images highlight various morphologies of thyroid follicle degeneration. Size bars for three of the images are 20 µm, but for the larger collapsed follicles in the lower left image, the size bar is larger.

Supplemental Fig. S5. Immunofluorescence of CD45 (red) in thyroid glands from *$TG^{+/cog}$;$ATG7^{control}$* and *$TG^{+/cog}$;$ATG7^{TPO}$* mice. DAPI counter-stain (blue), n=2-5 mice per group, 6.1 ± 0.5 mo. The negative results for these genotypes used identical immunostaining conditions to the positive results shown in Fig. 6B.

Supplemental Fig. S6. Anti-CD3, anti-CD8a and anti-CD19 immunofluorescence of mouse spleen (DAPI counter-stain). The spleen was used as a positive control for the studies of Figure 8B.

Supplemental Fig. S7. Testing for serum anti-Tg autoantibodies in *$TG^{+/+}$*, *$TG^{+/cog}$*, *$TG^{+/cog}$;$Hrd1^{control}$* and *$TG^{+/cog}$;$Hrd1^{TPO}$* mice. Each dot represents an individual animal (squares = males; circles = females; n=11-17 mice per group). The positive control was from the serum of NOD.H-2^{h4} mice treated with 0.05% NaI in the drinking water (1); with mean ± s.d.; ****$p < 0.0001$.

1. Braley-Mullen H, Sharp GC, Medling B, and Tang H. Spontaneous autoimmune thyroiditis in NOD.H-2h4 mice. *J Autoimmun.* 1999;12(3):157-65.

Supplemental Fig. S8. A slice through the entire mouse thyroid gland. H&E staining (tiled composite image) highlights regions cleared of debris from degenerated follicles of *$TG^{+/cog}$;$Hrd1^{TPO}$* thyroid (center).

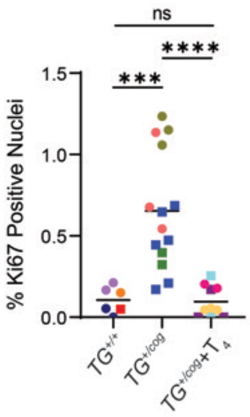

Zhang et al., Supplemental Fig. S1

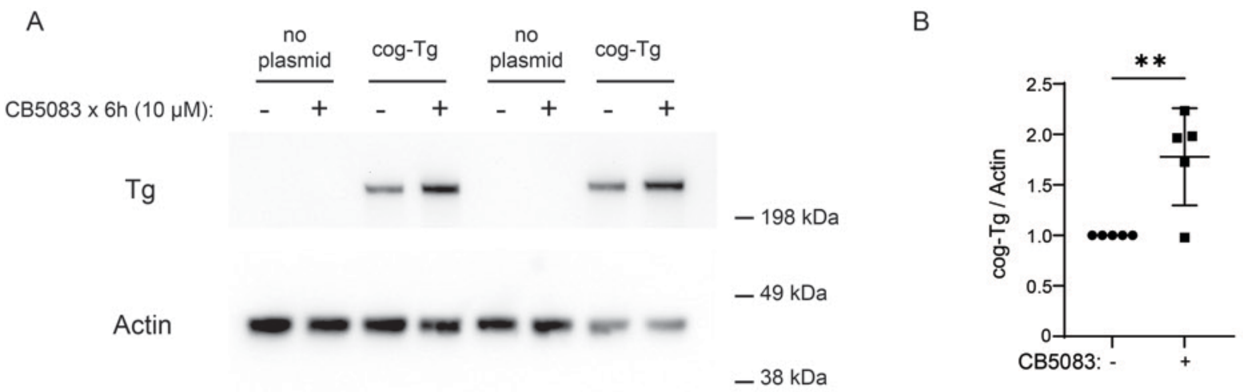

Zhang et al., Supplemental Fig. S2

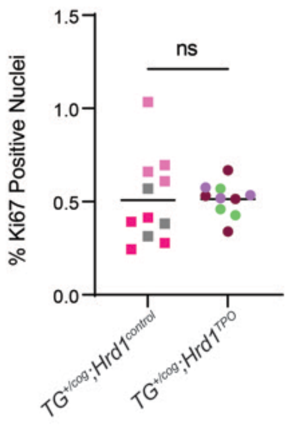

Zhang et al., Supplemental Fig. S3

H&E images showing different stages of thyroid follicle degeneration in $TG^{+/cog}$;$Hrd1^{TPO}$ mice

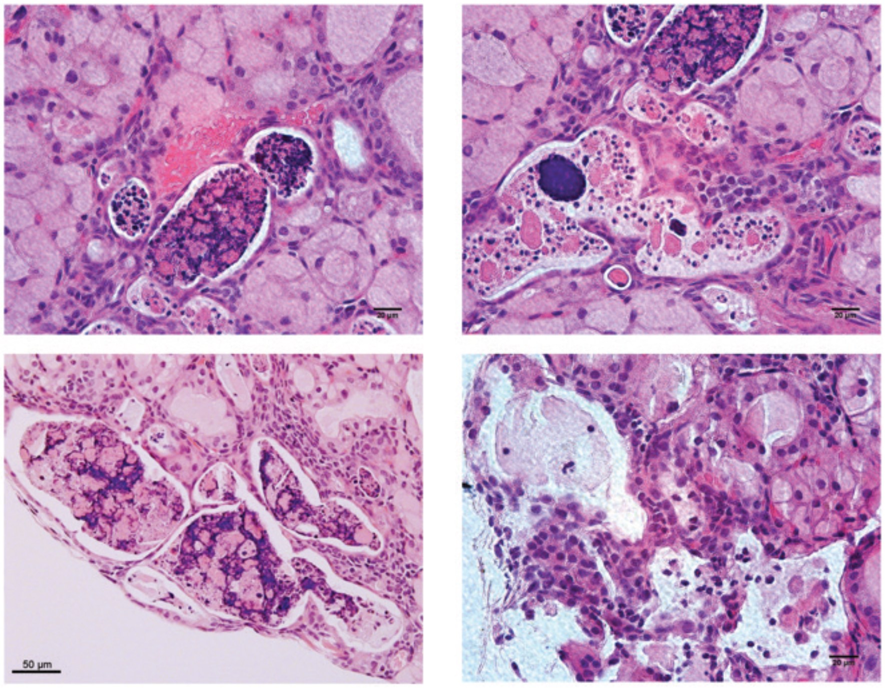

Zhang et al., Supplemental Fig. S4

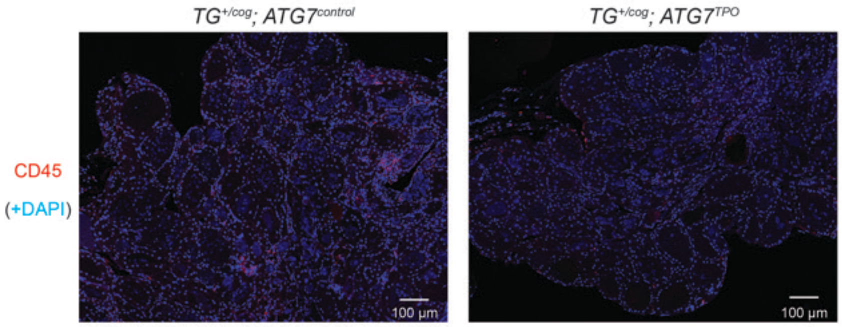

Zhang et al., Supplemental Fig. S5

Spleen (Positive control for Main Fig. 8)

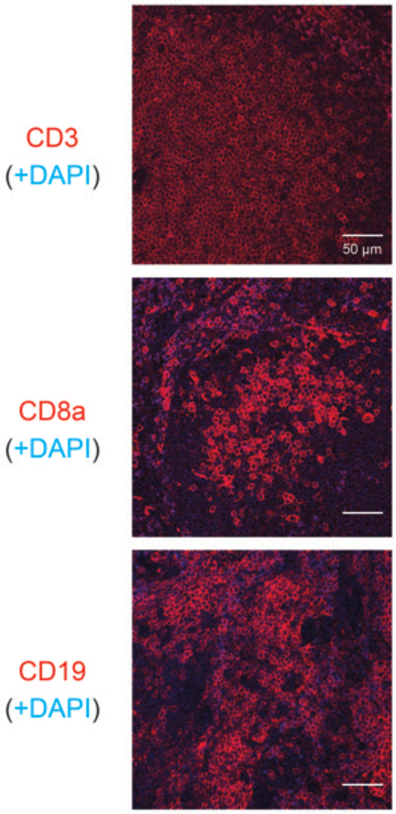

Zhang et al., Supplemental Fig. S6

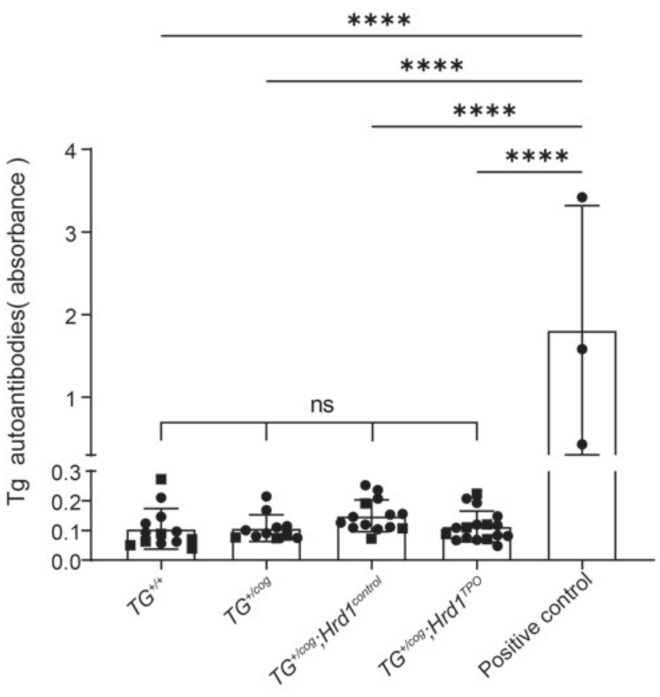

Zhang et al., Supplemental Fig. S7

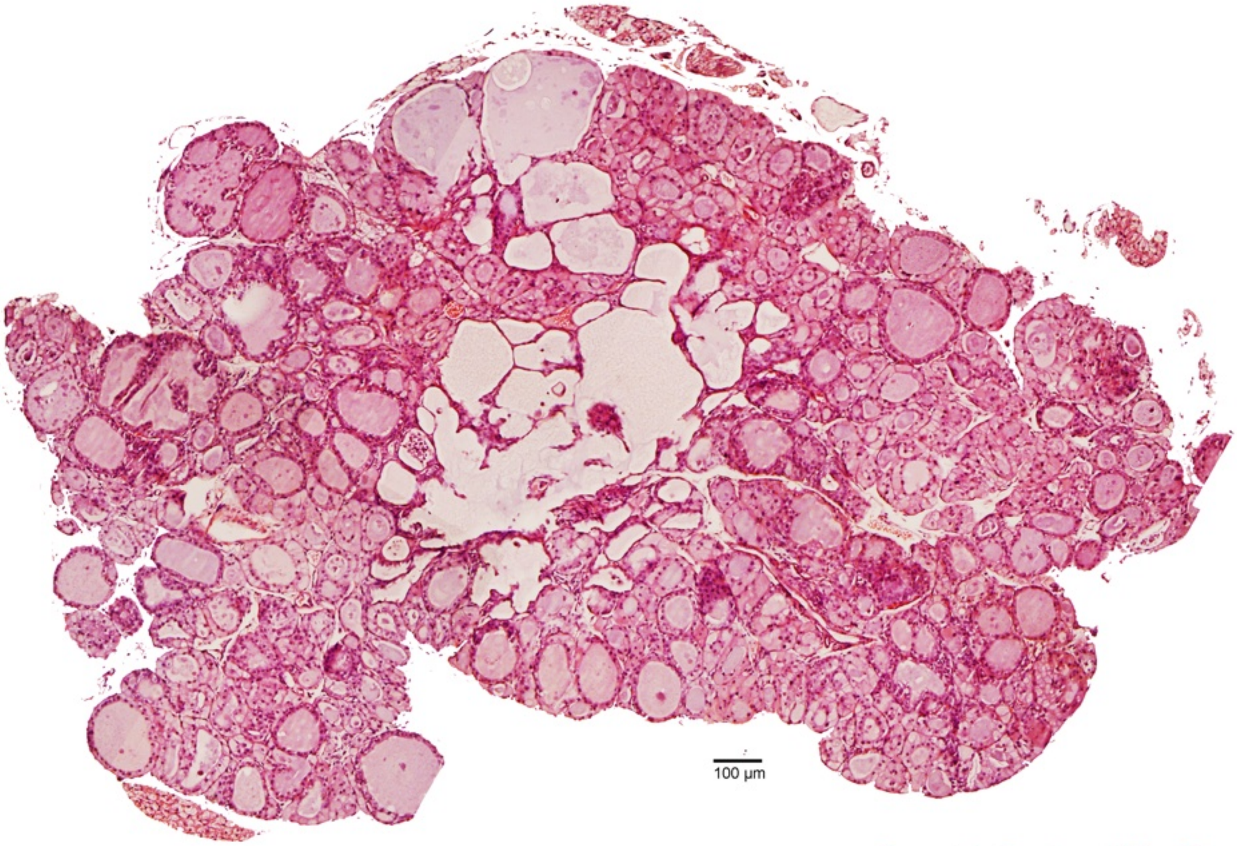

Zhang et al., Supplemental Fig. S8
