# Supplementary material for: Perturbation of endoplasmic reticulum proteostasis triggers tissue injury in the thyroid gland
Source: JCI Insight. 2023 Jun 22;8(12):e169937. doi: 10.1172/jci.insight.169937 (PMC10371246; doi:10.1172/jci.insight.169937)
Supplement: Supplemental data set 1 [file jciinsight-8-169937-s017.pdf]

Fig. 1A

Lanes in the figure are numbered here.

Lane 1-5: *Hrd1*^{control}

Lane 6-10: *Hrd1*^{TPO}

Lane: 1 2 3 4 5 6 7 8 9 10

Bright field image

Hrd1

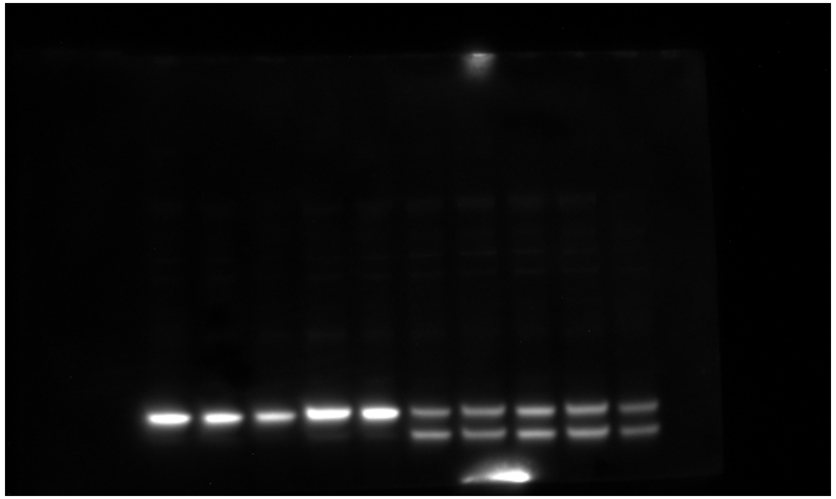

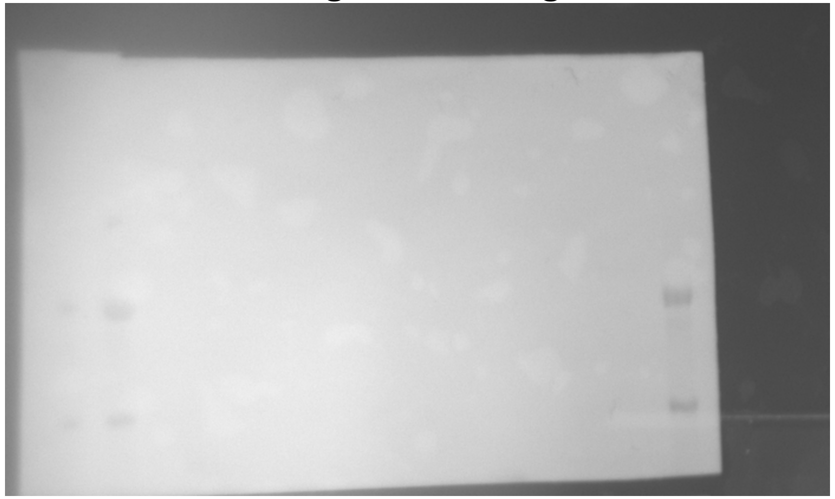

Lane: 1 2 3 4 5 6 7 8 9 10

Actin

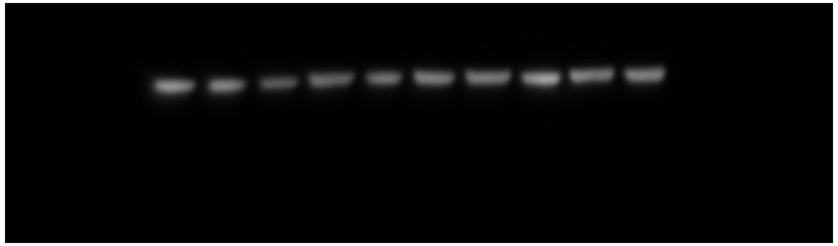

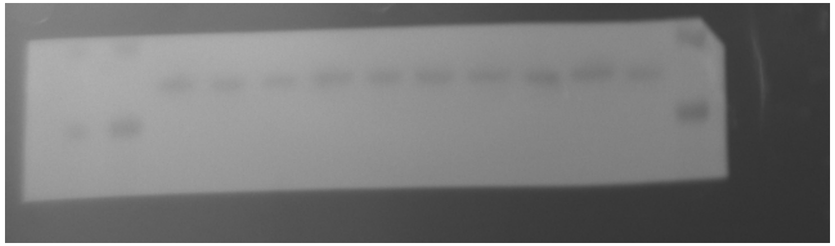

Quantification:

Lane	Hrd1	Actin	Hrd1/Actin	mean of control	fold change
1	32188778	12313856	2.61402911	3.058761311	0.854604
2	27504092	10177216	2.70251629		0.883533
3	23099808	7468160	3.09310566		1.011228
4	34760994	10236224	3.39588055		1.110214
5	30294747	8684736	3.48827495		1.140421
6	13928688	11305152	1.23206552		0.402799
7	15151664	11948432	1.26808806		0.414576
8	15690384	13304064	1.17936775		0.38557
9	15100880	12033792	1.25487294		0.410255
10	9717392	11033120	0.88074742		0.287943

Fig. 1B

Mouse tag#	*Genotype*	TSH (mU/L)	T_4 (µg/dL)	T_3 (ng/dL)
17732	*control*	99	1.09	68.1
17725	*control*	98	2.37	79.5
17818	*control*	104	2.13	79.7
17910	*control*	134	3.64	95.4
17628	*control*	<10	3.23	92.5
17689	*control*	26	1.49	84.0
17737	*control*	<10	2.88	85.5
17727	*control*	48	0.91	47.8
17919	*control*	32	2.60	69.0
17918	*control*	66	0.49	43.6
17914	*control*	117	3.85	71.7
17687	$Hrd1^{TPO}$	43	3.35	74.4
17681	$Hrd1^{TPO}$	96	2.42	64.7
17714	$Hrd1^{TPO}$	89	3.91	83.7
17905	$Hrd1^{TPO}$	227	2.28	71.7
17923	$Hrd1^{TPO}$	271	3.51	89.4
17917	$Hrd1^{TPO}$	157	3.26	79.7
17916	$Hrd1^{TPO}$	<20	3.00	66.8
17913	$Hrd1^{TPO}$	116	2.79	71.7
17911	$Hrd1^{TPO}$	110	3.14	84.6
17716	$Hrd1^{TPO}$	14	1.24	66.2
17735	$Hrd1^{TPO}$	<10	2.93	81.6
17693	$Hrd1^{TPO}$	<10	2.43	86.4

Fig. 1C

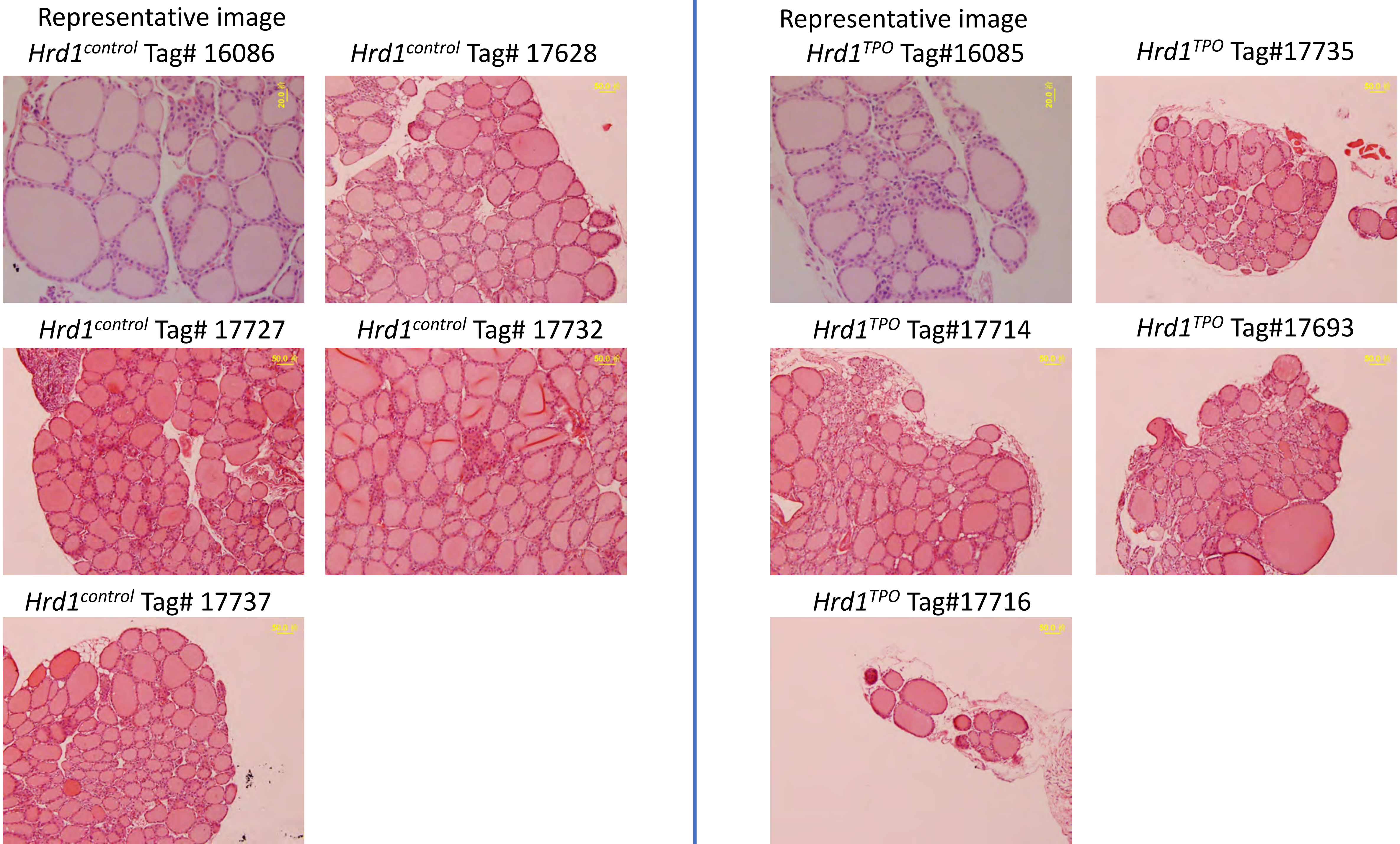

Representative image
Hrd1control Tag# 16086
Hrd1control Tag# 17628
Hrd1control Tag# 17727
Hrd1control Tag# 17732
Hrd1control Tag# 17737
Representative image
Hrd1TPO Tag#16085
Hrd1TPO Tag#17735
Hrd1TPO Tag#17714
Hrd1TPO Tag#17693
Hrd1TPO Tag#17716

Fig. 1D Lanes in the figure are numbered here.

Lane 1-5: *Hrd1*^{control}

Lane 6-10: *Hrd1*^{TPO}

Quantification:

	lane	bands intensity	actin	/actin	mean of control	fold change
ERdj6	1	17223456	10709680	1.608214	1.192014353	1.34915647
	2	13936640	10768592	1.294193		1.08571959
	3	12492016	9504992	1.314258		1.102552533
	4	18193712	21278189	0.85504		0.717307165
	5	16390368	18450024	0.888366		0.745264242
	6	24481360	25123482	0.974441		0.81747452
	7	27692336	23282805	1.18939		0.997798408
	8	25318624	17400621	1.455041		1.22065762
	9	37478418	21228037	1.765515		1.48111892
	10	32146385	13640832	2.356629		1.977014236
	lane	bands intensity	t-eIF2a	p/t-eIF2a	mean of control	fold change
p-eIF2a	1	6724912	21736896	0.309378	0.42307306	0.731263179
	2	10432112	23381408	0.446171		1.054596216
	3	9765280	20533968	0.475567		1.124077998
	4	11124144	28282624	0.393321		0.929675808
	5	10794400	21987728	0.490928		1.160386799
	6	16076384	26622111	0.603873		1.427350098
	7	16596960	26948112	0.615886		1.455743406
	8	15240176	26972880	0.565018		1.335510448
	9	13932736	31782047	0.438384		1.03618946
	10	16041264	21308304	0.752817		1.779403048
	lane	bands intensity	actin	/actin	mean of control	fold change
BiP	1	14041232	10709680	1.311079	0.993885543	1.319144428
	2	11500192	10768592	1.067938		1.074508351
	3	10223888	9504992	1.075634		1.082250897
	4	14323888	21278189	0.673172		0.677313733
	5	15527632	18450024	0.841605		0.846782592
	6	18034384	25123482	0.71783		0.72224594
	7	22261680	23282805	0.956143		0.962024786
	8	15741136	17400621	0.904631		0.910196058
	9	19826352	21228037	0.93397		0.939715961
	10	19515408	13640832	1.430661		1.439462654
IRE1	1	5680338	10709680	0.530393	0.371949395	1.42598129
	2	4086495	10768592	0.379483		1.020253733
	3	3328652	9504992	0.3502		0.941527005
	4	5354559	21278189	0.251645		0.676558243
	5	6421079	18450024	0.348026		0.935679729
	6	13069755	25123482	0.520221		1.398632963
	7	17467559	23282805	0.750234		2.017033268
	8	13624747	17400621	0.783003		2.105134467
	9	15211125	21228037	0.716558		1.926493919
	10	6894716	13640832	0.505447		1.358913036
OS9	1	7676163	10709680	0.71675	0.527344409	1.359168648
	2	3536575	10768592	0.328416		0.622772754
	3	6468619	9504992	0.68055		1.290522173
	4	7333467	21278189	0.344647		0.65355228
	5	10449347	18450024	0.56636		1.073984146
	6	17054114	25123482	0.678812		1.28722654
	7	18628296	23282805	0.800088		1.517202269
	8	26512102	17400621	1.52363		2.889249644
	9	29489514	21228037	1.389178		2.634289092
	10	25663755	13640832	1.881392		3.567672614

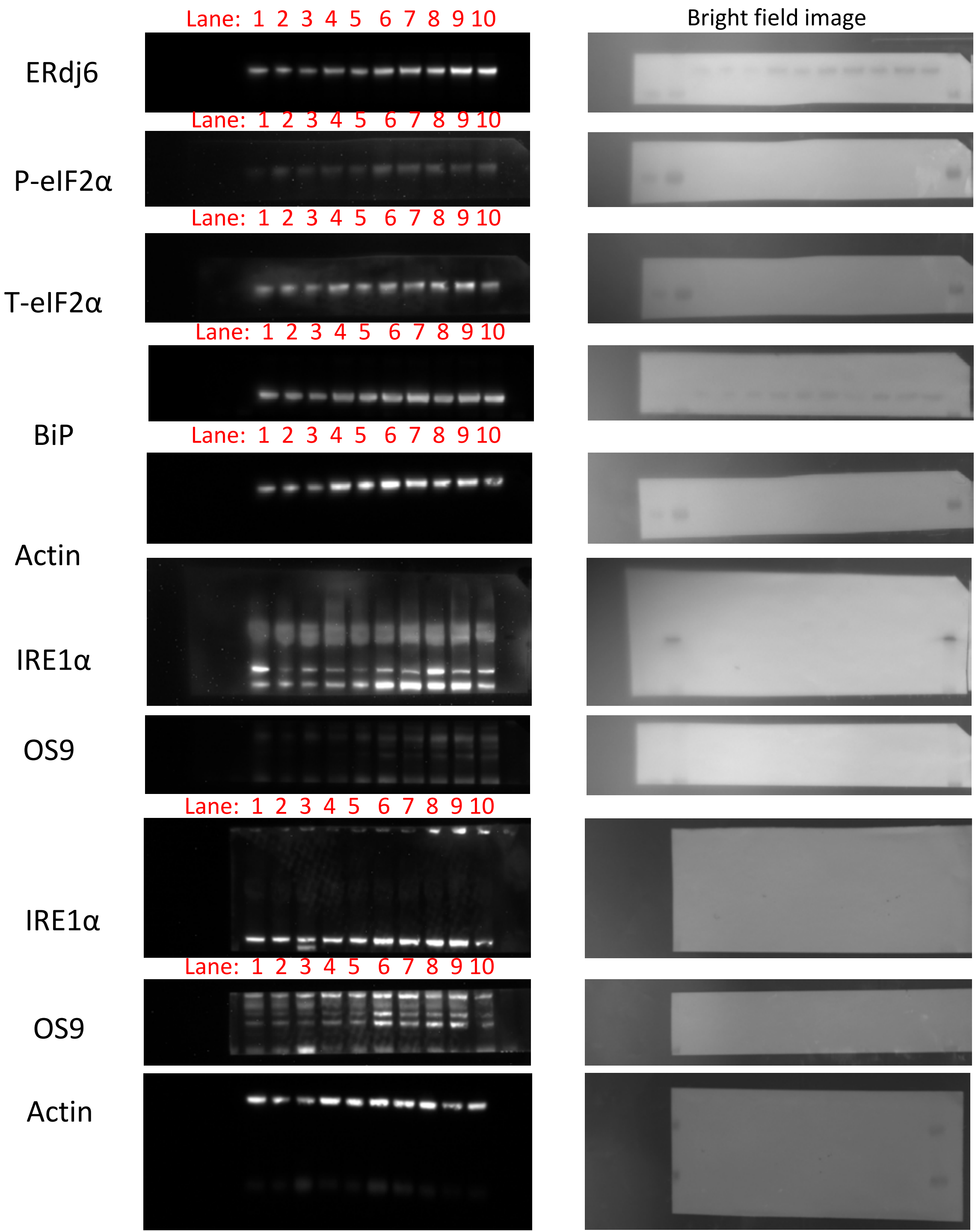

Fig. 1E Lanes in the figure are numbered here.

Lane 1-4: *Hrd1*^{control}

Lane 5-10: *Hrd1*^{TPO}

Lane: 1 2 3 4 5 6 7 8 9 10

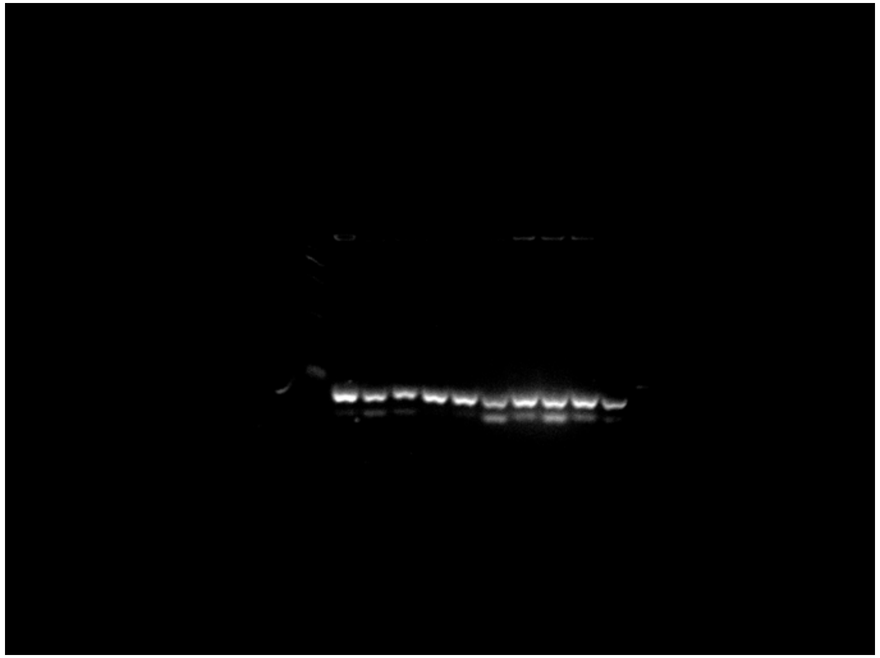

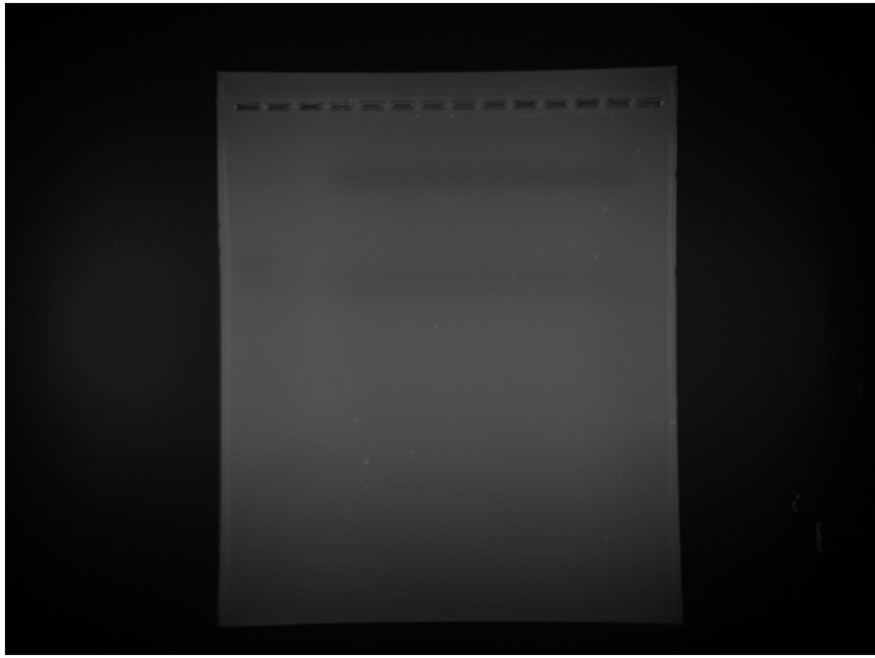

Quantification:

lane	unspliced	spliced	spliced/unspliced	mean of control	fold change
1	52904355	2542722	0.048062622	0.078675539	0.610896633
2	37674052	6075413	0.161262532		2.049716269
3	35928957	3361047	0.093547024		1.189022986
4	48184876	570026	0.011829978		0.150364112
5	48590052	3076550	0.063316458		0.804779469
6	42219575	22644990	0.536362339		6.817396462
7	60035509	19936157	0.332072757		4.220787842
8	58410295	31176523	0.53375048		6.78419861
9	52149941	13921769	0.266956563		3.393132952
10	23481167	1939574	0.082601261		1.04989762

Fig. 1F

Representative image

Hrd1^{control} Tag# 16086

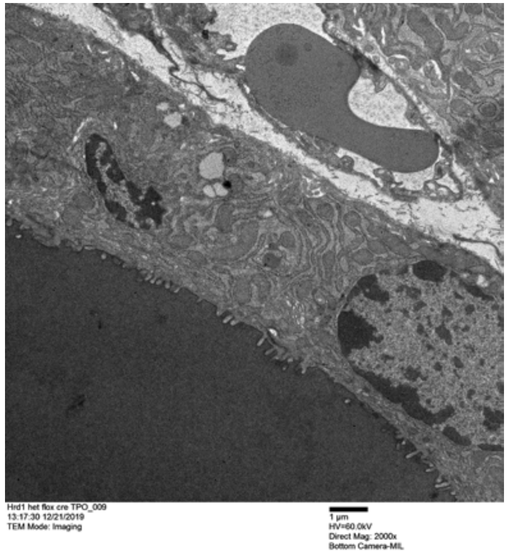

Hrd1^{control} Tag# 16086

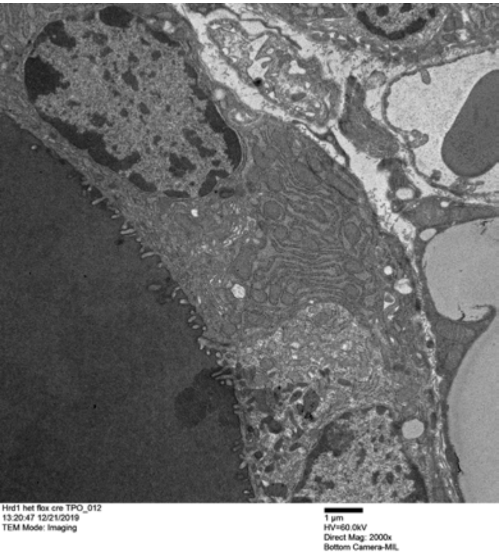

Hrd1^{control} Tag# 16086

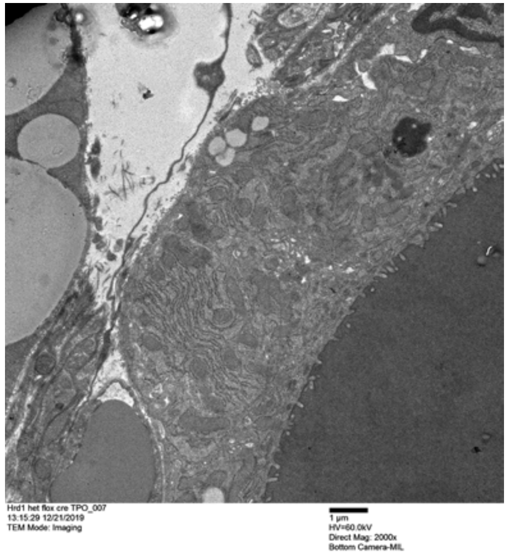

Representative image

Hrd1^{TPO} Tag#16085

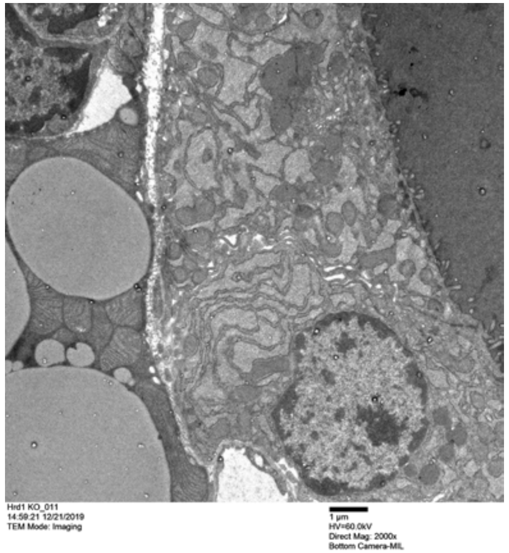

Hrd1^{TPO} Tag#16085

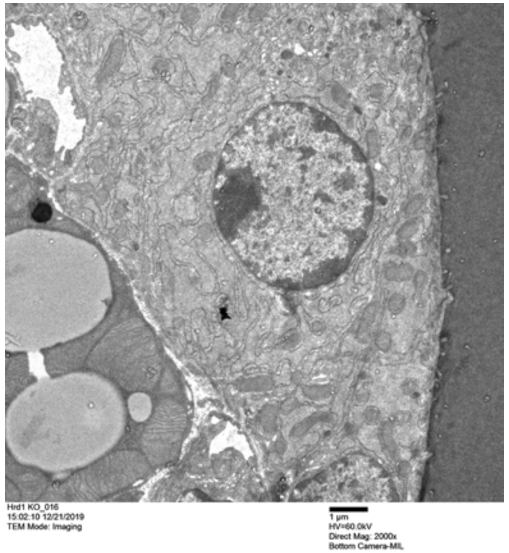

Hrd1^{TPO} Tag#16085

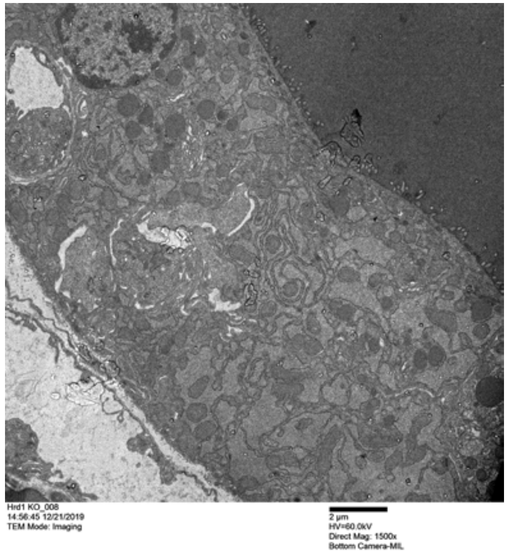

Hrd1^{TPO} Tag#15973

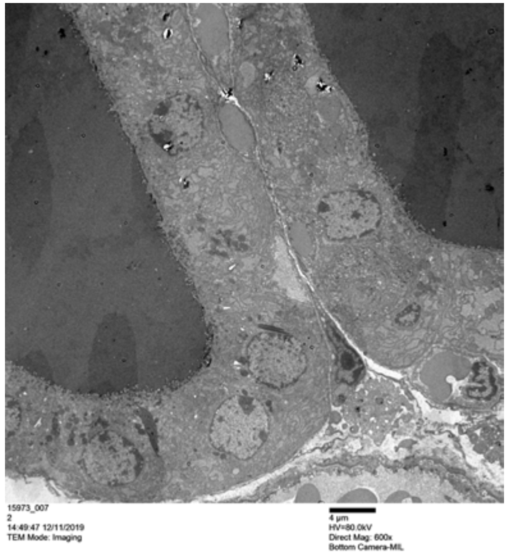

Hrd1^{TPO} Tag#15973

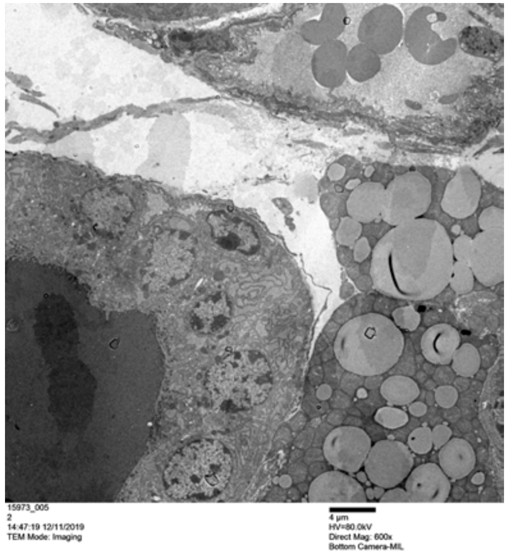

Hrd1^{TPO} Tag#15973

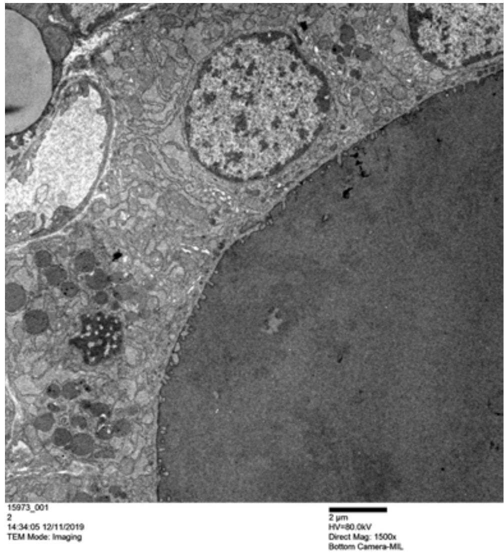

Fig. 1G Lanes in the figure are numbered here.

Lane 1: *Hrd1*^{control}

Lane 2: *Hrd1*^{TPO}

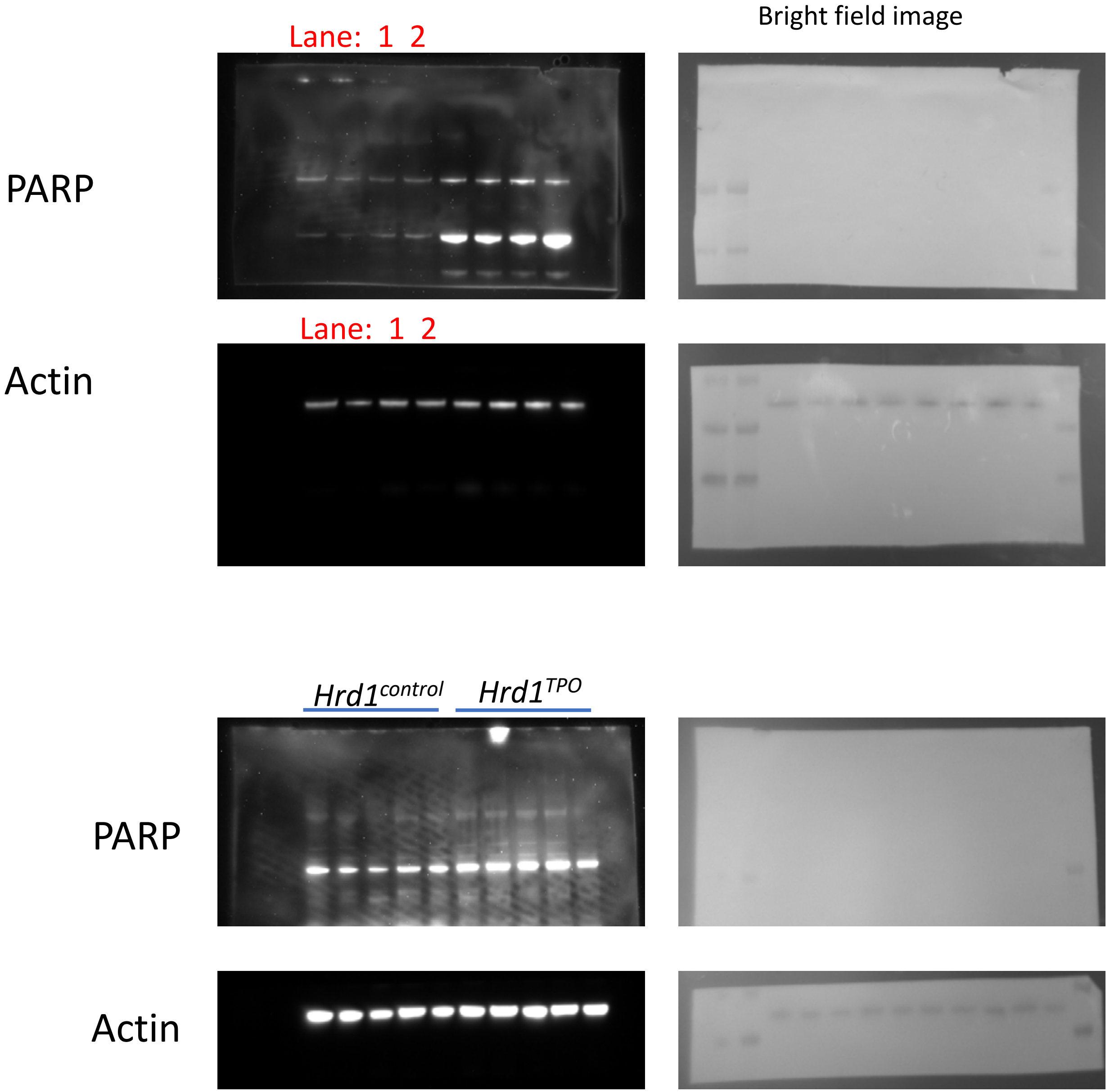

Fig. 1H

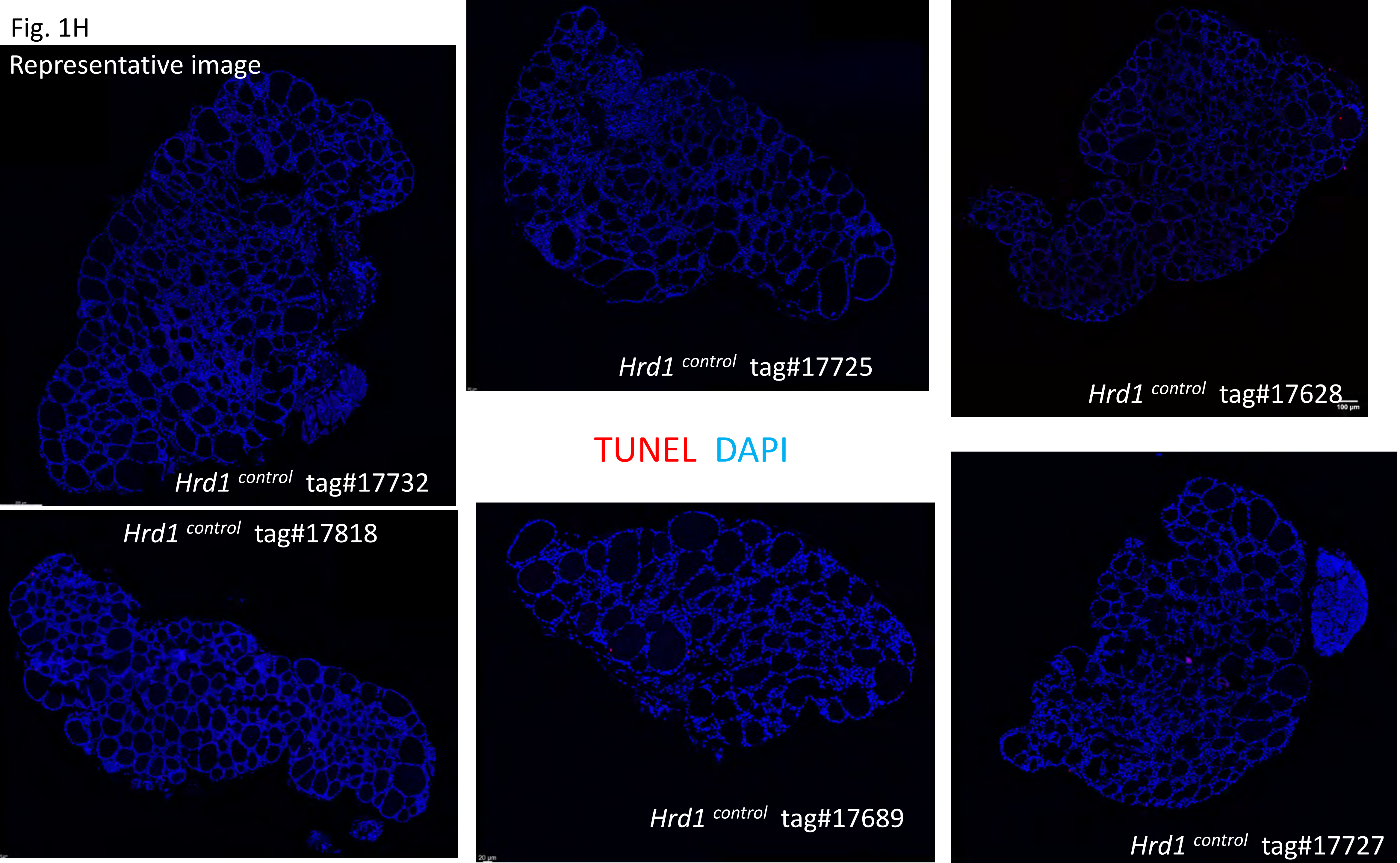
Representative image
Hrd1 control tag#17732
Hrd1 control tag#17725
Hrd1 control tag#17628
100 µm
TUNEL DAPI
Hrd1 control tag#17818
Hrd1 control tag#17689
20 µm
Hrd1 control tag#17727

Fig. 1H TUNEL

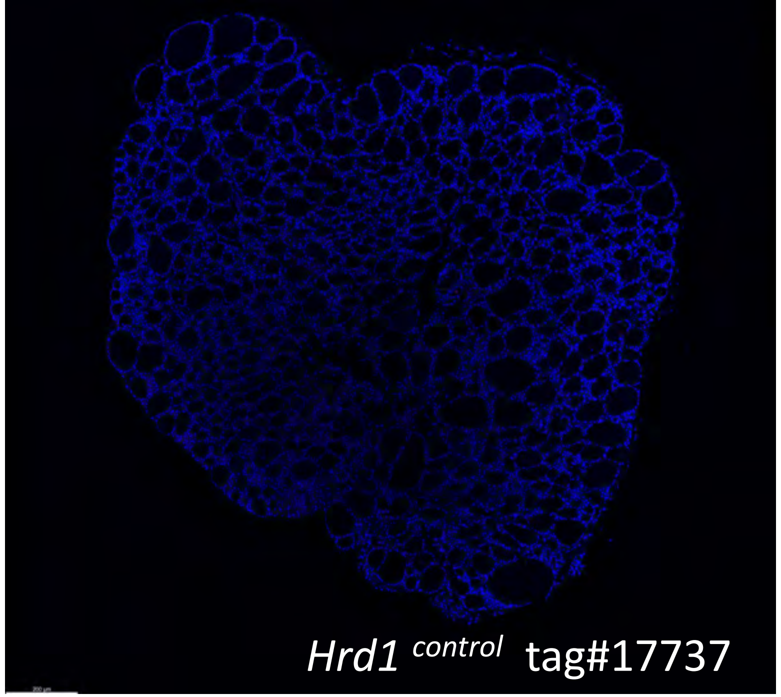

TUNEL DAPI

Quantification:

Mouse tag#	Genotype	Gender	Age months	TUNEL+follicles	Total follicles	% TUNEL+ follicles
17732	$Hrd1^{control}$	M	4.50	0	233	0
17725	$Hrd1^{control}$	M	4.63	2	152	1.315789474
17818	$Hrd1^{control}$	M	4.70	1	230	0.434782609
17628	$Hrd1^{control}$	F	4.63	2	434	0.460829493
17689	$Hrd1^{control}$	F	4.53	1	89	1.123595506
17727	$Hrd1^{control}$	F	4.63	0	155	0
17737	$Hrd1^{control}$	F	4.50	1	435	0.229885057
17681	$Hrd1^{TPO}$	M	4.67	0	32	0
17714	$Hrd1^{TPO}$	M	4.50	1	140	0.714285714
17716	$Hrd1^{TPO}$	F	4.50	4	391	1.023017903
17693	$Hrd1^{TPO}$	F	4.50	1	156	0.641025641
17735	$Hrd1^{TPO}$	F	4.50	1	158	0.632911392

Fig. 1H TUNEL

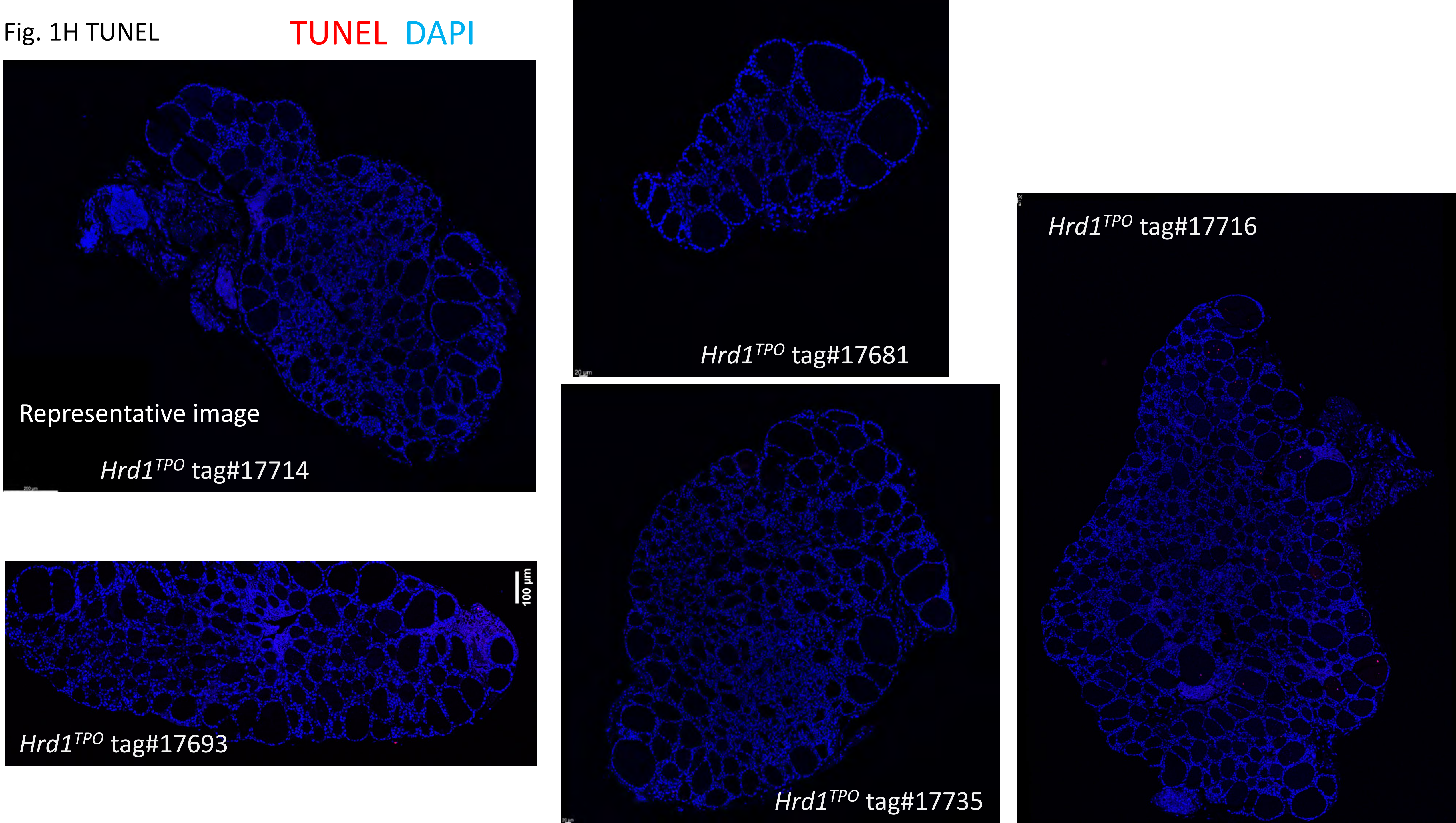
TUNEL DAPI
Representative image
Hrd1^{TPO} tag#17714
Hrd1^{TPO} tag#17681
Hrd1^{TPO} tag#17716
100 µm
Hrd1^{TPO} tag#17693
Hrd1^{TPO} tag#17735

Fig. 2A

Lanes in the figures are labeled here.

Lanes 1-2: *TG*$^{+/+}$; *ATG7*^{control}
Lanes 3-4: *TG*$^{+/+}$; *ATG7*^{TPO}

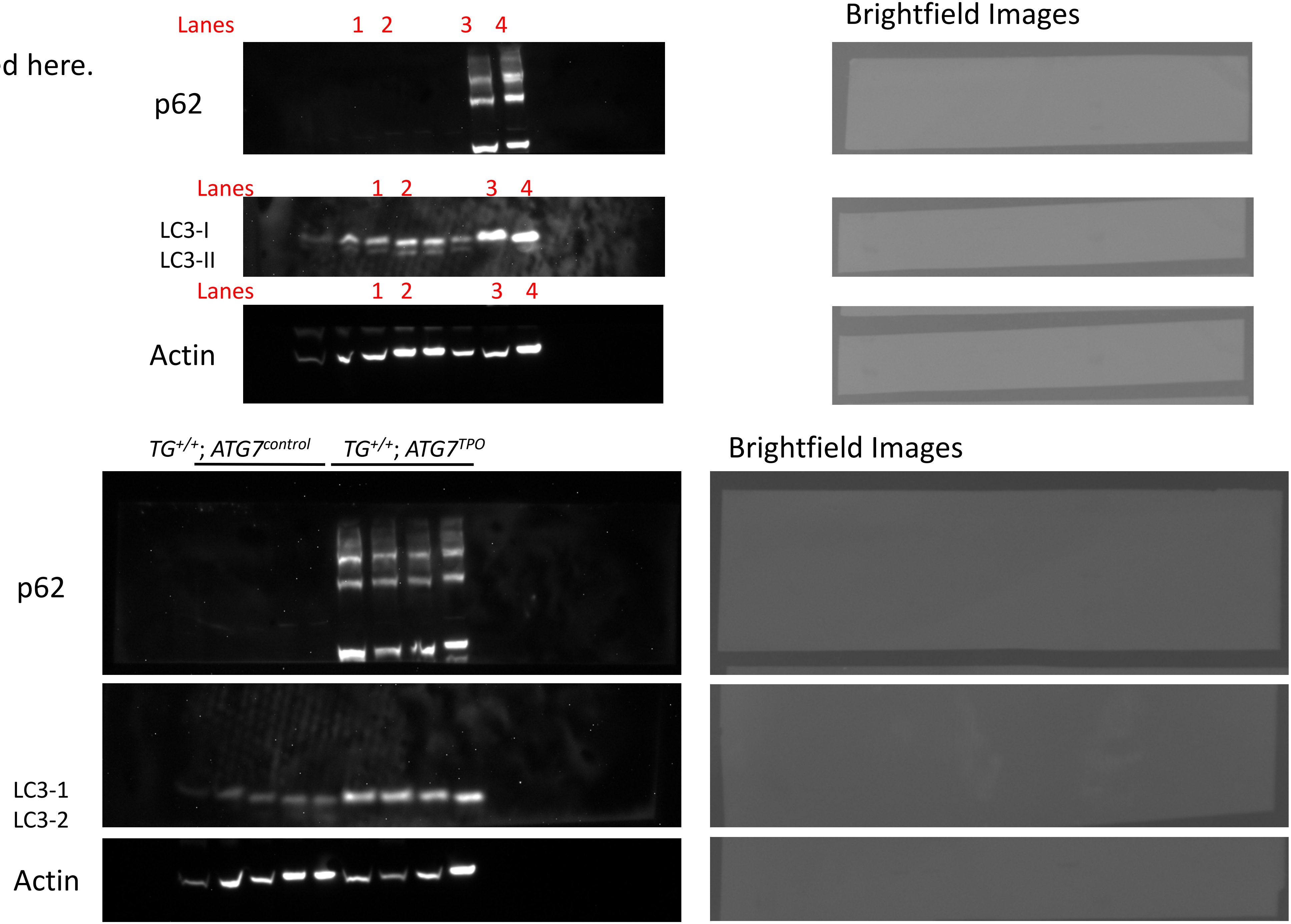

Fig. 2A

Lanes in the figures are labeled here.

Lanes 1-2: *TG*$^{+/+}$; *ATG7*^{control}
Lanes 3-4: *TG*$^{+/+}$; *ATG7*^{TPO}

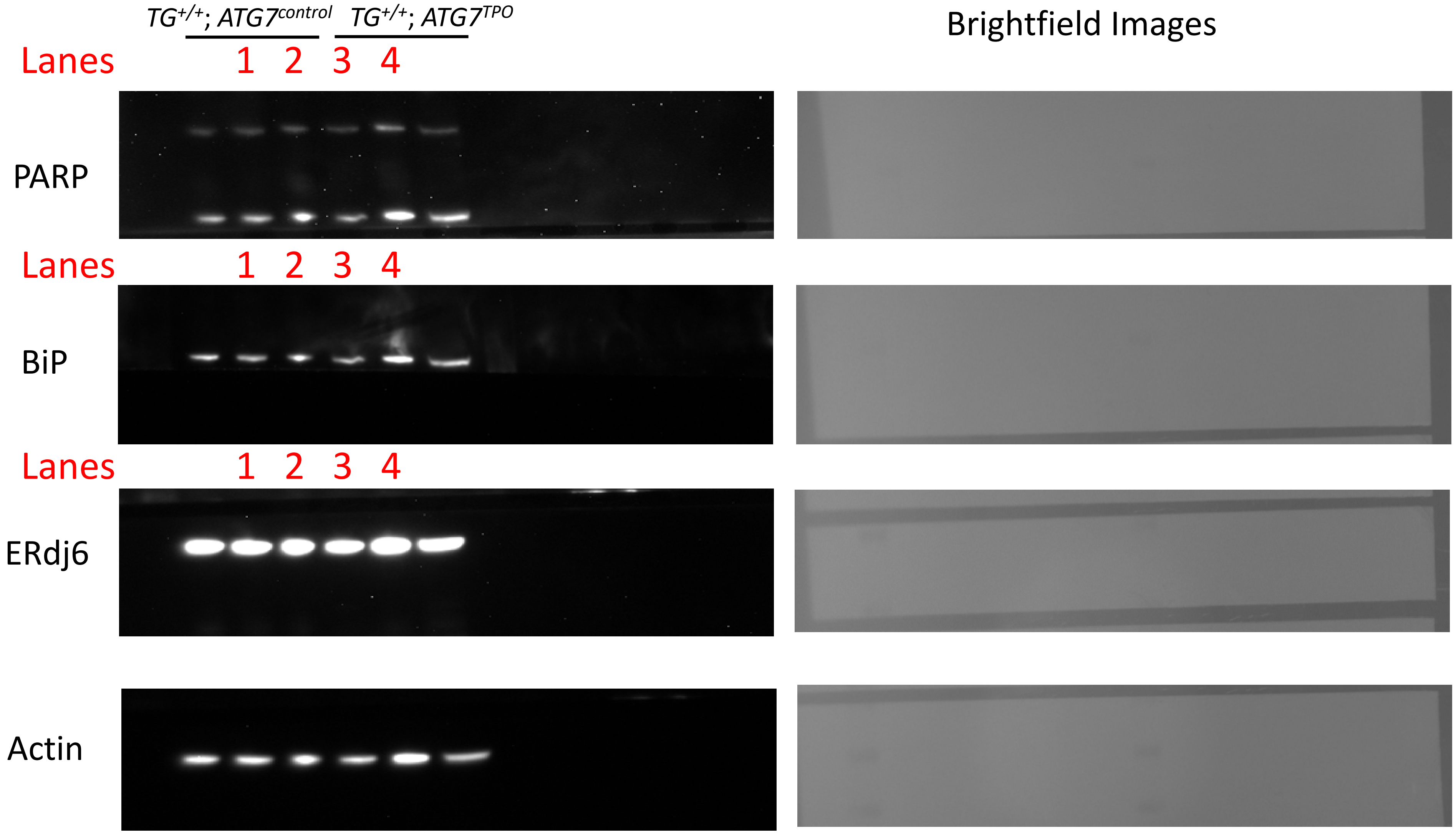

Fig. 2B

Mouse tag#	Genotype	TSH (mU/L)	T_4 (µg/dL)	T_3 (ng/dL)
16628	$ATG7^{control}$	36	3.59	68.8
16630	$ATG7^{control}$	98	3.65	94.4
16633	$ATG7^{control}$	28	3.43	63.6
16848	$ATG7^{control}$	90	3.38	84.7
16850	$ATG7^{control}$	70	2.88	68.3
16619	$ATG7^{control}$	<10	2.67	52.9
16620	$ATG7^{control}$	29	4.71	84.2
16638	$ATG7^{control}$	31	3.83	72.4
16864	$ATG7^{control}$	41	4.63	74.4
16865	$ATG7^{control}$	105	3.08	61.6
17563	$ATG7^{TPO}$	98	2.7	55.4
16627	$ATG7^{TPO}$	313	3.81	82.6
16636	$ATG7^{TPO}$	48	4.32	81.6
16847	$ATG7^{TPO}$	151	4.62	68.8
16849	$ATG7^{TPO}$	32	2.87	61.1
16637	$ATG7^{TPO}$	22	3.58	68.8
16862	$ATG7^{TPO}$	<10	4.79	60.0

Fig. 2C upper panel

Representative image

ATG7^{control} Tag# 16628

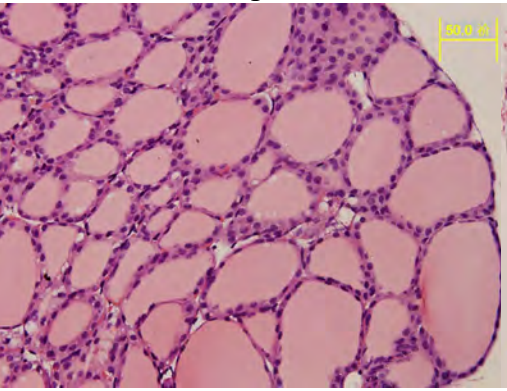

ATG7^{control} Tag# 16630

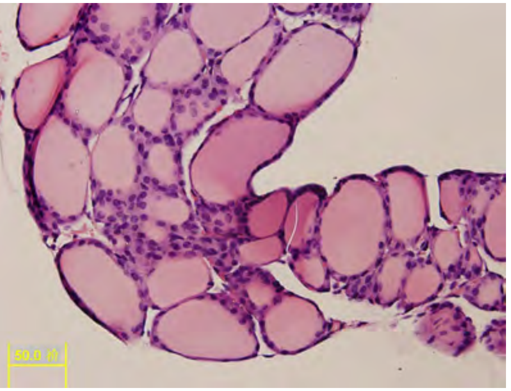

ATG7^{control} Tag# 16633

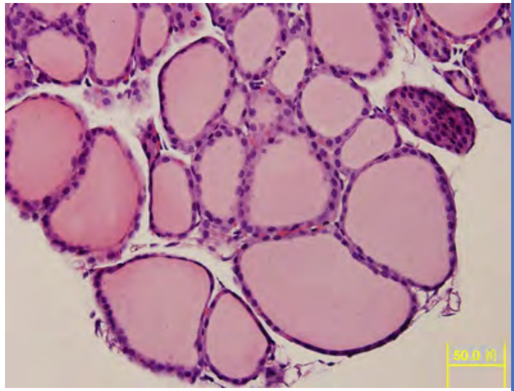

ATG7^{control} Tag# 16638

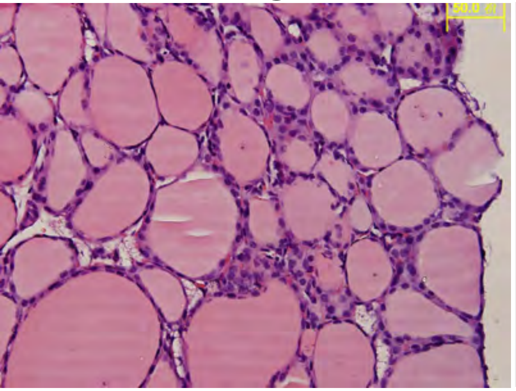

ATG7^{control} Tag# 16848

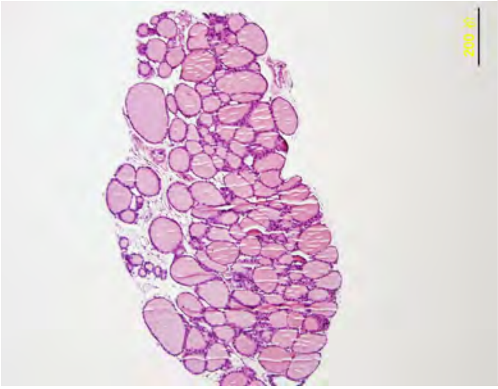

ATG7^{control} Tag# 16850

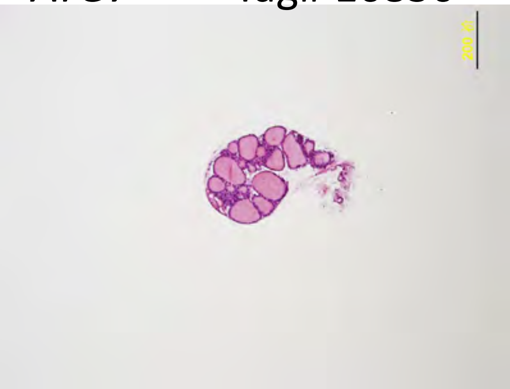

ATG7^{control} Tag# 16864

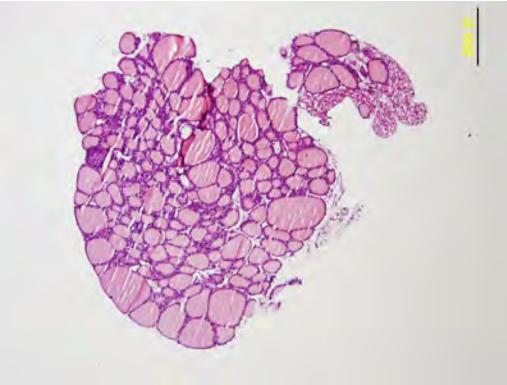

ATG7^{control} Tag# 16865

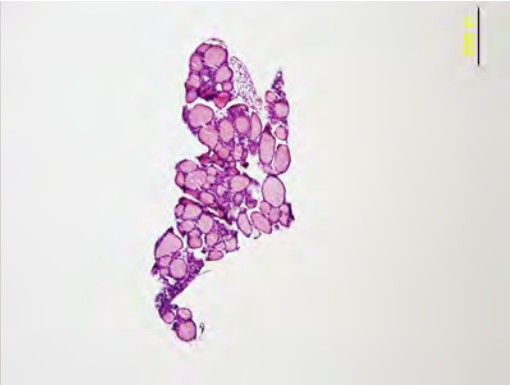

Representative image

ATG7^{TPO} Tag#16636

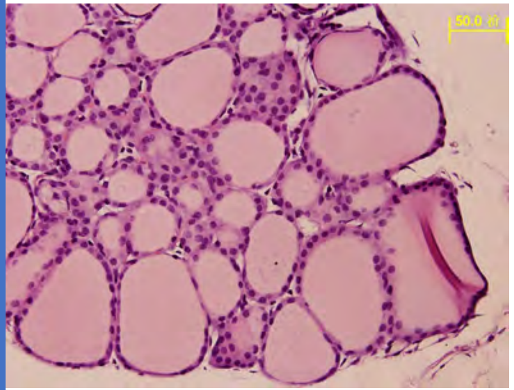

ATG7^{TPO} Tag#16637

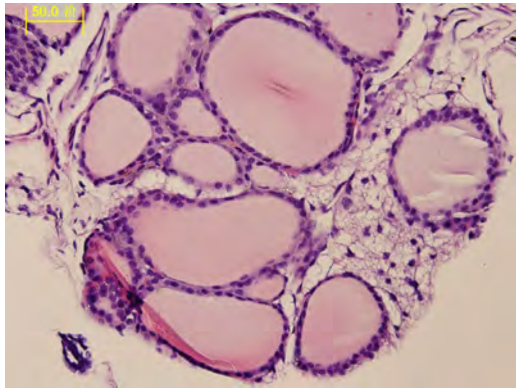

ATG7^{TPO} Tag#16627

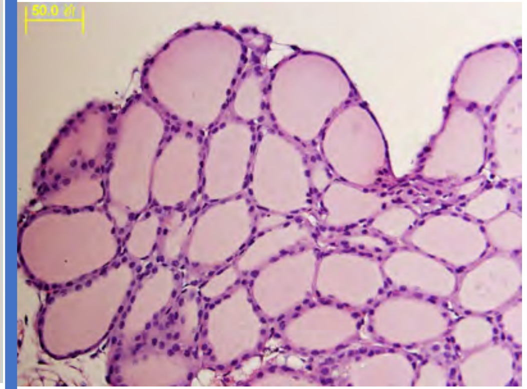

ATG7^{TPO} Tag#16847

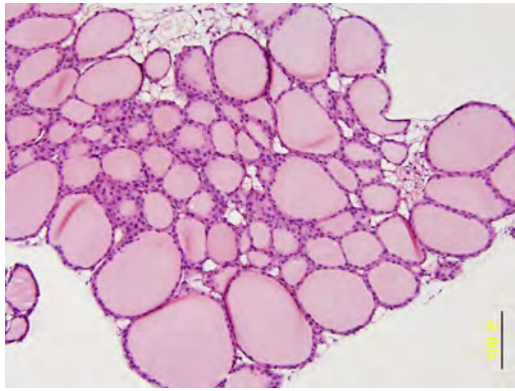

ATG7^{TPO} Tag#16849

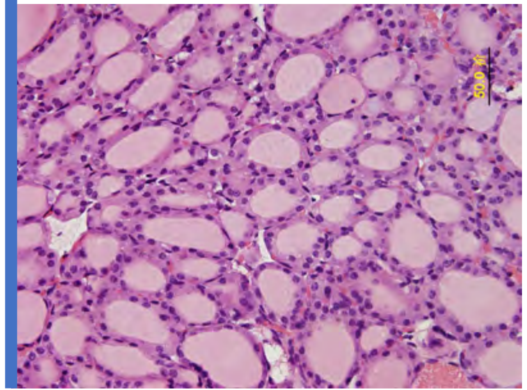

ATG7^{TPO} Tag#16862

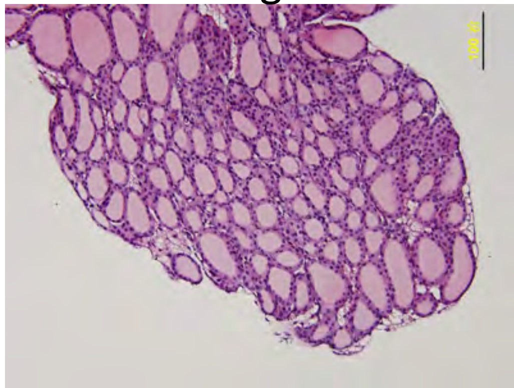

Fig. 2C lower panel

Representative image

ATG7^{control} Tag# 16848

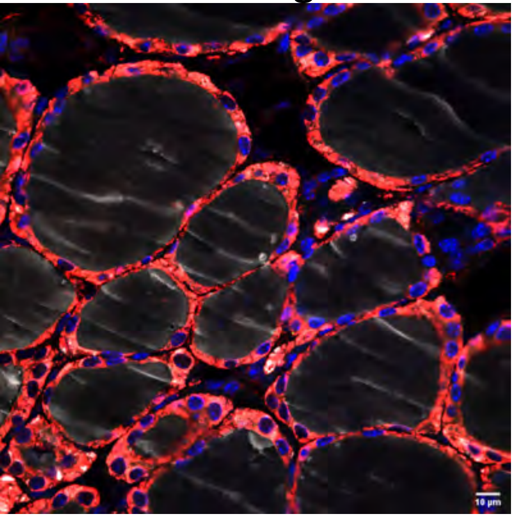

ATG7^{control} Tag# 16850

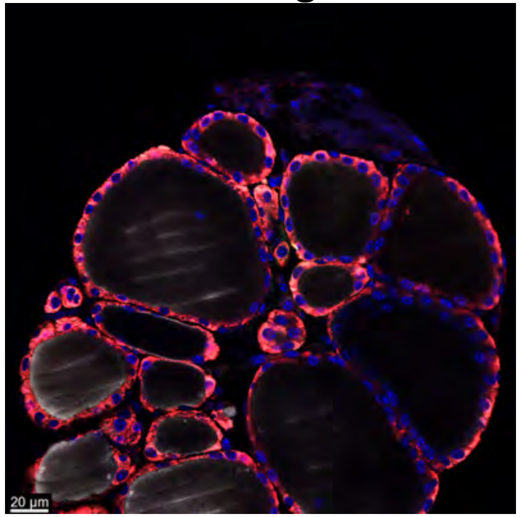

ATG7^{control} Tag# 16864

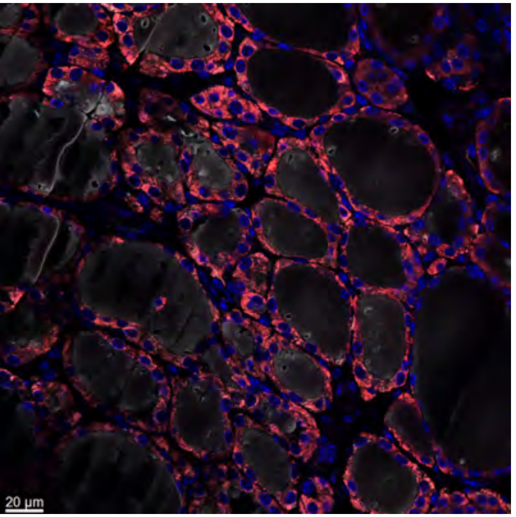

ATG7^{control} Tag# 16865

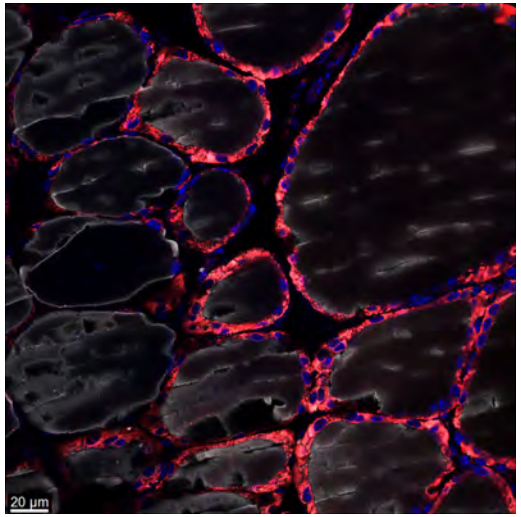

Representative image

ATG7^{TPO} Tag#16849

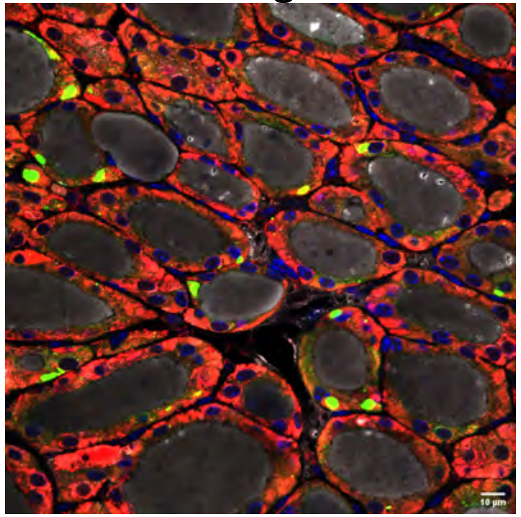

ATG7^{TPO} Tag#16862

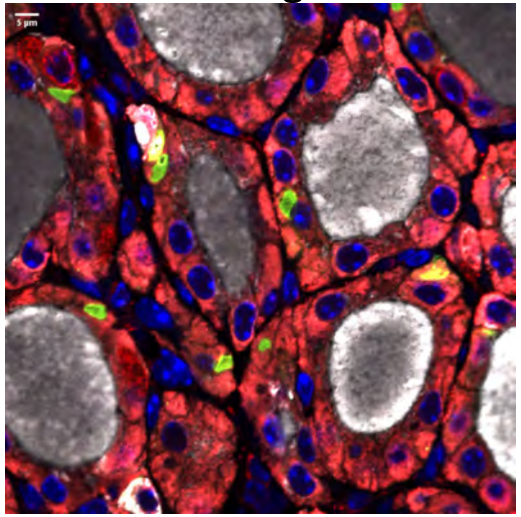

ATG7^{TPO} Tag#16770

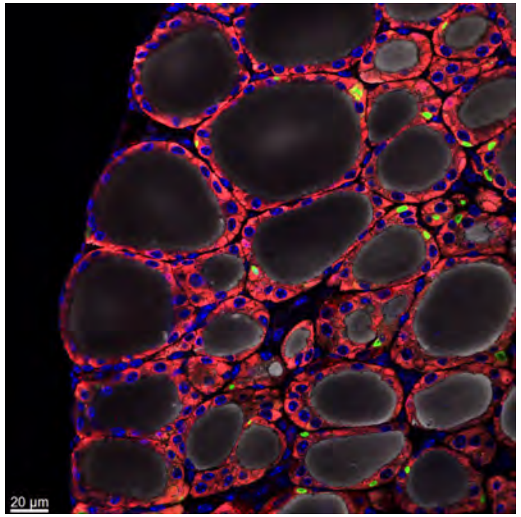

Fig. 3A

Left three panels

Mouse tag#	Genotype	TSH (mU/L)	T_4 (µg/dL)	T_3 (ng/dL)	Treatment
17232	$TG^{+/+}$	<10	3.55	97.1	
17233	$TG^{+/+}$	26	2.47	62.8	
17174	$TG^{+/+}$	32	2.84	70.0	
17234	$TG^{+/+}$	<10	3.84	75.3	
17175	$TG^{+/+}$	<10	2.95	79.7	
17176	$TG^{+/+}$	<10	3.01	90.8	
17177	$TG^{+/+}$	<10	0.87	63.7	
17472	$TG^{+/cog}$	602	2.24	83.5	
17523	$TG^{+/cog}$	318	1.63	70.5	
17524	$TG^{+/cog}$	242	1.40	107.7	
17525	$TG^{+/cog}$	125	2.24	63.7	
17476	$TG^{+/cog}$	2022	2.28	63.7	
17475	$TG^{+/cog}$	154	2.15	82.1	
17571	$TG^{+/cog}$	243	3.20	81.1	
17558	$TG^{+/cog}$	10	3.12	77.3	
17557	$TG^{+/cog}$	<10	3.71	92.2	
17556	$TG^{+/cog}$	14	2.43	102.4	
17529	$TG^{+/cog}$	149	2.44	97.5	
17992	$TG^{+/cog}$	<10	1.24	27.2	T_4
17993	$TG^{+/cog}$	52	1.36	35.9	T_4
17994	$TG^{+/cog}$	53	1.94	22.9	T_4
17995	$TG^{+/cog}$	<10	6.08	91.8	T_4
17996	$TG^{+/cog}$	<10	6.12	50.3	T_4
17997	$TG^{+/cog}$	<10	5.20	40.2	T_4
17998	$TG^{+/cog}$	<10	5.58	75.0	T_4

Fig. 3A Panel on the right

$TG^{+/+}$

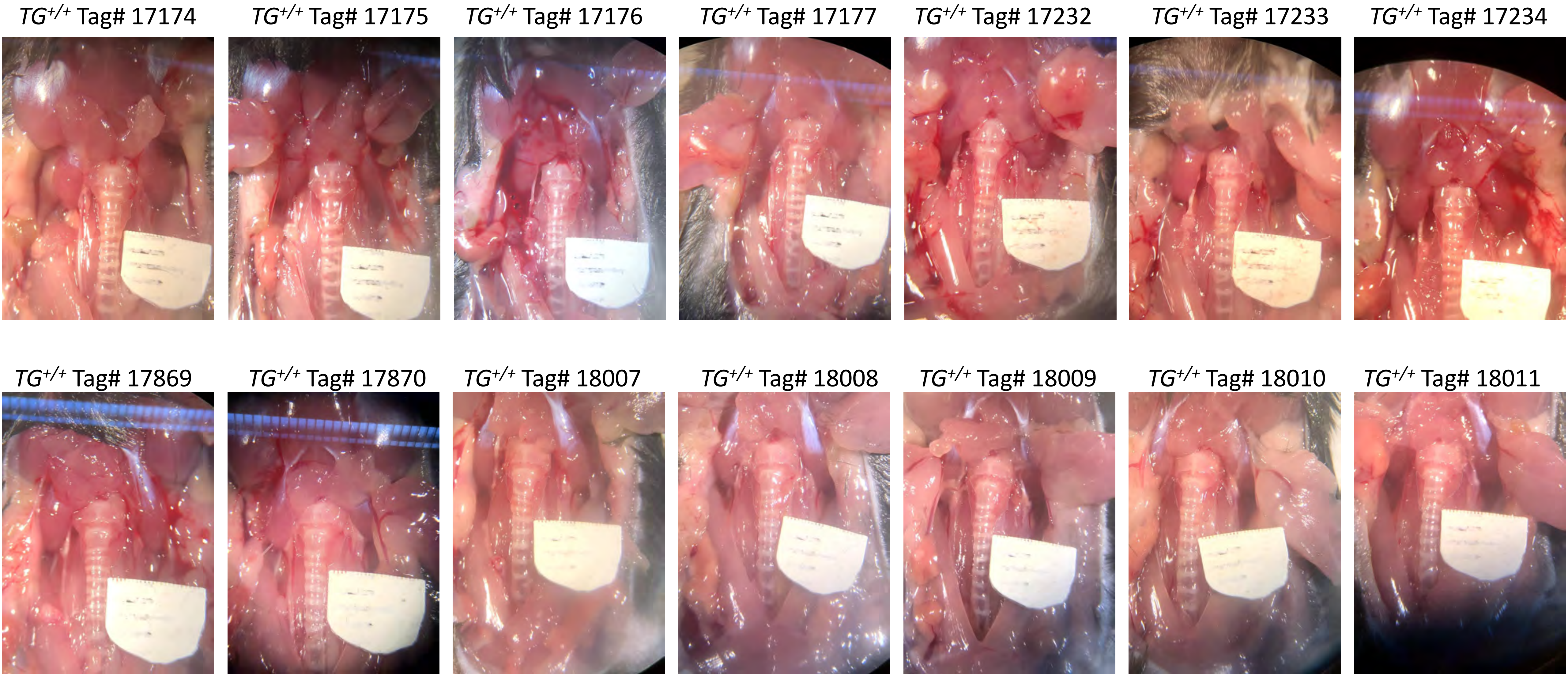

Fig. 3A Panel on the right

$TG^{+/cog}$

$TG^{+/cog}$ Tag# 17472

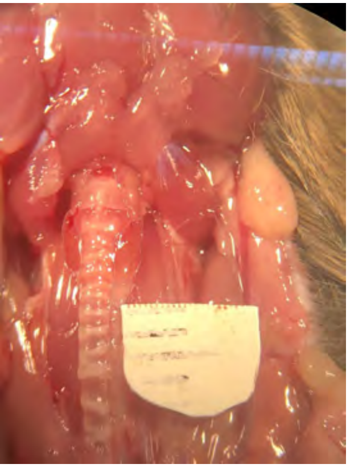

$TG^{+/cog}$ Tag# 17475

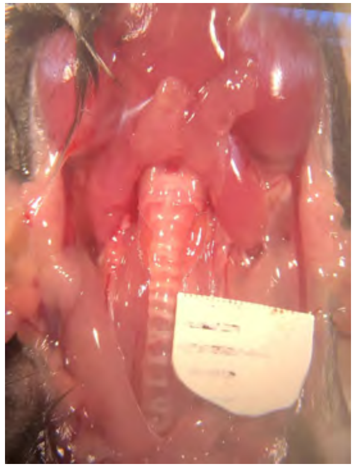

$TG^{+/cog}$ Tag# 17476

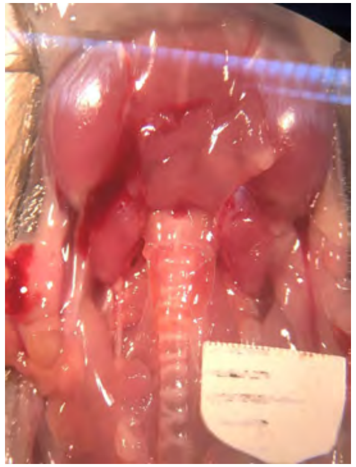

$TG^{+/cog}$ Tag# 17556

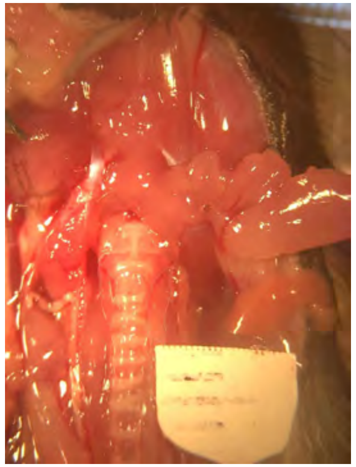

$TG^{+/cog}$ Tag# 17557

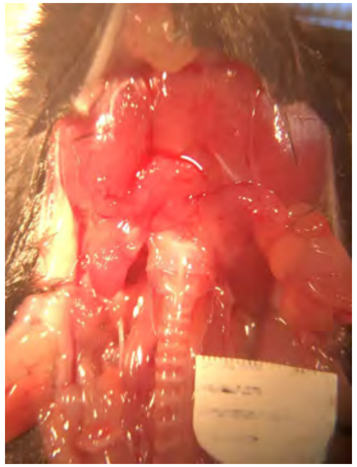

$TG^{+/cog}$ Tag# 17558

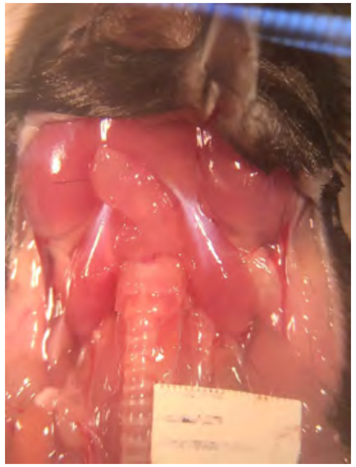

$TG^{+/cog}$ Tag# 17827

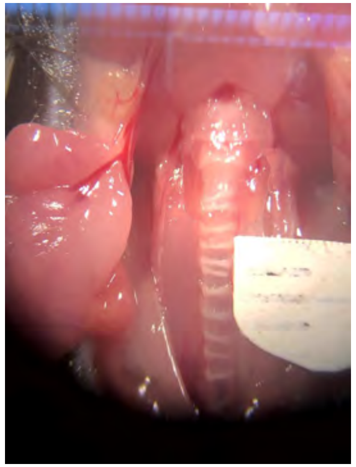

$TG^{+/cog}$ Tag# 17828

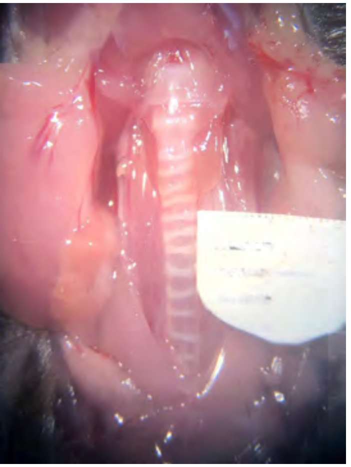

$TG^{+/cog}$ Tag# 17829

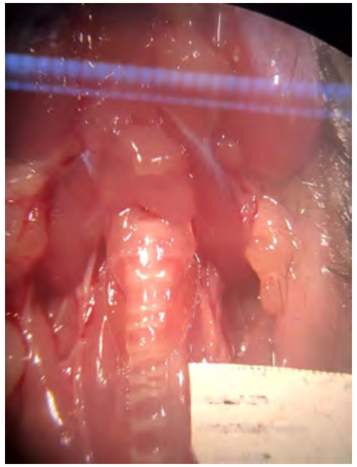

$TG^{+/cog}$ Tag# 17833

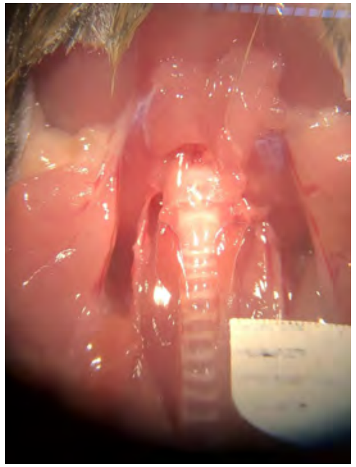

Fig. 3A Panel on the right

$TG^{+/cog} + T_4$

$TG^{+/cog}$ Tag# 17992

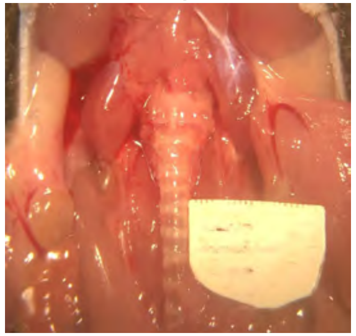

$TG^{+/cog}$ Tag# 17993

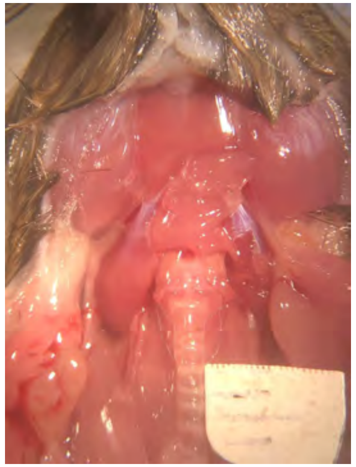

$TG^{+/cog}$ Tag# 17994

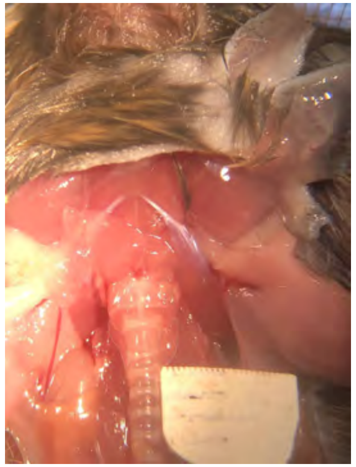

$TG^{+/cog}$ Tag# 17995

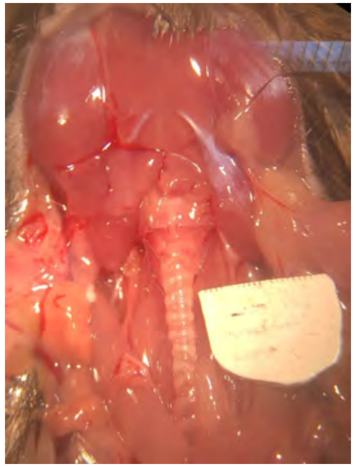

$TG^{+/cog}$ Tag# 17996

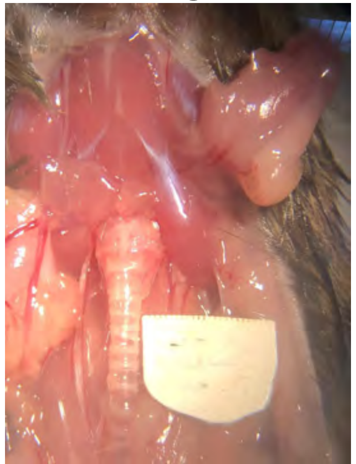

$TG^{+/cog}$ Tag# 17997

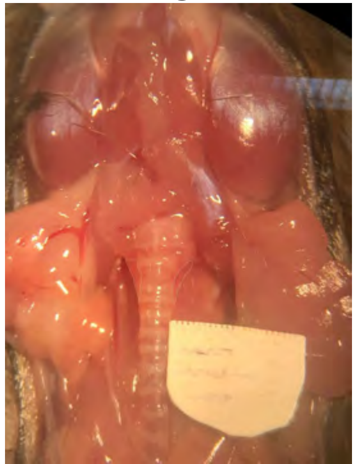

$TG^{+/cog}$ Tag# 17998

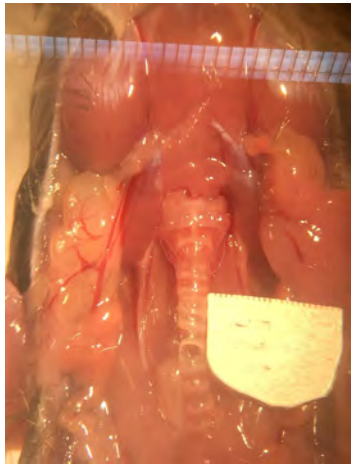

Quantification:

#tag	Genotype	Body weight (g)	area_left lobe (mm^2)	area_right lobe (mm2)	total area (mm2)	thyroid area / body weight	Treatment
17175	$TG^{+/+}$	25	0.623	1.16	1.783	0.07132	
17176	$TG^{+/+}$	21.5	1.459	1.701	3.16	0.146976744	
17177	$TG^{+/+}$	21.1	0.651	1.531	2.182	0.103412322	
17234	$TG^{+/+}$	23.3	0.507	1.693	2.2	0.094420601	
17869	$TG^{+/+}$	23.6	0.549	2.024	2.573	0.109025424	
17870	$TG^{+/+}$	22.7	0.896	1.751	2.647	0.11660793	
17174	$TG^{+/+}$	27.6	0.691	1.562	2.253	0.081630435	
17232	$TG^{+/+}$	27.4	1.673	3.297	4.97	0.181386861	
17233	$TG^{+/+}$	31.6	1.118	1.943	3.061	0.096867089	
18007	$TG^{+/+}$	29.1	0.865	1.948	2.813	0.096666667	
18008	$TG^{+/+}$	29.12	1.274	2.124	3.398	0.11668956	
18009	$TG^{+/+}$	30.32	1.097	1.76	2.857	0.094228232	
18010	$TG^{+/+}$	27.99	0.689	2.173	2.862	0.102250804	
18011	$TG^{+/+}$	30.7	1.921	2.809	4.73	0.154071661	
17475	$TG^{+/cog}$	20.6	1.37	2.942	4.312	0.209320388	
17476	$TG^{+/cog}$	21.4	1.954	2.65	4.604	0.215140187	
17556	$TG^{+/cog}$	21.5	2.341	2.196	4.537	0.211023256	
17557	$TG^{+/cog}$	18	0.484	1.75	2.234	0.124111111	
17558	$TG^{+/cog}$	19.9	0.702	1.987	2.689	0.135125628	
17828	$TG^{+/cog}$	22.7	0.739	1.785	2.524	0.111189427	
17829	$TG^{+/cog}$	25.9	1.456	2.34	3.796	0.146563707	
17472	$TG^{+/cog}$	28.8	1.215	1.808	3.023	0.104965278	
17827	$TG^{+/cog}$	27.3	0.928	2.441	3.369	0.123406593	
17833	$TG^{+/cog}$	28.8	1.054	1.894	2.948	0.102361111	
17995	$TG^{+/cog}$	22.6	1.055	0.613	1.668	0.07380531	T_4
17996	$TG^{+/cog}$	22.2	0.295	0.804	1.099	0.049504505	T_4
17997	$TG^{+/cog}$	21	0.57	0.571	1.141	0.054333333	T_4
17998	$TG^{+/cog}$	19.9	0.432	0.595	1.027	0.05160804	T_4
17992	$TG^{+/cog}$	28.3	1.013	0.66	1.673	0.059116608	T_4
17993	$TG^{+/cog}$	28.9	0.878	1.218	2.096	0.072525952	T_4
17994	$TG^{+/cog}$	24.7	0.831	1.381	2.212	0.089554656	T_4

Fig. 3B Upper panel

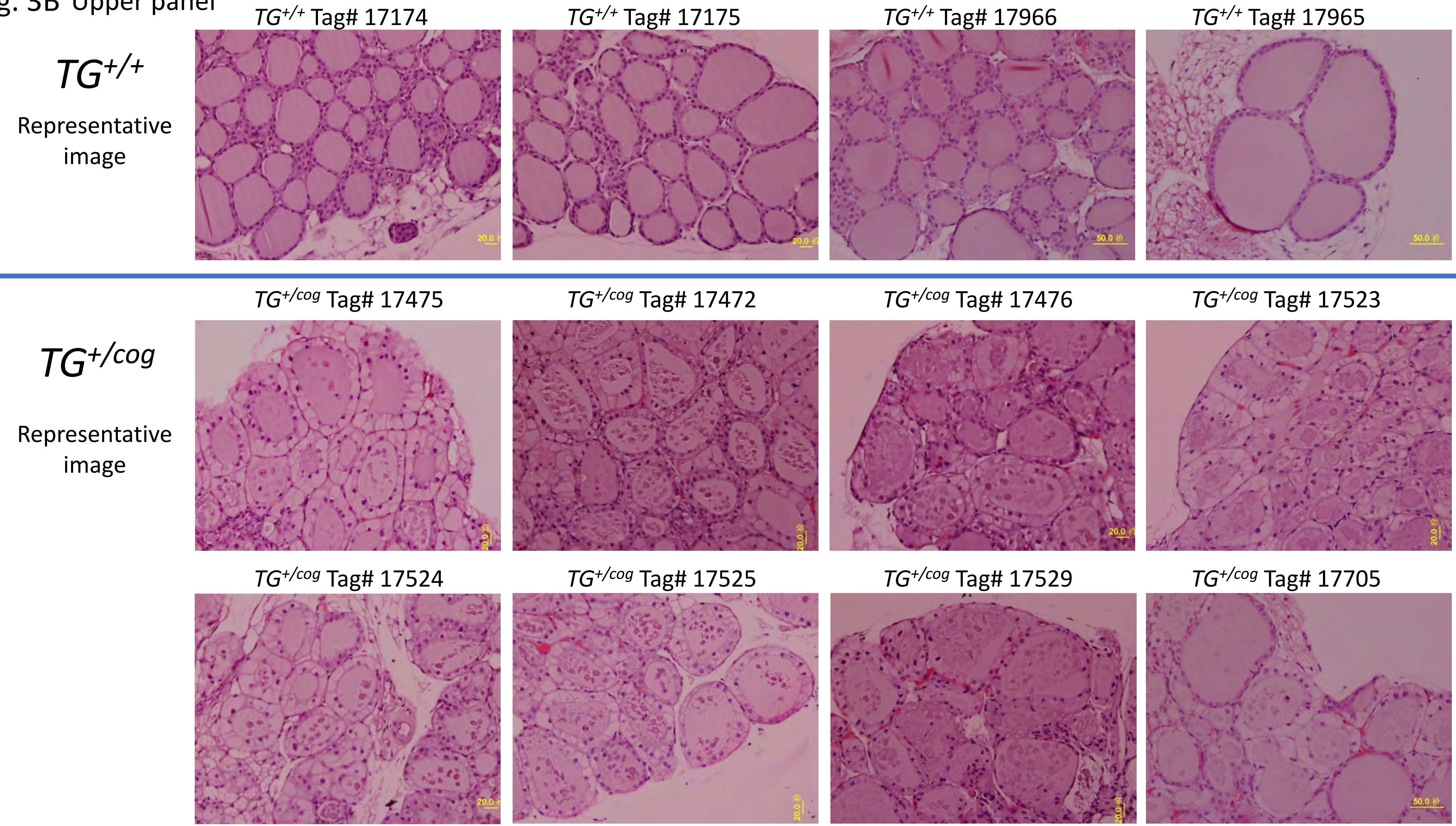

Fig. 3B Upper panel

TG$^{+/cog}$ + T_4

Representative image

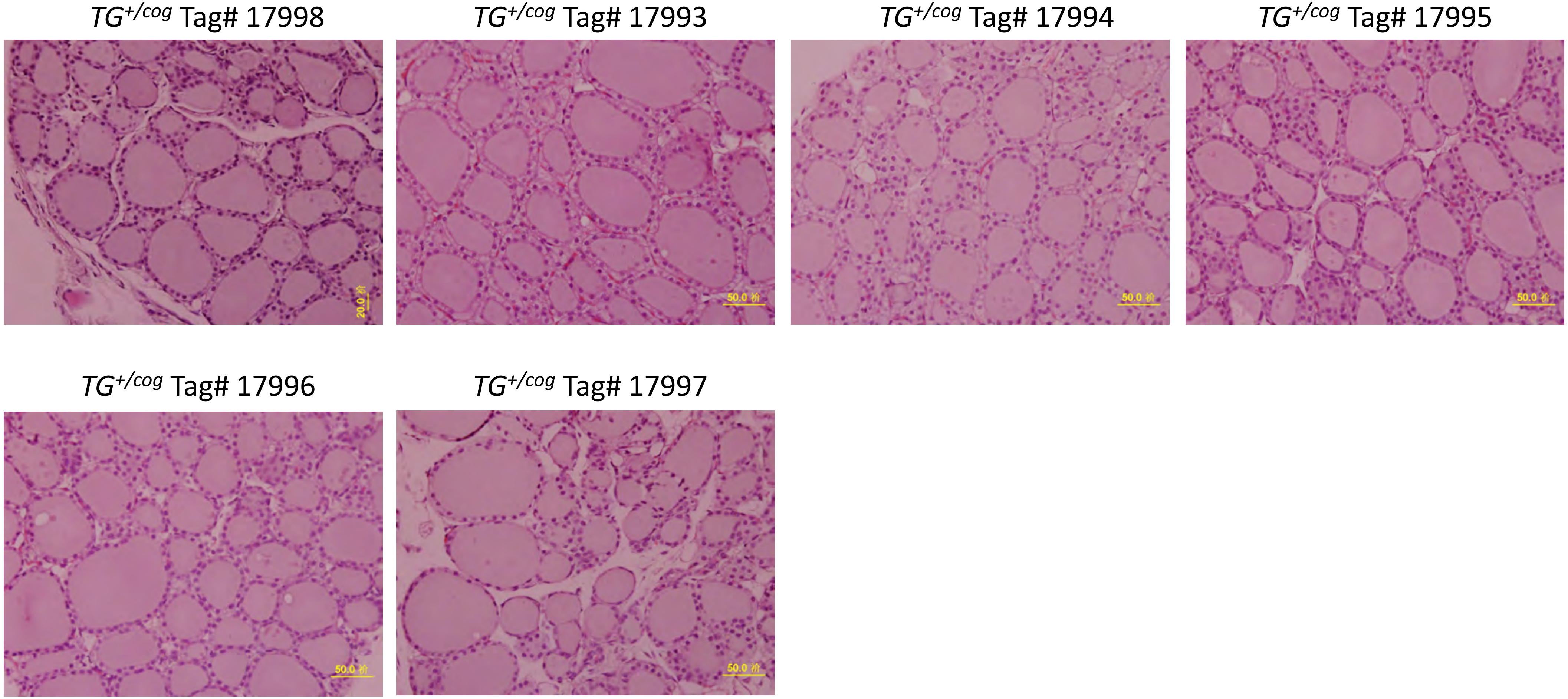

Fig 3B Lower panel

These images were used in the figure as representative.

Tag# 17177 *TG*$^{+/+}$

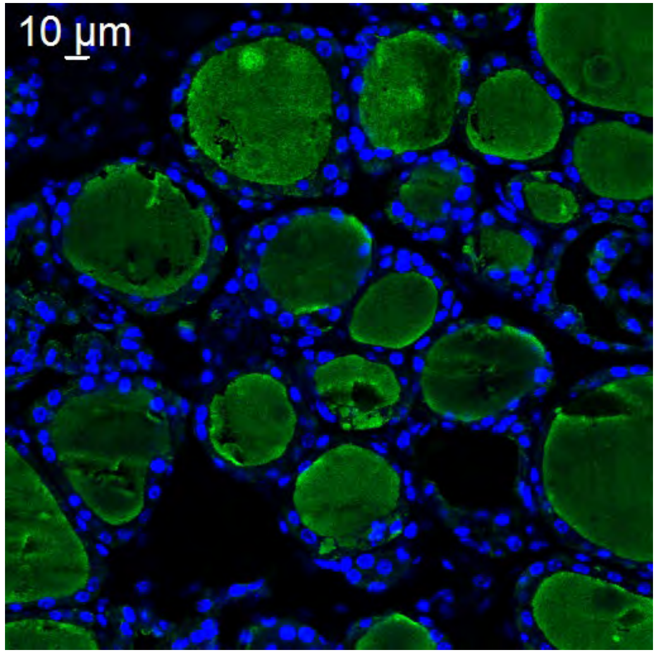

Tag# 17475 *TG*$^{+/cog}$

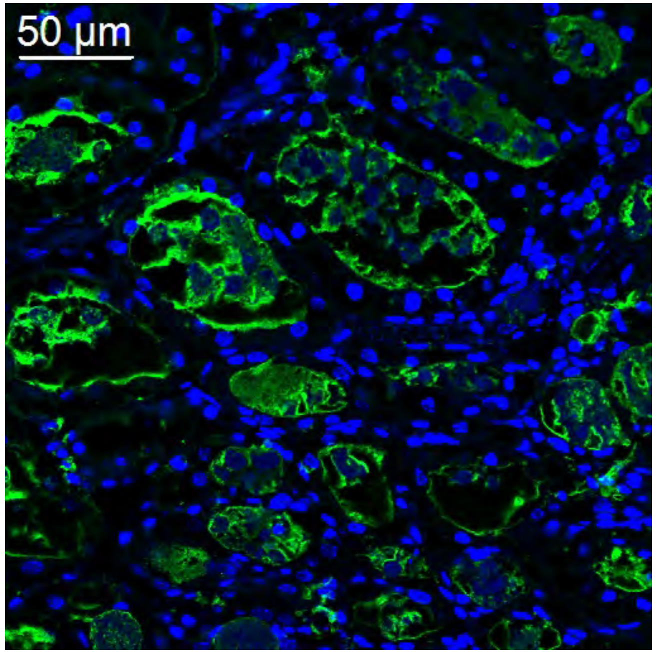

Tag# 17995 *TG*$^{+/cog}$ + T_4

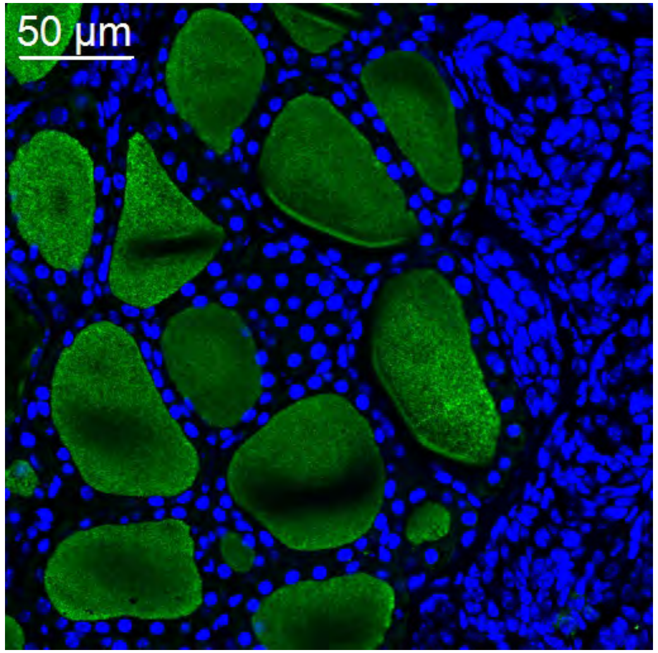

Fig 3B Lower panel

Additional images.

Tag# 17472 $TG^{+/cog}$

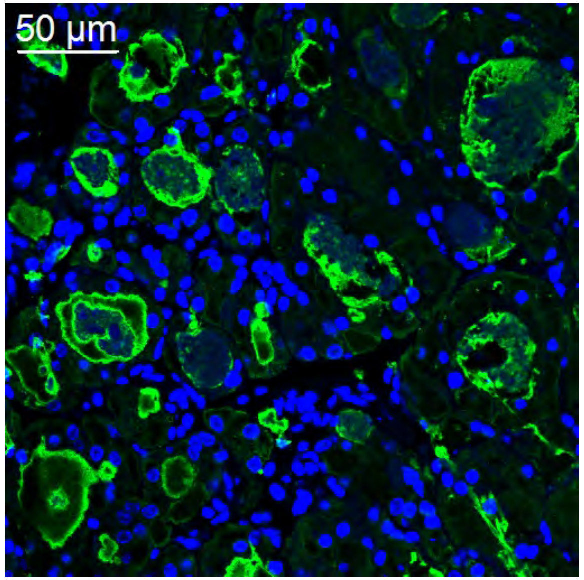

Tag# 17476 $TG^{+/cog}$

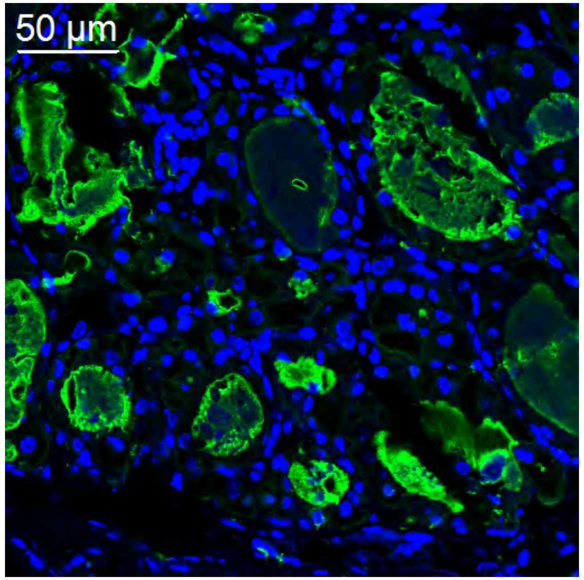

Tag# 17529 $TG^{+/cog}$

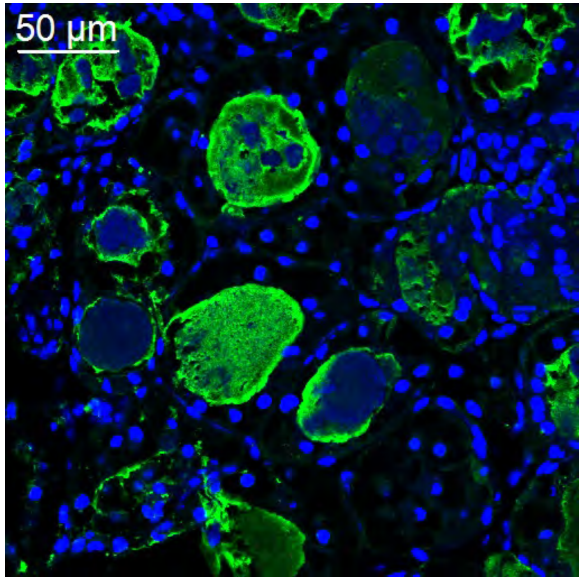

Tag# 17869 $TG^{+/+}$

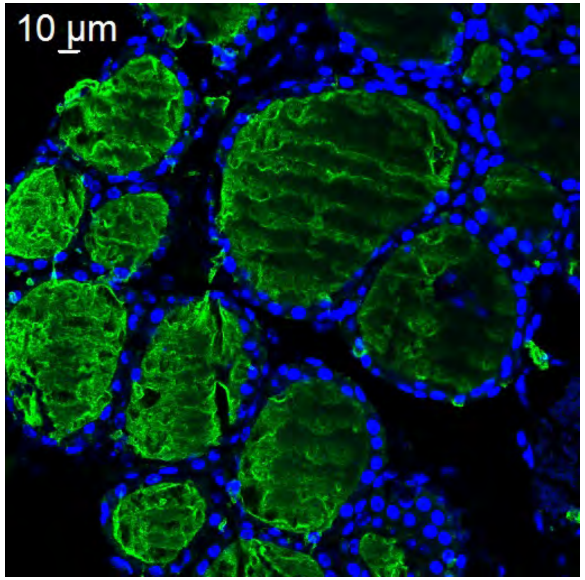

Tag# 17996 $TG^{+/cog}$ + T_4

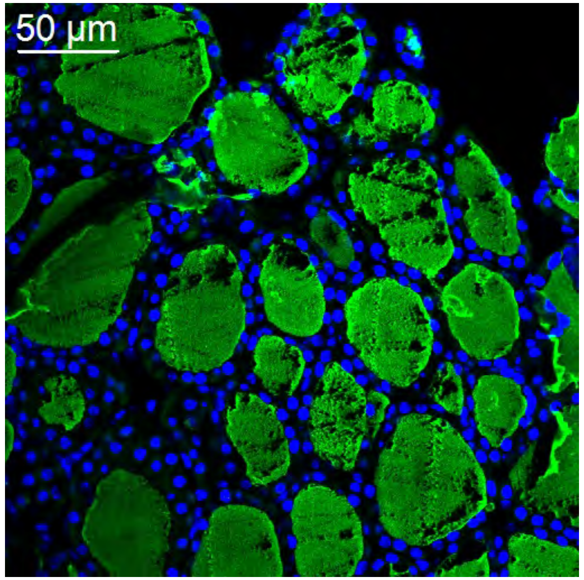

Tag# 17997 $TG^{+/cog}$ + T_4

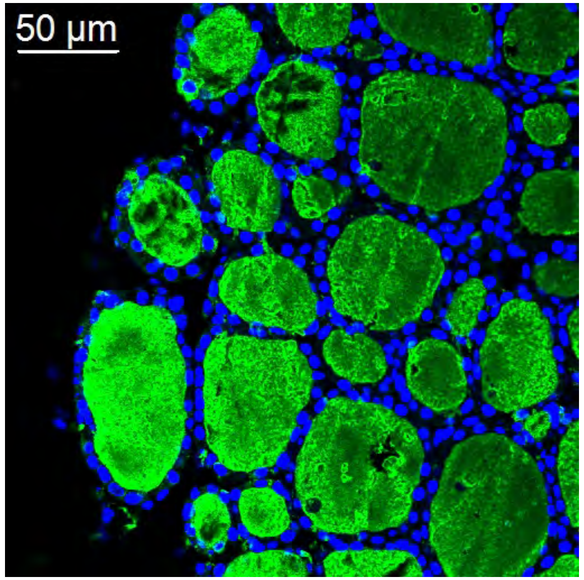

Tag# 17998 $TG^{+/cog}$ + T_4

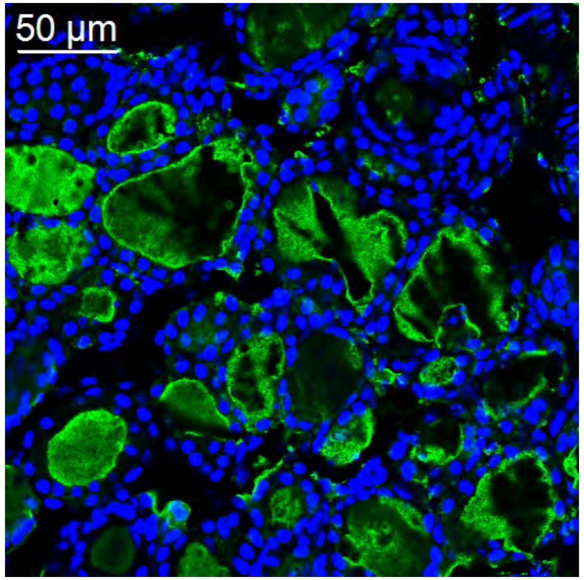

Fig. 3C qPCR

Ct (3 replicates):

tag#18007	rep1	rep2	rep3	Mean		tag#18008	rep1	rep2	rep3	mean
Tg	13.16703129	12.95415306	12.7510767	12.95742		Tg	13.1933918	13.3217	13.37412	13.2964
chop	24.77302933	24.94067764	24.935463	24.88306		chop	25.2556324	25.02275	25.22066	25.16635
18s	13.00080585	12.82466125	13.0092888	12.94492		18s	12.8445034	13.04183	13.09953	12.99529
tag#18009	rep1	rep2	rep3	mean		tag#18010	rep1	rep2	rep3	mean
Tg	12.77206707	12.57423496	12.3215361	12.55595		Tg	15.02876759	15.11505	15.21083	15.11821
chop	25.00681686	24.93216896	24.6948605	24.87795		chop	27.15423012	27.25795	27.12808	27.18009
18s	12.81876278	12.79088402	13.2535925	12.95441		18s	14.19305992	14.46142	14.33741	14.33063
tag#18011	rep1	rep2	rep3	mean		tag#18029	rep1	rep2	rep3	mean
Tg	13.52591991	13.42884541	13.7999458	13.5849		Tg	11.93099308	11.83379	11.74644	11.83708
chop	25.57158089	25.48855591	25.4114761	25.49054		chop	21.65073013	21.68721	21.72664	21.68819
18s	13.0182972	12.96792507	12.9281883	12.97147		18s	11.06890774	12.78808	11.3948	11.7506
tag#18034	rep1	rep2	rep3	mean		tag#18035	rep1	rep2	rep3	mean
Tg	12.03057861	11.91415882	11.7316322	11.89212		Tg	11.73976326	12.9328	12.20193	12.2915
chop	22.14948082	22.07621193	22.0254173	22.0837		chop	20.86255074	20.41062	20.98329	20.75215
18s	10.74282932	11.02647686	11.1580811	10.9758		18s	11.36037922	11.3775	11.21716	11.31835
tag#18036	rep1	rep2	rep3	mean		tag#18036	rep1	rep2	rep3	mean
Tg	11.83016682	12.51915741	11.9652929	12.10487		Tg	11.8895874	12.15633	12.02916	12.02503
chop	20.65986443	20.72397614	20.6047802	20.66287		chop	20.85978889	20.88022	20.83102	20.85701
18s	11.14535236	11.18970299	11.2654743	11.20018		18s	11.6445303	11.49561	12.86552	12.00189

Mean of Ct from 3 replicates:

$TG^{+/+}$ tag#	18007	18008	18009	18010	18011		$TG^{+/cog}$ tag#	18029	18034	18035	18036	18037
Tg	12.95742035	13.29640357	12.555946	15.11821	13.584904			11.83708	11.89212	12.2915	12.02503	12.10487
chop	24.88305664	25.16634623	24.8779488	27.18009	25.490538			21.68819	22.0837	20.75215	20.85701	20.66287
18s	12.94491863	12.99528853	12.9544131	14.33063	12.97147			11.7506	10.9758	11.31835	11.20018	12.00189

ΔCt:

$TG^{+/+}$ tag#	18007	18008	18009	18010	18011	mean of control	$TG^{+/cog}$ tag#	18029	18034	18035	18036	18037
Tg	0.012501717	0.301115036	-0.3984671	0.787584	0.6134335	0.263233376		0.08648	0.916327	0.973148	0.82485	0.102985
chop	11.93813801	12.1710577	11.9235357	12.84946	12.519067	12.28025131		9.937597	11.10791	9.433805	9.656835	8.660987

ΔΔCt:

$TG^{+/+}$ tag#	18007	18008	18009	18010	18011		$TG^{+/cog}$ tag#	18029	18034	18035	18036	18037
Tg	-0.250731659	0.03788166	-0.6617004	0.52435	0.3502001			-0.17675	0.653094	0.709915	0.561617	-0.16025
chop	-0.342113304	-0.109193611	-0.3567156	0.569206	0.2388161			-2.34265	-1.17234	-2.84645	-2.62342	-3.61926

$2^{-\Delta\Delta Ct}$

$TG^{+/+}$ tag#	18007	18008	18009	18010	18011		$TG^{+/cog}$ tag#	18029	18034	18035	18036	18037
Tg	1.189810371	0.974084166	1.58194609	0.695272	0.7844753			1.130337	0.635915	0.611356	0.677542	1.117479
chop	1.267612072	1.078625175	1.28050745	0.673987	0.8474404			5.072349	2.253775	7.192265	6.162074	12.28874

Fig. 3D upper panel

Lanes in the figure are numbered here.

Lane 1-4: *TG*$^{+/+}$

Lane 5-8: *TG*$^{+/cog}$

Lane 9-13: *TG*$^{+/cog}$ + T_4

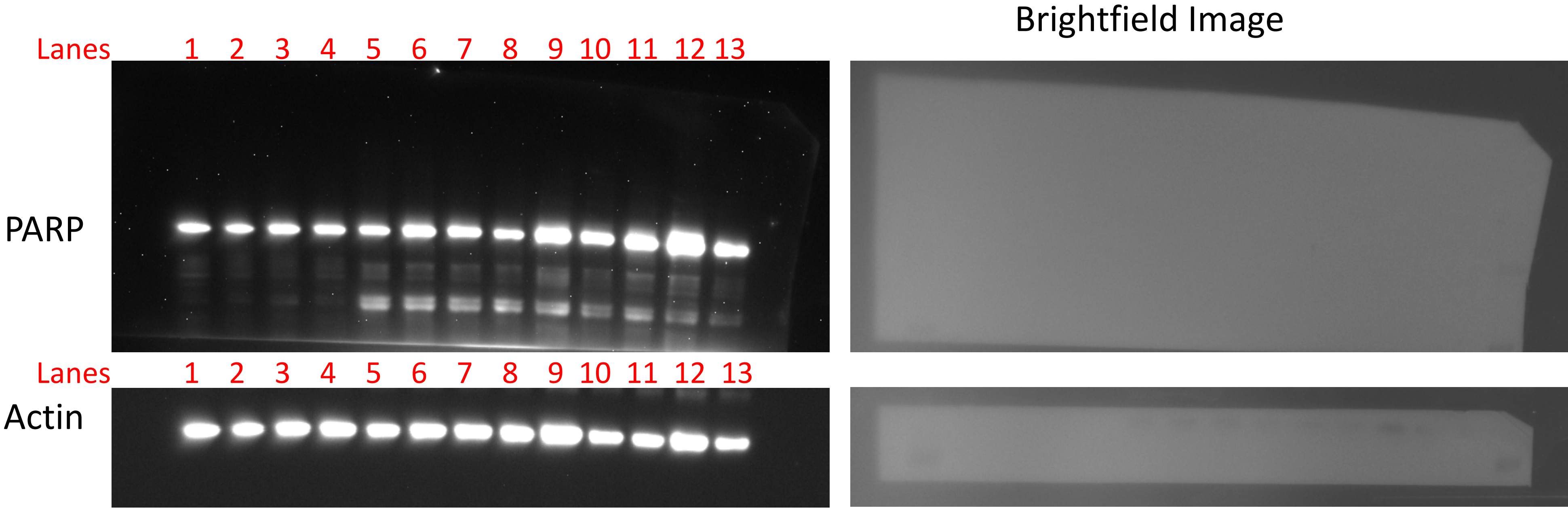

Quantification:

	Lane:	1	2	3	4	5	6	7	8	9	10	11	12	13
	Full-length PARP	351856	297745	400857	384369	374319	512689	491473	409400	697361	553683	636650	905671	520351
	Cleaved PARP	4057	8127	34642	43648	240118	257674	243153	232208	257984	186188	216513	183200	104140
	CL/FL PARP	0.01153	0.027295	0.08642	0.113558	0.64148	0.502593	0.494743	0.567191	0.369943	0.336272	0.340082	0.202281	0.200134
Mean of control	CL/FL PARP	0.059701												
	Lane:	1	2	3	4	5	6	7	8	9	10	11	12	13
Fold change	CL/FL PARP	0.193135	0.4572	1.447551	1.902114	10.74492	8.418546	8.28706	9.500574	6.196631	5.632627	5.696443	3.38825	3.352291

Fig. 3D lower panel

Lanes in the figure are numbered here.

Lane 1-4: $TG^{+/+}$

Lane 5-8: $TG^{+/cog}$

Lane 9-12: $TG^{+/cog}$ + T_4

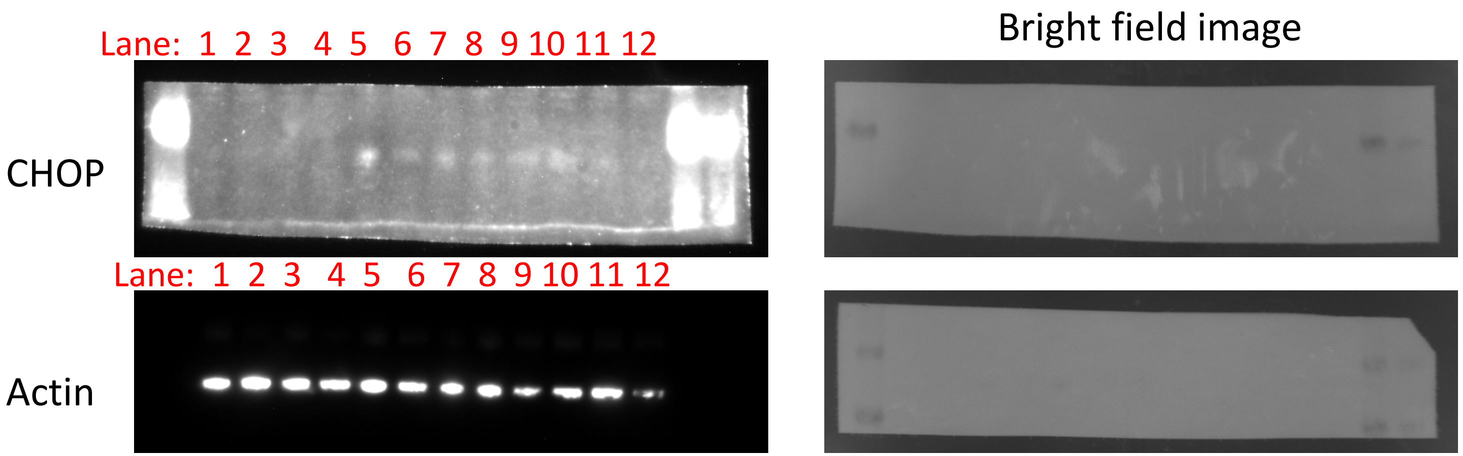

Quantification:

Lane	CHOP	Background (CHOP)	CHOP - background	Actin	Background (Actin)	Actin - background	CHOP/Actin	Mean of control	Fold change
1	5885369	3721736	2163633	29003907	1011	29002896	0.074600585	0.065093	1.146053
2	9902229	9243919	658310	35942744	192781	35749963	0.018414285		0.28289
3	11350425	7050471	4299954	31722453	1010	31721443	0.135553543		2.082445
4	9726000	8790454	935546	29415656	992	29414664	0.031805429		0.488612
5	22363234	7263337	15099897	30585274	0	30585274	0.493698275		7.584453
6	12412057	5924232	6487825	26817026	0	26817026	0.241929325		3.716646
7	21912590	10280257	11632333	24362108	66583	24295525	0.478785003		7.355347
8	24260896	17021554	7239342	25732313	0	25732313	0.281332735		4.321982
9	32261674	23250037	9011637	16114361	0	16114361	0.55923018		8.591188
10	37670769	24071390	13599379	23522818	0	23522818	0.578135621		8.881624
11	29247533	24157940	5089593	30633963	2799	30631164	0.166157349		2.552597
12	25394993	19302172	6092821	8971687	0	8971687	0.679116536		10.43295

Fig. 3E

Lane 1-12 are shown in the figure.

Lane 1-4: *TG*$^{+/+}$

Lane 5-8: *TG*$^{+/cog}$

Lane 9-13: *TG*$^{+/cog}$ + T_4

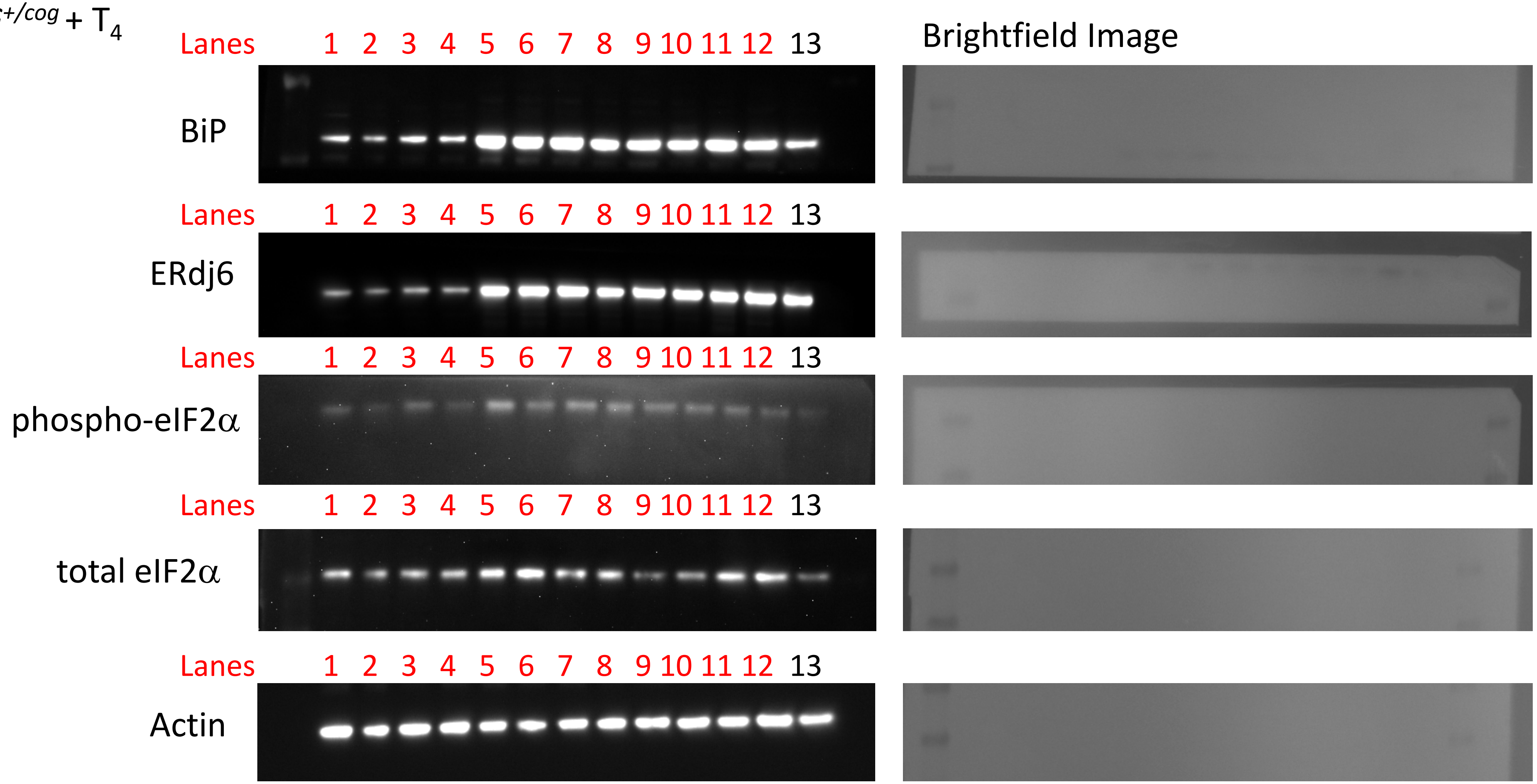

Fig. 3E

Lane 1-12 are shown in the figure.

Lane 1-4: $TG^{+/+}$
Lane 5-8: $TG^{+/cog}$
Lane 9-13: $TG^{+/cog}$ + T_4

Quantification:

	Lane:	1	2	3	4	5	6	7	8	9	10	11	12	13
	BiP	217221	149909	254131	234907	664499	636166	708874	552893	609840	536838	622992	562477	341849
	ERdj6	171035	121403	181728	181271	575569	596055	626737	499684	597327	593690	604217	684620	581841
	p-eIF2a	118880	92173	174418	140275	367651	289755	373819	357907	334640	285820	230273	171735	94030
	t-eIF2a	599077	473463	568222	564947	654939	695716	628021	597681	408396	520517	684297	763099	417552
	Actin1	625360	439393	621047	600874	470515	472129	495338	543525	589538	592966	536333	663826	480252
	Actin2	22248	17374	22751	24700	21111	25643	26745	26036	37071	19523	19829	30814	16226
	BiP /Actin1	0.347354	0.341173	0.409198	0.390942	1.41228	1.347441	1.431091	1.017236	1.034437	0.905344	1.161577	0.847326	0.711812
	ERdj6 / Actin 2	7.687657	6.987625	7.987693	7.338907	27.26394	23.24436	23.4338	19.19204	16.11305	30.40977	30.47138	22.21782	35.85856
	P-eIF2a / Actin2	5.343402	5.305226	7.666388	5.67915	17.41514	11.29957	13.97715	13.74662	9.027002	14.64017	11.61294	5.573278	5.79502
	T-eIF2a /Actin1	0.957971	1.077539	0.914942	0.940209	1.391962	1.473572	1.267864	1.099638	0.692739	0.877819	1.275881	1.149547	0.869444
	P/T-eIF2a	5.57783	4.923467	8.379097	6.040307	12.51122	7.668153	11.02418	12.50104	13.03088	16.67788	9.101901	4.84824	6.665206
Mean of control	BiP	0.372167												
	ERdj6	7.500471												
	P/T-eIF2a	6.230175												
	Lane:	1	2	3	4	5	6	7	8	9	10	11	12	13
Fold change	BiP	0.933328	0.916721	1.099501	1.050449	3.794753	3.620532	3.845298	2.73328	2.7795	2.43263	3.121121	2.276738	1.912616
	ERdj6	1.024957	0.931625	1.064959	0.97846	3.634964	3.099053	3.124311	2.558778	2.148272	4.054382	4.062596	2.962191	4.780841
	P/T-eIF2a	0.857665	0.851537	1.230525	0.911555	2.795289	1.813685	2.243461	2.206458	1.448916	2.349881	1.863983	0.894562	0.930154

Fig. 3E

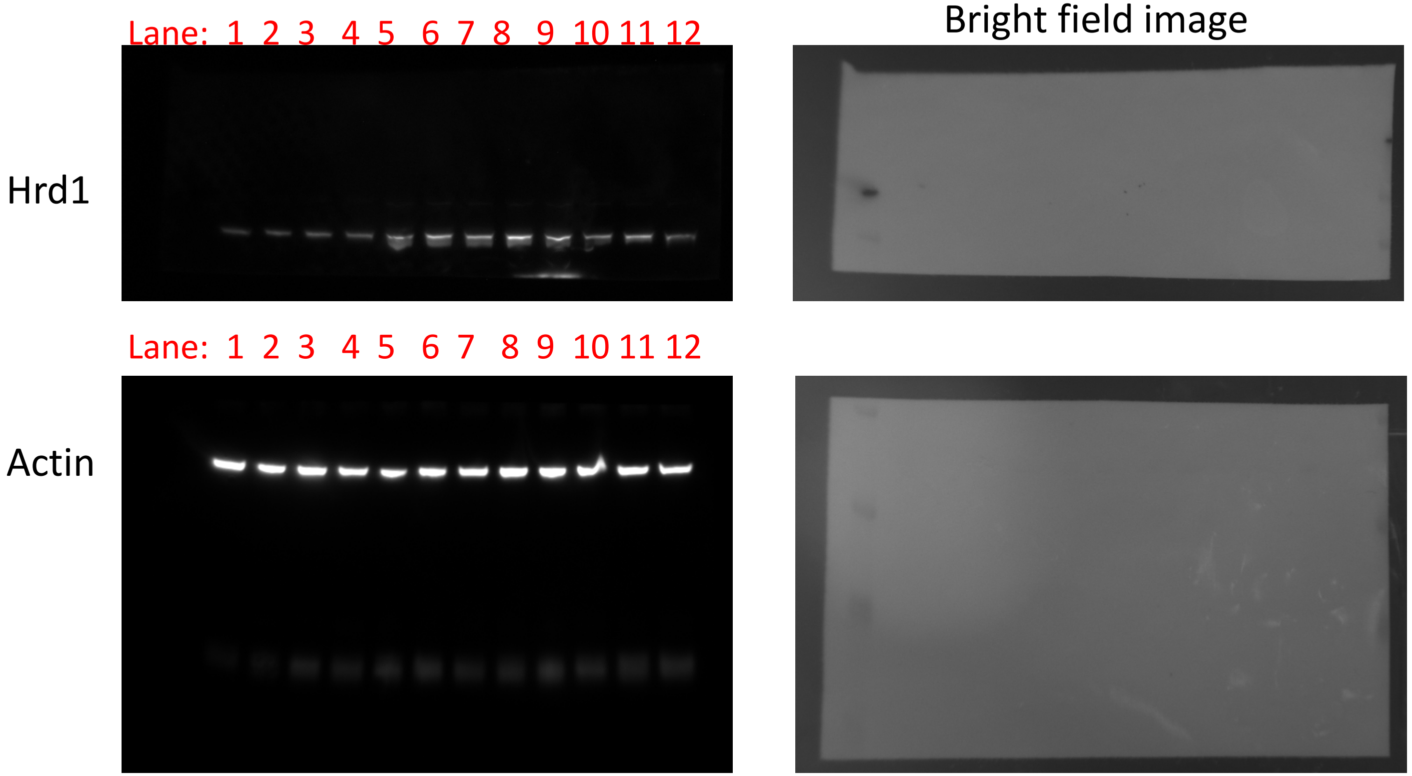

Lanes in the figure are numbered here.
Lane 1-4: $TG^{+/+}$
Lane 5-9: $TG^{+/cog}$
Lane 10-12: $TG^{+/cog}$ + T_4

Lane #9 ($TG^{+/cog}$) is put on a white box to make a final panel with slide#11. Quantification of $TG^{+/cog}$ is made from all 5 lanes (#5-9).

Quantification:

Lane	Hrd1	Background (Hrd1)	Hrd1 - background	Actin	Background (Actin)	Actin - background	Hrd1/Actin	Mean of control	Fold change
1	2608512	526256	2082256	18543883	879888	17663995	0.11788137	0.171072	0.689074
2	2923200	583056	2340144	15988681	1692976	14295705	0.1636956		0.956881
3	3794816	837136	2957680	18368756	2098720	16270036	0.18178694		1.062633
4	4113520	1189520	2924000	14664215	1428928	13235287	0.22092456		1.291412
5	6146048	1676304	4469744	12410154	1126912	11283242	0.39614005		2.315632
6	7802848	1557168	6245680	15199497	1123008	14076489	0.44369587		2.593619
7	7154128	1406256	5747872	14604862	805056	13799806	0.41651832		2.434753
8	9388256	1440960	7947296	18989444	954000	18035444	0.44064876		2.575807
9	7825456	1737312	6088144	18552866	1146976	17405890	0.34977493		2.044605
10	5526544	1029552	4496992	17047328	915264	16132064	0.27876111		1.629495
11	7202592	1017648	6184944	16283543	652304	15631239	0.39567842		2.312933
12	4478816	749840	3728976	13827449	509168	13318281	0.27998929		1.636674

Fig 3F TUNEL DAPI

These images were used in the figure as representative.

Tag# 17175 *TG*$^{+/+}$ | Tag# 17472 *TG*$^{+/cog}$ | Tag# 17996 *TG*$^{+/cog}$ + T_4

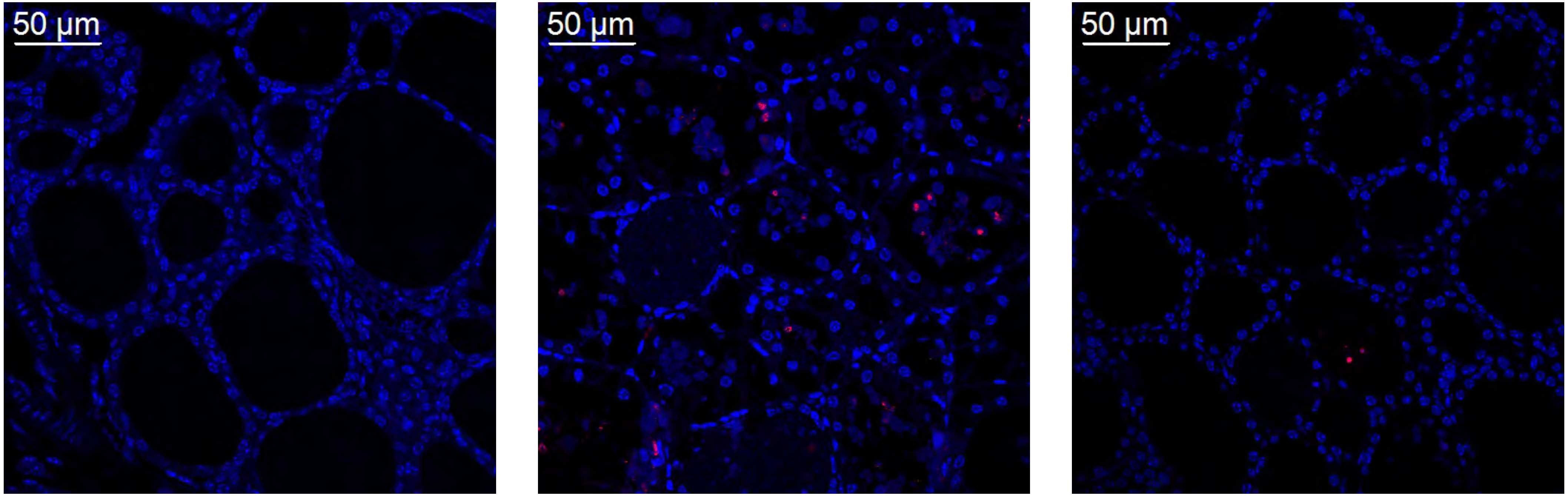

Fig 3F TUNEL DAPI

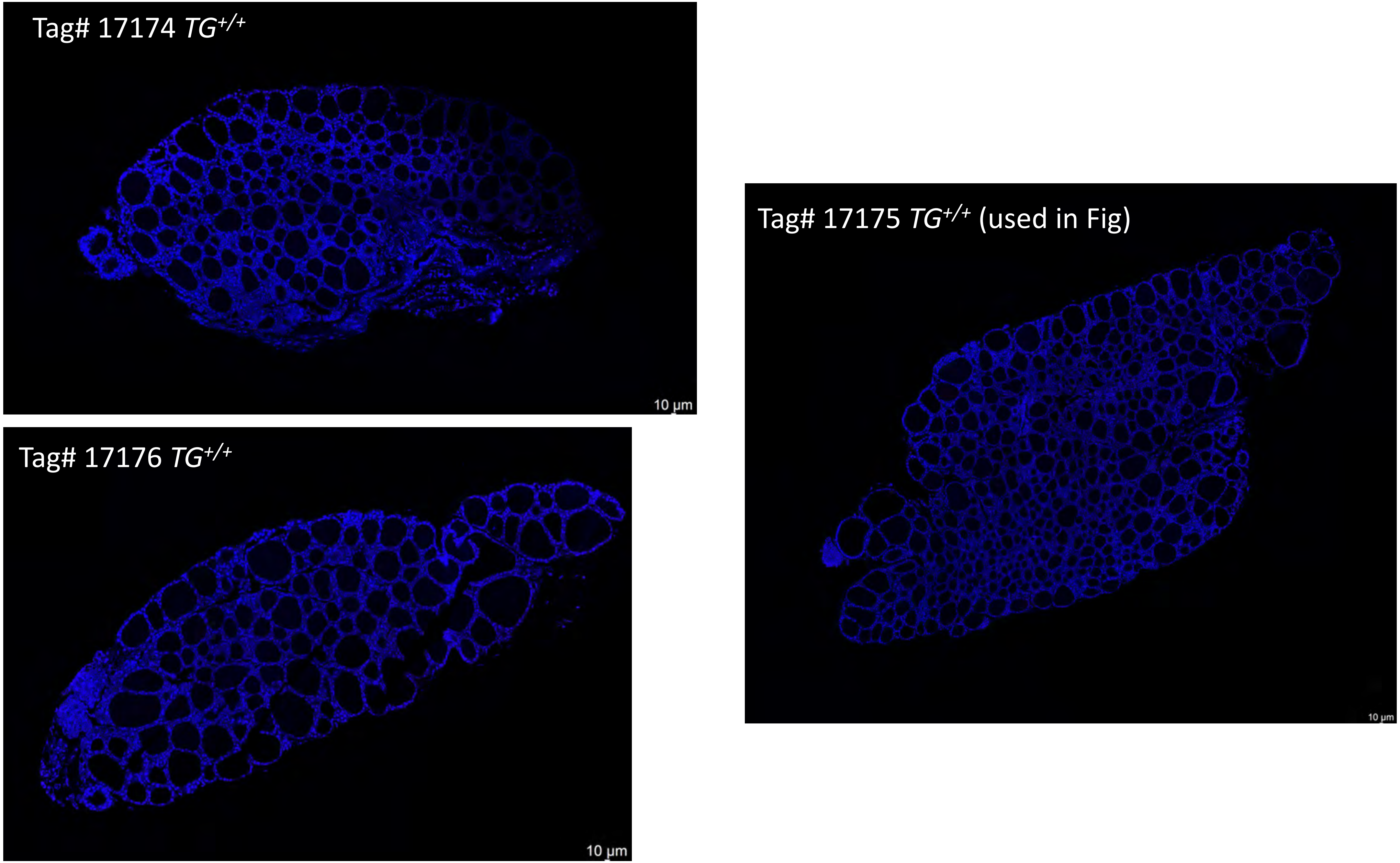

Fig 3F

TUNEL DAPI

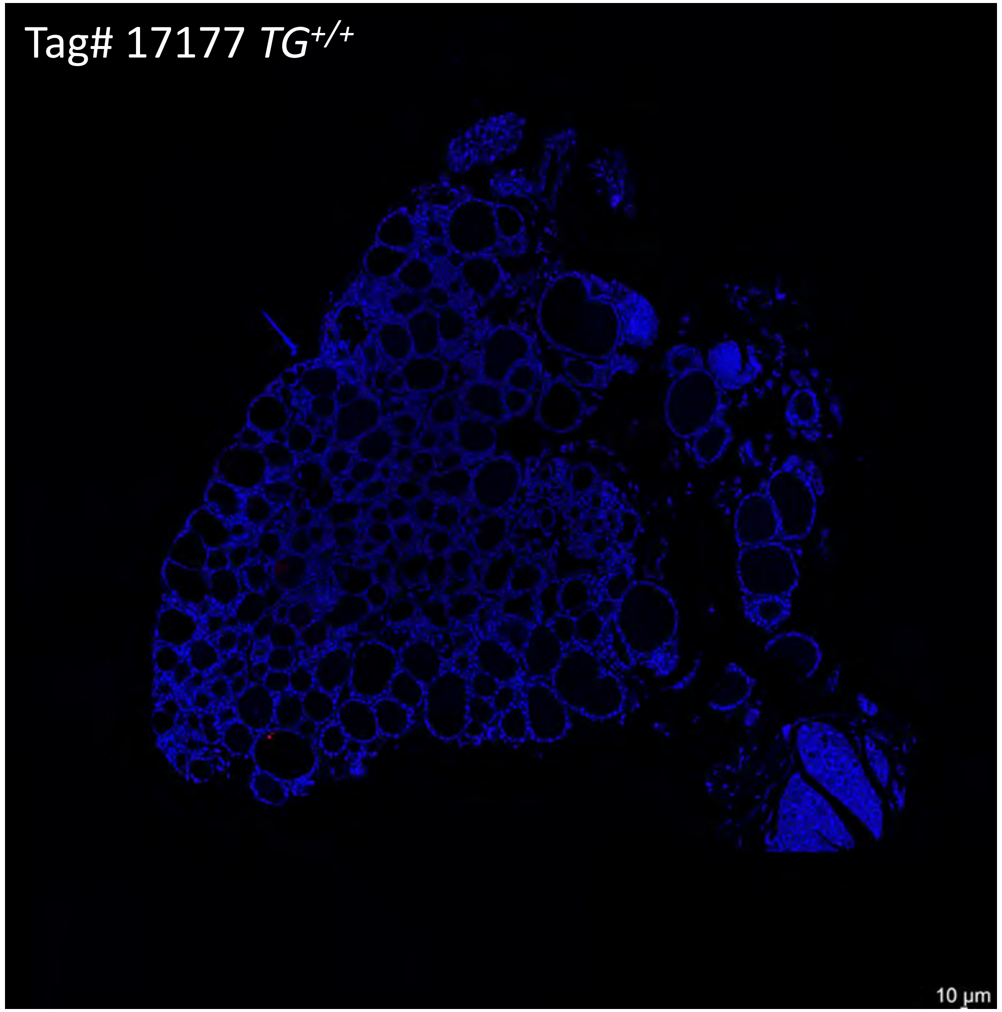

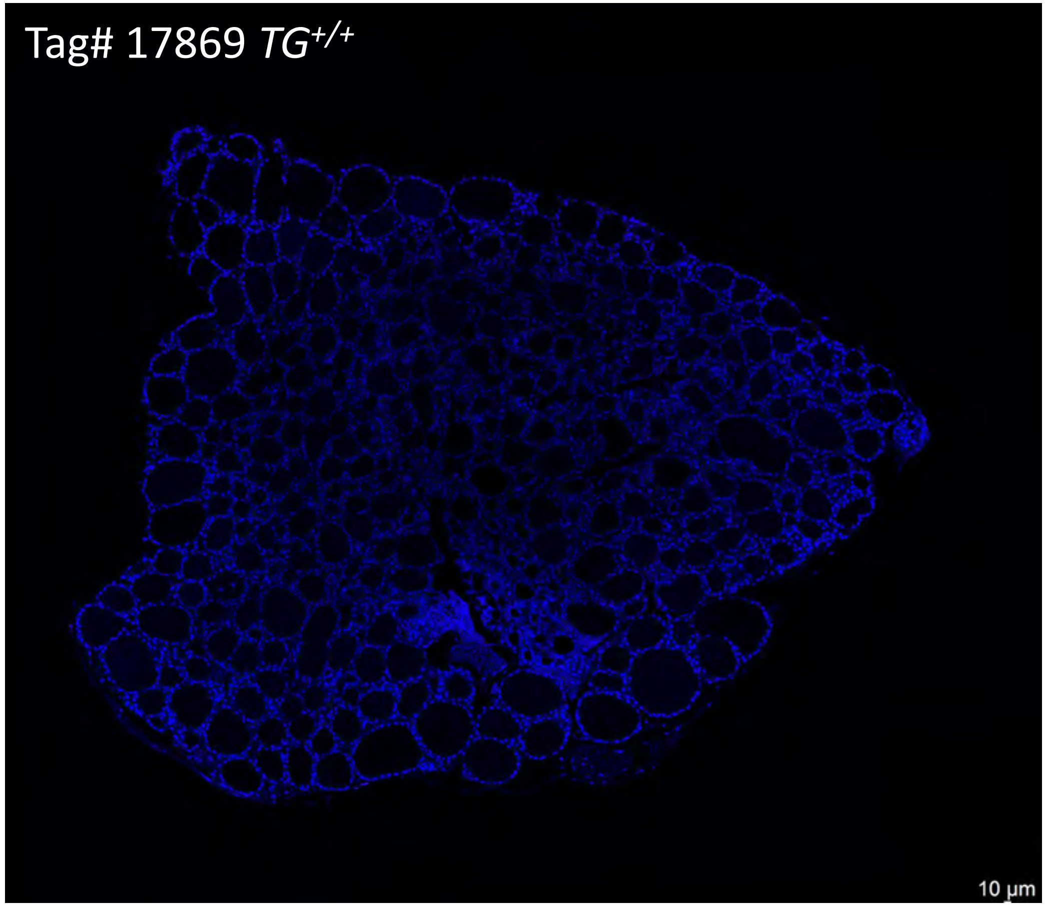

Fig 3F TUNEL DAPI

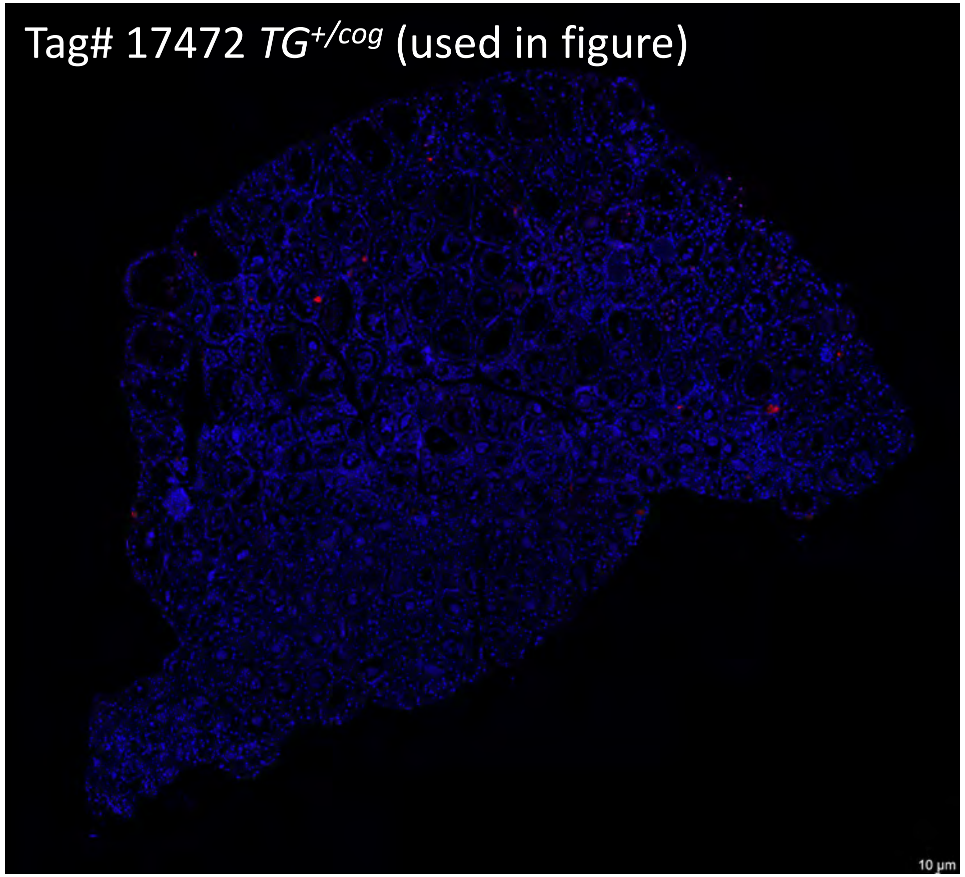

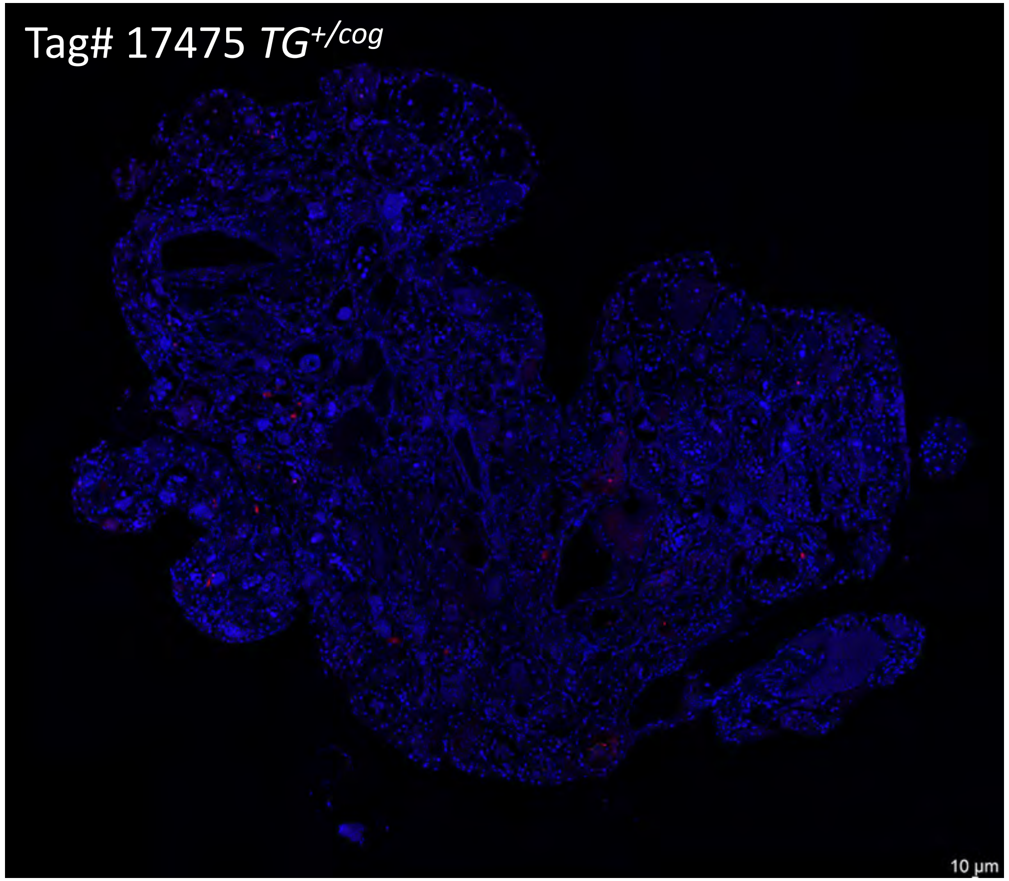

Fig 3F

TUNEL DAPI

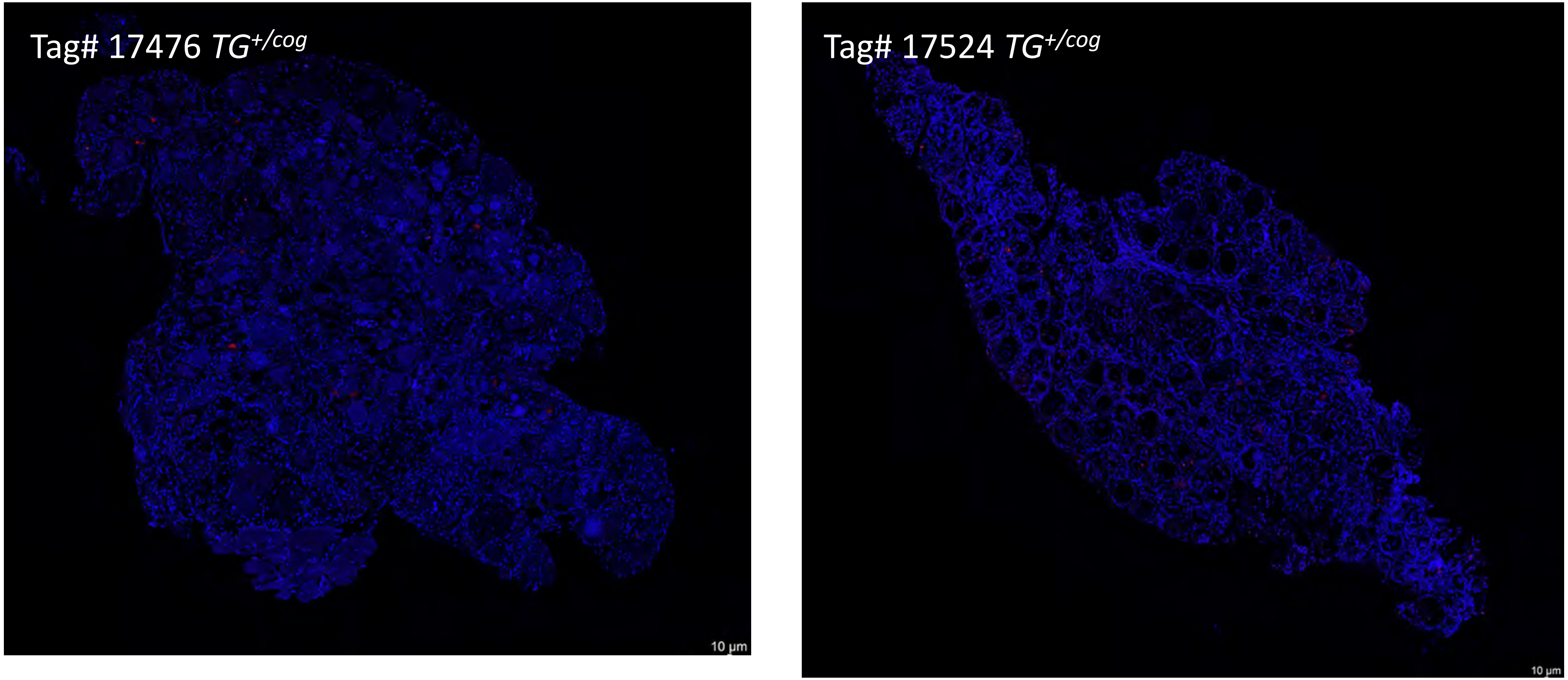

Fig 3F

TUNEL DAPI

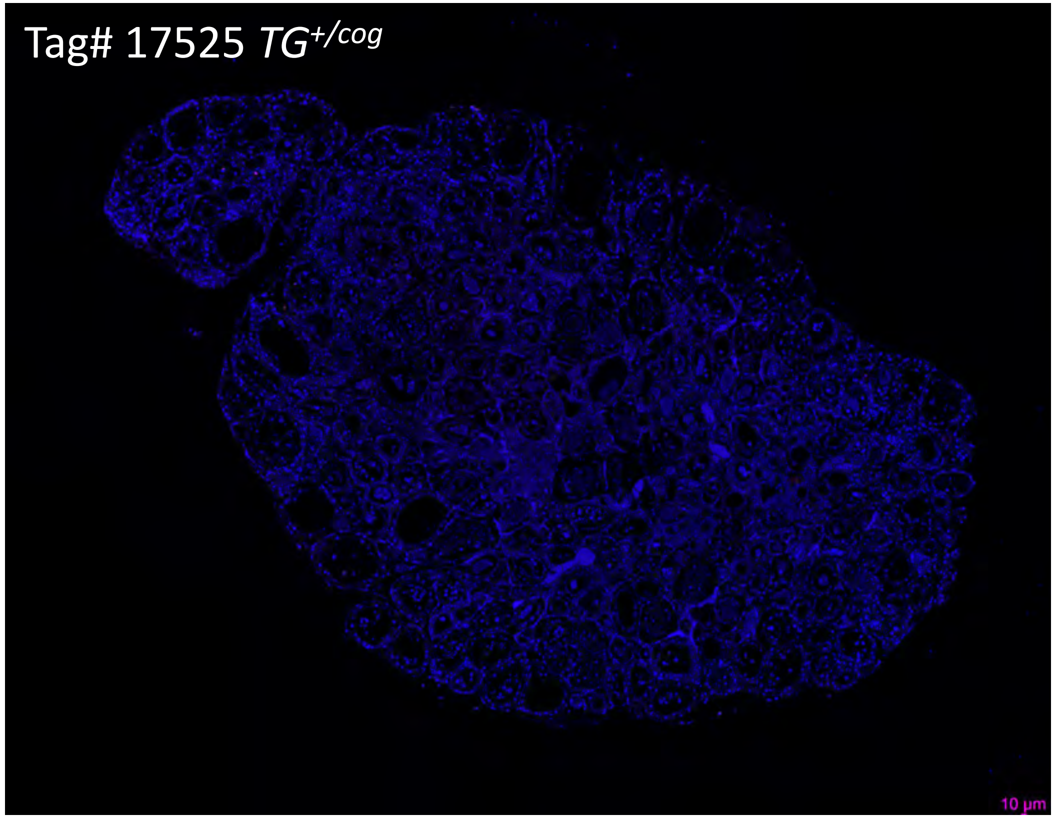

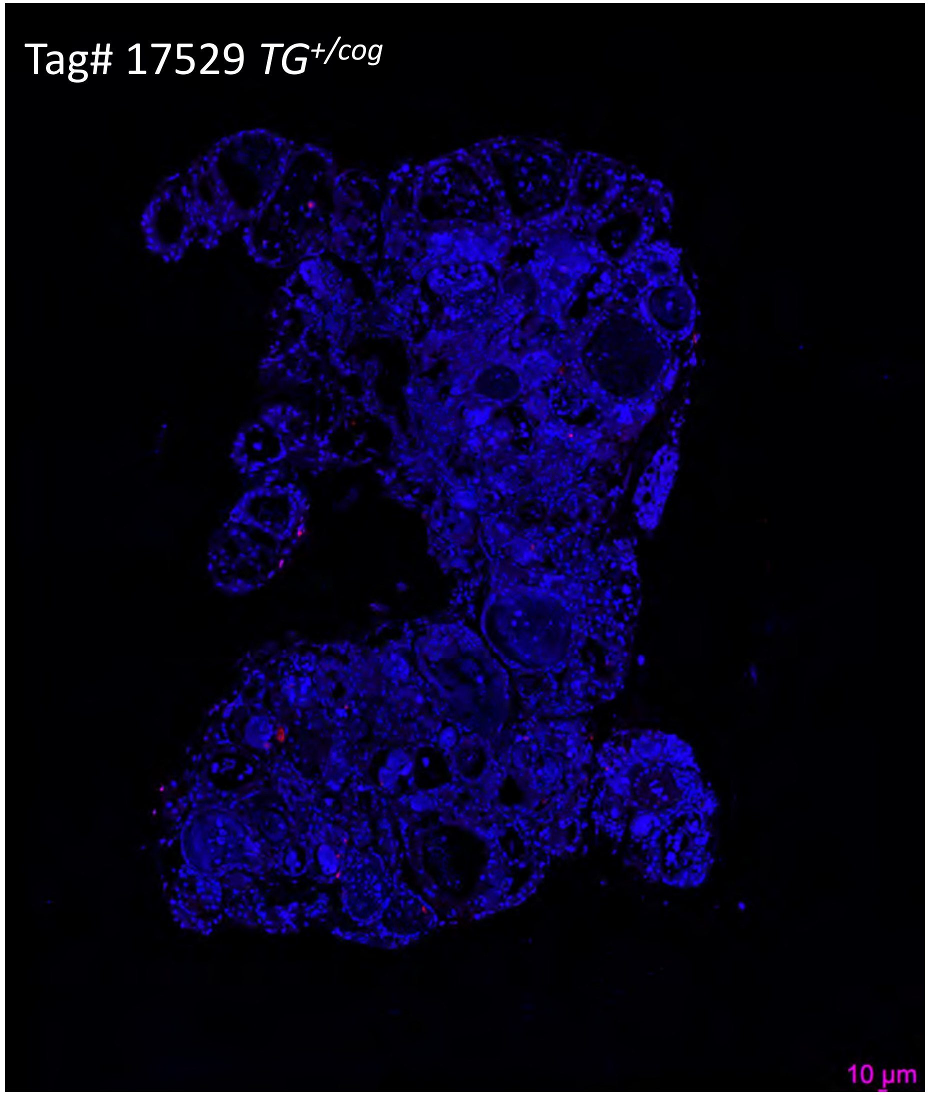

Fig3F

TUNEL DAPI

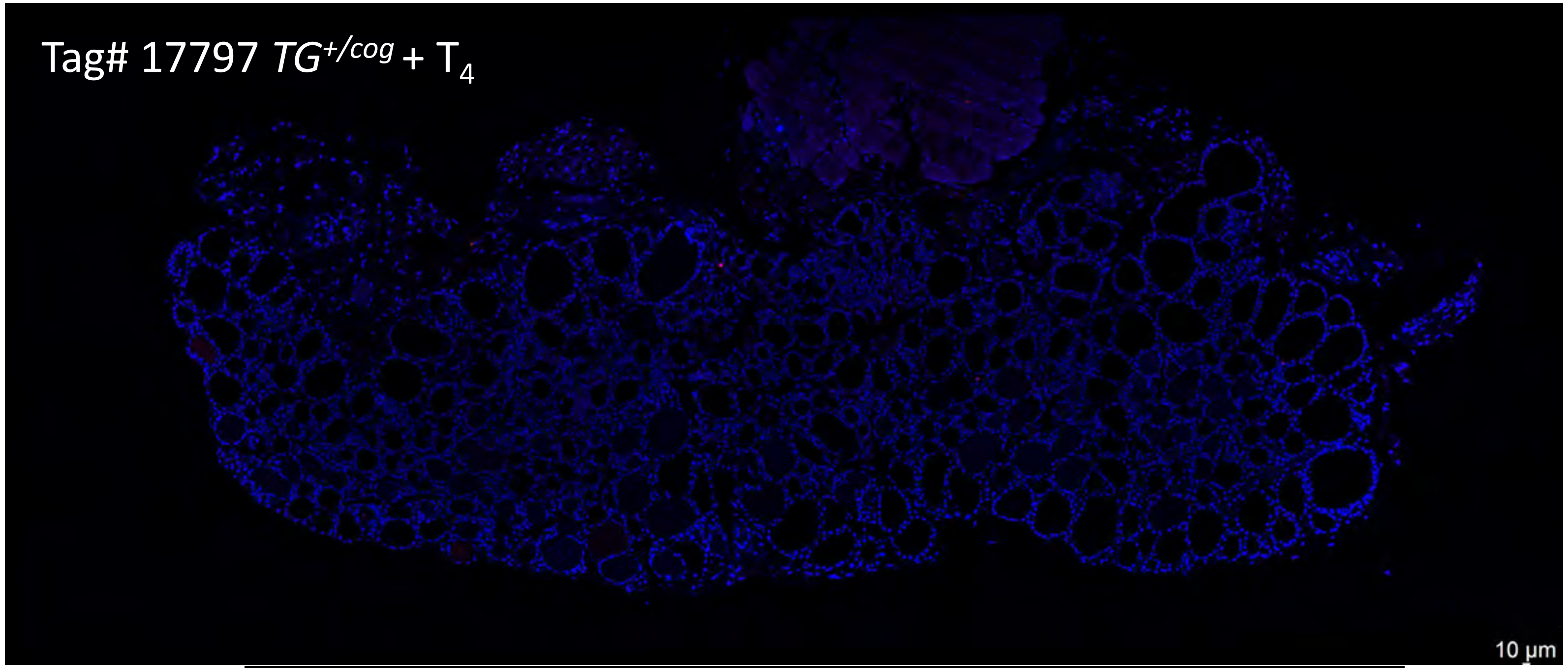

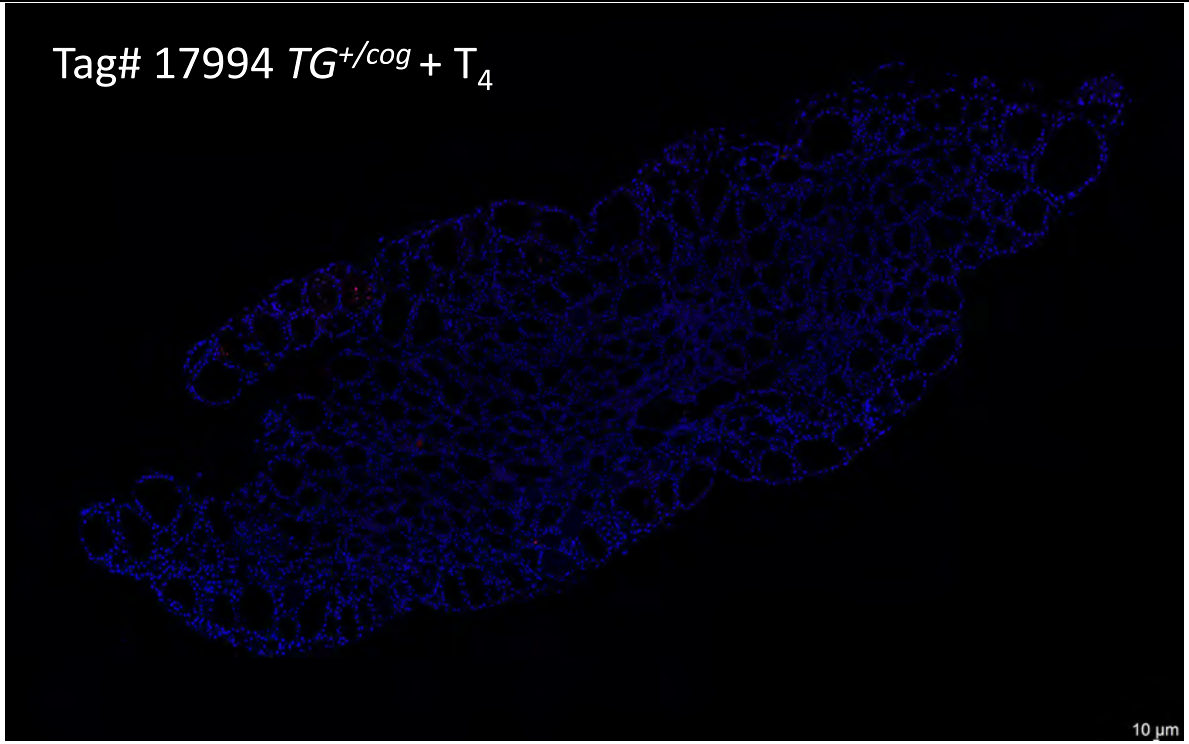

Fig 3F

TUNEL DAPI

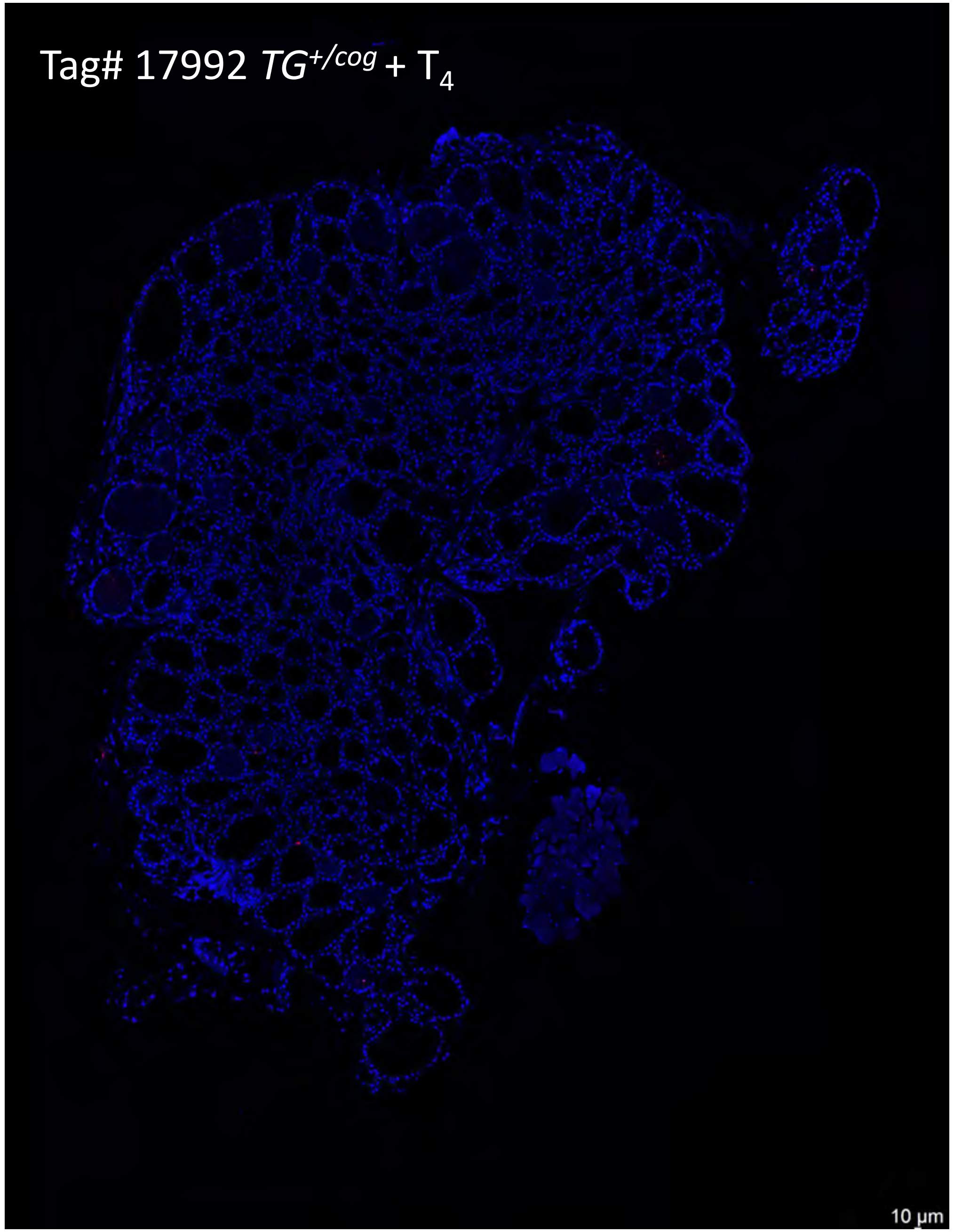

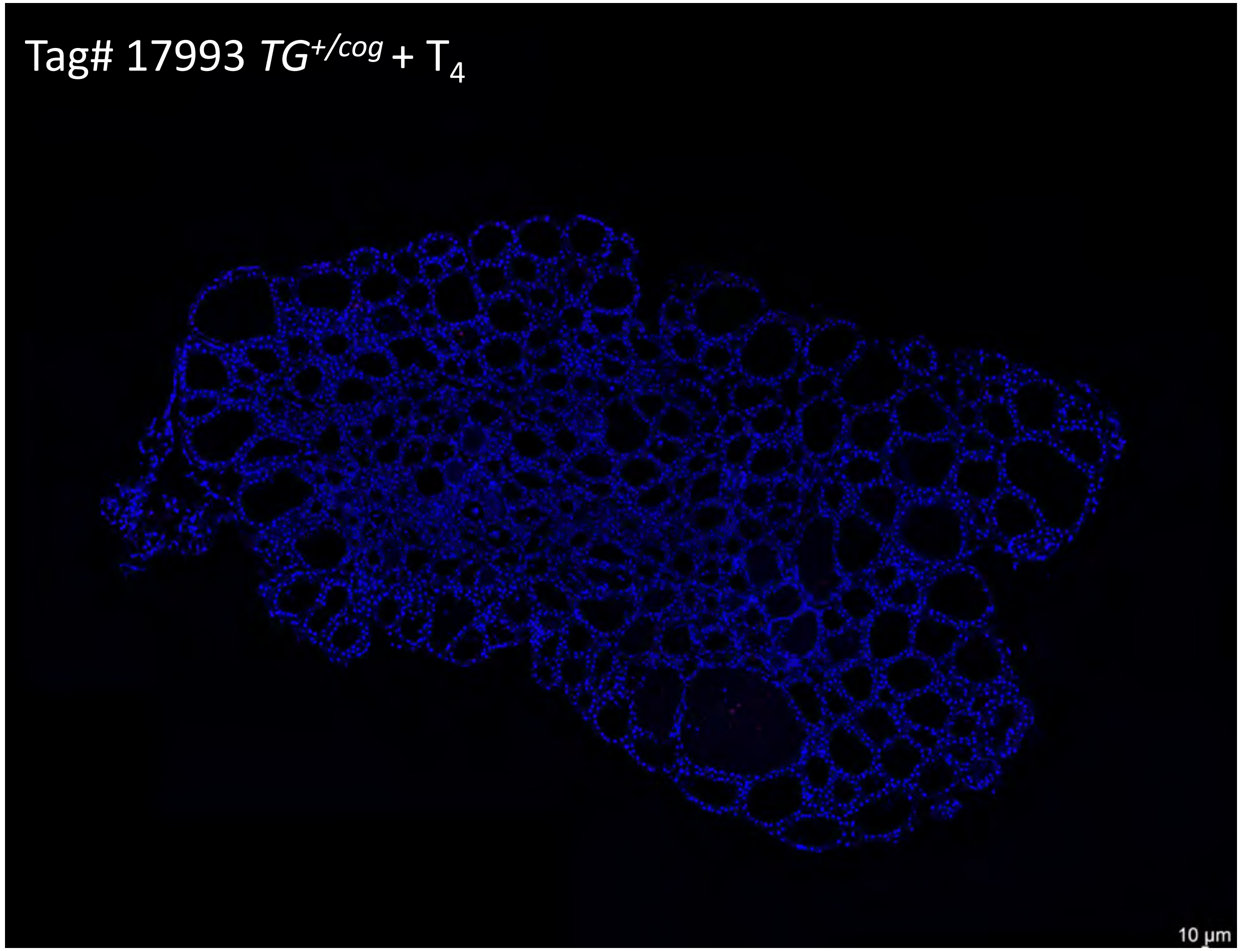

Fig 3F

TUNEL DAPI

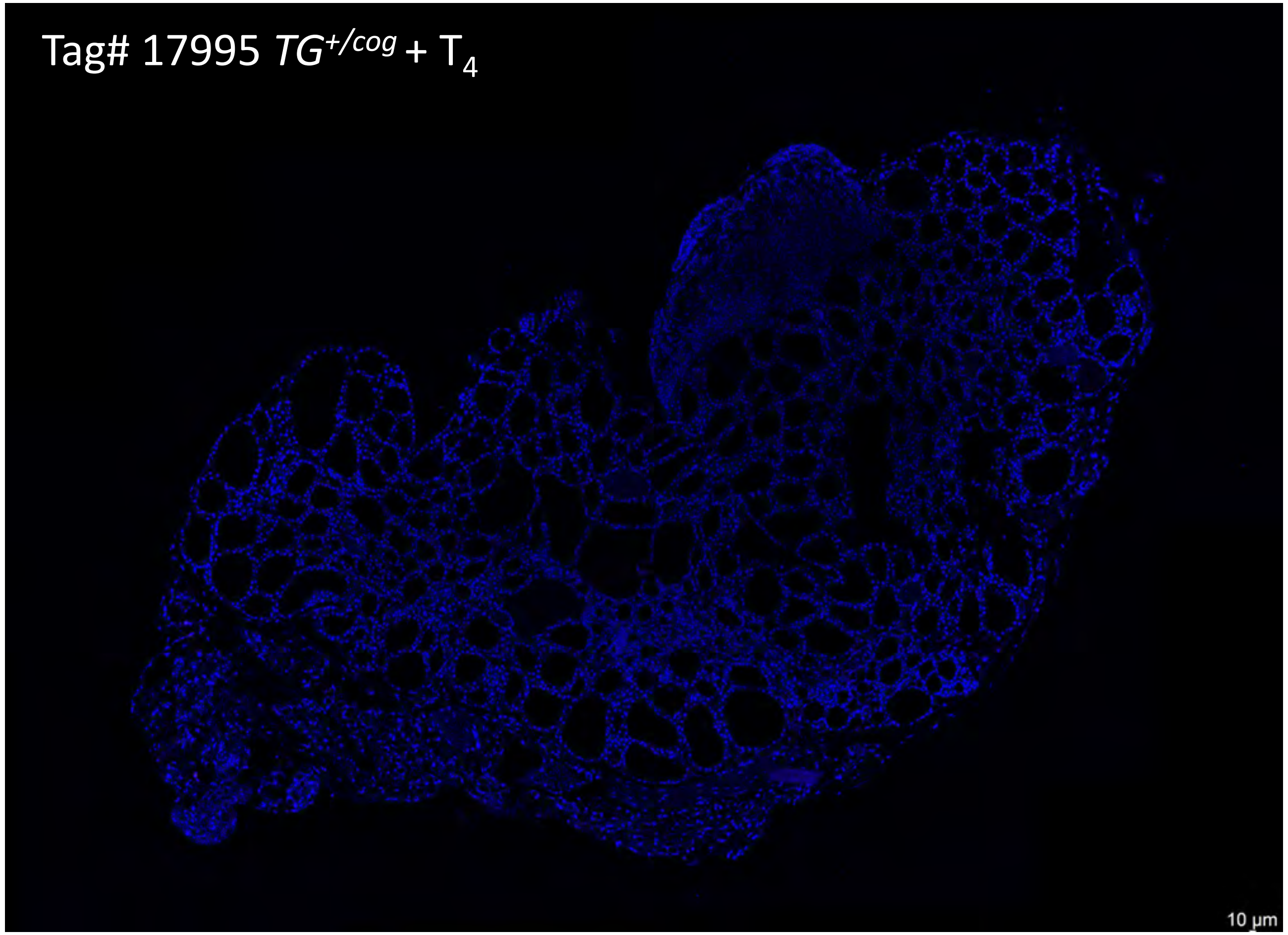

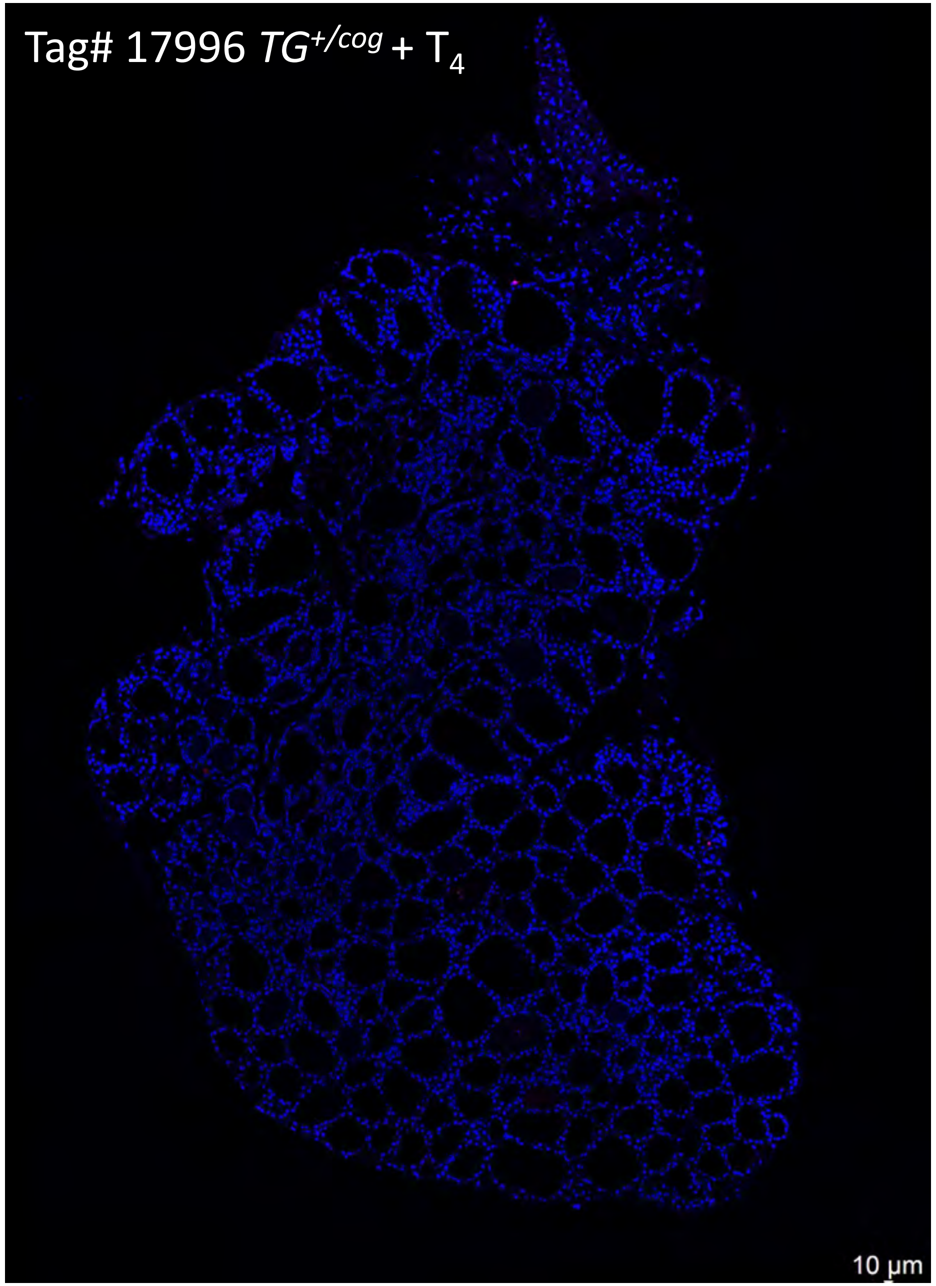

Fig 3F

TUNEL DAPI

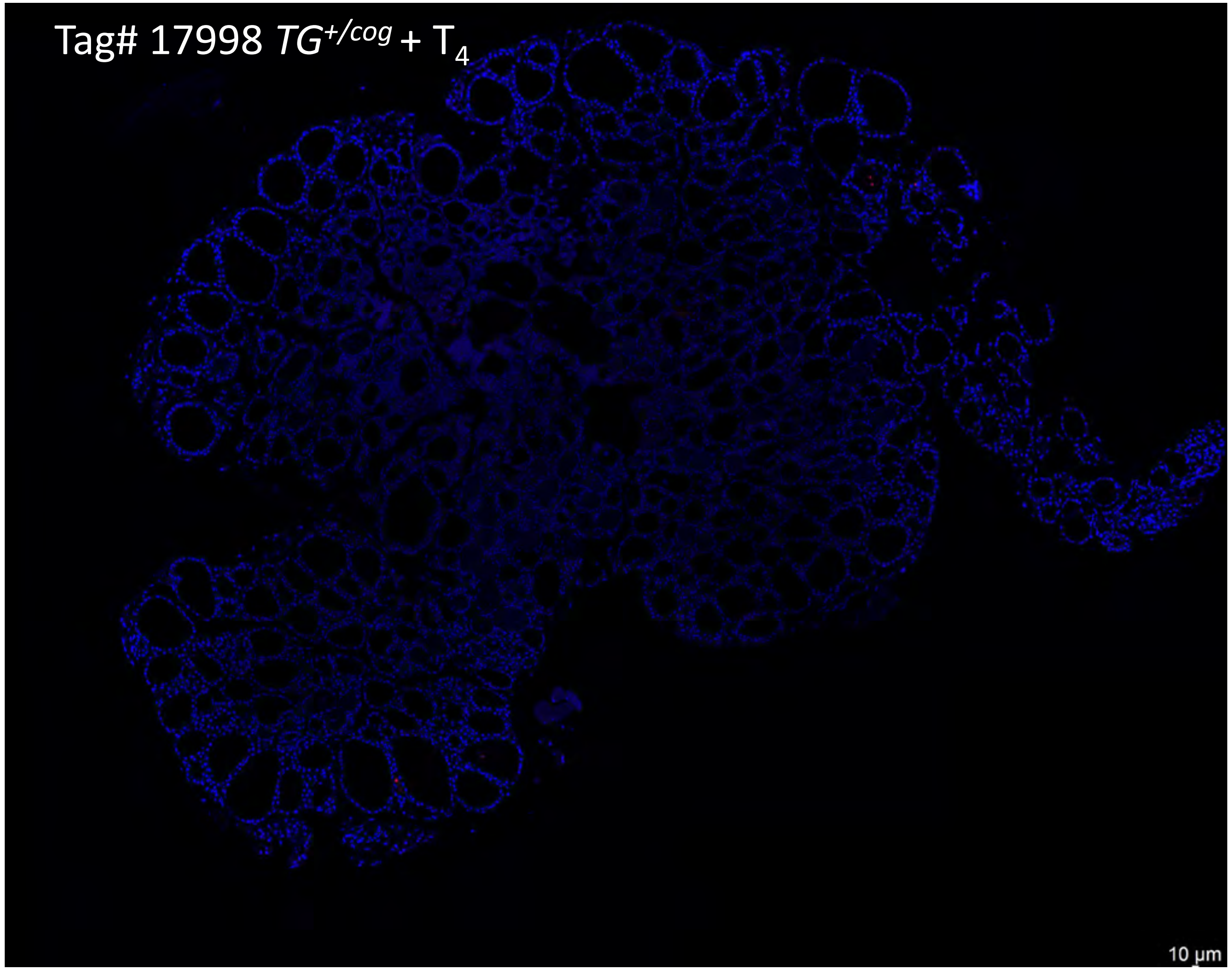

Quantification:

Tag	Genotype	Gender	Total Follicle	TUNEL positive follicle	Percentage TUNEL Positive Follicles
17174	*TG*$^{+/+}$	M	144	1	0.6944444444
17175	*TG*$^{+/+}$	F	373	5	1.340482574
17176	*TG*$^{+/+}$	F	107	2	1.869158879
17177	*TG*$^{+/+}$	F	188	4	2.127659574
17869	*TG*$^{+/+}$	F	284	1	0.3521126761
17472	*TG*$^{+/cog}$	M	318	70	22.01257862
17475	*TG*$^{+/cog}$	F	196	54	27.55102041
17476	*TG*$^{+/cog}$	F	258	35	13.56589147
17524	*TG*$^{+/cog}$	M	248	66	26.61290323
17525	*TG*$^{+/cog}$	M	233	31	13.30472103
17529	*TG*$^{+/cog}$	F	70	25	35.71428571
17797	*TG*$^{+/cog}$ + T_4	F	258	10	3.875968992
17992	*TG*$^{+/cog}$ + T_4	M	248	10	4.032258065
17993	*TG*$^{+/cog}$ + T_4	M	244	10	4.098360656
17994	*TG*$^{+/cog}$ + T_4	M	316	14	4.430379747
17995	*TG*$^{+/cog}$ + T_4	F	265	2	0.7547169811
17996	*TG*$^{+/cog}$ + T_4	F	210	8	3.80952381
17998	*TG*$^{+/cog}$ + T_4	F	327	8	2.44648318

Fig. 3G

Gel1 is shown in the figure.

Lane 1-2: *TG*$^{+/+}$

Lane 3-4: *TG*$^{+/cog}$

Lane 5-6: *TG*$^{+/cog}$ + T_4

Gel 1

Lane: 1 2 3 4 5 6

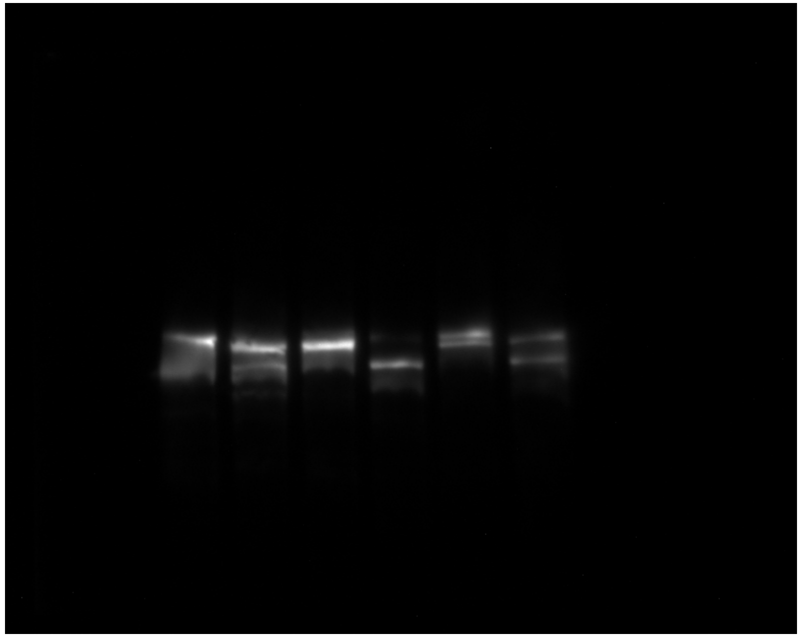

Bright field image

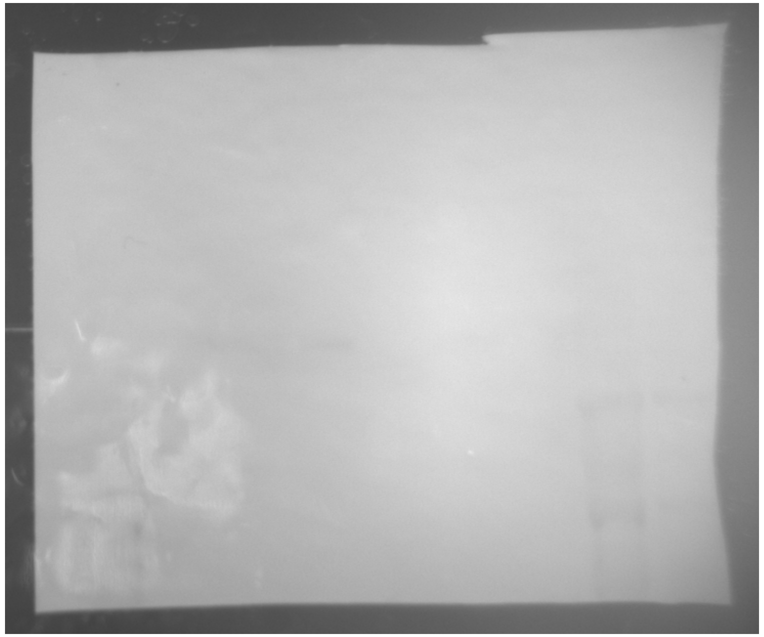

Gel 2

Lane 1-2: *TG*$^{+/+}$

Lane 3-4: *TG*$^{+/cog}$

Lane 5-6: *TG*$^{+/cog}$ + T_4

Lane 7-8: *TG*$^{+/+}$

Gel 3

Lane 1-2: *TG*$^{+/+}$

Lane 3-4: *TG*$^{+/cog}$

Lane 5-6: *TG*$^{+/cog}$ + T_4

Lane 7-8: *TG*$^{+/cog}$

Lane: 1 2 3 4 5 6 7 8 Lane: 1 2 3 4 5 6 7 8

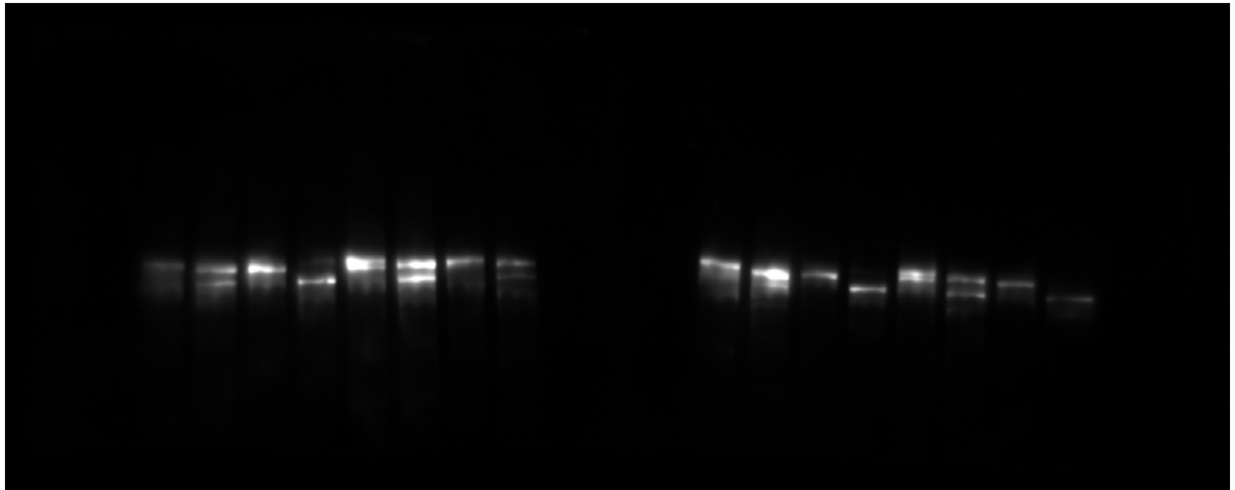

Bright field image

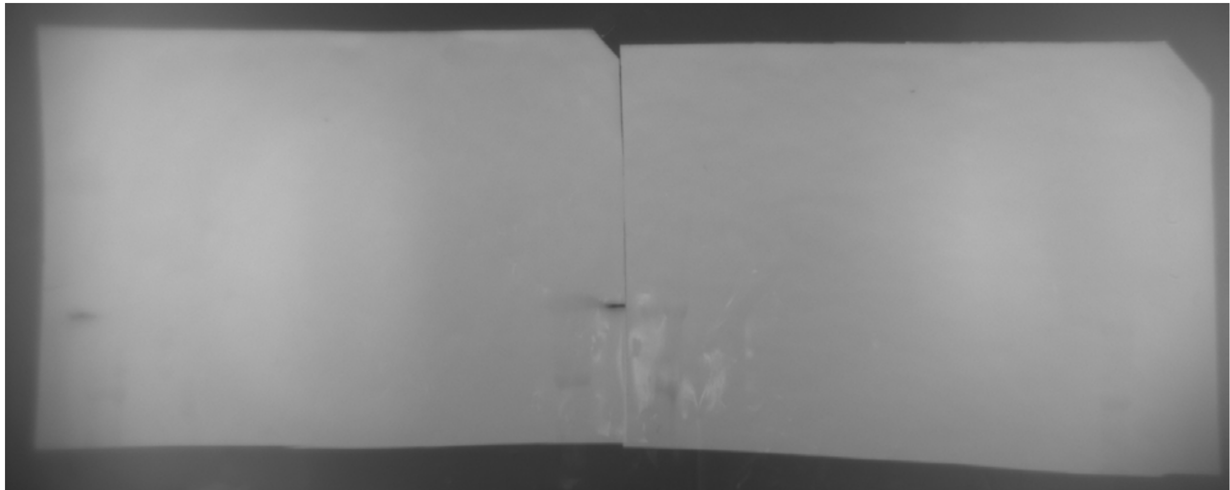

Fig. 3G

Quantification:

Gel 1	Lane	band intensity	resistant	sensitive	resistant / sensitive	
	1	29838624	29838624	18466400	1.615833297	$TG^{+/+}$
	2	18466400				
	3	2825504	2825504	13044624	0.216602947	$TG^{+/cog}$
	4	13044624				
	5	10039024	10039024	11025376	0.910538017	$TG^{+/cog}+T_4$
	6	11025376				
Gel 2	Lane	band intensity	resistant	sensitive	resistant / sensitive	
	1	17378016	17378016	13518112	1.285535732	$TG^{+/+}$
	2	13518112				
	3	19363056	19363056	53860437	0.35950425	$TG^{+/cog}$
	4	53860437				
	5	34302537	34302537	31617008	1.084939378	$TG^{+/cog}+T_4$
	6	31617008				
	7	25541776	25541776	16571472	1.54131003	$TG^{+/+}$
	8	16571472				
Gel 3	Lane	band intensity	resistant	sensitive	resistant / sensitive	
	1	47680713	47680713	25999088	1.833937906	$TG^{+/+}$
	2	25999088				
	3	7353184	7353184	36432849	0.20182841	$TG^{+/cog}$
	4	36432849				
	5	33029988	33029988	29140219	1.133484549	$TG^{+/cog}+T_4$
	6	29140219				
	7	8588032	8588032	34704930	0.24745856	$TG^{+/cog}$
	8	34704930				

Fig. 4A
Left 3 panels

Mouse tag#	Genotype	TSH (mU/L)	T_4 (µg/dL)	T_3 (ng/dL)
17514	$TG^{+/cog}$; $ATG7^{control}$	211	2.43	75.4
17517	$TG^{+/cog}$; $ATG7^{control}$	125	3.74	88.3
17501	$TG^{+/cog}$; $ATG7^{control}$	249	4.2	108.3
17549	$TG^{+/cog}$; $ATG7^{control}$	176	3.42	101.6
17566	$TG^{+/cog}$; $ATG7^{control}$	46	3.36	71.8
17565	$TG^{+/cog}$; $ATG7^{control}$	146	3	86.7
17515	$TG^{+/cog}$; $ATG7^{TPO}$	961	3.32	108.3
17509	$TG^{+/cog}$; $ATG7^{TPO}$	735	1.38	79.0
17564	$TG^{+/cog}$; $ATG7^{TPO}$	962	2.45	63.6
17511	$TG^{+/cog}$; $ATG7^{TPO}$	75	3.42	69.8
17512	$TG^{+/cog}$; $ATG7^{TPO}$	364	2.99	54.9

Fig. 4A
Right panel

$TG^{+/cog};ATG7^{control}$
Tag#16926

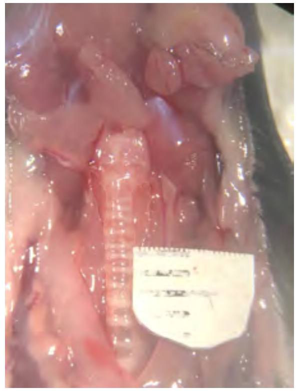

$TG^{+/cog};ATG7^{control}$
Tag#17145

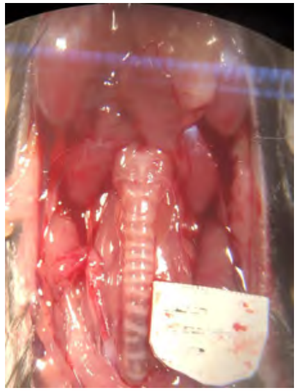

$TG^{+/cog};ATG7^{control}$
Tag#17144

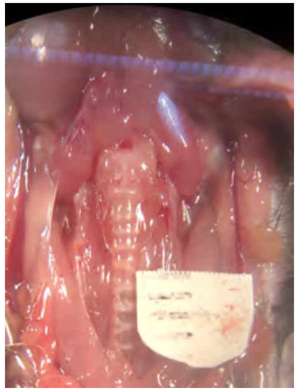

$TG^{+/cog};ATG7^{control}$
Tag#17565

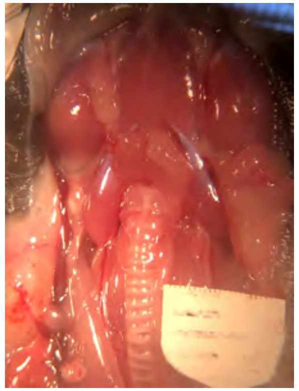

$TG^{+/cog};ATG7^{control}$
Tag#17566

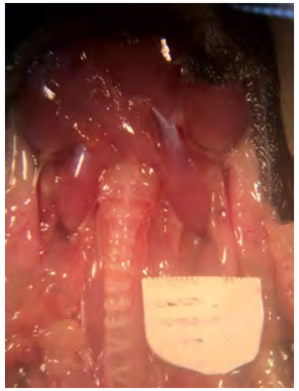

$TG^{+/cog};ATG7^{control}$
Tag#17373

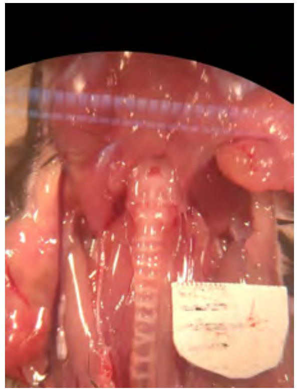

$TG^{+/cog};ATG7^{control}$
Tag#17320

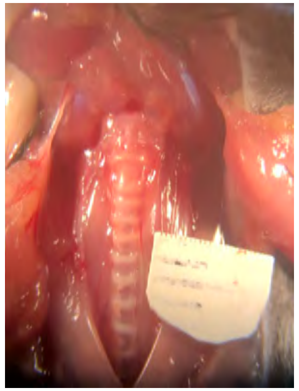

$TG^{+/cog};ATG7^{TPO}$
Tag#16976

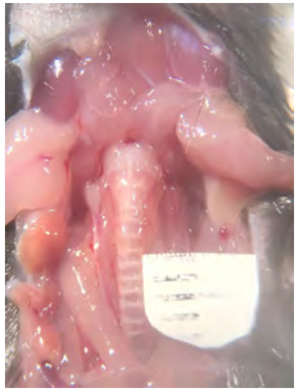

$TG^{+/cog};ATG7^{TPO}$
Tag#17146

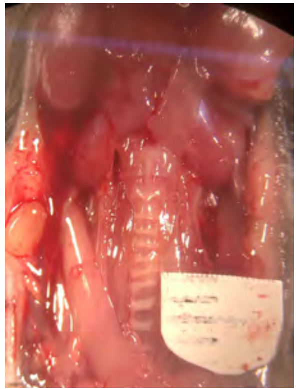

$TG^{+/cog};ATG7^{TPO}$
Tag#17147

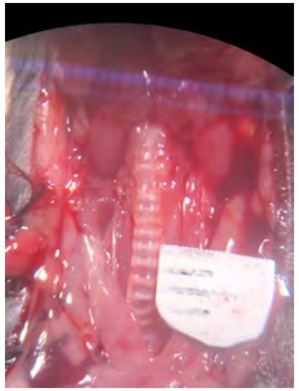

$TG^{+/cog};ATG7^{TPO}$
Tag#17375

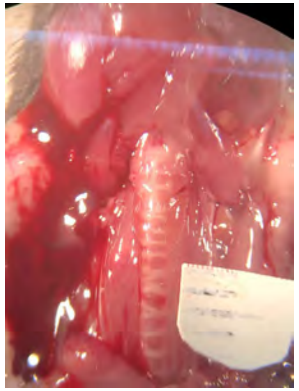

$TG^{+/cog};ATG7^{TPO}$
Tag#17564

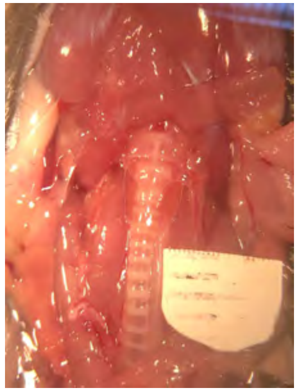

$TG^{+/cog};ATG7^{TPO}$
Tag#17306

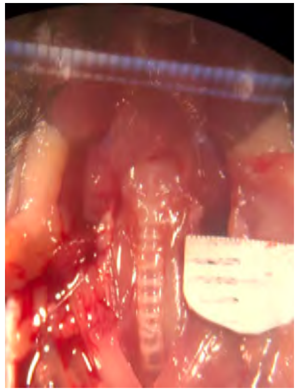

$TG^{+/cog};ATG7^{TPO}$
Tag#17305

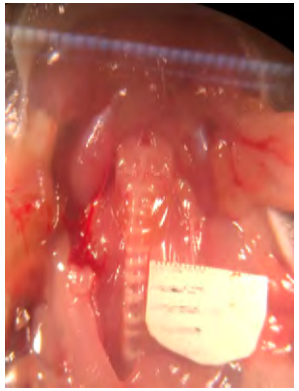

Fig. 4A
Right panel

Quantification:

#tag	*Genotype*	Body weight (g)	area_left lobe (mm^2)	area_right lobe (mm2)	total area (mm2)	thyroid area / body weight
16926	*TG$^{+/cog}$; ATG7^{control}*	20.1	1.189	2.28	3.469	0.172587065
17145	*TG$^{+/cog}$; ATG7^{control}*	20.5	1.111	2.304	3.415	0.166585366
17144	*TG$^{+/cog}$; ATG7^{control}*	24.1	2.029	2.26	4.289	0.177966805
17565	*TG$^{+/cog}$; ATG7^{control}*	17.9	1.178	1.63	2.808	0.156871508
17566	*TG$^{+/cog}$; ATG7^{control}*	23.8	1.565	1.859	3.424	0.143865546
17373	*TG$^{+/cog}$; ATG7^{control}*	22.5	1.087	2.353	3.44	0.152888889
17320	*TG$^{+/cog}$; ATG7^{control}*	29.6	2.142	2.994	5.136	0.173513514
16976	*TG$^{+/cog}$; ATG7^{TPO}*	19	3.122	3.063	6.185	0.325526316
17146	*TG$^{+/cog}$; ATG7^{TPO}*	20	2.419	2.606	5.025	0.25125
17147	*TG$^{+/cog}$; ATG7^{TPO}*	20.7	1.121	2.909	4.03	0.19468599
17375	*TG$^{+/cog}$; ATG7^{TPO}*	21.4	1.547	2.199	3.746	0.175046729
17564	*TG$^{+/cog}$; ATG7^{TPO}*	24.3	1.442	2.224	3.666	0.150864198
17306	*TG$^{+/cog}$; ATG7^{TPO}*	26.1	1.766	3.964	5.73	0.21954023
17305	*TG$^{+/cog}$; ATG7^{TPO}*	25	1.849	3.691	5.54	0.2216

Fig. 4B

Representative image $TG^{+/cog};ATG7^{control}$

Tag#17144

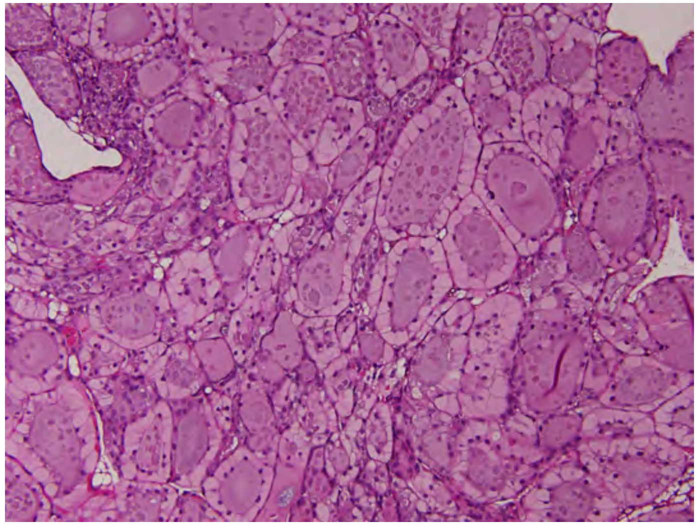

$TG^{+/cog};ATG7^{control}$

Tag#17145

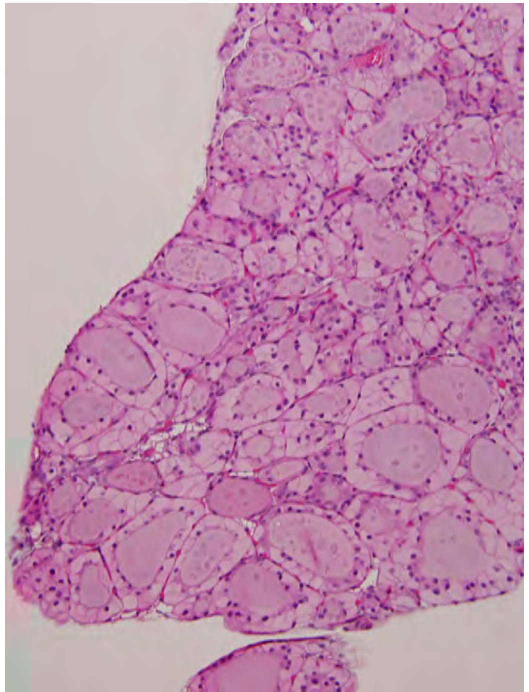

$TG^{+/cog};ATG7^{control}$

Tag#16926

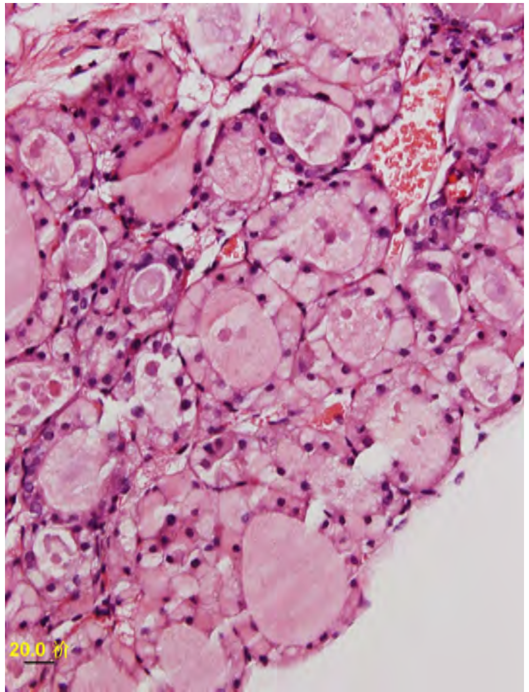

Representative image $TG^{+/cog};ATG7^{TPO}$

Tag#17146

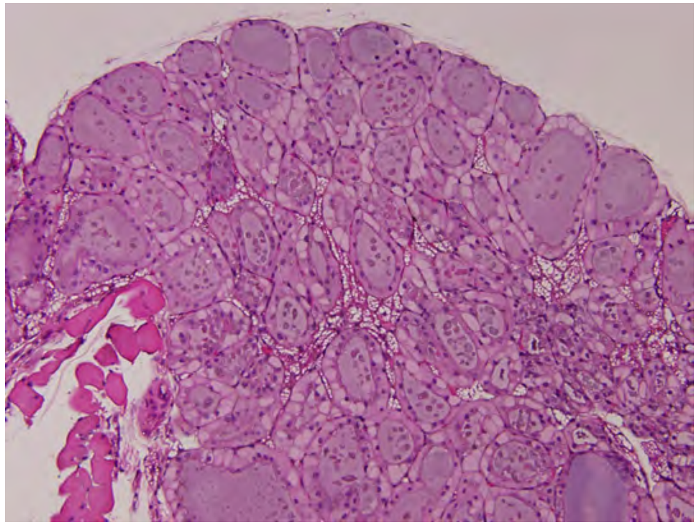

$TG^{+/cog};ATG7^{TPO}$

Tag#17147

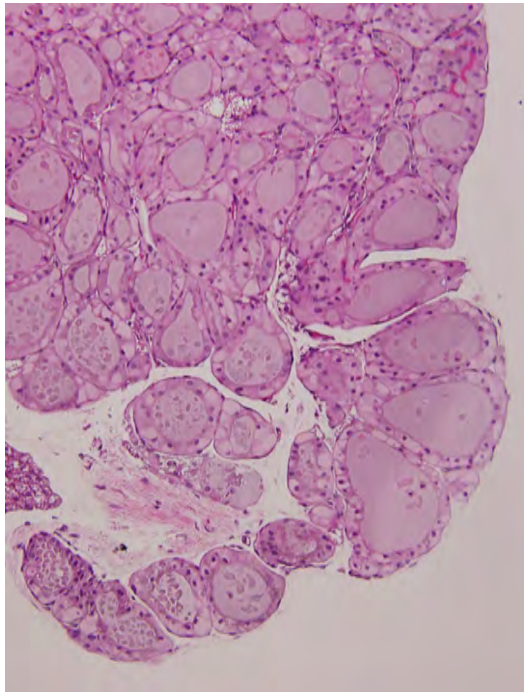

$TG^{+/cog};ATG7^{TPO}$

Tag#16976

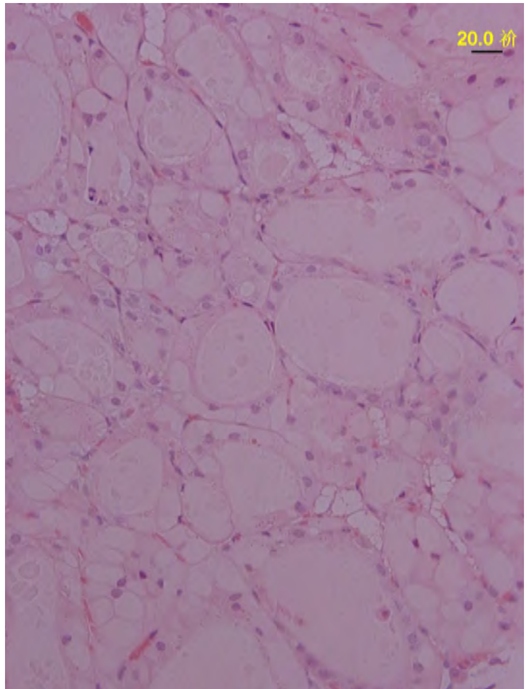

Fig. 4C

Lanes 1-6: *TG*$^{+/cog}$; *ATG7*^{control}

Lanes 7-10: *TG*$^{+/cog}$; *ATG7*^{TPO}

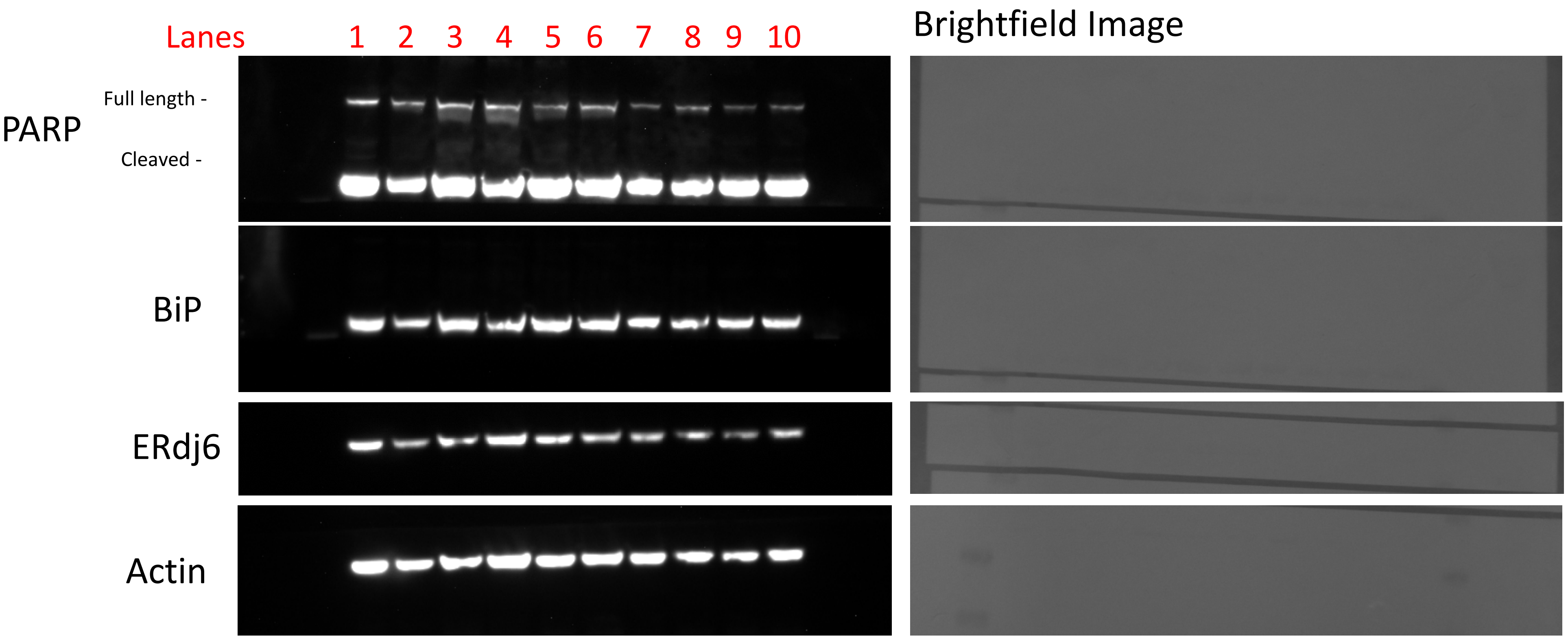

Quantitation

	Actin			Cl Parped					BiP					ERdj6				
Lane	Band	Background	Band-background	Band	Background	Band-background	Cl/Actin	Normalized	Band	Background	Band-background		Normalized	Band	Background	Band-background		Normalized
1	1711710	170033	1541677	969403	59566	909837	0.59016058	1.04573167	1639488	90388	1549100	1.00481489	1.0053625	1274481	161203	1113278	0.72212143	1.18843509
2	1427126	144503	1282623	617809	65718	552091	0.43043903	0.76271397	1216132	45752	1170380	0.91248948	0.91298678	752846	76022	676824	0.5276874	0.86844428
3	1586488	133469	1453019	930173	64465	865708	0.5957995	1.05572352	1833201	105991	1727210	1.18870435	1.18935218	915816	130379	785437	0.54055522	0.88962156
4	1905778	225676	1680102	668353	66974	601379	0.35794196	0.6342532	1397034	96350	1300684	0.77416966	0.77459158	1002412	175077	827335	0.49243141	0.81042155
5	1661849	157511	1504338	1282387	66023	1216364	0.80857095	1.43274266	1810548	116158	1694390	1.12633597	1.12694981	1166564	100729	1065835	0.70850766	1.16603017
6	1642527	143423	1499104	965277	61019	904258	0.60319898	1.06883497	1571466	87027	1484439	0.99021749	0.99075715	1105884	124811	981073	0.65443959	1.07704735
7	1457309	125519	1331790	807471	64573	742898	0.55781918	0.98842449	1364186	54654	1309532	0.98328715	0.98382304	851648	103583	748065	0.56169892	0.92441891
8	1291597	92024	1199573	663909	69639	594270	0.49540128	0.87782346	1258120	54585	1203535	1.00330284	1.00384963	1052859	78698	974161	0.8120898	1.33650101
9	1116133	75038	1041095	734766	67527	667239	0.64090117	1.13564115	1363250	60157	1303093	1.25165619	1.25233833	824032	78802	745230	0.71581364	1.17805401
10	1319575	77327	1242248	902691	73393	829298	0.66757845	1.18291182	1416615	48278	1368337	1.10150067	1.10210097	1033478	79230	954248	0.76816223	1.26420699

Fig 4D

TUNEL DAPI

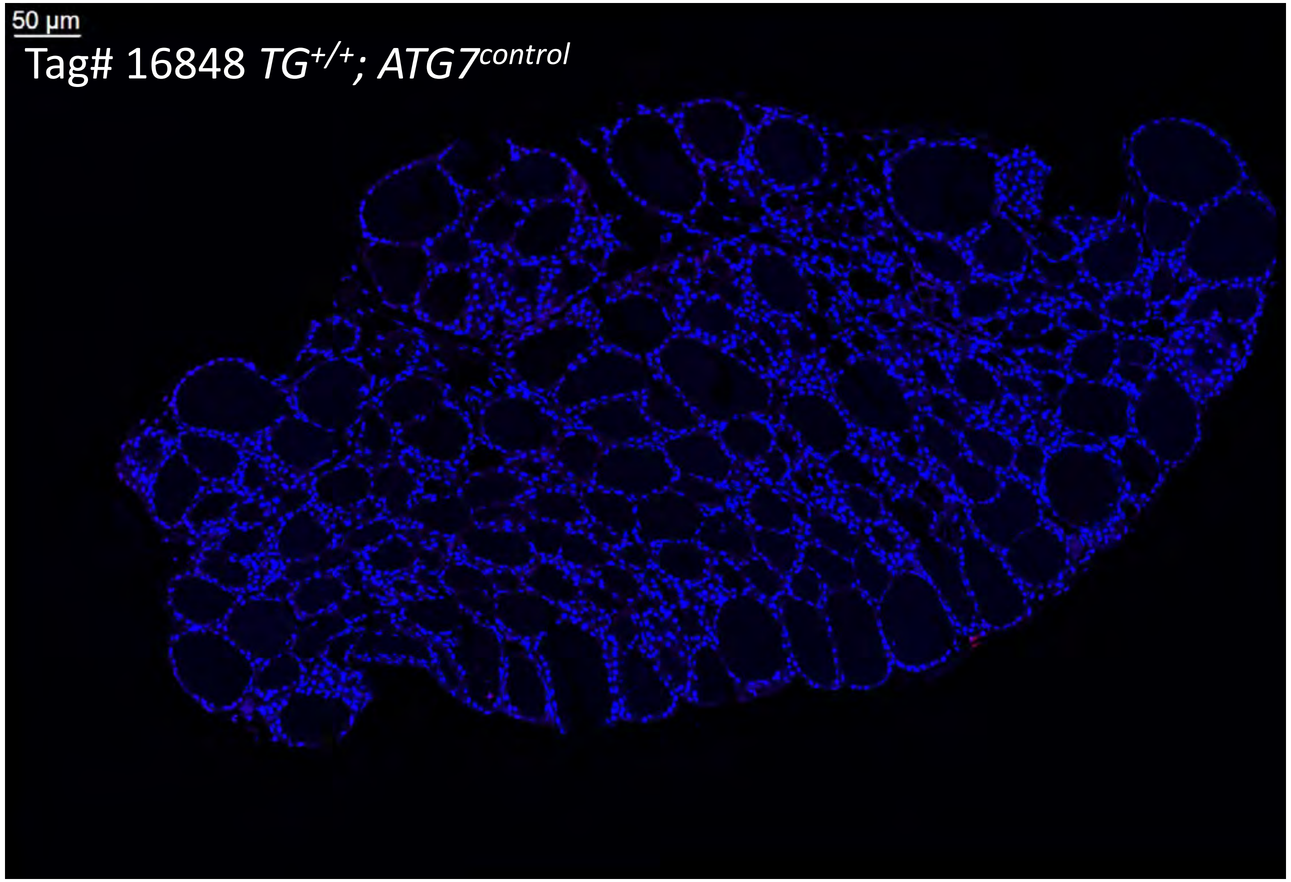

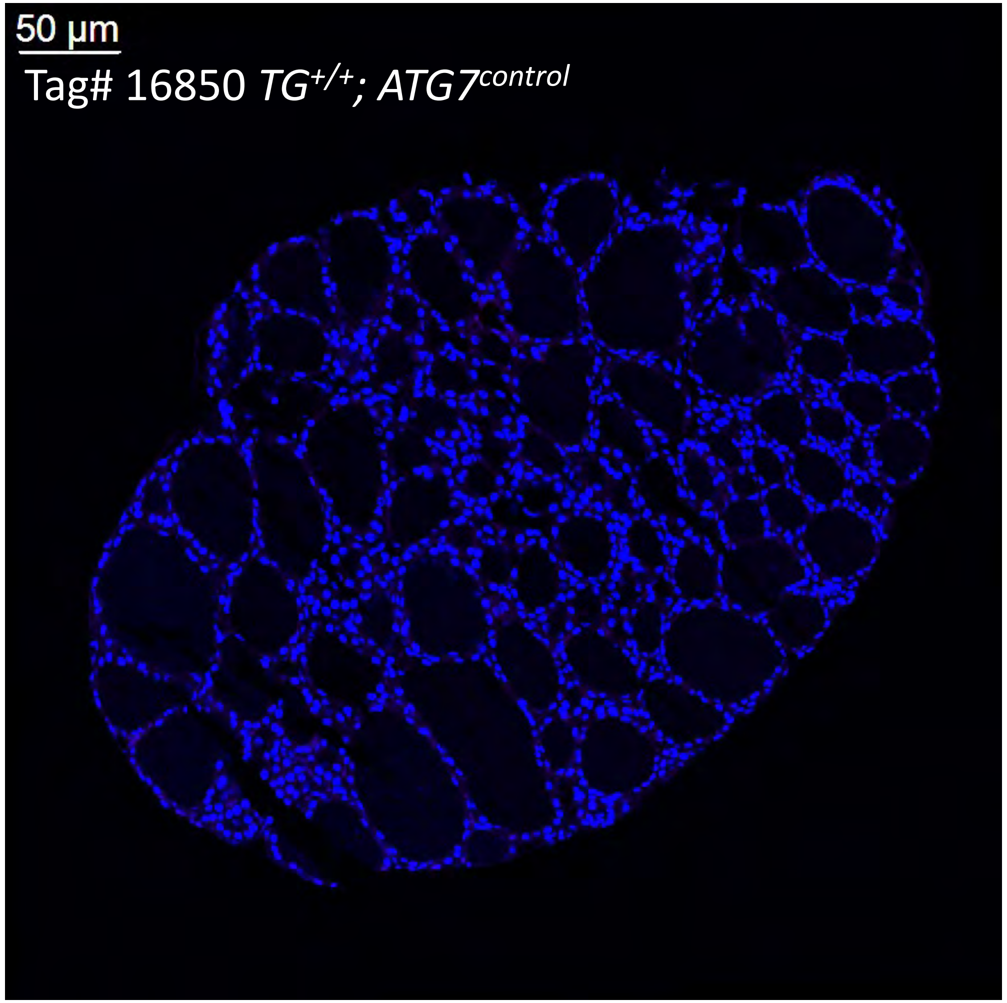

Fig 4D

TUNEL DAPI

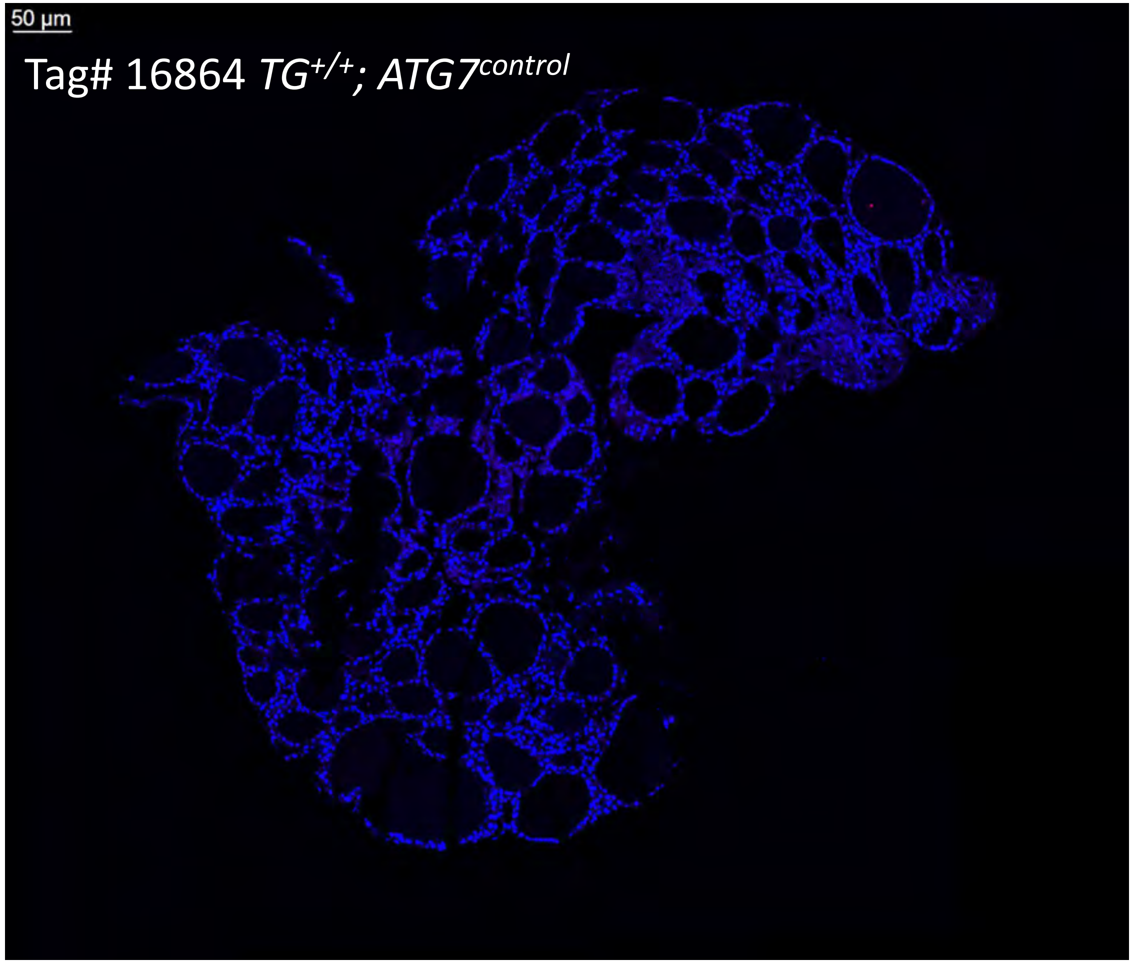

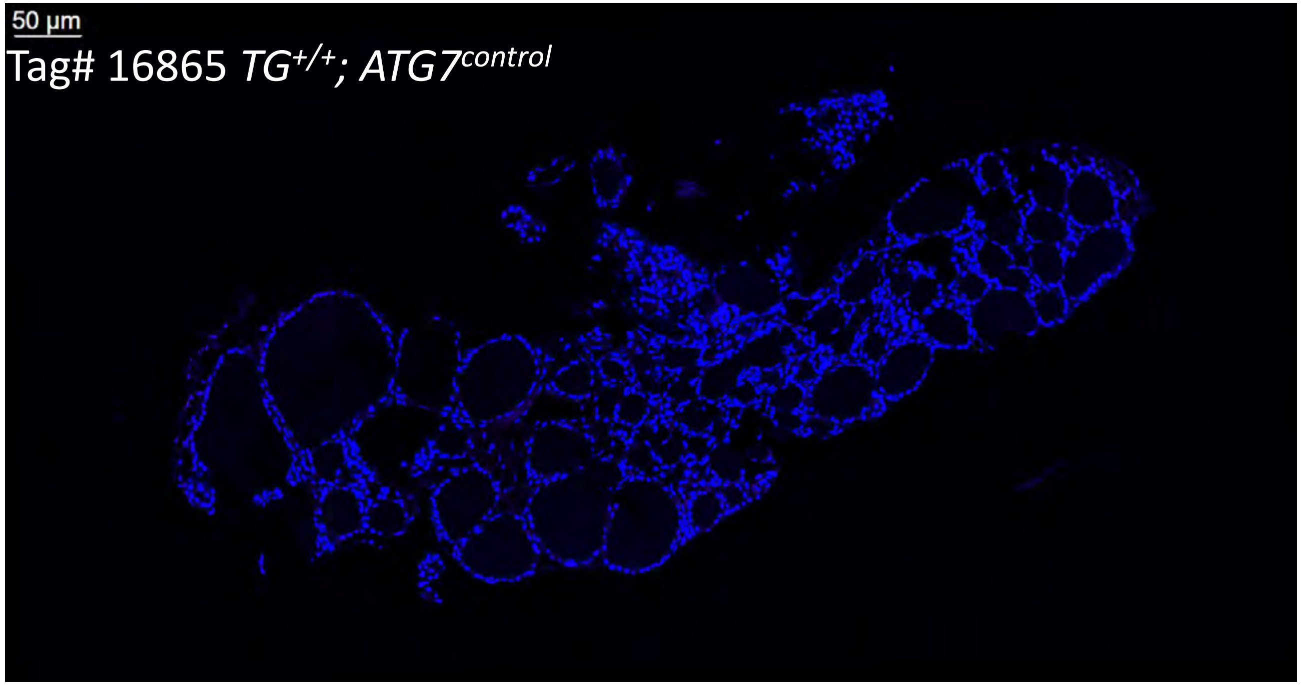

Fig 4D

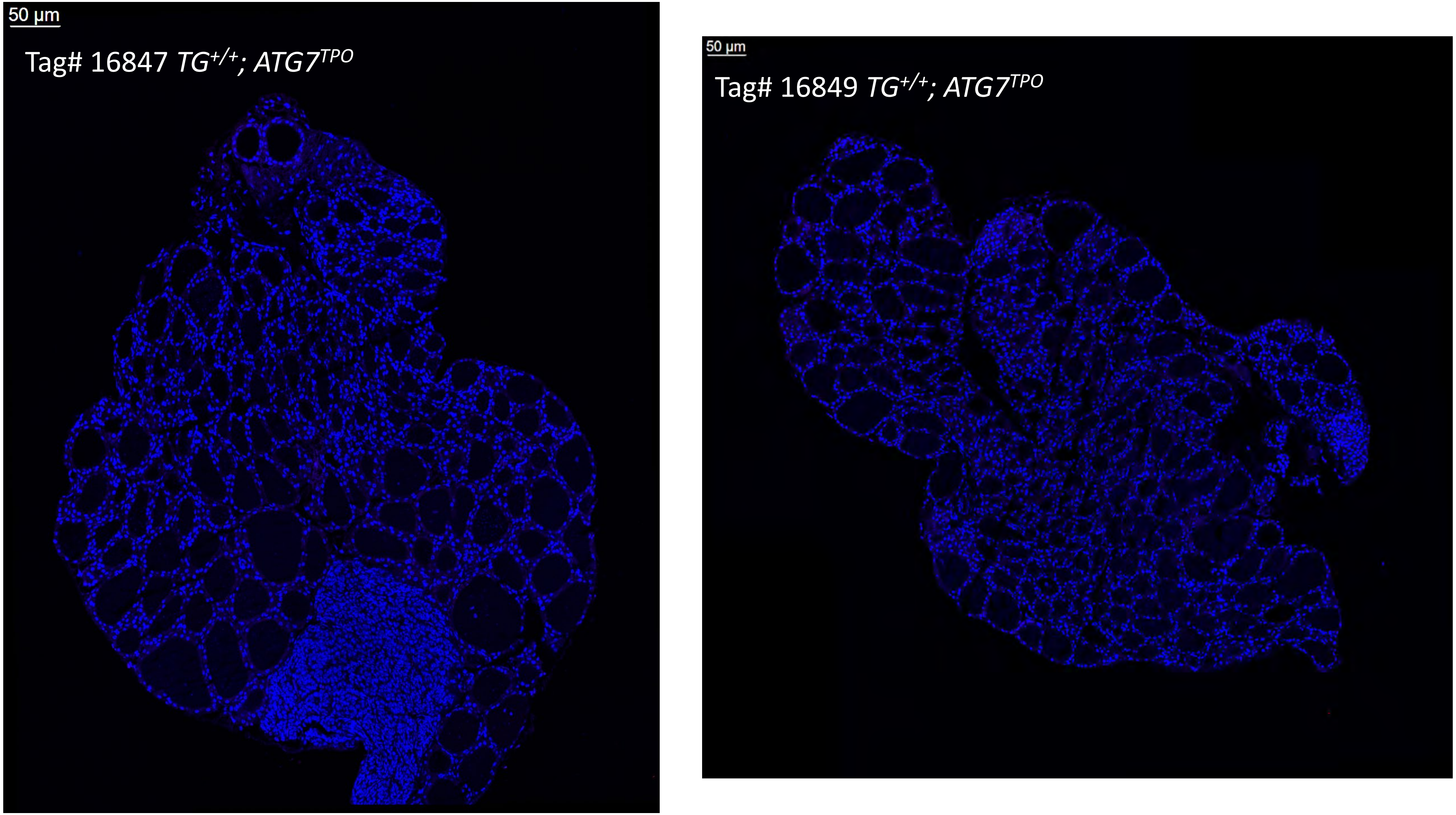
TUNEL DAPI
50 µm
Tag# 16847 TG+/+; ATG7TPO
50 µm
Tag# 16849 TG+/+; ATG7TPO

Fig 4D

TUNEL DAPI

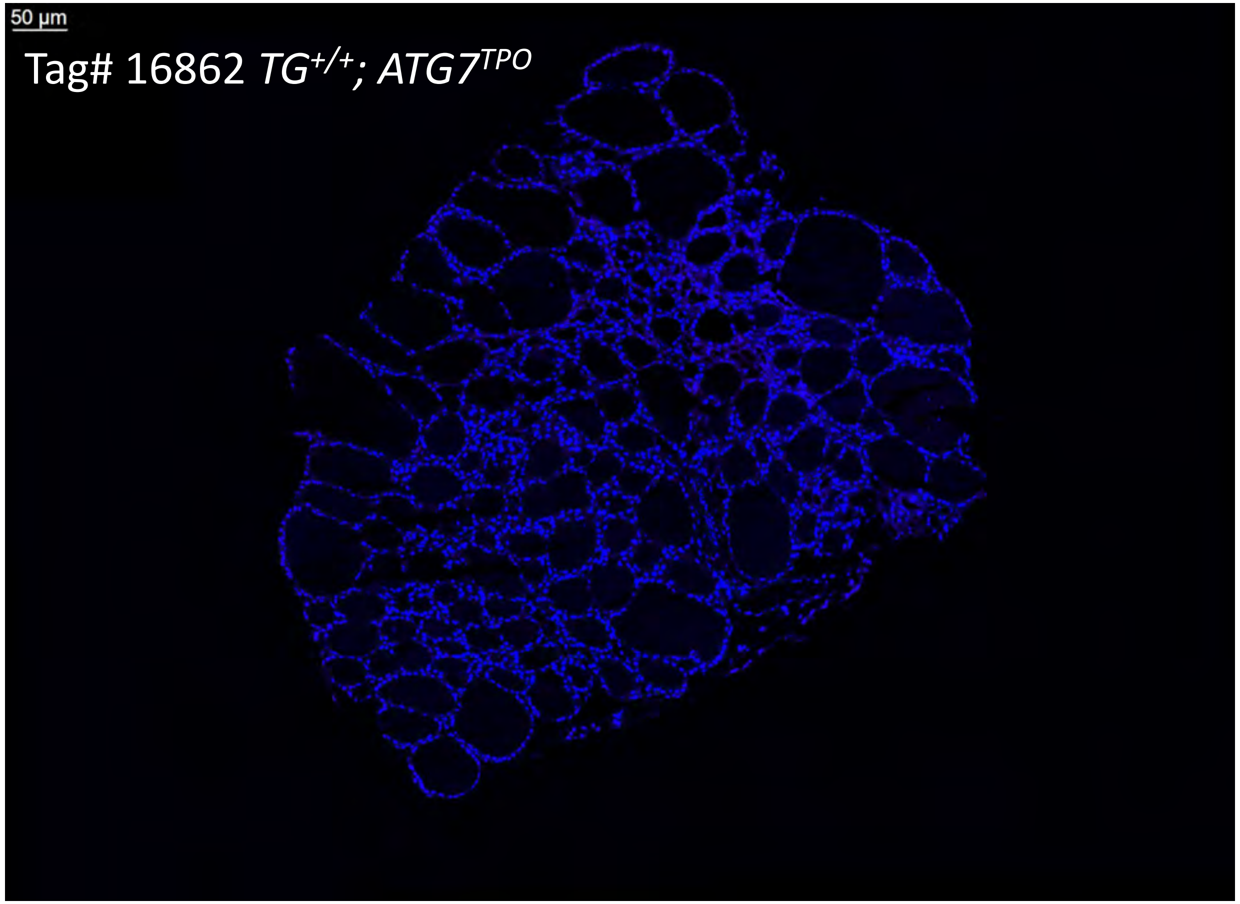

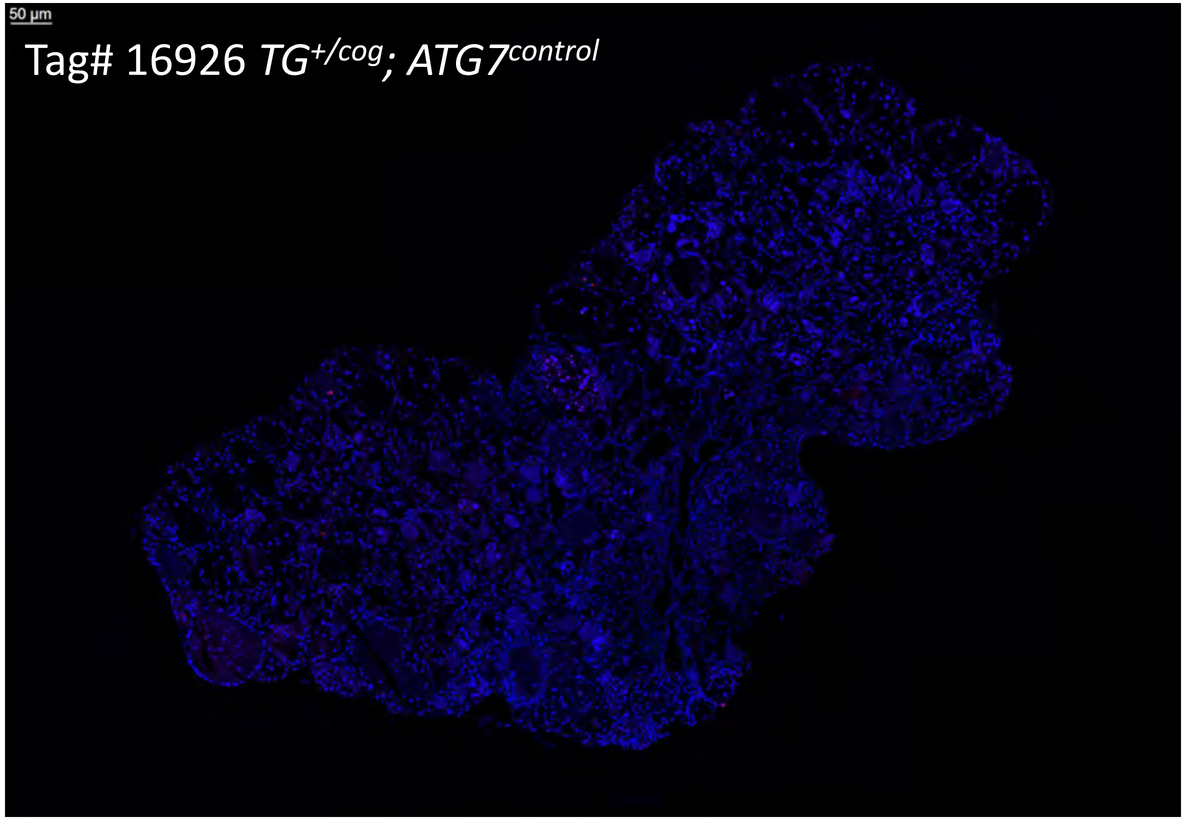

Fig 4D

TUNEL DAPI

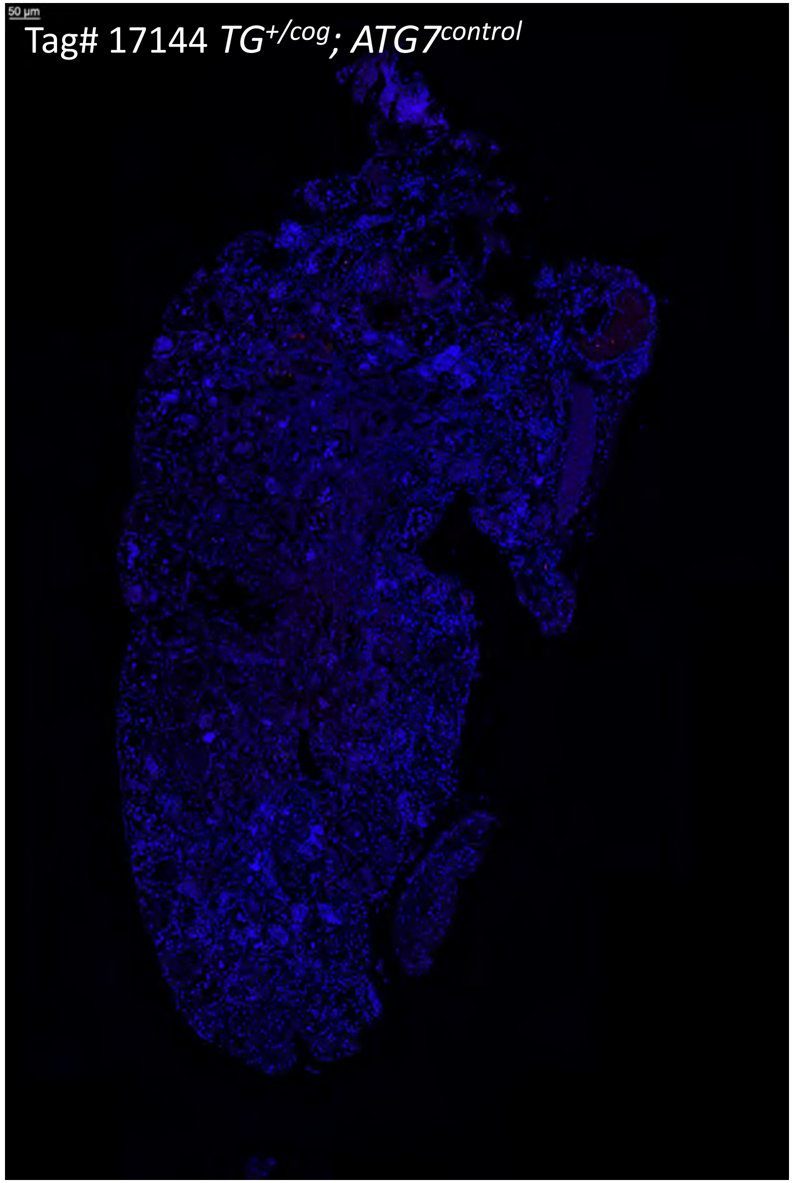

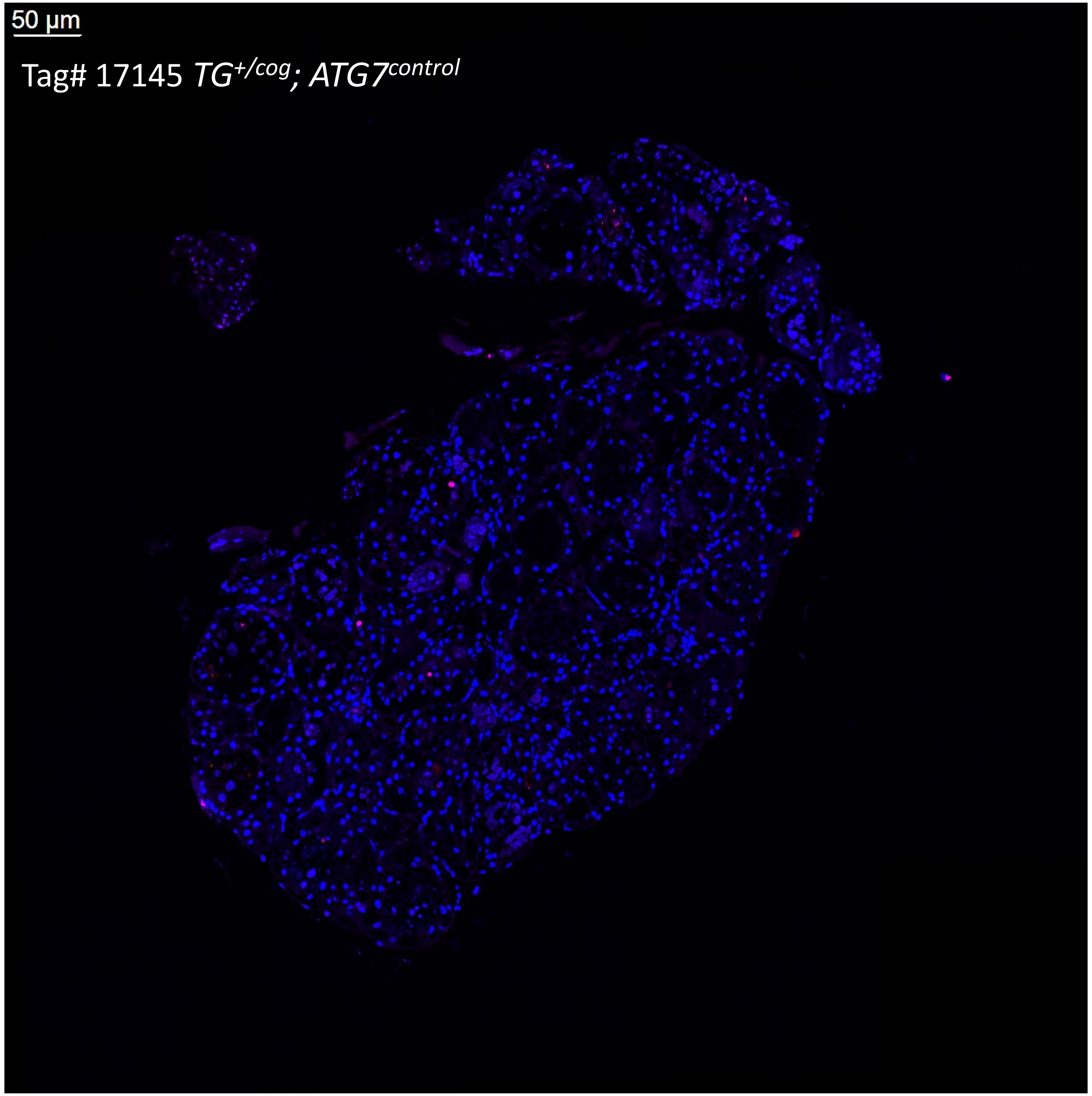

Fig 4D

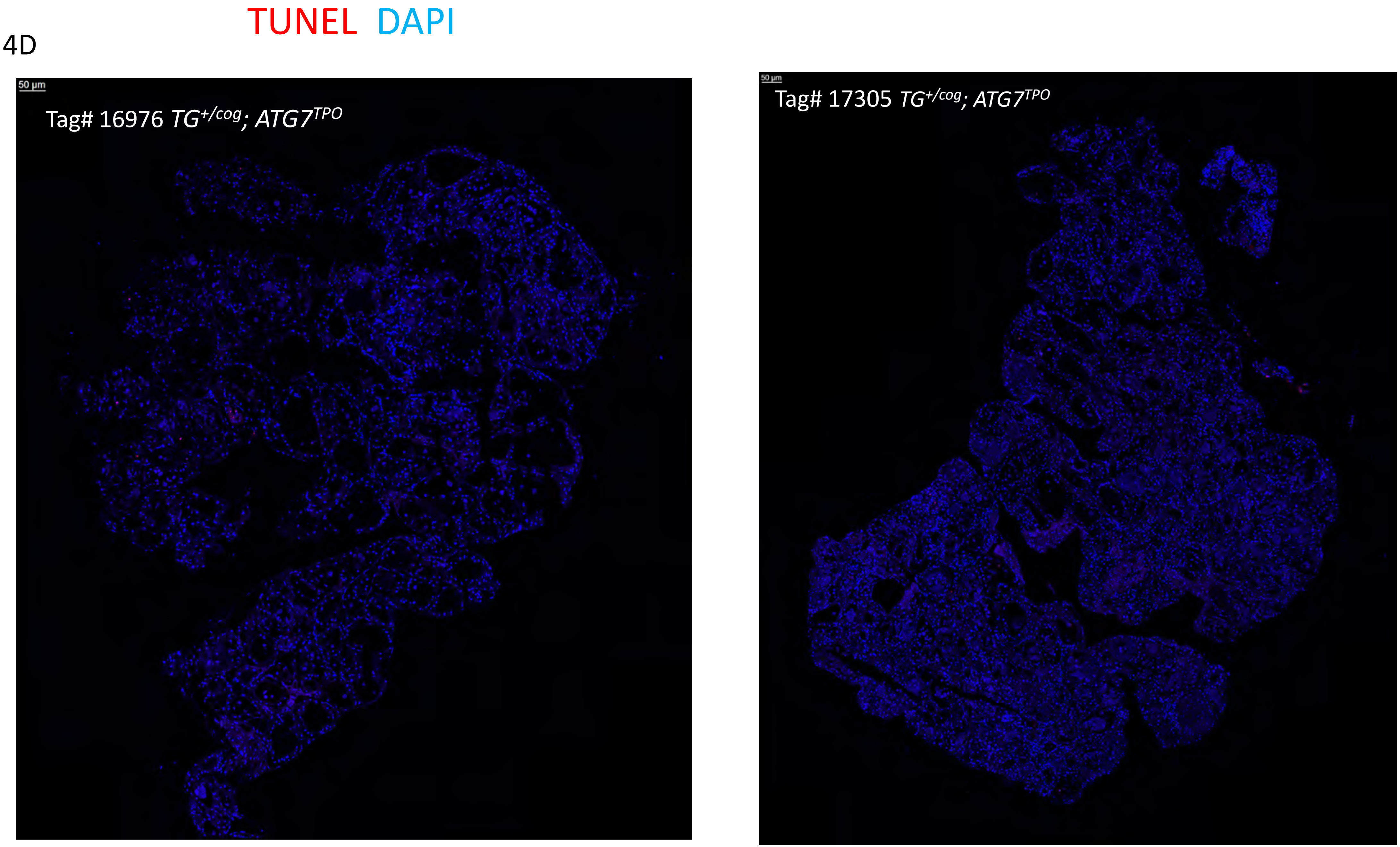
TUNEL DAPI
50 μm
Tag# 16976 TG+/cog; ATG7TPO
50 μm
Tag# 17305 TG+/cog; ATG7TPO

Fig 4D

TUNEL DAPI

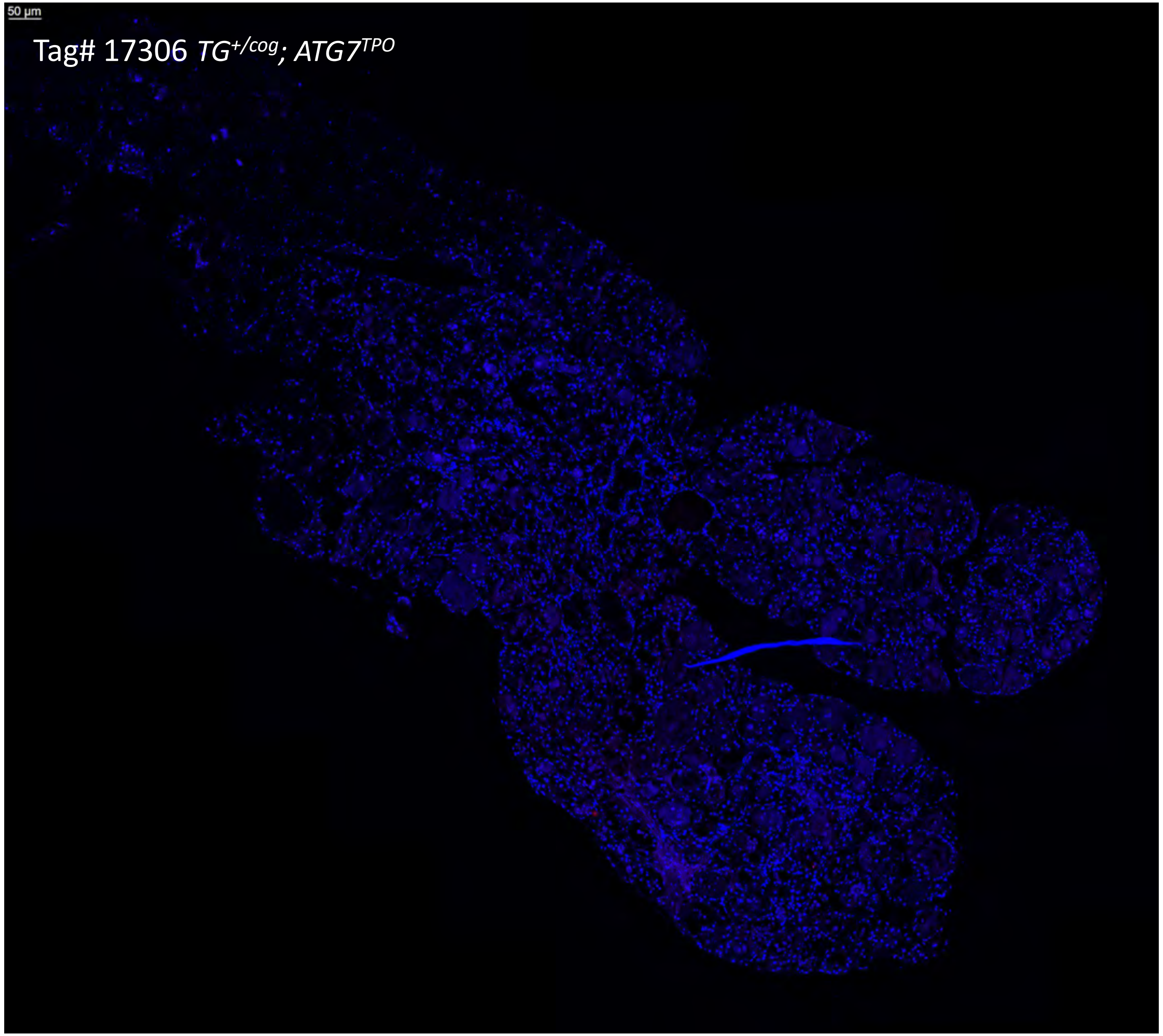

Quantitation

Tag	Genotype	Total Follicle	TUNEL positive follicle	Percentage Positive
16926	*TG+/cog; ATG7control*	141	28	19.85815603
17144	*TG+/cog; ATG7control*	147	21	14.28571429
17145	*TG+/cog; ATG7control*	51	12	23.52941176
16847	*ATG7TPO*	159	0	0
16849	*ATG7TPO*	170	0	0
16862	*ATG7TPO*	125	0	0
16976	*TG+/cog; ATG7TPO*	147	18	12.24489796
17305	*TG+/cog; ATG7TPO*	176	24	13.63636364
17306	*TG+/cog; ATG7TPO*	272	27	9.926470588
16848	*ATG7control*	159	3	1.886792453
16850	*ATG7control*	73	0	0
16864	*ATG7control*	108	2	1.851851852
16865	*ATG7control*	55	0	0

Fig. 5A
Left panel

Lanes in the figure are numbered here.
Lane 1-5: $TG^{+/cog}$;$Hrd1^{control}$
Lane 6-10: $TG^{+/cog}$; $Hrd1^{TPO}$

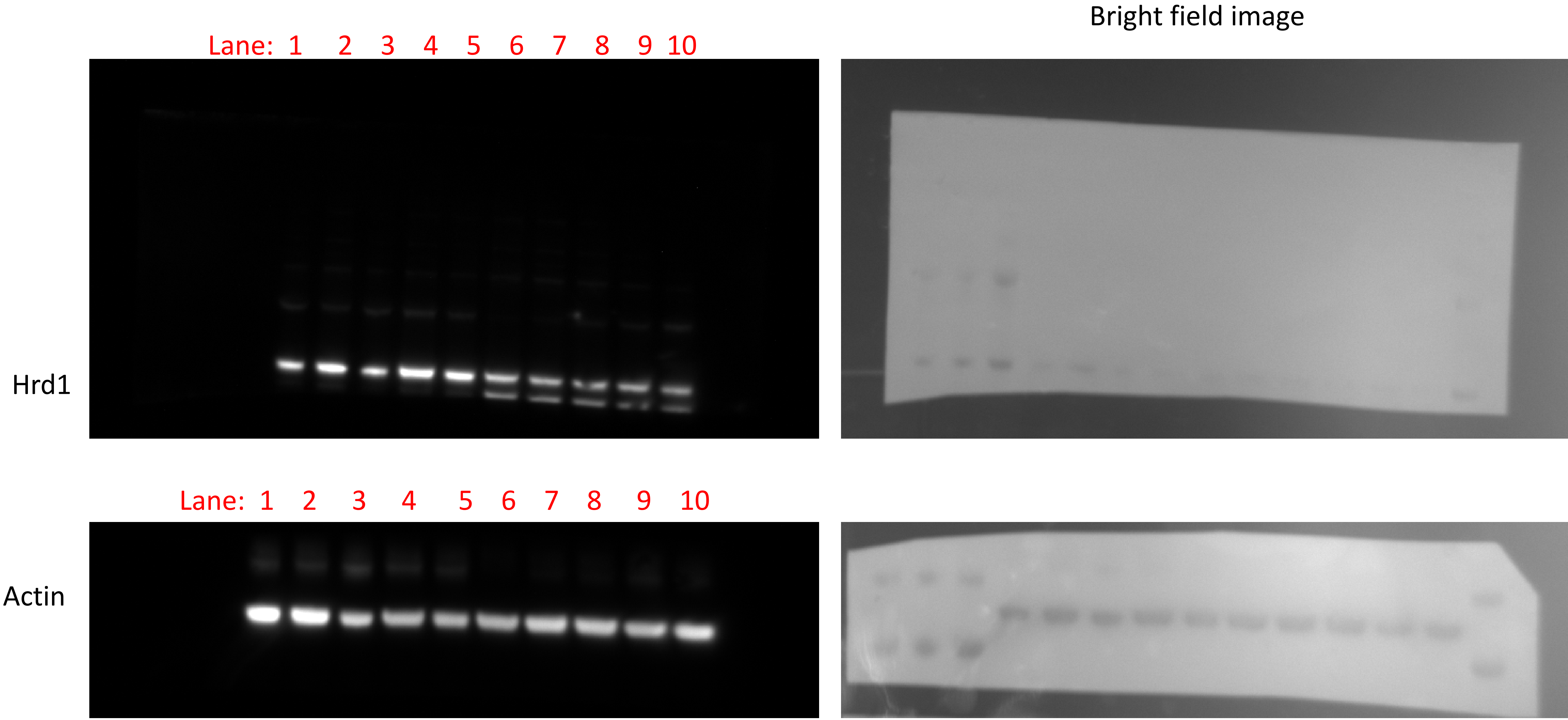

Fig. 5A
Right panel

Gel 1 is shown in the figure.

Lane 1-4: $TG^{+/cog}$;$Hrd1^{control}$
Lane 5-8: $TG^{+/cog}$; $Hrd1^{TPO}$

Gel 2:

Lane 1-2: $TG^{+/cog}$;$Hrd1^{control}$
Lane 3-4: $TG^{+/cog}$; $Hrd1^{TPO}$

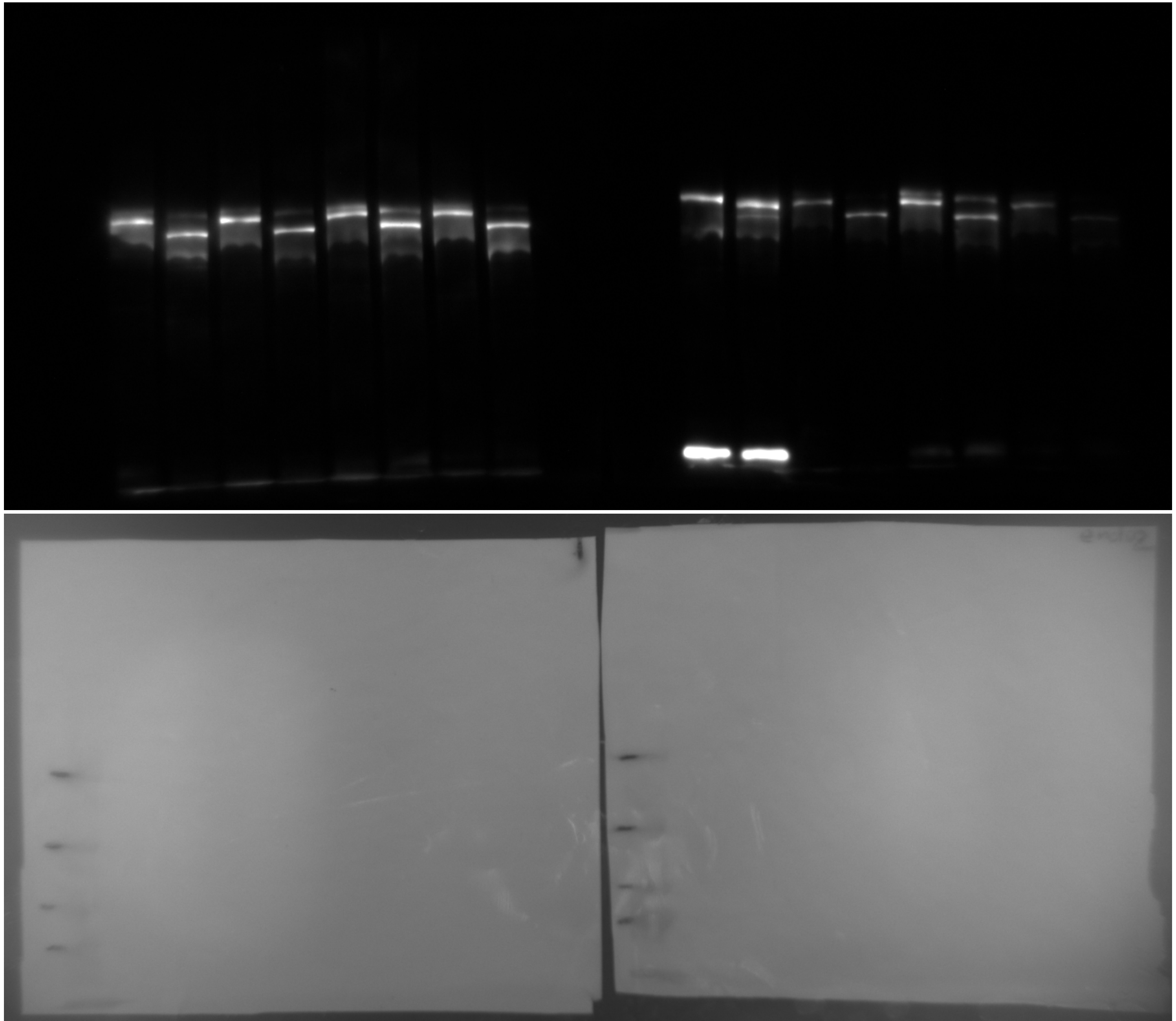

Fig. 5B

Lanes in the figure are numbered here.

Lane 1-5: *TG*$^{+/cog}$;*Hrd1*^{control}

Lane 6-10: *TG*$^{+/cog}$; *Hrd1*^{TPO}

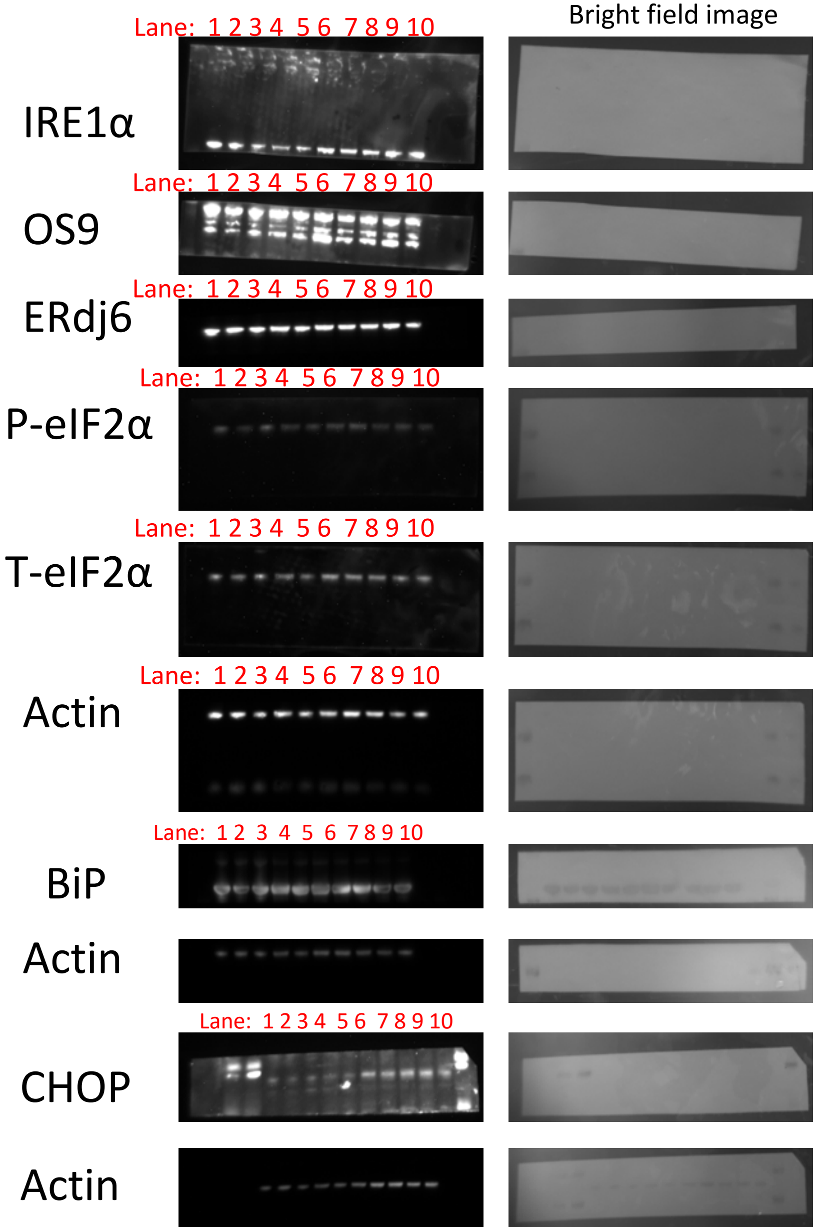

IRE1a	lane	IRE1a	background	IRE1a-background	IRE1a / Actin	Mean of control	Fold change
	1	34261627	8764624	25497003	1.248929126	0.960790721	1.299897155
	2	31483289	9823376	21659913	1.049581845		1.092414634
	3	26825454	11501456	15323998	1.121299215		1.167058746
	4	25104976	12028319	13076657	0.608633108		0.633471051
	5	24212032	12736688	11475344	0.775510314		0.807158414
	6	33109698	12170944	20938754	1.039920318		1.082358826
	7	30302666	13316416	16986250	0.714895761		0.744070218
	8	31841179	15032416	16808763	0.872940882		0.908565062
	9	36156313	17234896	18921417	1.488202942		1.548935589
	10	37966979	11314352	26652627	1.788549886		1.861539507
OS9	lane	OS9	background	OS9-background	OS9 / Actin	Mean of control	Fold change
	1	46322129	19260448	27061681	1.325572327	1.406115906	0.942719104
	2	43858932	19521440	24337492	1.17933021		0.838714793
	3	47513003	26187248	21325755	1.560464334		1.10976935
	4	55169444	30132784	25036660	1.165293254		0.828732005
	5	54283633	27649952	26633681	1.799919403		1.280064749
	6	79995668	22676032	57319636	2.846771784		2.024564101
	7	55621012	18205519	37415493	1.574695849		1.119890503
	8	62474557	18347888	44126669	2.29166021		1.629780447
	9	62878315	17527968	45350347	3.566885072		2.536693496
	10	49484231	14669808	34814423	2.33625497		1.661495301
ERdj6	lane	ERdj6	background	ERdj6-background	ERdj6 / Actin	Mean of control	Fold change
	1	32158425	1891440	30266985	1.482578918	1.666011087	0.88989739
	2	33177494	2468160	30709334	1.488092747		0.893206989
	3	30203068	2624032	27579036	2.018034158		1.211296956
	4	35709314	2374000	33335314	1.55154148		0.931291209
	5	28484767	2000704	26484063	1.789808133		1.074307456
	6	34128095	1854736	32273359	1.602851905		0.962089579
	7	30239081	1855424	28383657	1.194575382		0.71702727
	8	31204518	1850960	29353558	1.524438223		0.915022856
	9	31250508	2009056	29241452	2.299891964		1.380478186
	10	23178786	1389056	21789730	1.462220557		0.877677567
Actin	lane	Actin	background	Actin-background			
	1	21091668	676576	20415092			
	2	21156227	519520	20636707			
	3	14126576	460288	13666288			
	4	22203831	718544	21485287			
	5	15493184	696032	14797152			
	6	20909968	775008	20134960			
	7	24554905	794448	23760457			
	8	19766528	511200	19255328			
	9	13212016	497744	12714272			
	10	15392064	490256	14901808			
P-eIF2a	lane	P-eIF2a	background	P-eIF2a-background	P-eIF2a / T-eIF2a	Mean of control	Fold change
	1	16240191	6966557	9273634	1.065396244	0.935877897	1.138392355
	2	12526506	6608621	5917885	0.724299268		0.773924964
	3	17932618	7406079	10526539	1.189996649		1.271529815
	4	15544038	7888896	7655142	0.807232032		0.862539904
	5	14601424	8528776	6072648	0.892465293		0.953612961
	6	19046705	9272960	9773745	0.774537405		0.82760519
	7	20475779	8856641	11619138	1.163839256		1.243580235
	8	15447581	9471958	5975623	0.578480765		0.618115639
	9	17712102	9501071	8211031	0.892939745		0.954119921
	10	13244059	6511039	6733020	0.744793952		0.795823851
T-eIF2a	lane	T-eIF2a	background	T-eIF2a-background			
	1	10211631	1507232	8704399			
	2	9977968	1807471	8170497			
	3	10714560	1868704	8845856			
	4	12419439	2936240	9483199			
	5	9513088	2708736	6804352			
	6	15987200	3368384	12618816			
	7	14914880	4931424	9983456			
	8	13902480	3572624	10329856			
	9	12111520	2916016	9195504			
	10	13065615	4025504	9040111			

Quantification:

	Lane	BiP	Actin	BiP/Actin	Mean of control	Fold change
BiP	1	18958672	15794117	1.200363	1.122710192	1.069165403
	2	14039584	18676917	0.751708		0.669547489
	3	19895552	16362039	1.215958		1.08305598
	4	21985024	19519437	1.126314		1.003210322
	5	21773024	16504620	1.319208		1.175020806
	6	18882160	24421755	0.77317		0.688663615
	7	23426176	24409772	0.959705		0.854810826
	8	24348480	20160956	1.207705		1.075704704
	9	13351792	17851056	0.747955		0.666205142
	10	12728032	19491051	0.653019		0.581645451

CHOP	Lane	CHOP	background	CHOP-background	CHOP/Actin	Mean of control	Fold change
	1	3636263	1702199	1934064	0.696716965	0.535031393	1.302198
	2	1948898	1590399	358499	0.088174457		0.164802
	3	3353406	994638	2358768	0.905335364		1.692116
	4	6616295	5233558	1382737	0.355709568		0.664839
	5	6771148	4411762	2359386	0.629220609		1.176044
	6	19693353	4767339	14926014	2.698231816		5.043128
	7	22396654	5617383	16779271	2.064163458		3.858023
	8	27262694	5149042	22113652	2.463756342		4.604882
	9	29712672	8959908	20752764	2.884187032		5.390687
	10	25538937	13585339	11953598	1.62836036		3.043486
Actin	Lane	Actin	background	Actin-background			
	1	2877696	101728	2775968			
	2	4180320	114528	4065792			
	3	2720640	115232	2605408			
	4	4011040	123776	3887264			
	5	3892336	142640	3749696			
	6	5671536	139760	5531776			
	7	8287424	158576	8128848			
	8	9183184	207600	8975584			
	9	7397184	201824	7195360			
	10	7492864	151984	7340880			

Fig. 5C
left 3 panels

Mouse tag#	Genotype	TSH (mU/L)	T_4 (µg/dL)	T_3 (ng/dL)
17686	$TG^{+/cog}$; $Hrd1^{control}$	550	2.53	87.4
17731	$TG^{+/cog}$; $Hrd1^{control}$	534	1.38	86.4
17633	$TG^{+/cog}$; $Hrd1^{control}$	411	2.50	74.4
17742	$TG^{+/cog}$; $Hrd1^{control}$	826	2.22	64.1
17740	$TG^{+/cog}$; $Hrd1^{control}$	468	2.22	75.4
17739	$TG^{+/cog}$; $Hrd1^{control}$	1117	1.42	52.8
17627	$TG^{+/cog}$; $Hrd1^{control}$	69	2.76	68.6
17690	$TG^{+/cog}$; $Hrd1^{control}$	294	3.23	61.8
17684	$TG^{+/cog}$; $Hrd1^{control}$	1043	3.43	70.0
17734	$TG^{+/cog}$; $Hrd1^{control}$	464	4.15	78.7
17759	$TG^{+/cog}$; $Hrd1^{control}$	854	3.21	83.5
17826	$TG^{+/cog}$; $Hrd1^{control}$	250	1.56	58.2
17824	$TG^{+/cog}$; $Hrd1^{control}$	177	3.05	51.7
17814	$TG^{+/cog}$; $Hrd1^{control}$	111	2.75	79.7
17908	$TG^{+/cog}$; $Hrd1^{control}$	241	1.89	57.7
17920	$TG^{+/cog}$; $Hrd1^{control}$	1416	1.82	47.4
17624	$TG^{+/cog}$; $Hrd1^{TPO}$	4854	1.50	57.0
17675	$TG^{+/cog}$; $Hrd1^{TPO}$	<10	1.44	87.4
17819	$TG^{+/cog}$; $Hrd1^{TPO}$	1404	1.54	62.0
17925	$TG^{+/cog}$; $Hrd1^{TPO}$	1931	2.23	60.9
17631	$TG^{+/cog}$; $Hrd1^{TPO}$	3116	2.04	70.5
17682	$TG^{+/cog}$; $Hrd1^{TPO}$	15	1.40	54.1
17736	$TG^{+/cog}$; $Hrd1^{TPO}$	2455	1.97	52.6
17738	$TG^{+/cog}$; $Hrd1^{TPO}$	760	2.10	54.1
17728	$TG^{+/cog}$; $Hrd1^{TPO}$	3279	2.12	66.6
17733	$TG^{+/cog}$; $Hrd1^{TPO}$	409	2.03	51.2
17772	$TG^{+/cog}$; $Hrd1^{TPO}$	1002	1.65	49.0
17760	$TG^{+/cog}$; $Hrd1^{TPO}$	607	2.65	66.8
17745	$TG^{+/cog}$; $Hrd1^{TPO}$	224	0.49	55.0
17822	$TG^{+/cog}$; $Hrd1^{TPO}$	609	2.57	89.4
17816	$TG^{+/cog}$; $Hrd1^{TPO}$	51	2.21	44.2
17859	$TG^{+/cog}$; $Hrd1^{TPO}$	1635	3.19	63.0
17692	$TG^{+/cog}$; $Hrd1^{TPO}$	391	1.43	49.3

Fig. 5C
Right panel

$TG^{+/cog}$;$Hrd1^{control}$

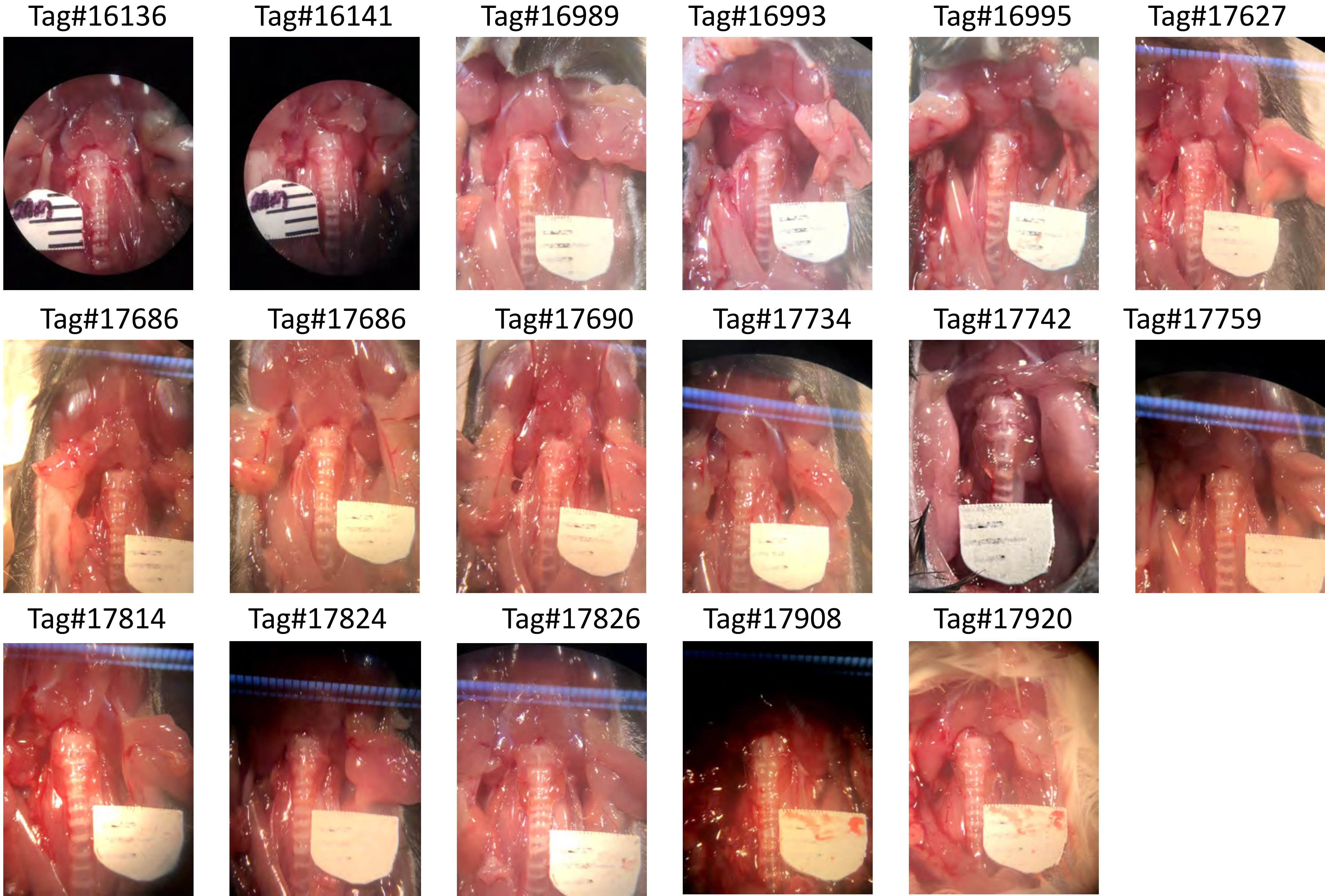

Fig. 5C
Right panel

TG$^{+/cog}$; *Hrd1*^{TPO}

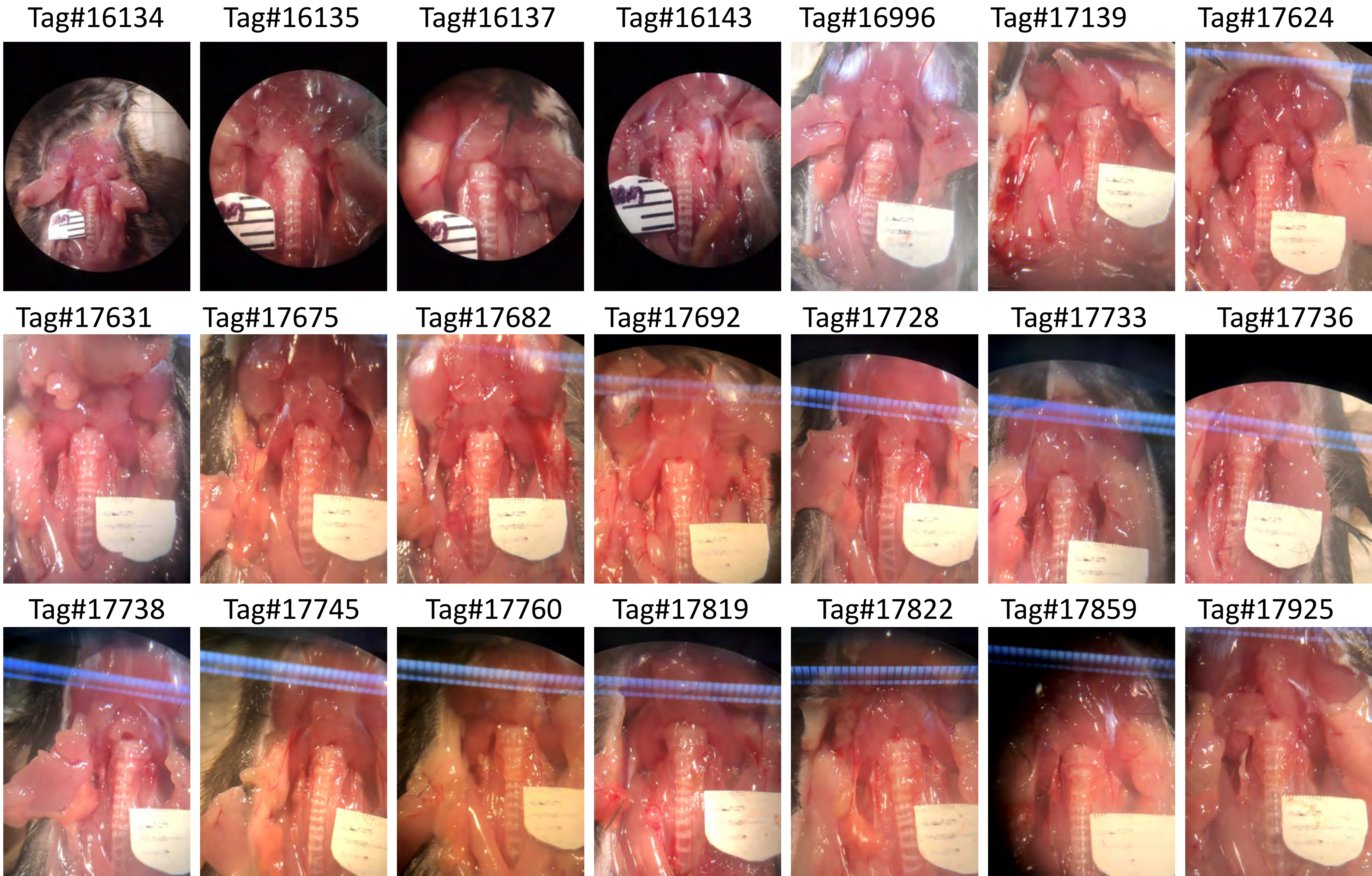

Fig. 5C
Right panel

Quantification:

#tag	*Genotype*	Body weight (g)	area_left lobe (mm^2)	area_right lobe (mm2)	total area (mm2)	thyroid area / body weight
16136	$TG^{+/cog}; Hrd1^{control}$	32.8	2.238	1.462	3.7	0.112804878
16141	$TG^{+/cog}; Hrd1^{control}$	21.9	1.309	0.622	1.931	0.088173516
16989	$TG^{+/cog}; Hrd1^{control}$	28.3	2.284	3.313	5.597	0.197773852
16993	$TG^{+/cog}; Hrd1^{control}$	24.3	2.098	3.532	5.63	0.231687243
16995	$TG^{+/cog}; Hrd1^{control}$	22.9	1.052	1.893	2.945	0.12860262
17627	$TG^{+/cog}; Hrd1^{control}$	22.2	1.336	1.8	3.136	0.141261261
17684	$TG^{+/cog}; Hrd1^{control}$	19.6	1.078	2.416	3.494	0.178265306
17686	$TG^{+/cog}; Hrd1^{control}$	32.5	1.834	2.098	3.932	0.120984615
17690	$TG^{+/cog}; Hrd1^{control}$	25.8	1.788	2.438	4.226	0.16379845
17734	$TG^{+/cog}; Hrd1^{control}$	22.3	1.912	2.491	4.403	0.197443946
17742	$TG^{+/cog}; Hrd1^{control}$	28.9	2.747	2.353	5.1	0.176470588
17759	$TG^{+/cog}; Hrd1^{control}$	25.3	1.159	1.656	2.815	0.111264822
17814	$TG^{+/cog}; Hrd1^{control}$	23	2.074	1.73	3.804	0.165391304
17824	$TG^{+/cog}; Hrd1^{control}$	18.7	0.841	2.128	2.969	0.158770053
17826	$TG^{+/cog}; Hrd1^{control}$	23.7	1.459	1.787	3.246	0.136962025
17908	$TG^{+/cog}; Hrd1^{control}$	24.7	1.819	2.21	4.029	0.163117409
17920	$TG^{+/cog}; Hrd1^{control}$	19.6	1.285	1.931	3.216	0.164081633
16134	$TG^{+/cog}; Hrd1^{TPO}$	25.2	2.112	2.093	4.205	0.166865079
16135	$TG^{+/cog}; Hrd1^{TPO}$	25.5	1.512	1.145	2.657	0.104196078
16137	$TG^{+/cog}; Hrd1^{TPO}$	29.6	1.472	1.924	3.396	0.11472973
16143	$TG^{+/cog}; Hrd1^{TPO}$	21.4	0.866	1.17	2.036	0.095140187
16996	$TG^{+/cog}; Hrd1^{TPO}$	16.9	0.565	2.003	2.568	0.151952663
17139	$TG^{+/cog}; Hrd1^{TPO}$	21.1	1.535	1.768	3.303	0.156540284
17624	$TG^{+/cog}; Hrd1^{TPO}$	25.8	1.665	1.445	3.11	0.120542636
17631	$TG^{+/cog}; Hrd1^{TPO}$	20.3	1.556	1.602	3.158	0.155566502
17675	$TG^{+/cog}; Hrd1^{TPO}$	25.9	1.101	2.541	3.642	0.140617761
17682	$TG^{+/cog}; Hrd1^{TPO}$	22.5	1.113	1.99	3.103	0.137911111
17692	$TG^{+/cog}; Hrd1^{TPO}$	22.4	1.23	1.372	2.602	0.116160714
17728	$TG^{+/cog}; Hrd1^{TPO}$	22.9	0.887	2.065	2.952	0.128908297
17733	$TG^{+/cog}; Hrd1^{TPO}$	23.8	0.639	1.947	2.586	0.108655462
17736	$TG^{+/cog}; Hrd1^{TPO}$	21.2	1.357	1.283	2.64	0.124528302
17738	$TG^{+/cog}; Hrd1^{TPO}$	21.4	1.06	1.484	2.544	0.118878505
17745	$TG^{+/cog}; Hrd1^{TPO}$	17.8	2.153	0.949	3.102	0.174269663
17760	$TG^{+/cog}; Hrd1^{TPO}$	25.3	2.447	1.397	3.844	0.151936759
17819	$TG^{+/cog}; Hrd1^{TPO}$	23.2	1.73	2.536	4.266	0.18387931
17822	$TG^{+/cog}; Hrd1^{TPO}$	23.1	0.719	2.806	3.525	0.152597403
17859	$TG^{+/cog}; Hrd1^{TPO}$	23	1.298	1.701	2.999	0.130391304
17925	$TG^{+/cog}; Hrd1^{TPO}$	29	1.429	1.797	3.226	0.111241379

Fig. 5D qPCR

Ct (3 replicates):

	CHOP				Tg				18s			
	rep1	rep2	rep3	mean	rep1	rep2	rep3	mean	rep1	rep2	rep3	mean
Tag#17908	24.75826	24.50126	24.76069	24.6734	15.12649	15.11122608	15.12374	15.12048	12.82974	13.30488	12.7356	12.95674
Tag#17920	23.80491	23.74461	23.71689	23.75547	14.65382	14.62825489	14.52975	14.60394	12.93192	12.19028	12.34374	12.48865
Tag#18145	24.90672	24.89988	24.96225	24.92295	14.79037	14.89355183	14.88892	14.85761	12.36593	12.39081	13.0699	12.60888
Tag#18147	24.7681	24.84078	24.74086	24.78325	14.94026	14.89025879	14.95287	14.9278	13.49479	13.21576	12.55016	13.0869
Tag#17925	21.91592	21.85071	21.88242	21.88302	14.41802	13.97973537	14.37775	14.2585	12.34083	12.29593	12.8256	12.48745
Tag#17983	22.77965	22.55802	22.61178	22.64982	15.00291	15.14506626	15.06192	15.06996	12.9372	12.79633	13.26268	12.99874
Tag#17987	22.97211	22.90448	22.9307	22.93576	15.16098	15.29744148	15.19695	15.21846	13.41969	13.17143	13.50668	13.36593
Tag#18146	24.79588	24.71032	24.73071	24.74563	15.26162	15.22129822	15.22484	15.23592	12.77809	12.58803	12.74197	12.7027

Mean of Ct from 3 replicates:

$TG^{+/cog}$; $Hrd1^{control}$	17908	17920	18145	18147	$TG^{+/cog}$; $Hrd1^{TPO}$	17925	17983	17987	18146
CHOP	24.6734	23.75547	24.92295	24.78325		21.88302	22.64982	22.93576	24.74563
Tg	15.12048	14.60394	14.85761	14.9278		14.2585	15.06996	15.21846	15.23592
18s	12.95674	12.48865	12.60888	13.0869		12.48745	12.99874	13.36593	12.7027

ΔCt:

	17908	17920	18145	18147	mean of control	17925	17983	17987	18146
CHOP	11.71666	11.26682	12.31407	11.69634	11.74847571	9.395569	9.651082	9.56983	12.04294
Tg	2.163743	2.115296	2.248734	1.840894	2.092166503	1.771047	2.071229	1.852523	2.533221

ΔΔCt:

	17908	17920	18145	18147		17925	17983	17987	18146
CHOP	-0.03181	-0.48165	0.565597	-0.05213		-2.35291	-2.09739	-2.17865	0.294462
Tg	0.071576	0.023129	0.156567	-0.25127		-0.32112	-0.02094	-0.23964	0.441055

$2^{-\Delta\Delta Ct}$

	17908	17920	18145	18147		17925	17983	17987	18146
CHOP	1.022297	1.396341	0.675676	1.036796		5.108526	4.279356	4.527285	0.815376
Tg	0.951598	0.984096	0.897157	1.190257		1.249299	1.014618	1.180701	0.736596

Fig. 5D

Lane 1-4: *TG*$^{+/cog}$;*Hrd1*^{control}
Lane 5-8: *TG*$^{+/cog}$; *Hrd1*^{TPO}

XBP1

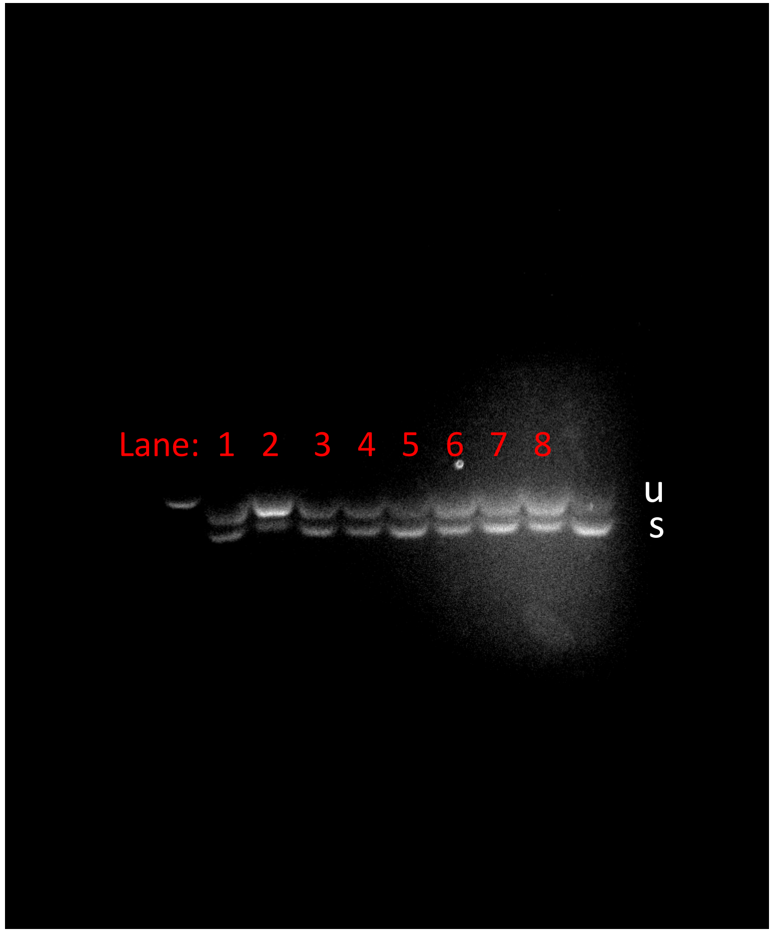

Bright field image

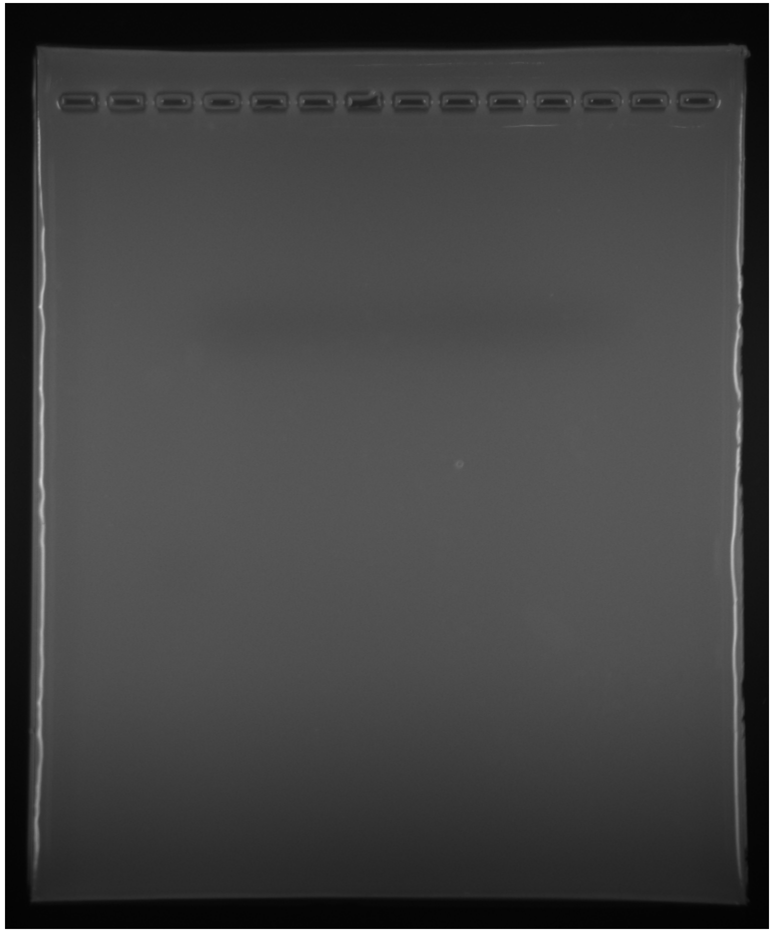

Quantification:

lane	Unspliced	Spliced	s/u	mean of control	fold change
1	9541674	5767655	0.604469928	0.638808899	0.946245317
2	23717120	6078445	0.256289339		0.401198761
3	10274800	8144983	0.792714505		1.240925897
4	10733607	9679157	0.901761822		1.411630025
5	15828092	18951611	1.19734021		1.874332391
6	8494020	7441452	0.87608129		1.371429378
7	18613488	20849142	1.120109353		1.753434173
8	29512371	19967936	0.676595452		1.059151577

Fig. 6A
Fig. 7A

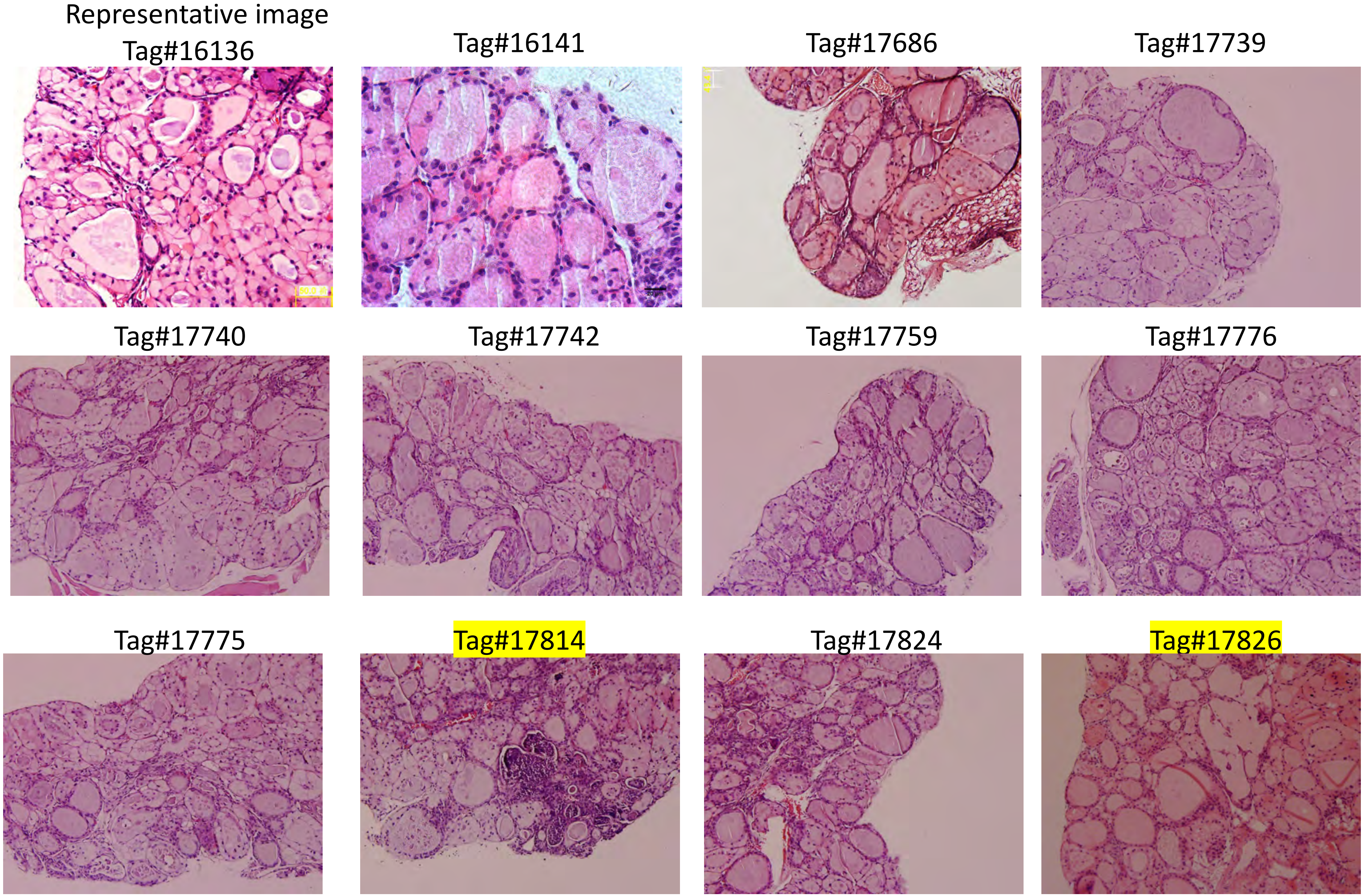

$TG^{+/cog}$;$Hrd1^{control}$

Degenerating thyroid follicles (highlighted) :

2/12 (16.7%)

(Recent degeneration is multiple condensed nuclei; at a later stage, follicular cells removed with lighter staining follicle remnant(s).

Fig. 6A
Fig. 7A

Representative image

TG$^{+/cog}$;Hrd1^{TPO}

Degenerating thyroid follicles (highlighted) :

13/20 (65%) (Recent degeneration is multiple condensed nuclei; at a later stage, follicular cells removed with lighter staining follicle remnant(s).

Tag#16134

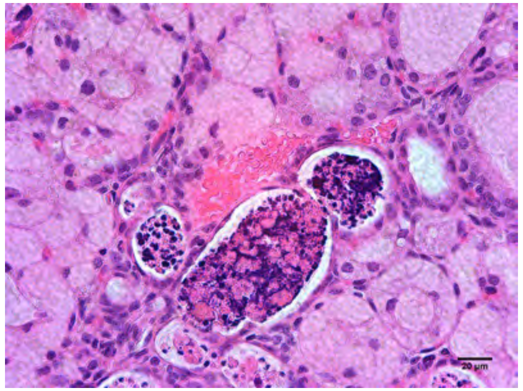

Tag#16135

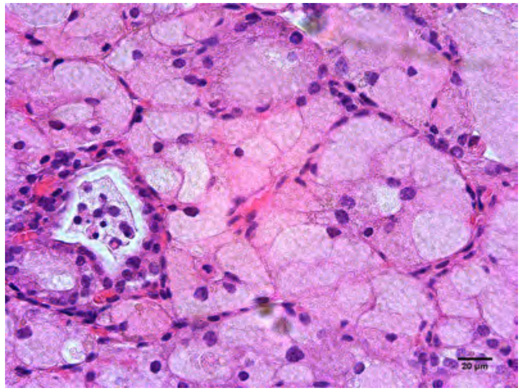

Tag#16137

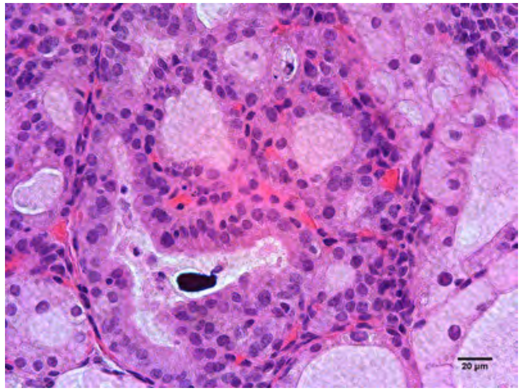

Tag#16143

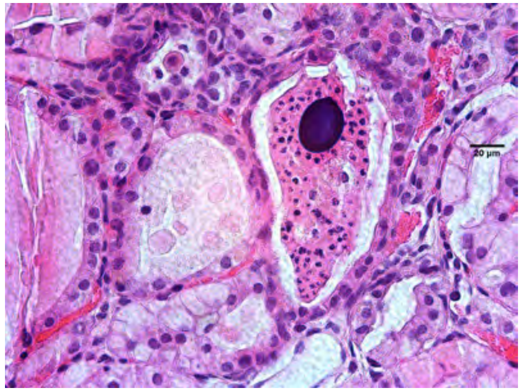

Tag#17624

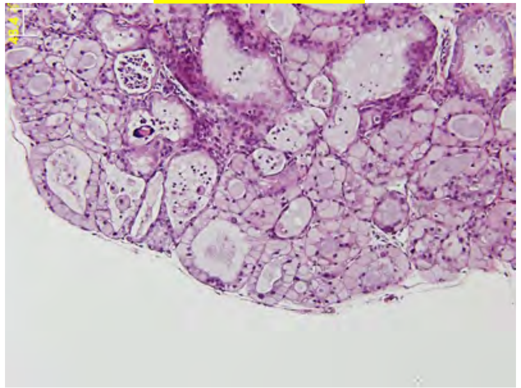

Tag#17631

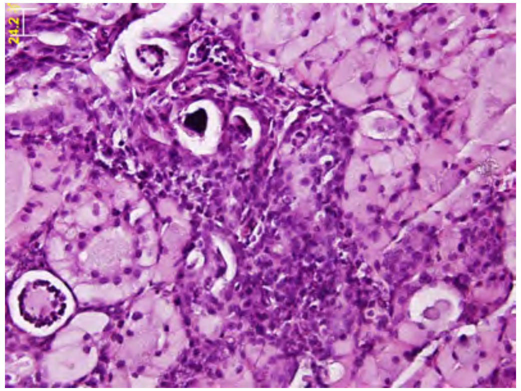

Tag#17675

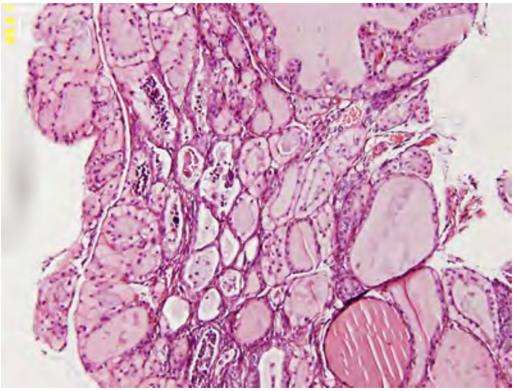

Tag#17682

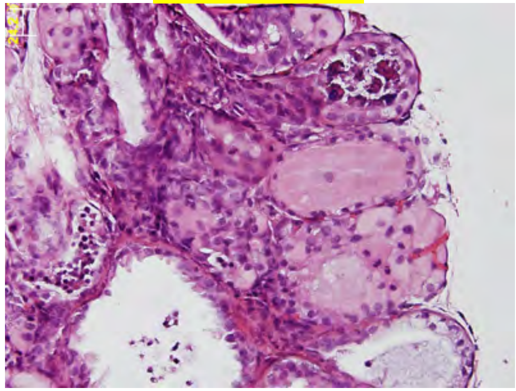

Tag#17692

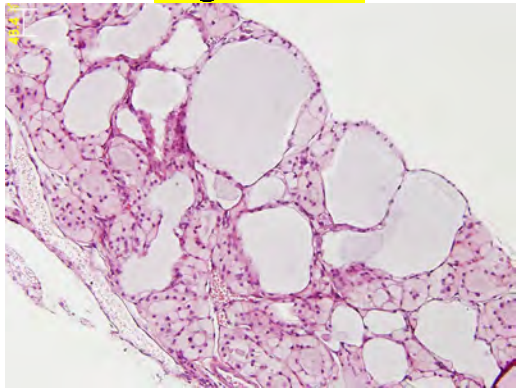

Tag#17728

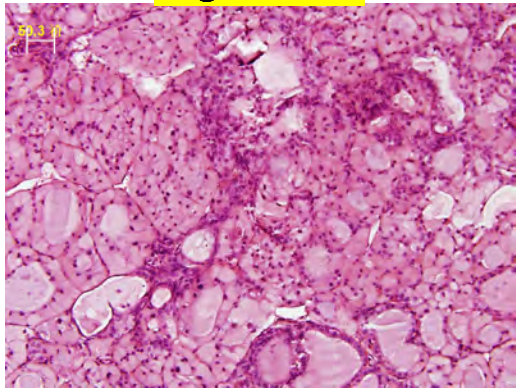

Tag#17733

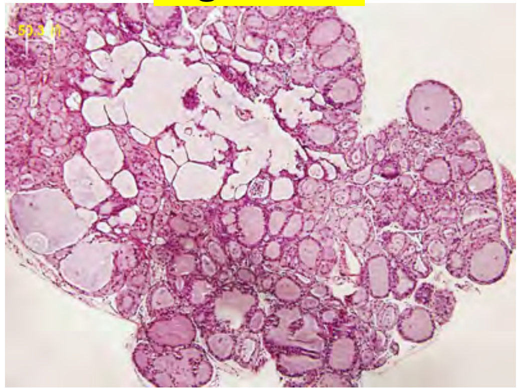

Tag#17738

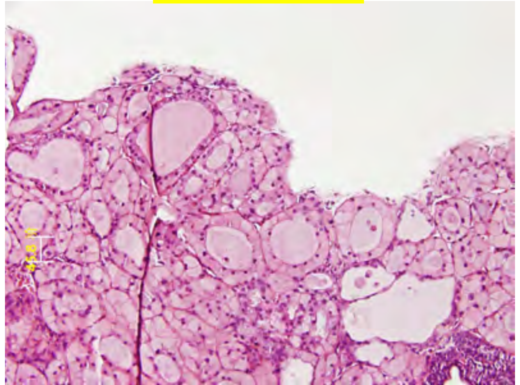

Fig. 6A

Fig. 7A

$TG^{+/cog}$;$Hrd1^{TPO}$

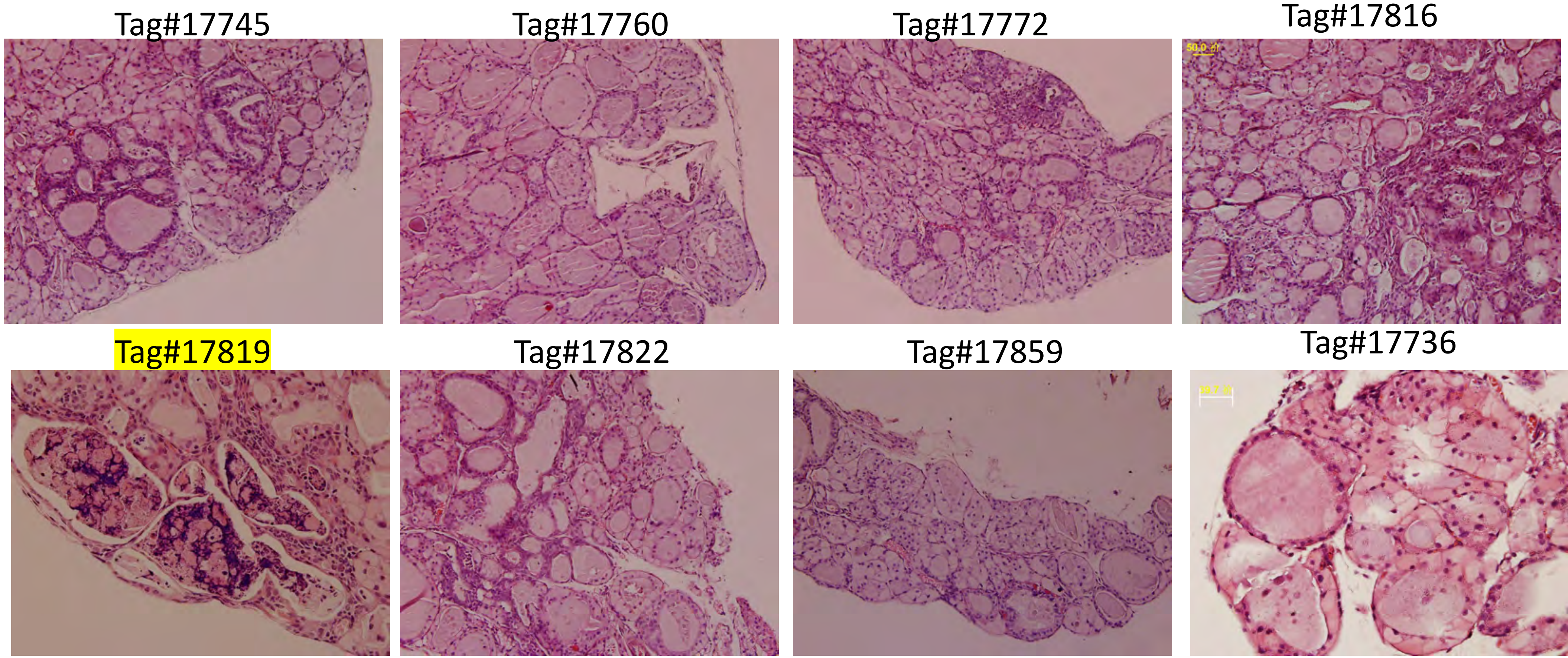

(Recent degeneration is multiple condensed nuclei; at a later stage, follicular cells removed with lighter staining follicle remnant(s).

Fig. 6B

TUNEL DAPI

$TG^{+/cog};Hrd1^{control}$

Representative image

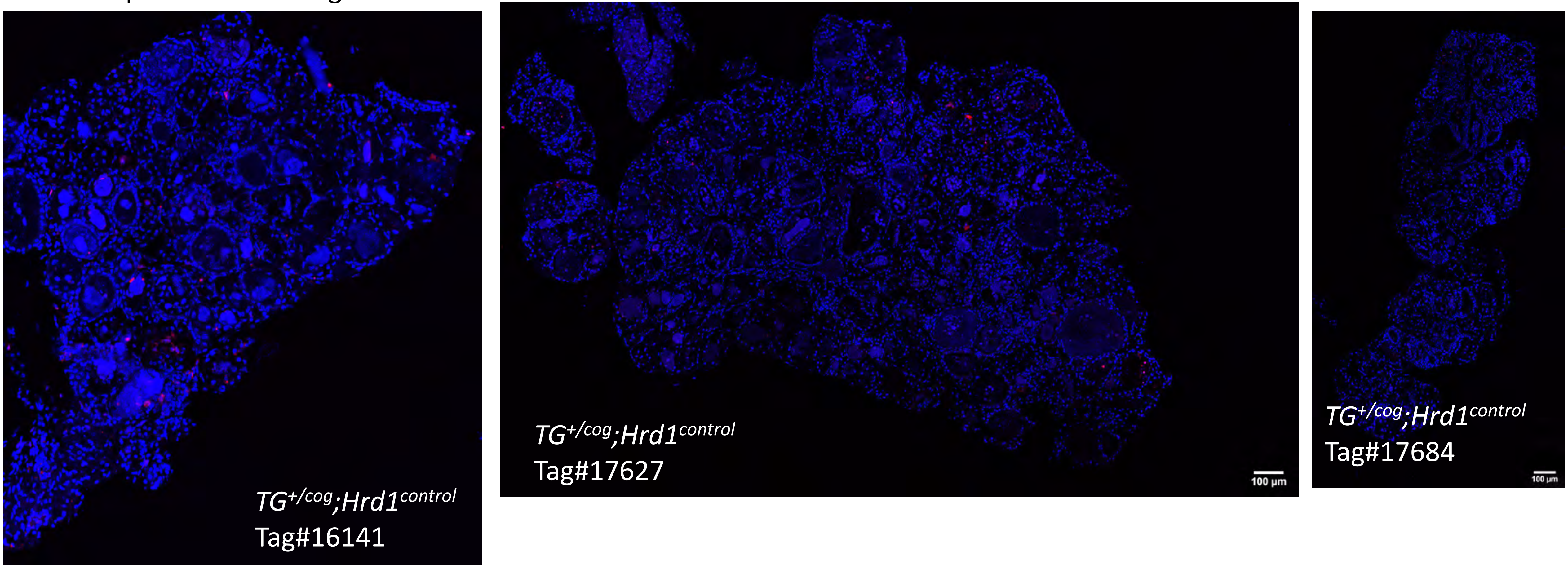

Fig. 6B TUNEL DAPI

TG$^{+/cog}$*;Hrd1*^{control}

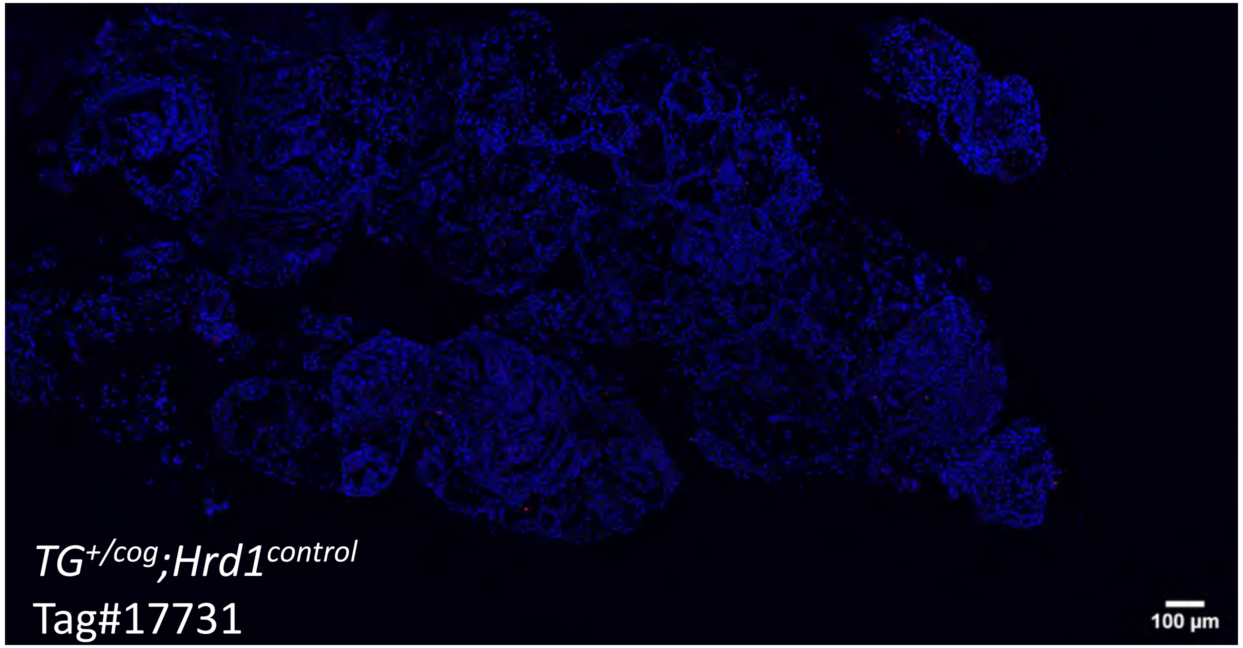

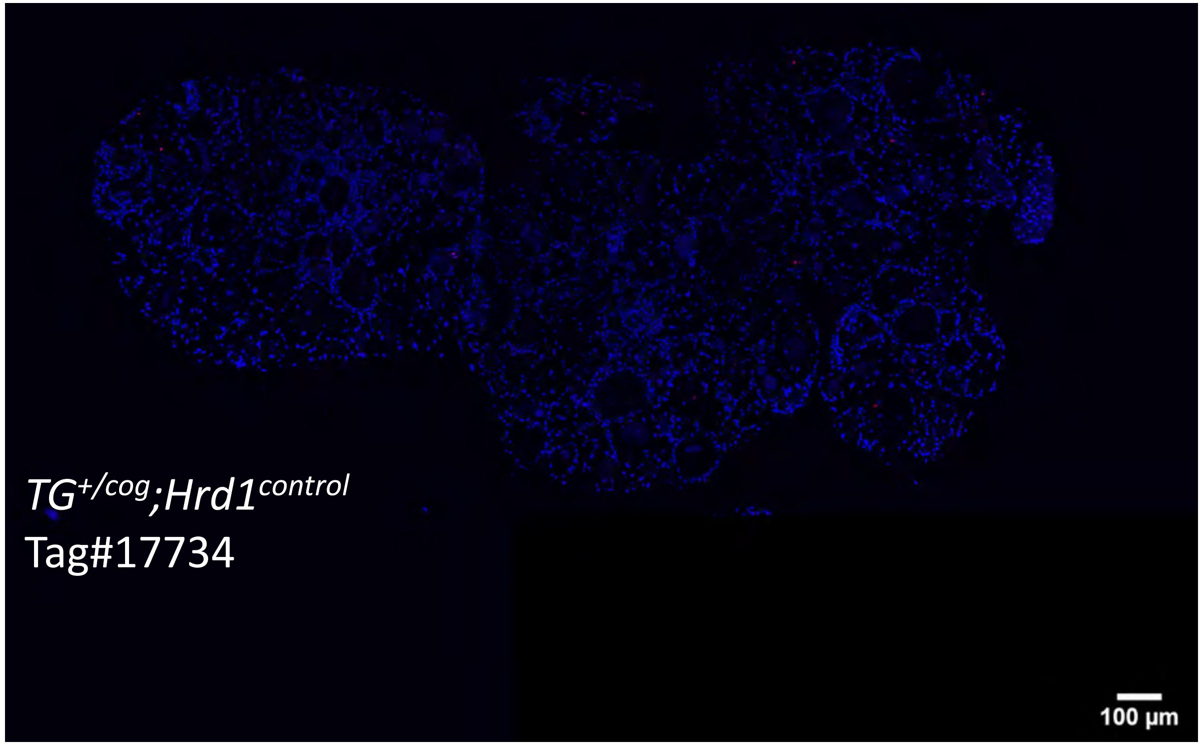

Fig. 6B TUNEL DAPI

TG$^{+/cog}$*;Hrd1*^{control}

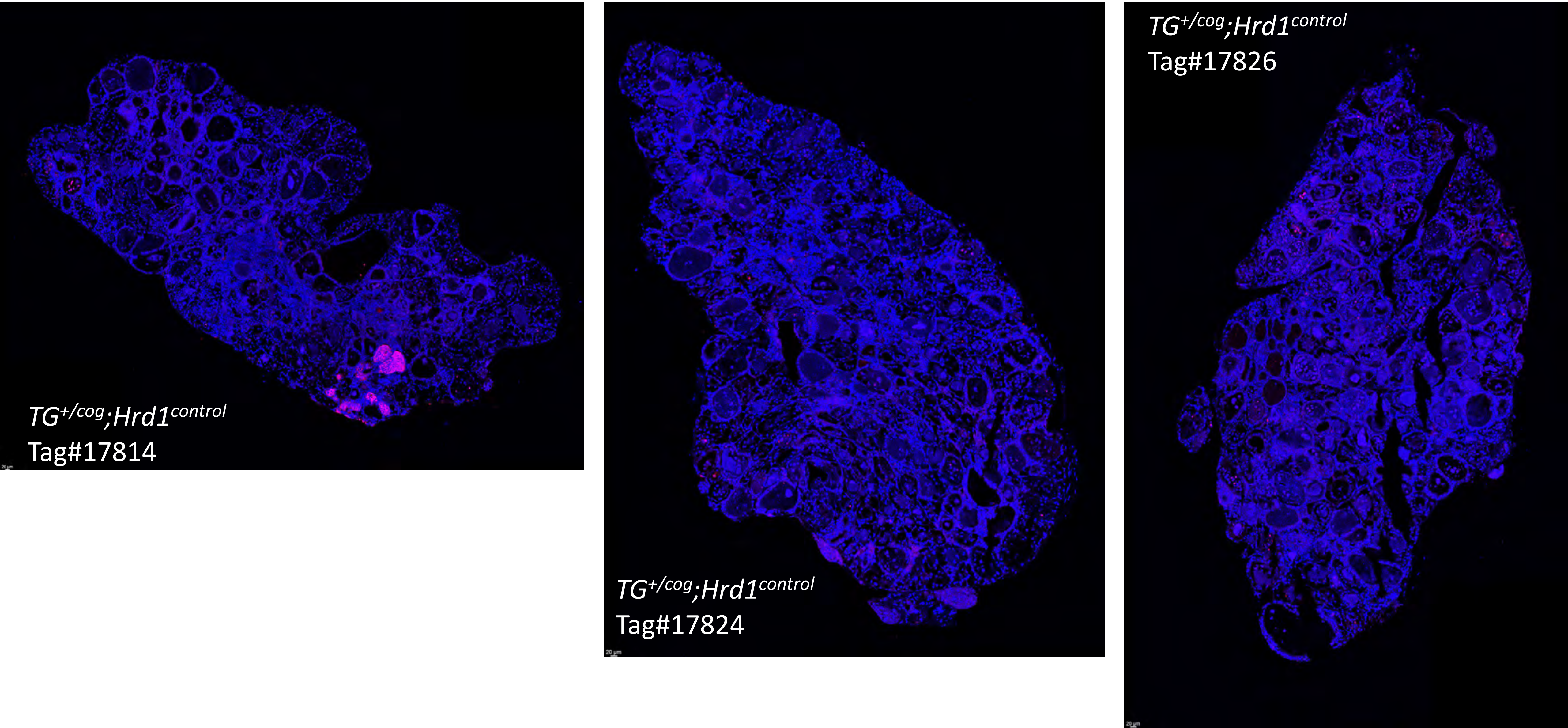

Fig. 6B

TUNEL DAPI

TG$^{+/cog}$*;Hrd1*^{control}

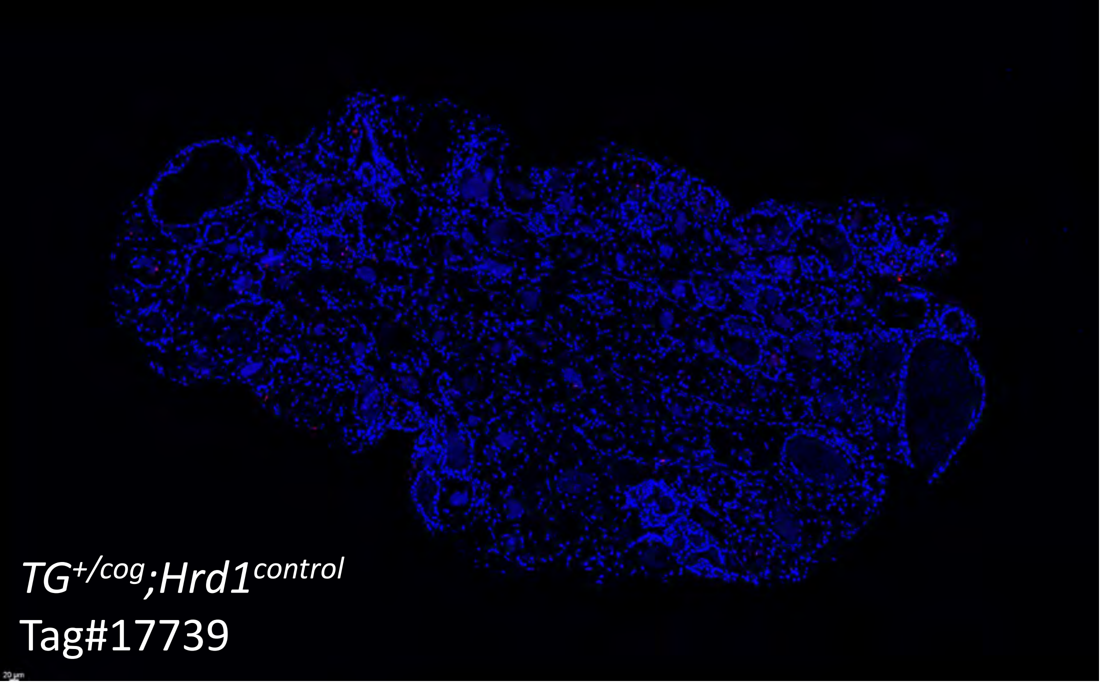

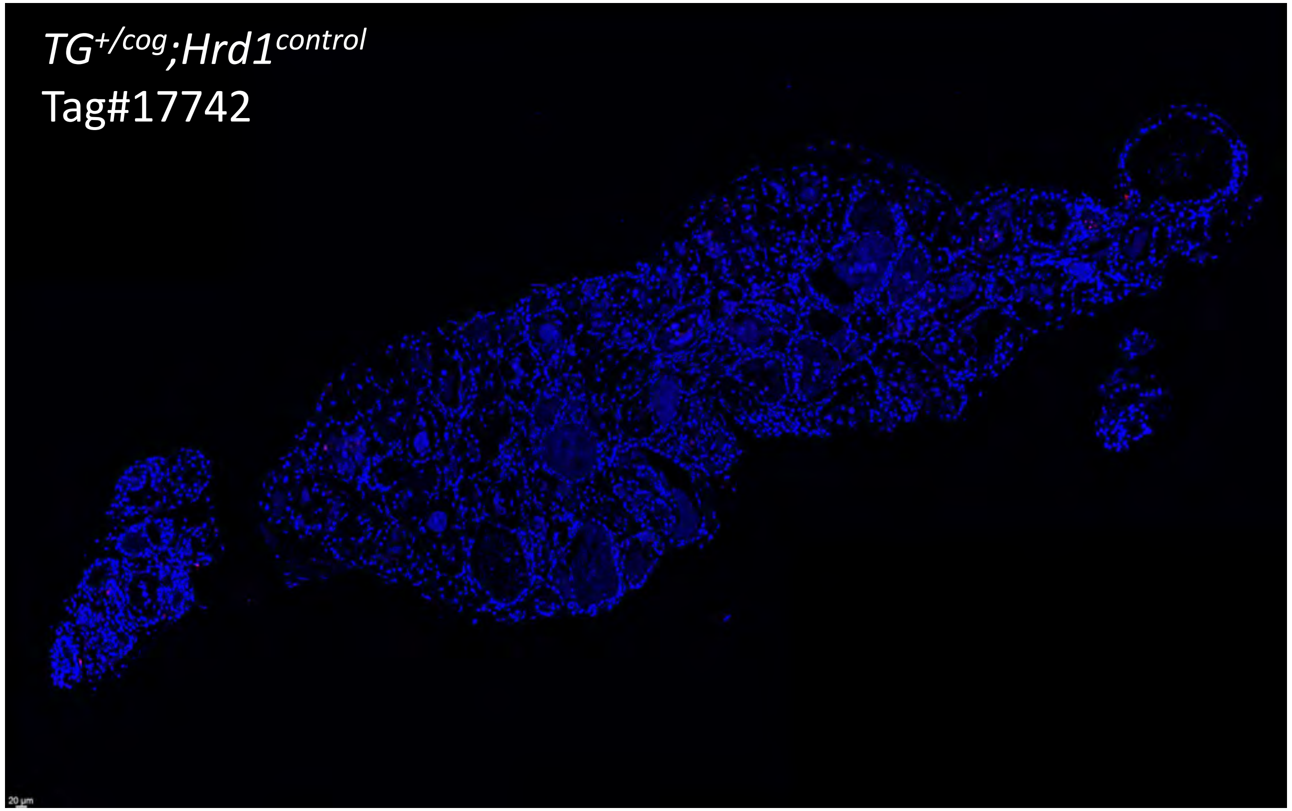

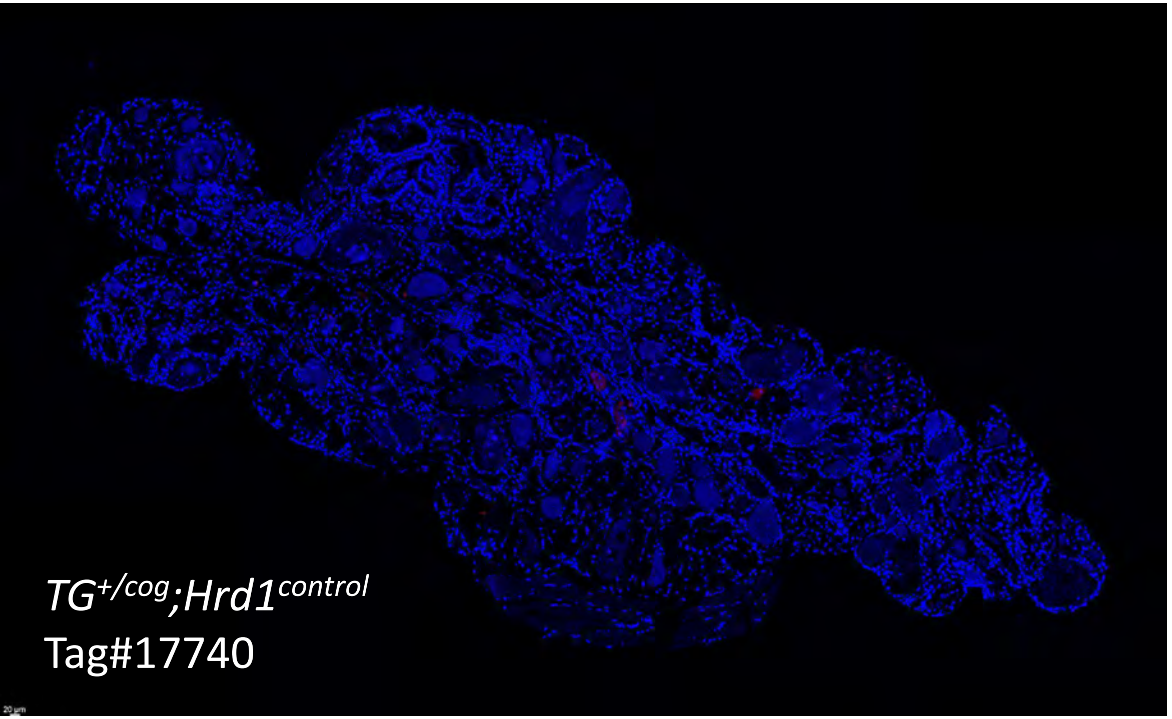

Fig. 6B TUNEL DAPI

$TG^{+/cog};Hrd1^{control}$

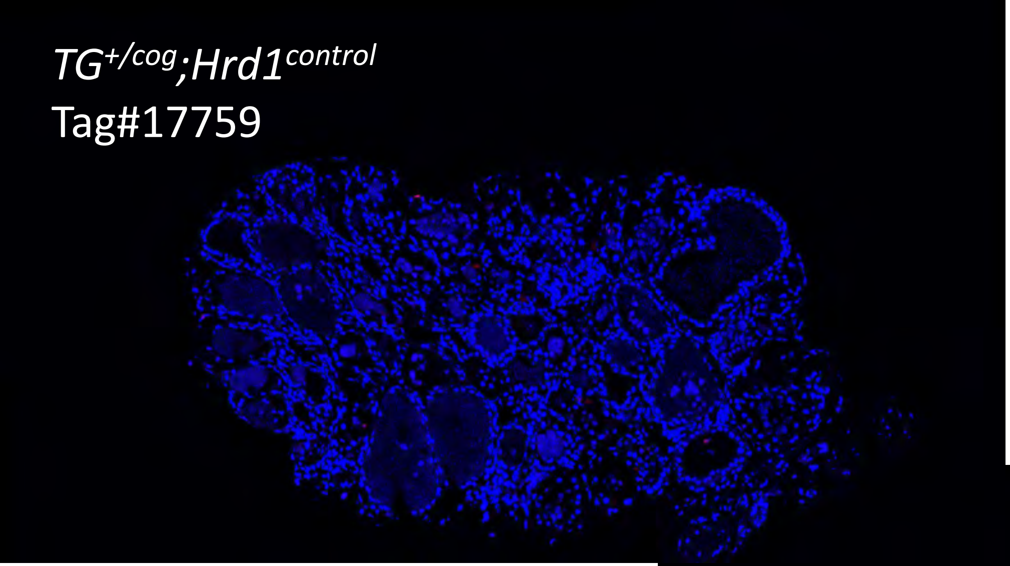

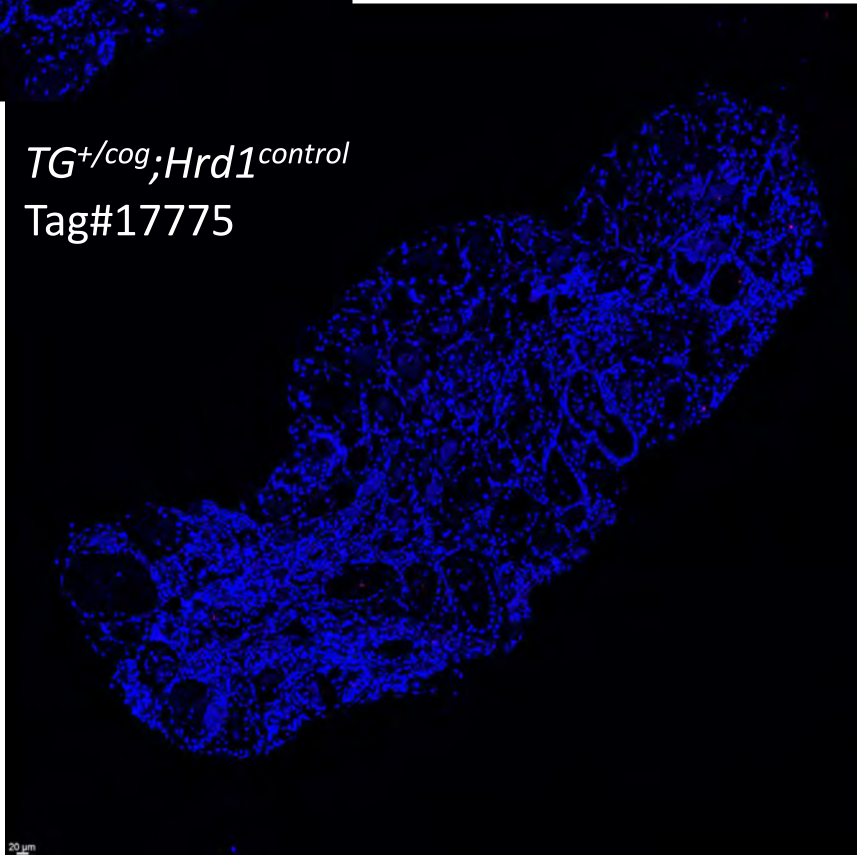

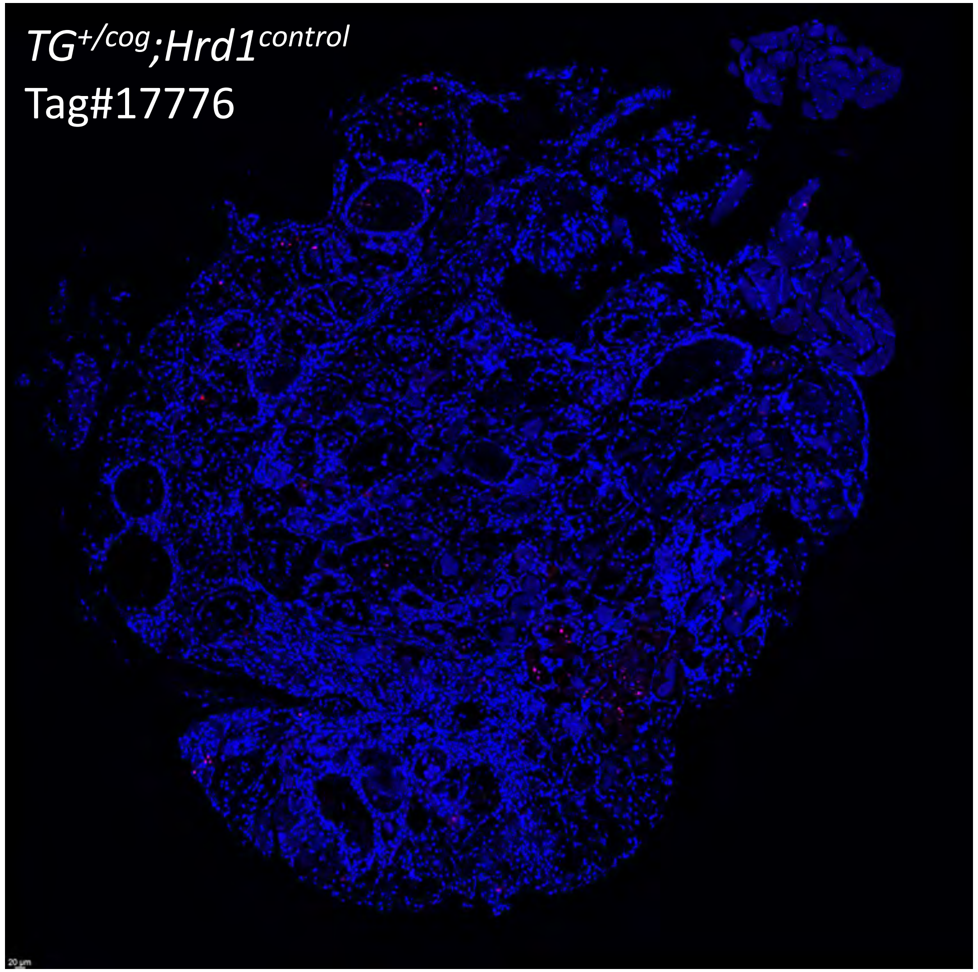

Fig. 6B

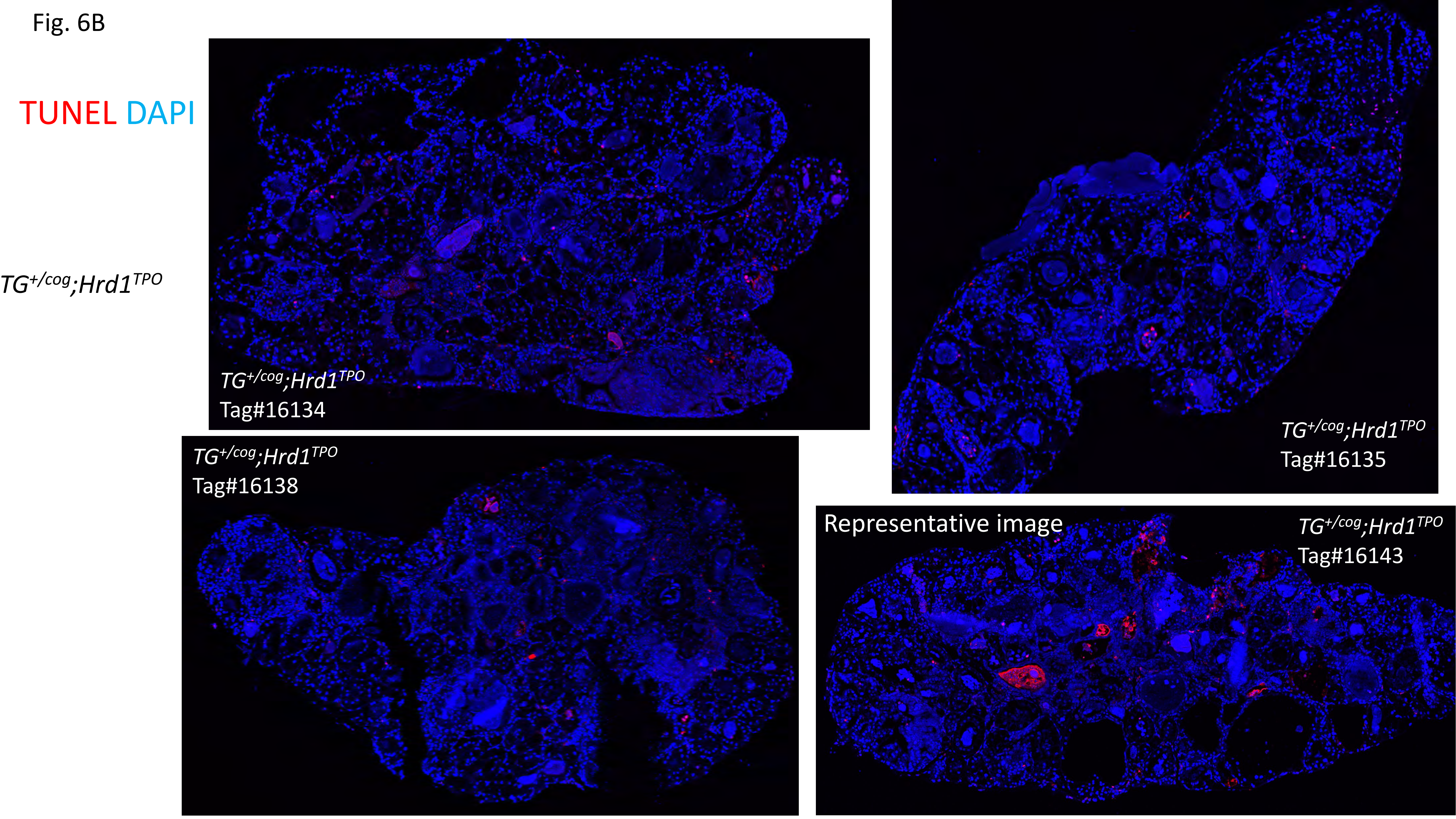
TUNEL DAPI
TG+/cog;Hrd1TPO
TG+/cog;Hrd1TPO
Tag#16134
TG+/cog;Hrd1TPO
Tag#16135
TG+/cog;Hrd1TPO
Tag#16138
Representative image
TG+/cog;Hrd1TPO
Tag#16143

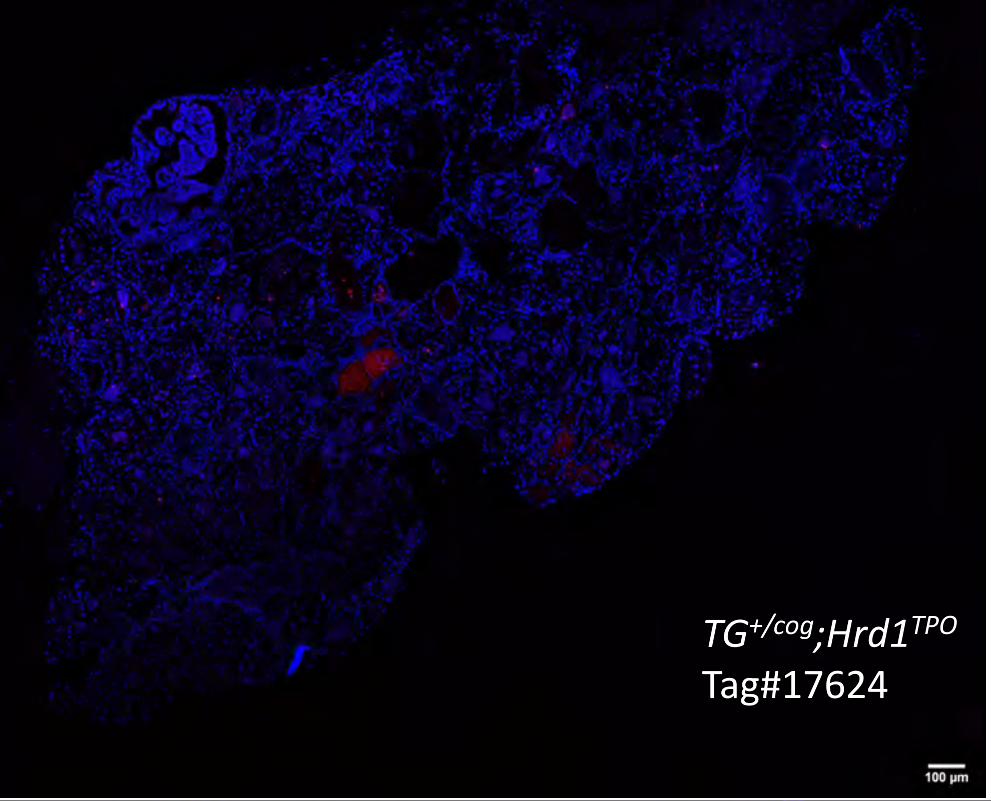

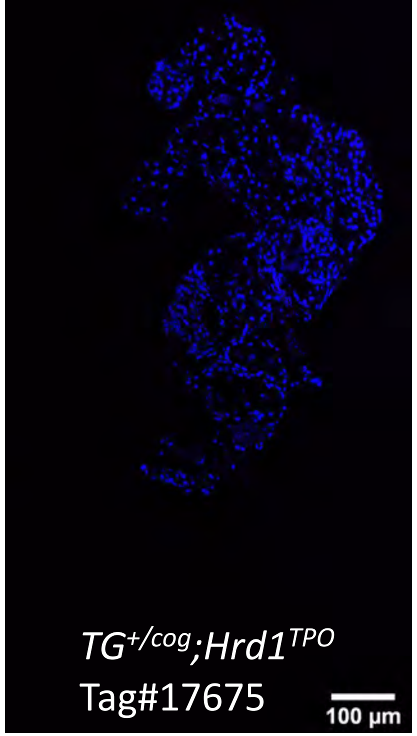

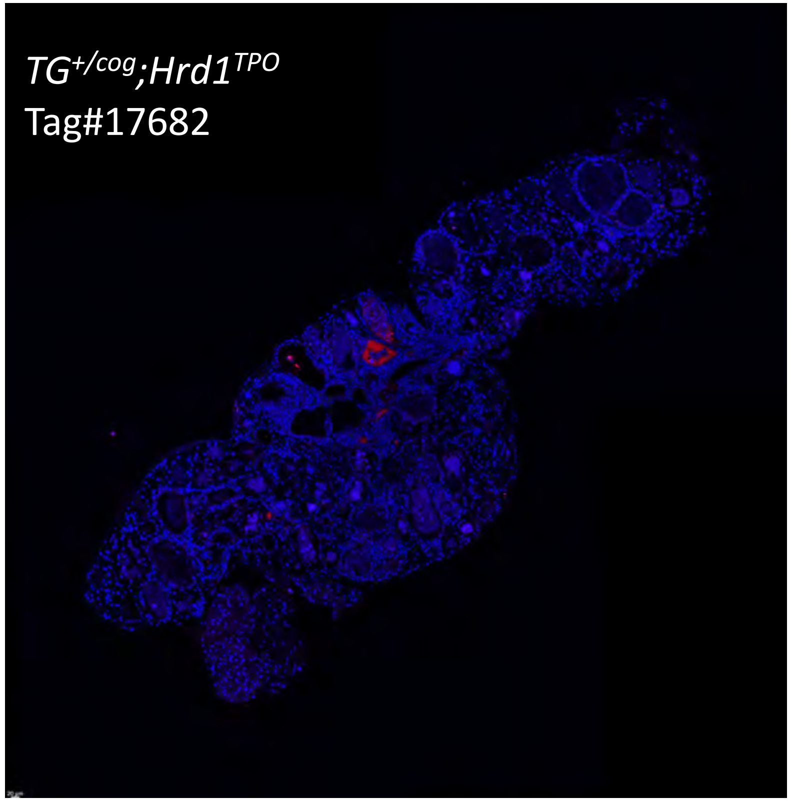

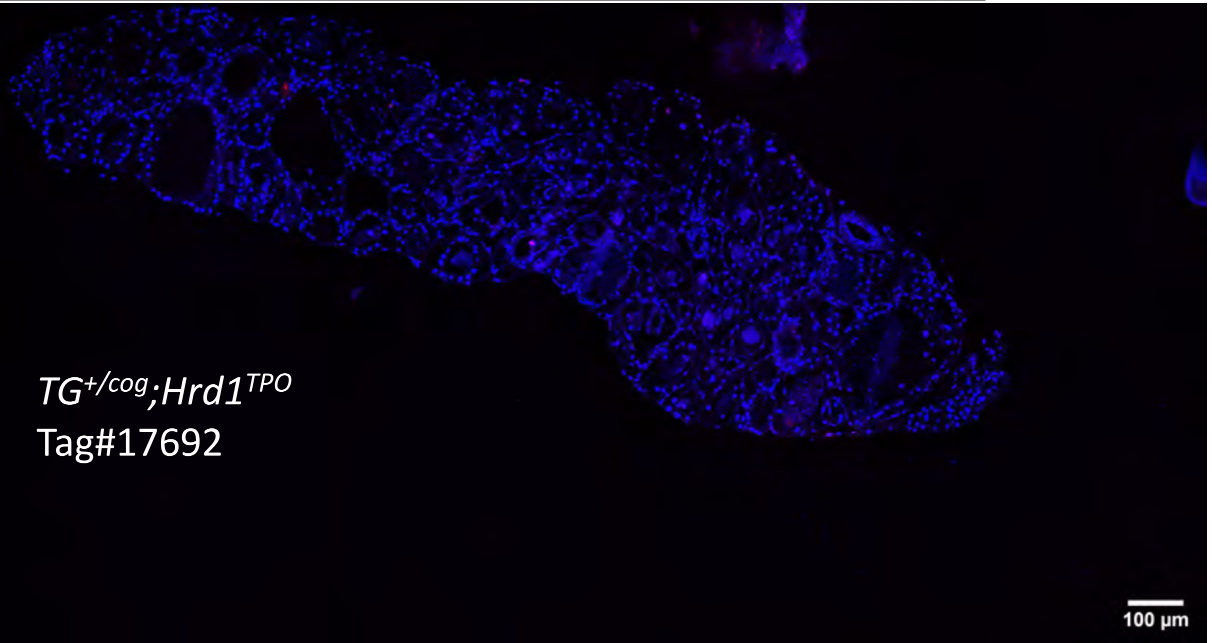

Fig. 6B TUNEL DAPI

$TG^{+/cog};Hrd1^{TPO}$

Fig. 6B

TUNEL DAPI

TG$^{+/cog}$*;Hrd1*^{TPO}

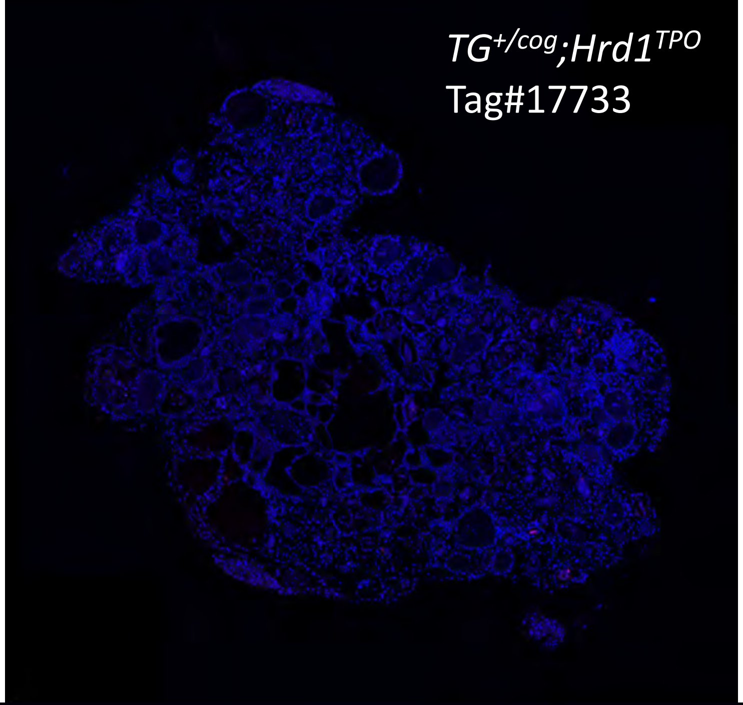

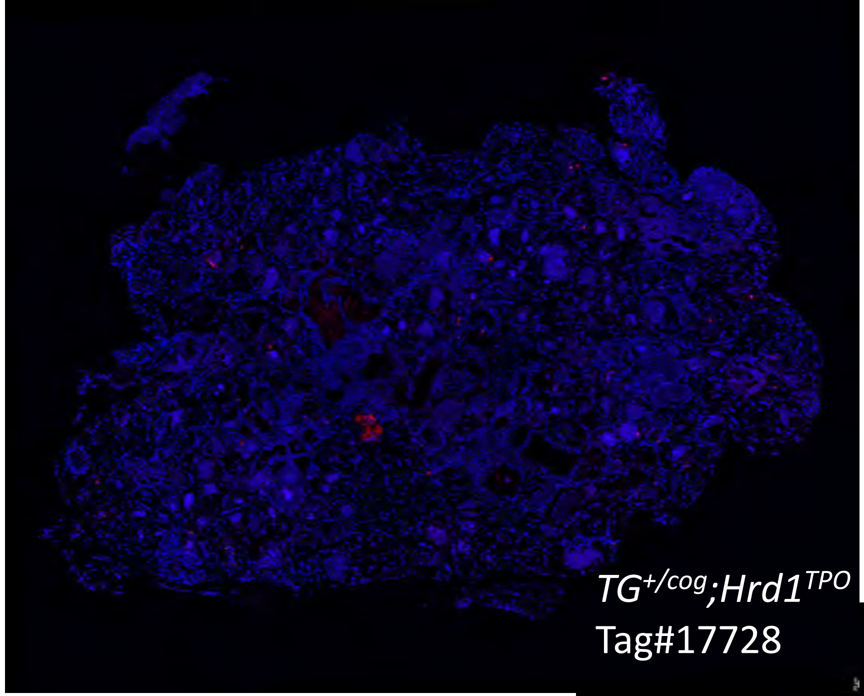

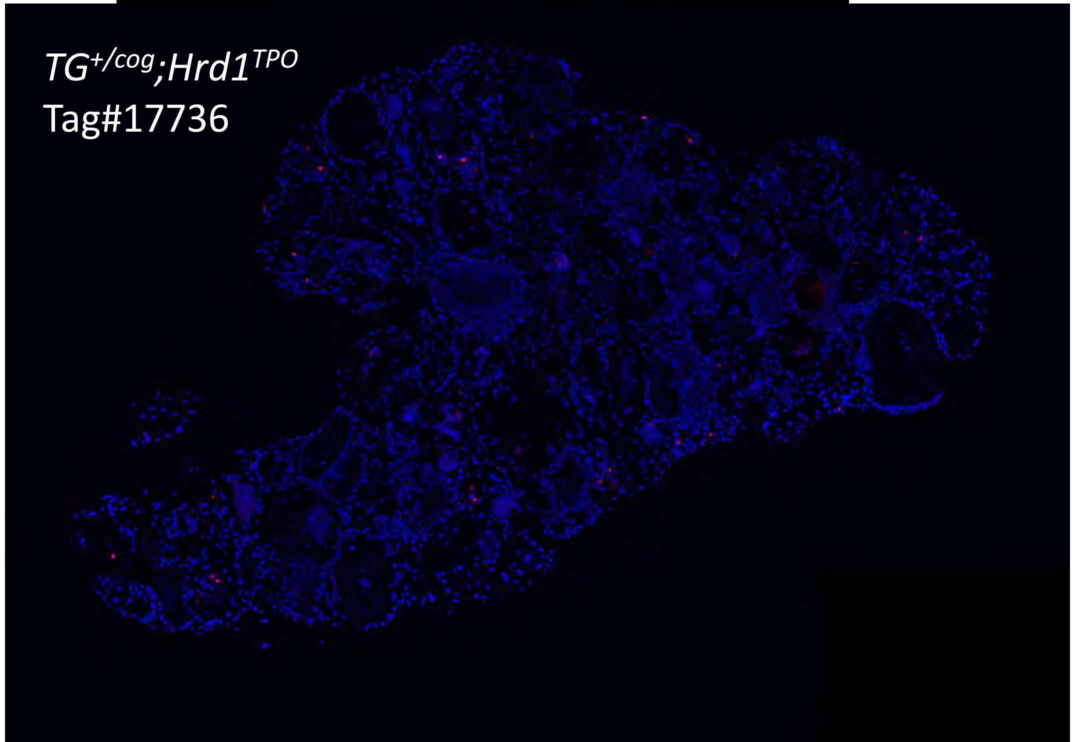

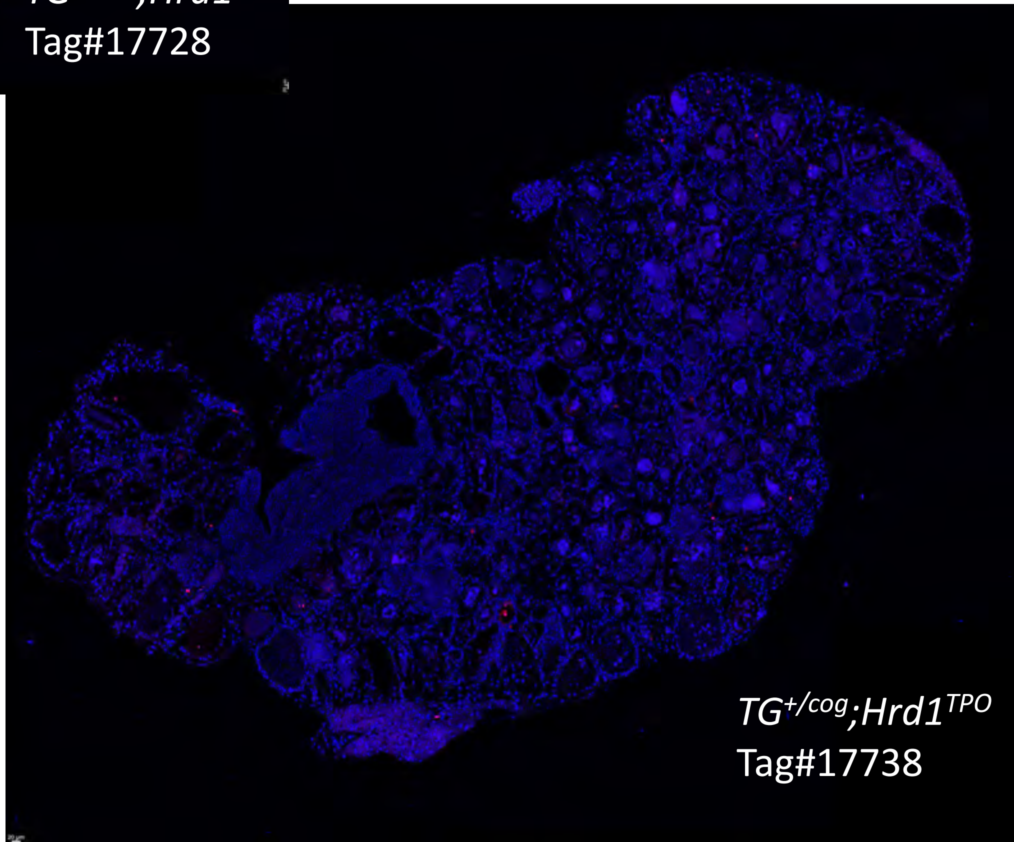

Fig. 6B

TUNEL DAPI

$TG^{+/cog}$;$Hrd1^{TPO}$

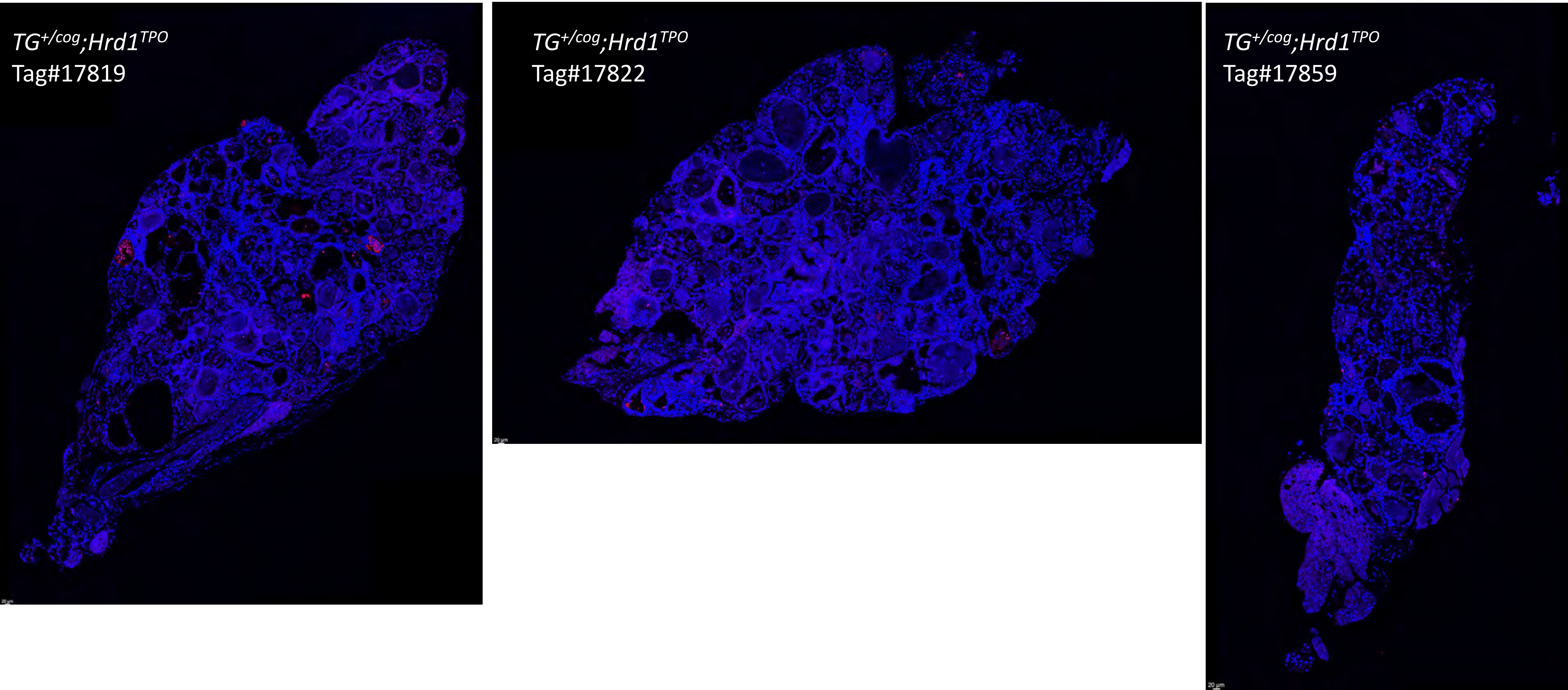

Fig. 6B

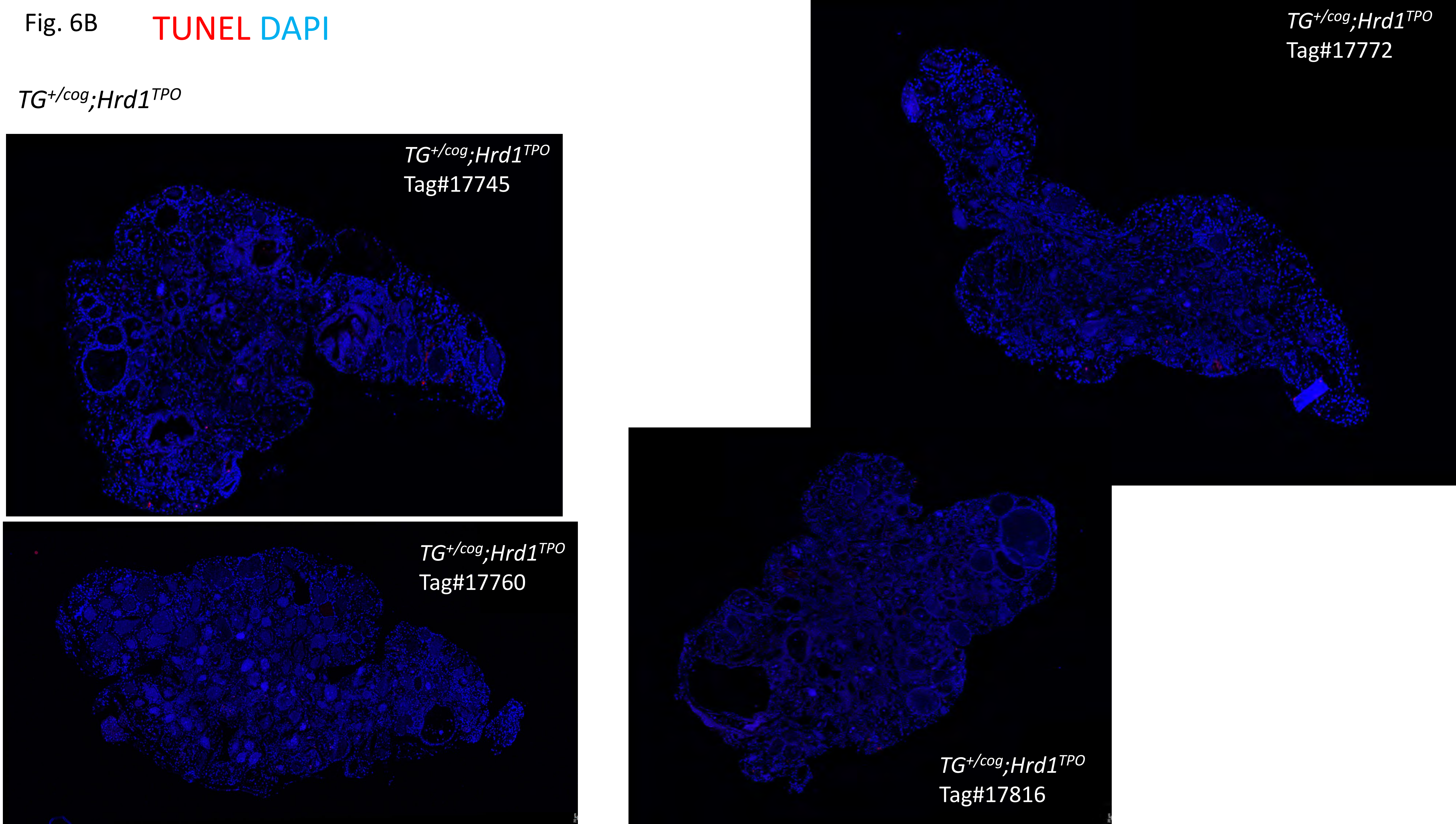
TUNEL DAPI
TG+/cog;Hrd1TPO
TG+/cog;Hrd1TPO
Tag#17745
TG+/cog;Hrd1TPO
Tag#17760
TG+/cog;Hrd1TPO
Tag#17772
TG+/cog;Hrd1TPO
Tag#17816

Fig. 7B

CD45 DAPI

Representative images

Tag# 16933 $TG^{+/cog}$; $Hrd1^{control}$

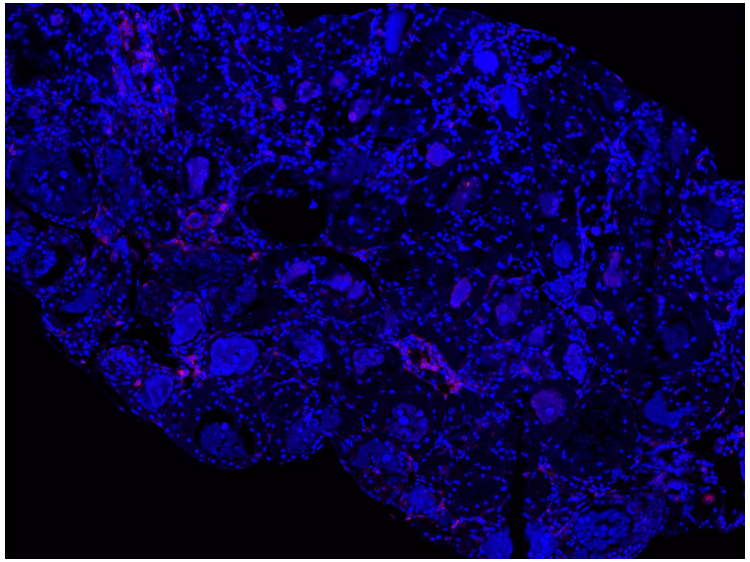

Tag# 17675 $TG^{+/cog}$; $Hrd1^{TPO}$

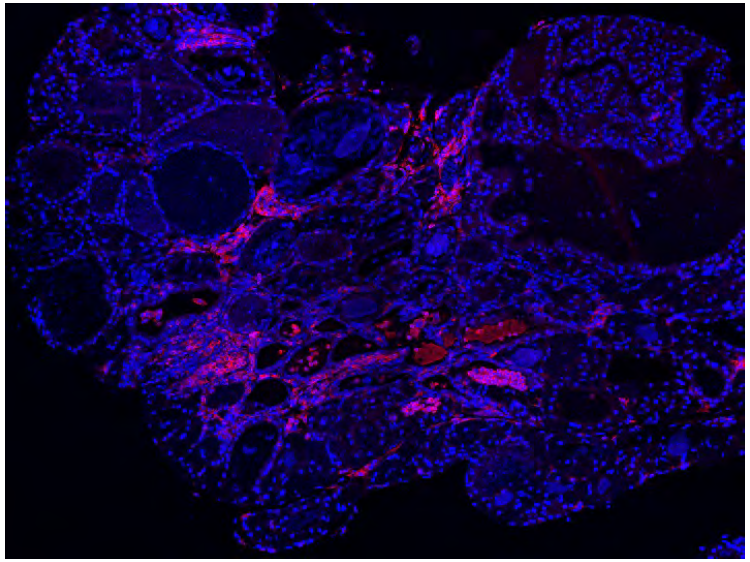

Quantification:

tag	*genotype*	total nuclei	cd45 positive	cd45 positive%
17684	$TG^{+/cog}$;$Hrd1^{control}$	1959	56	2.858601
17690	$TG^{+/cog}$;$Hrd1^{control}$	1041	23	2.209414
17734	$TG^{+/cog}$;$Hrd1^{control}$	6775	208	3.070111
17759	$TG^{+/cog}$;$Hrd1^{control}$	2246	62	2.760463
17776	$TG^{+/cog}$;$Hrd1^{control}$	7933	28	0.352956
17824	$TG^{+/cog}$;$Hrd1^{control}$	8786	154	1.752789
17633	$TG^{+/cog}$;$Hrd1^{control}$	9509	101	1.062152
17686	$TG^{+/cog}$;$Hrd1^{control}$	3009	49	1.628448
17731	$TG^{+/cog}$;$Hrd1^{control}$	12947	289	2.232177
17739	$TG^{+/cog}$;$Hrd1^{control}$	4788	122	2.548037
17740	$TG^{+/cog}$;$Hrd1^{control}$	5759	189	3.28182
17742	$TG^{+/cog}$;$Hrd1^{control}$	3371	96	2.84782
17692	$TG^{+/cog}$;$Hrd1^{TPO}$	2599	192	7.387457
17728	$TG^{+/cog}$;$Hrd1^{TPO}$	8514	202	2.372563
17733	$TG^{+/cog}$;$Hrd1^{TPO}$	9733	616	6.328984
17736	$TG^{+/cog}$;$Hrd1^{TPO}$	6578	227	3.450897
17738	$TG^{+/cog}$;$Hrd1^{TPO}$	6555	322	4.912281
17745	$TG^{+/cog}$;$Hrd1^{TPO}$	776	33	4.252577
17772	$TG^{+/cog}$;$Hrd1^{TPO}$	3518	119	3.382604
17859	$TG^{+/cog}$;$Hrd1^{TPO}$	3867	212	5.482286
17624	$TG^{+/cog}$;$Hrd1^{TPO}$	3421	324	9.470915
17675	$TG^{+/cog}$;$Hrd1^{TPO}$	2971	391	13.16055

Fig. 7B

CD45 DAPI

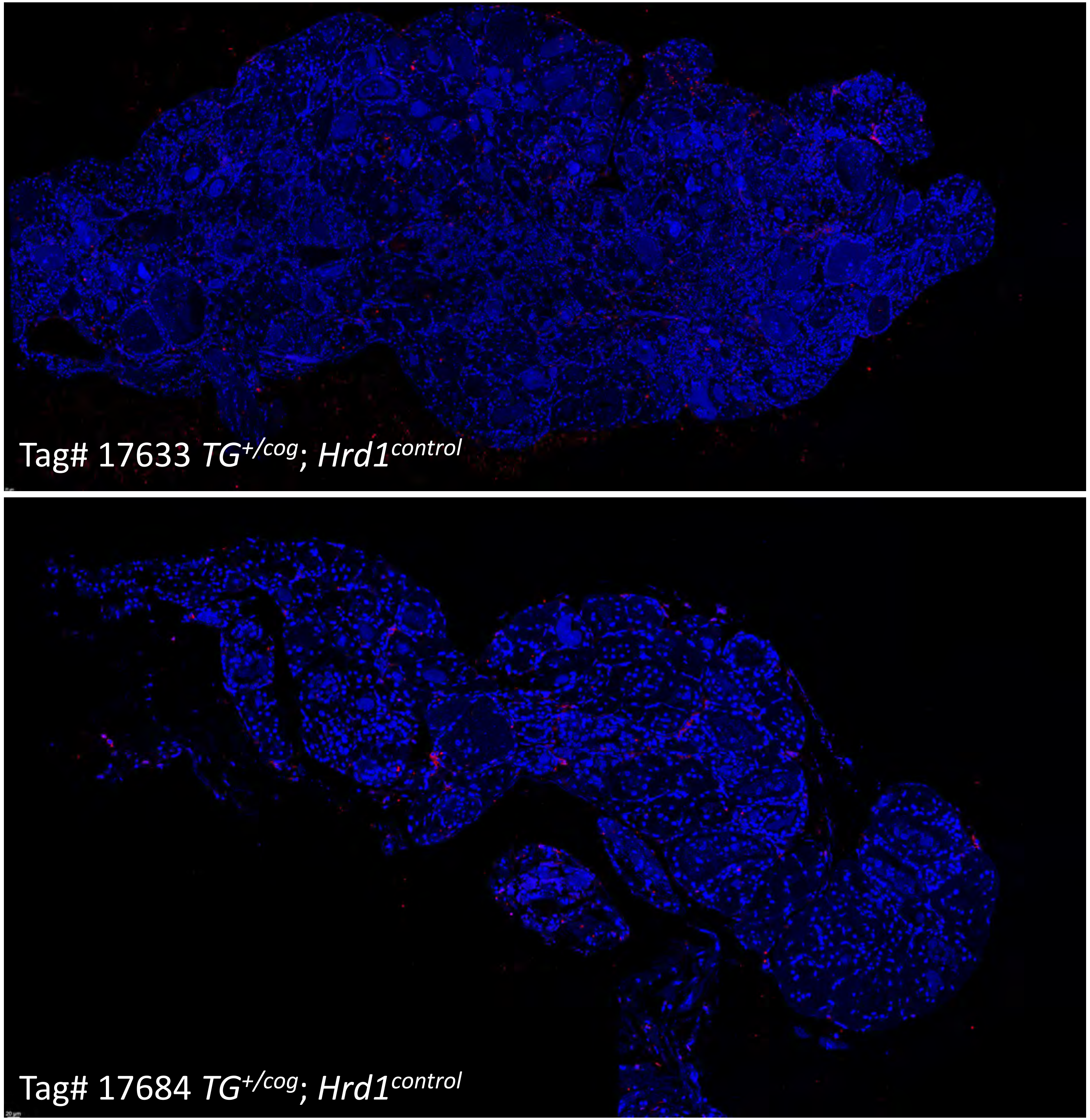

Fig. 7B

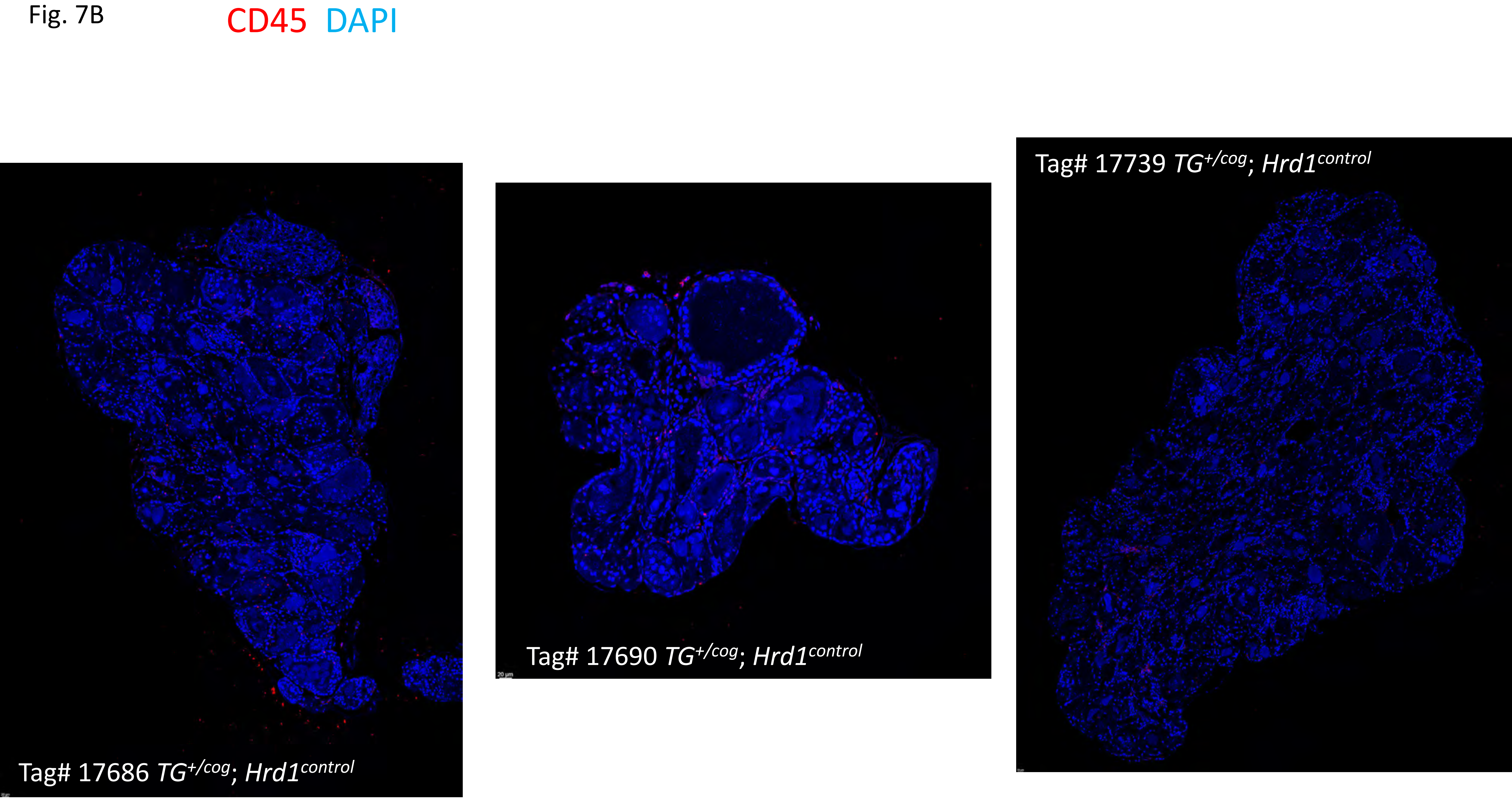
CD45 DAPI
Tag# 17686 TG+/cog; Hrd1control
Tag# 17690 TG+/cog; Hrd1control
Tag# 17739 TG+/cog; Hrd1control

Fig. 7B

CD45 DAPI

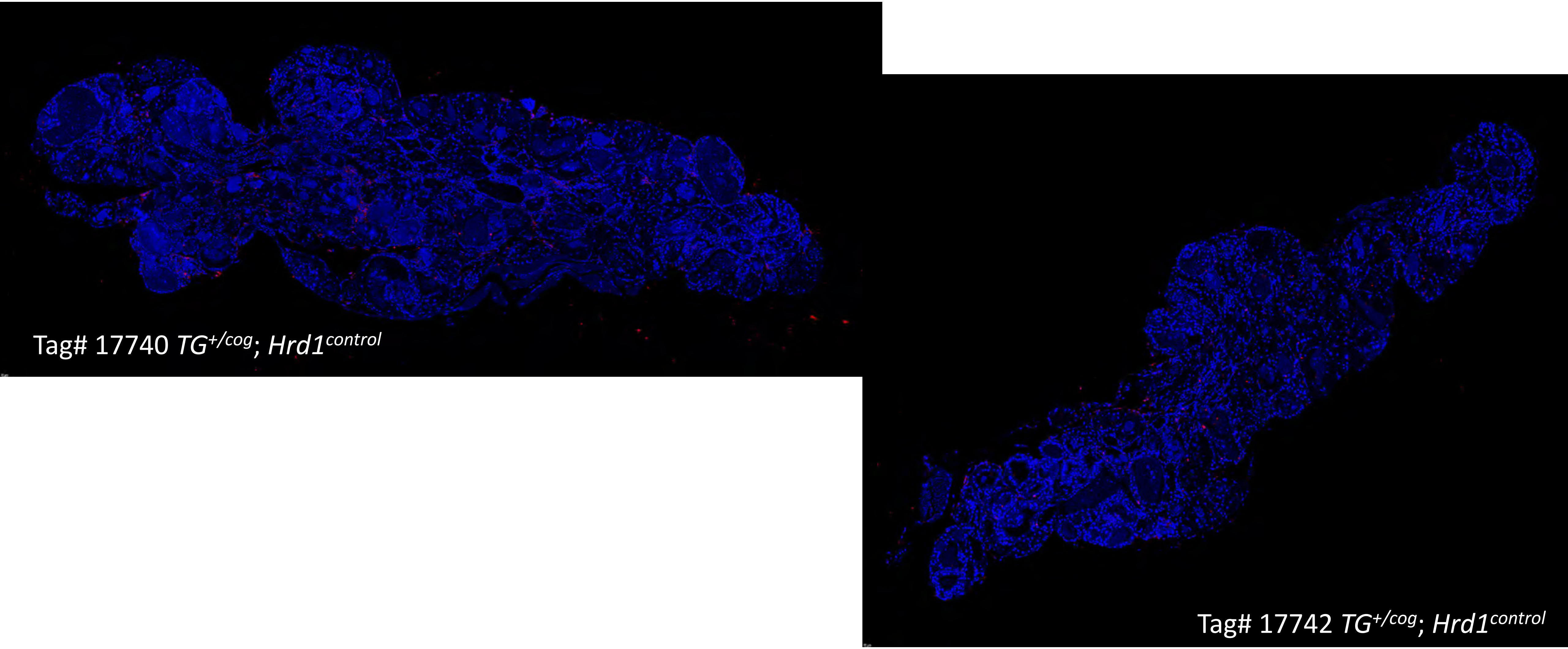

Fig. 7B

CD45 DAPI

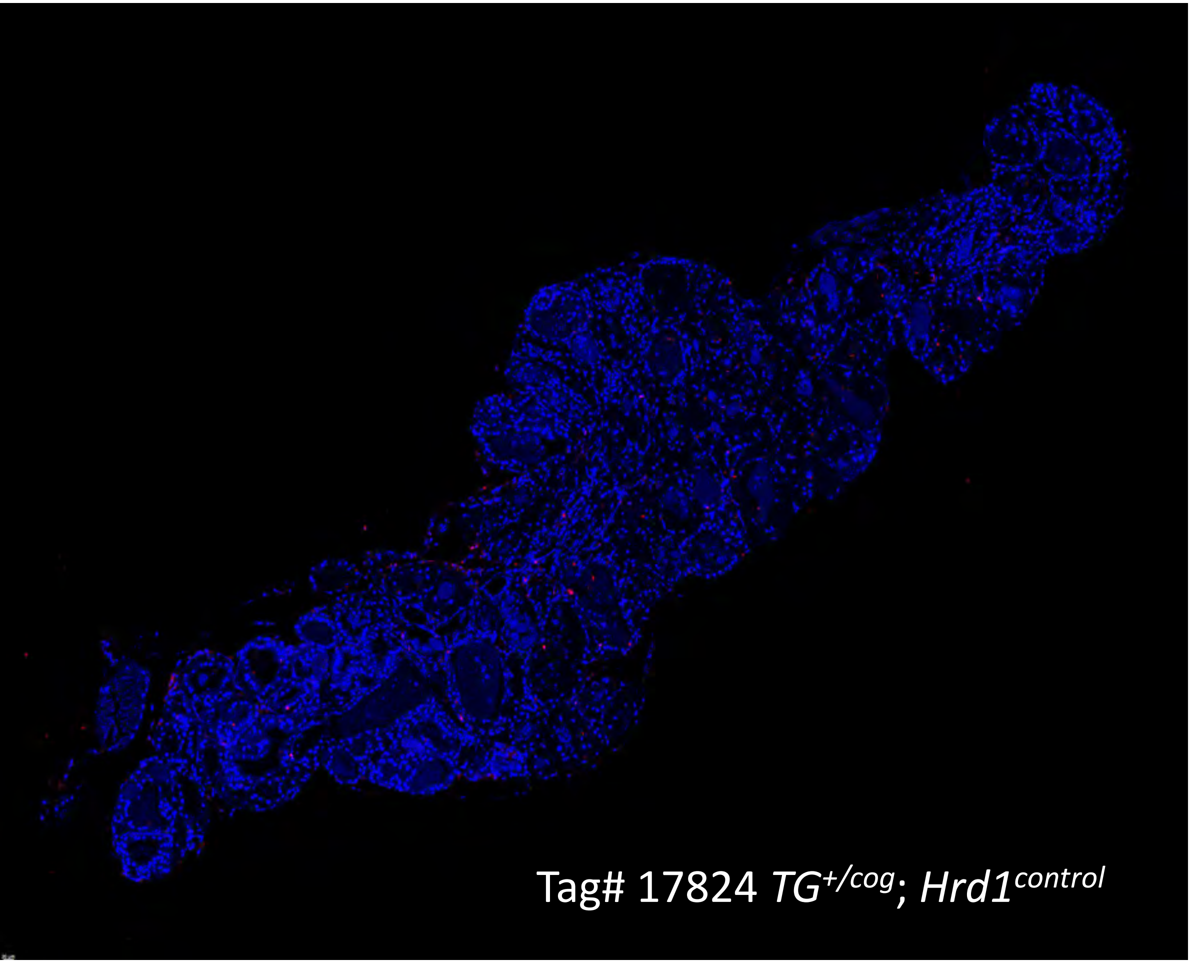

Fig. 7B

CD45 DAPI

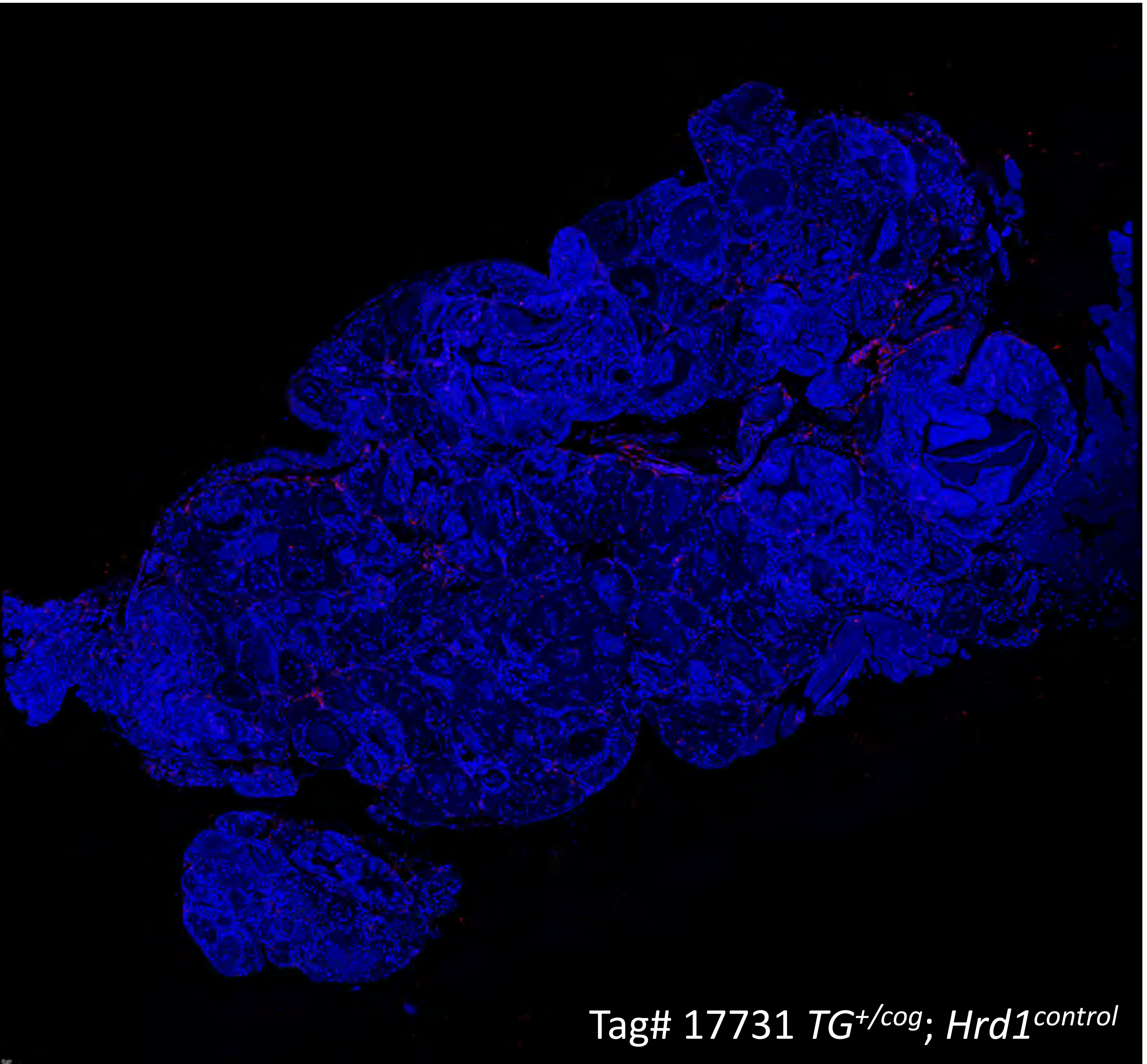

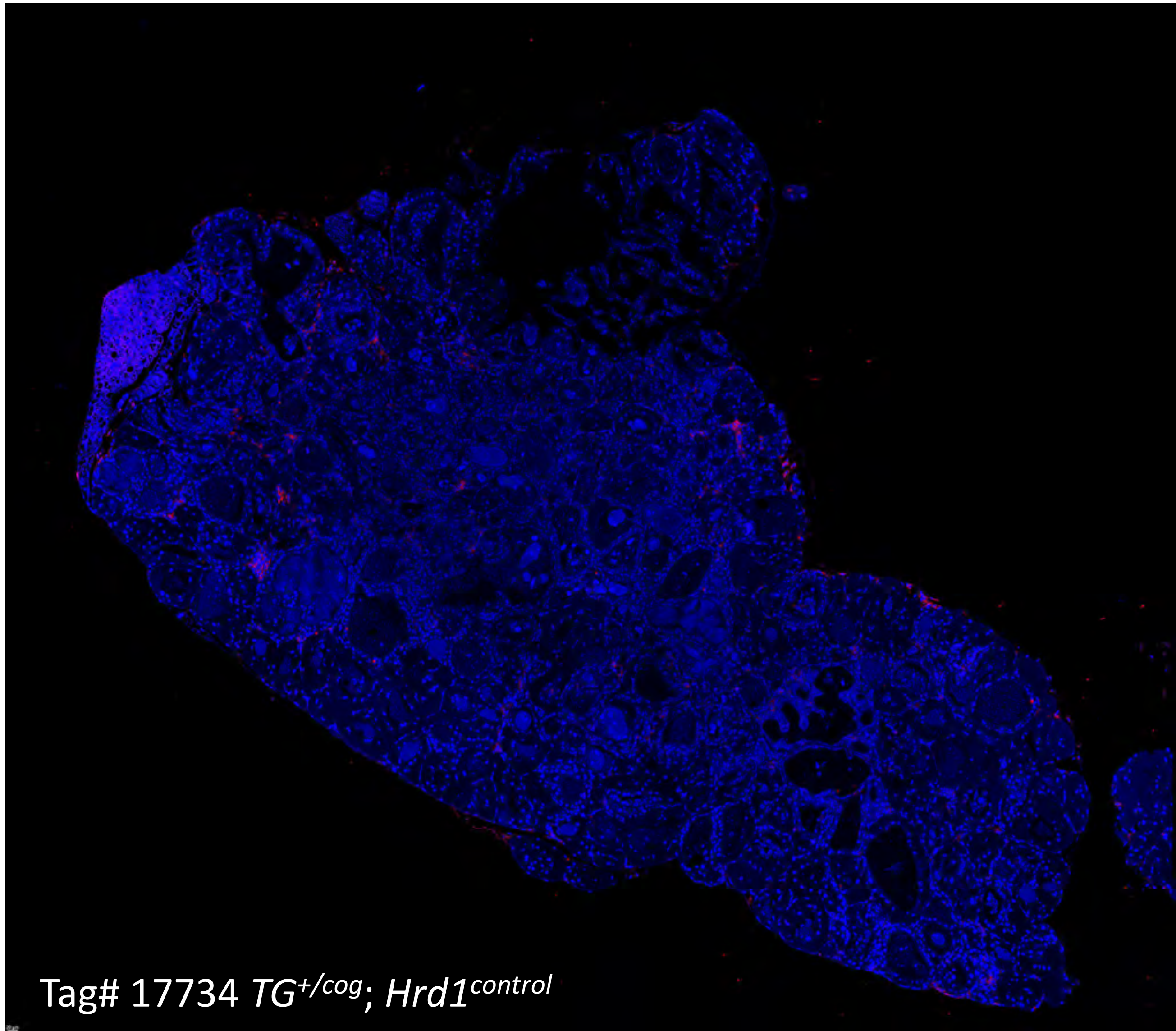

Fig. 7B

CD45 DAPI

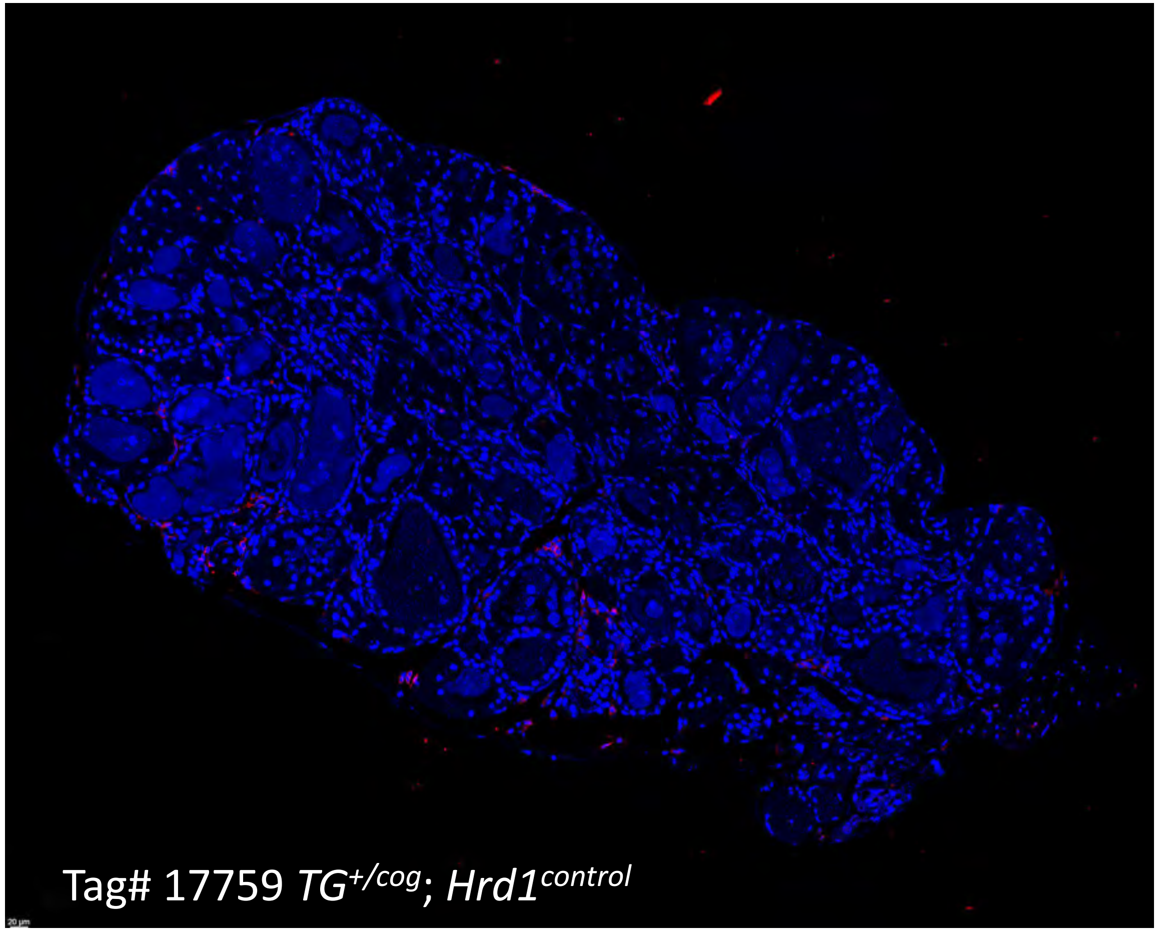

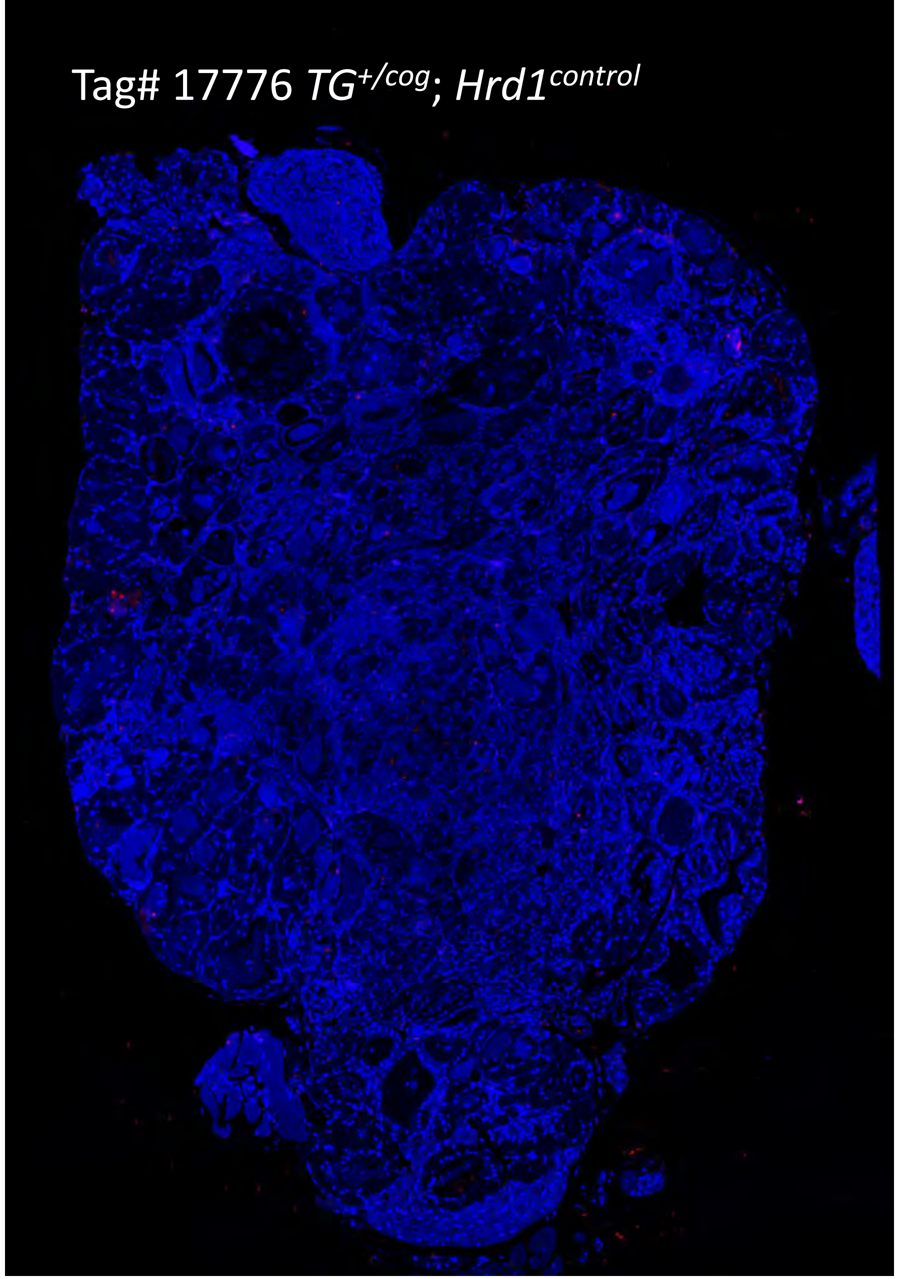

Fig. 7B

CD45 DAPI

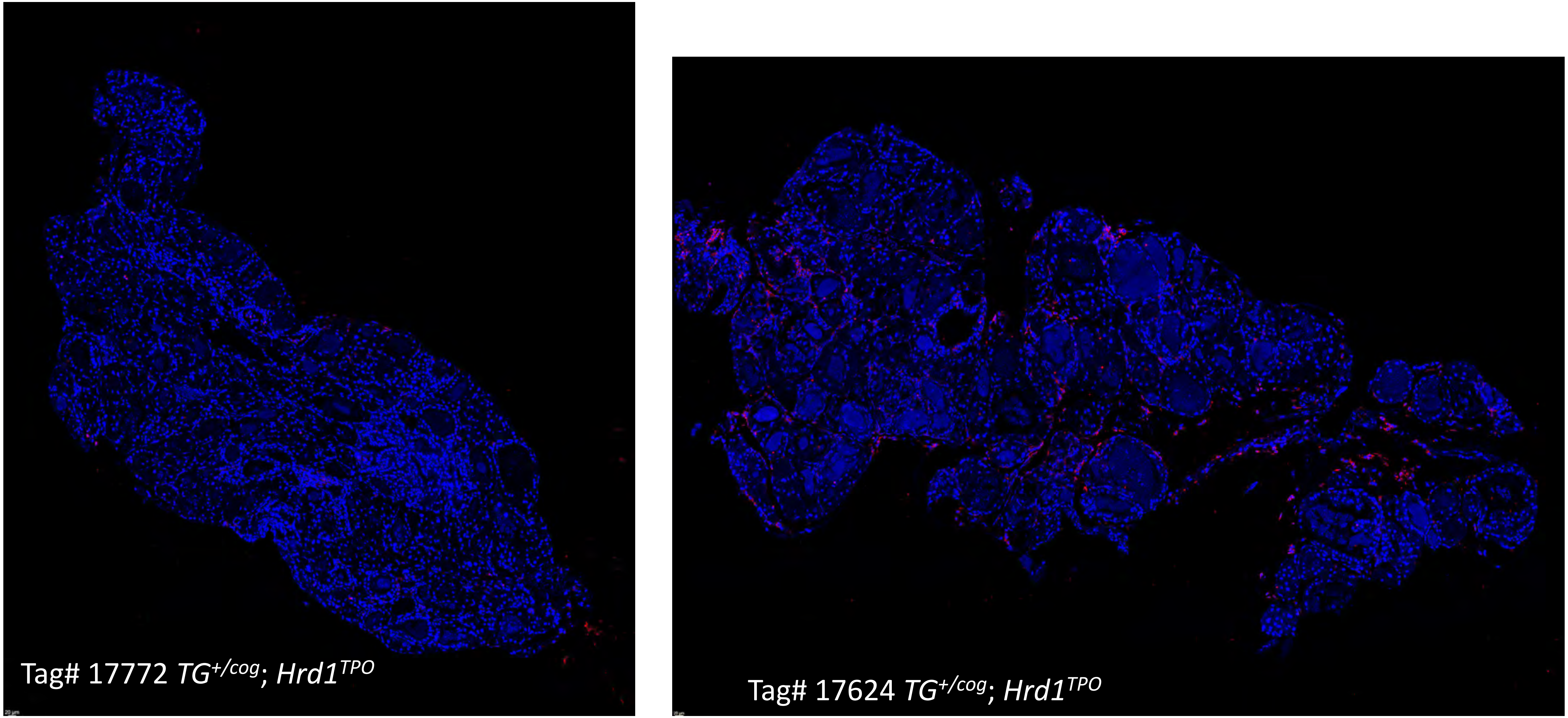

Fig. 7B

CD45 DAPI

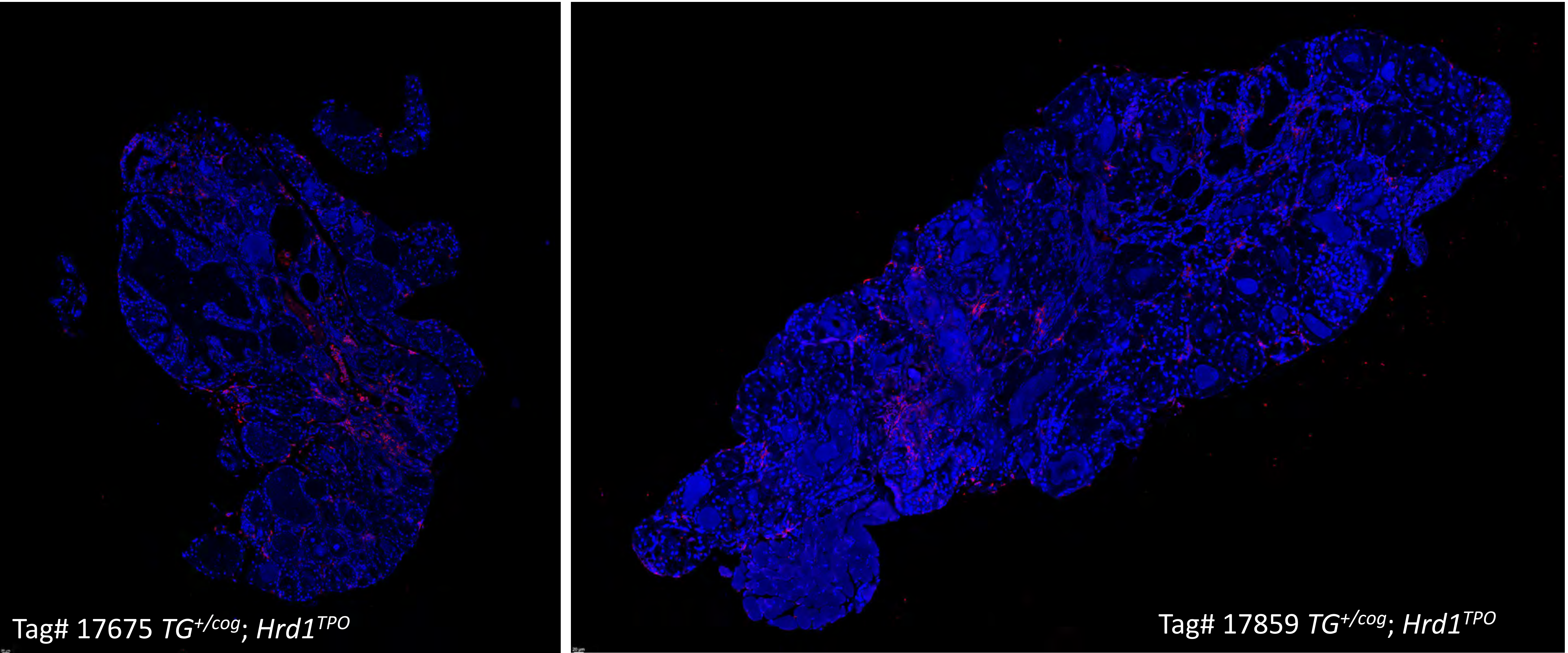

Fig. 7B

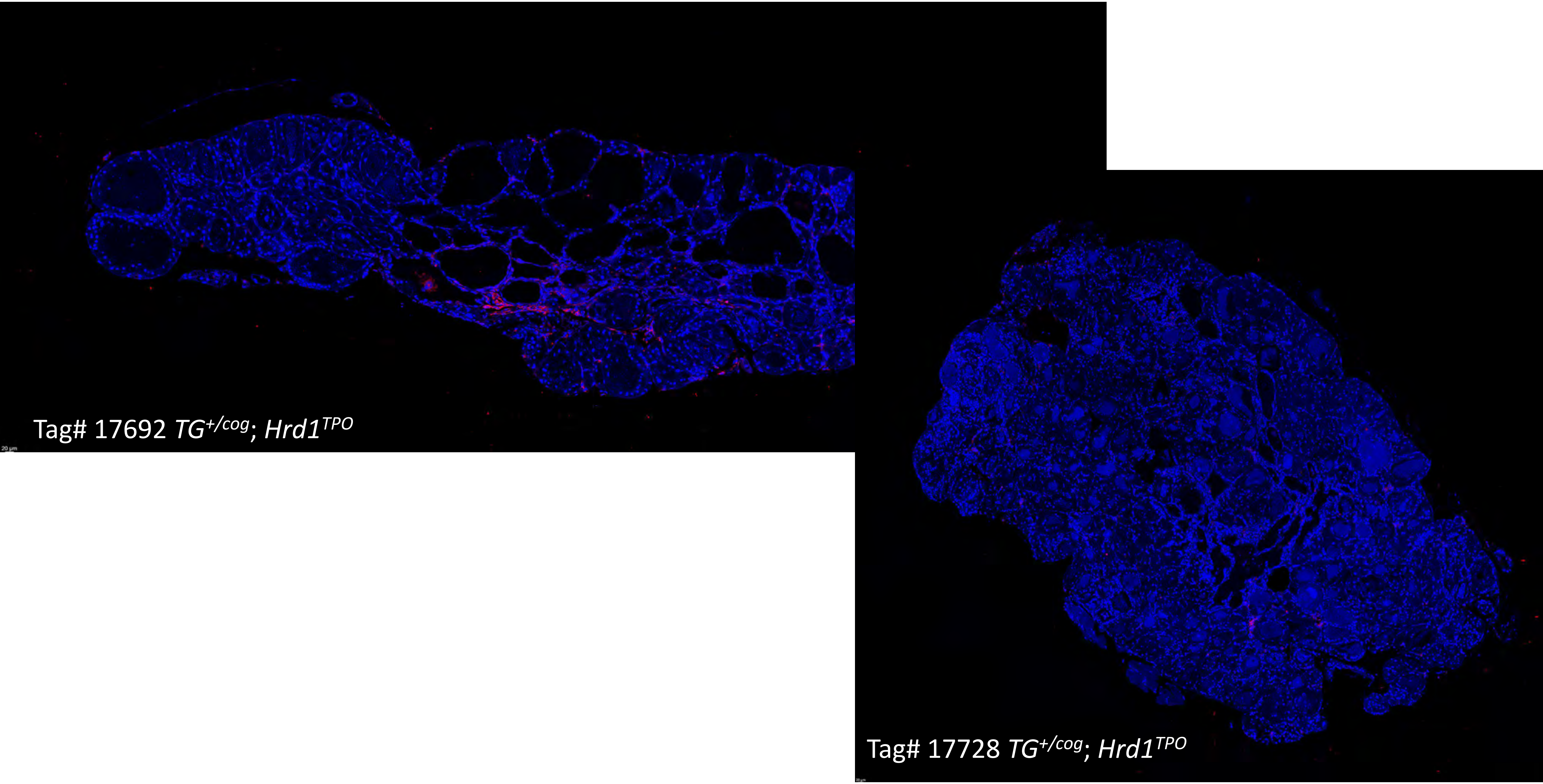

Fig. 7B

CD45 DAPI

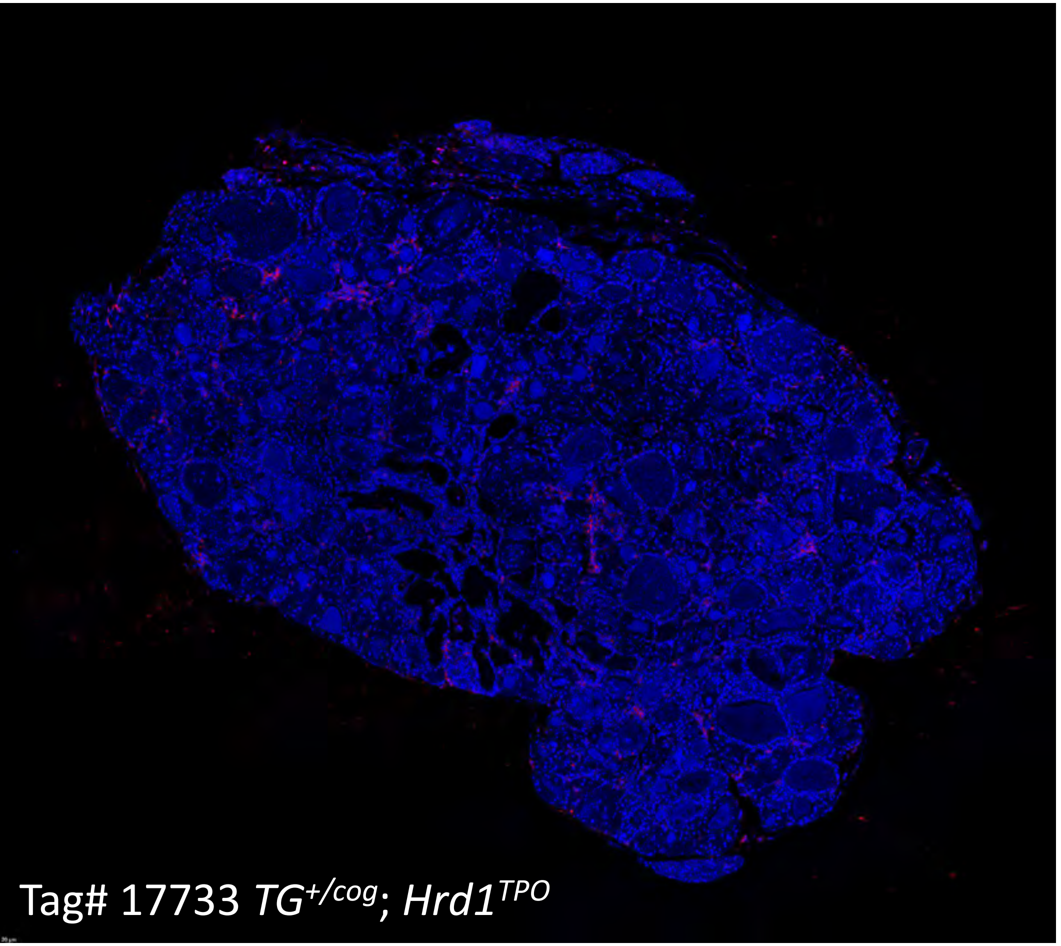

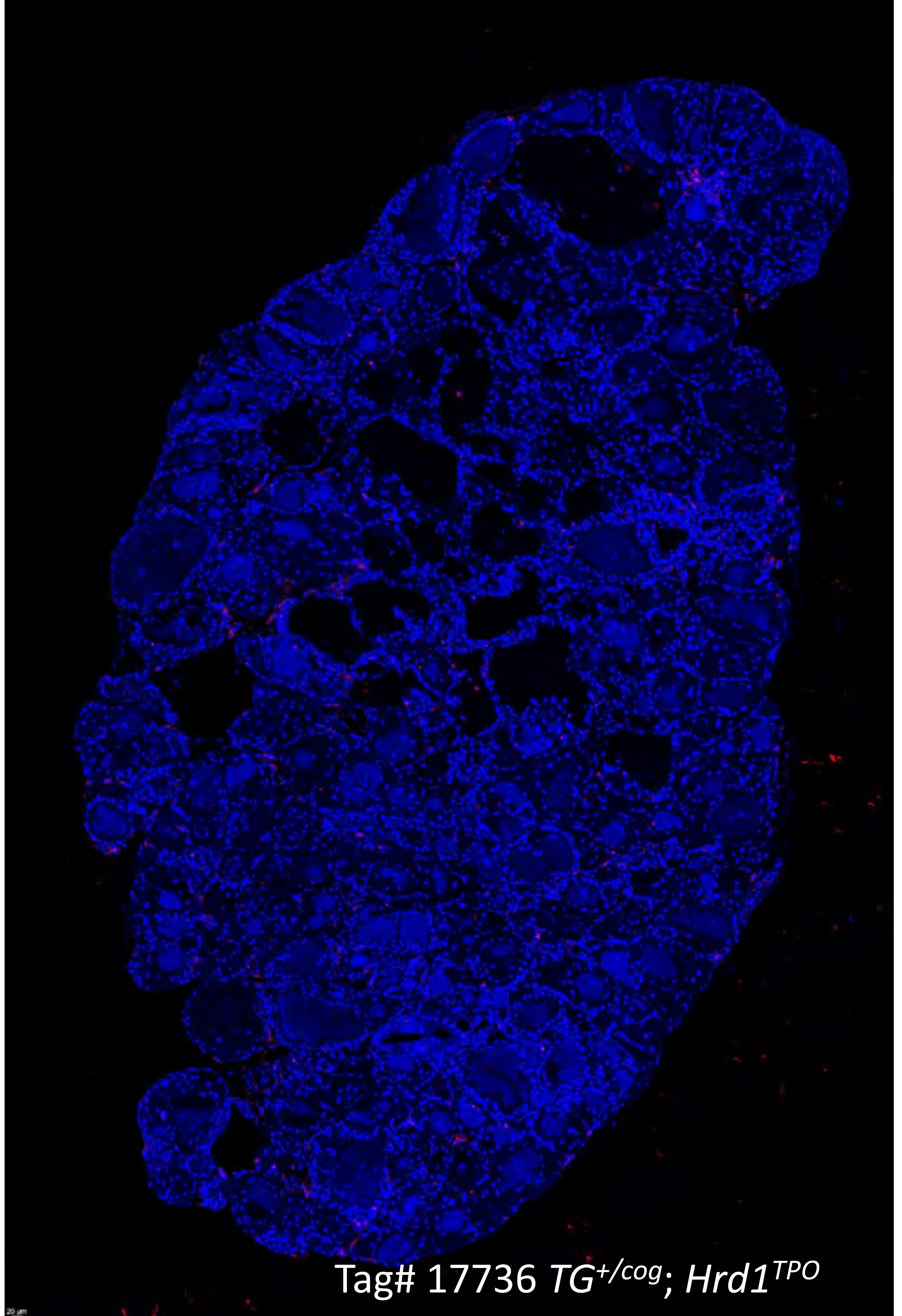

Fig. 7B

CD45 DAPI

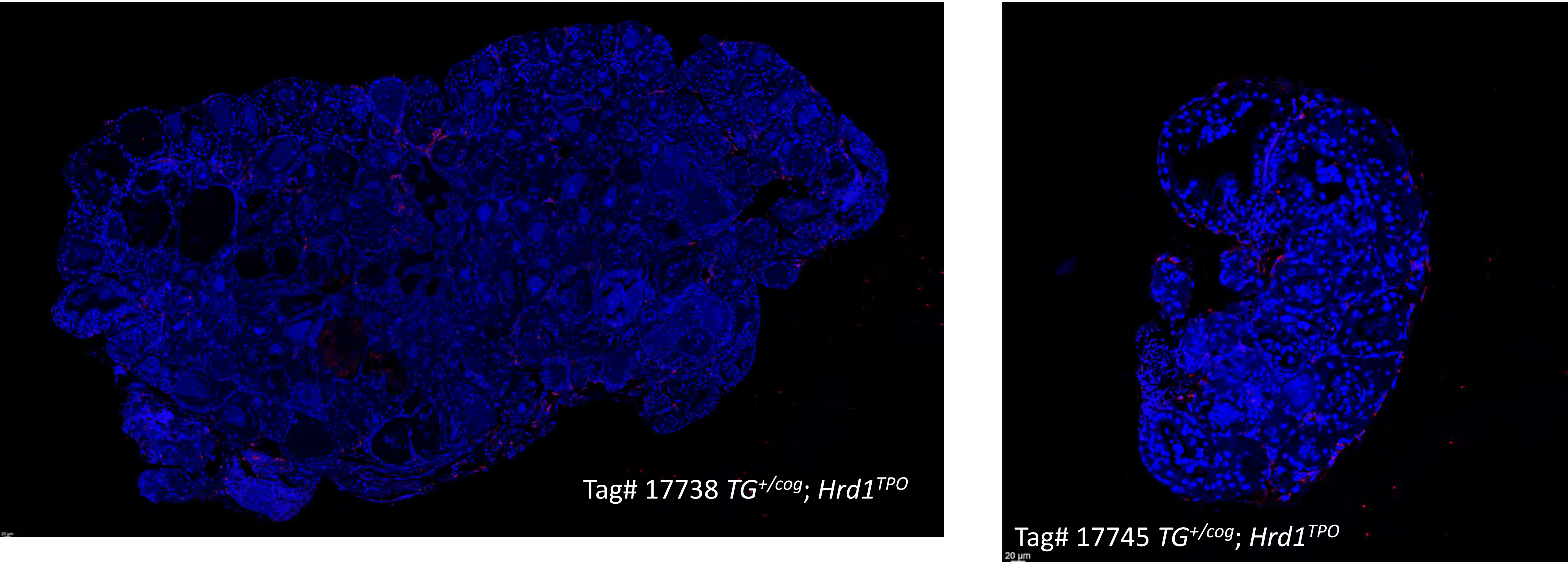

Fig. 8A Mac2 DAPI

$TG^{+/cog}$; $Hrd1^{control}$ Representative image

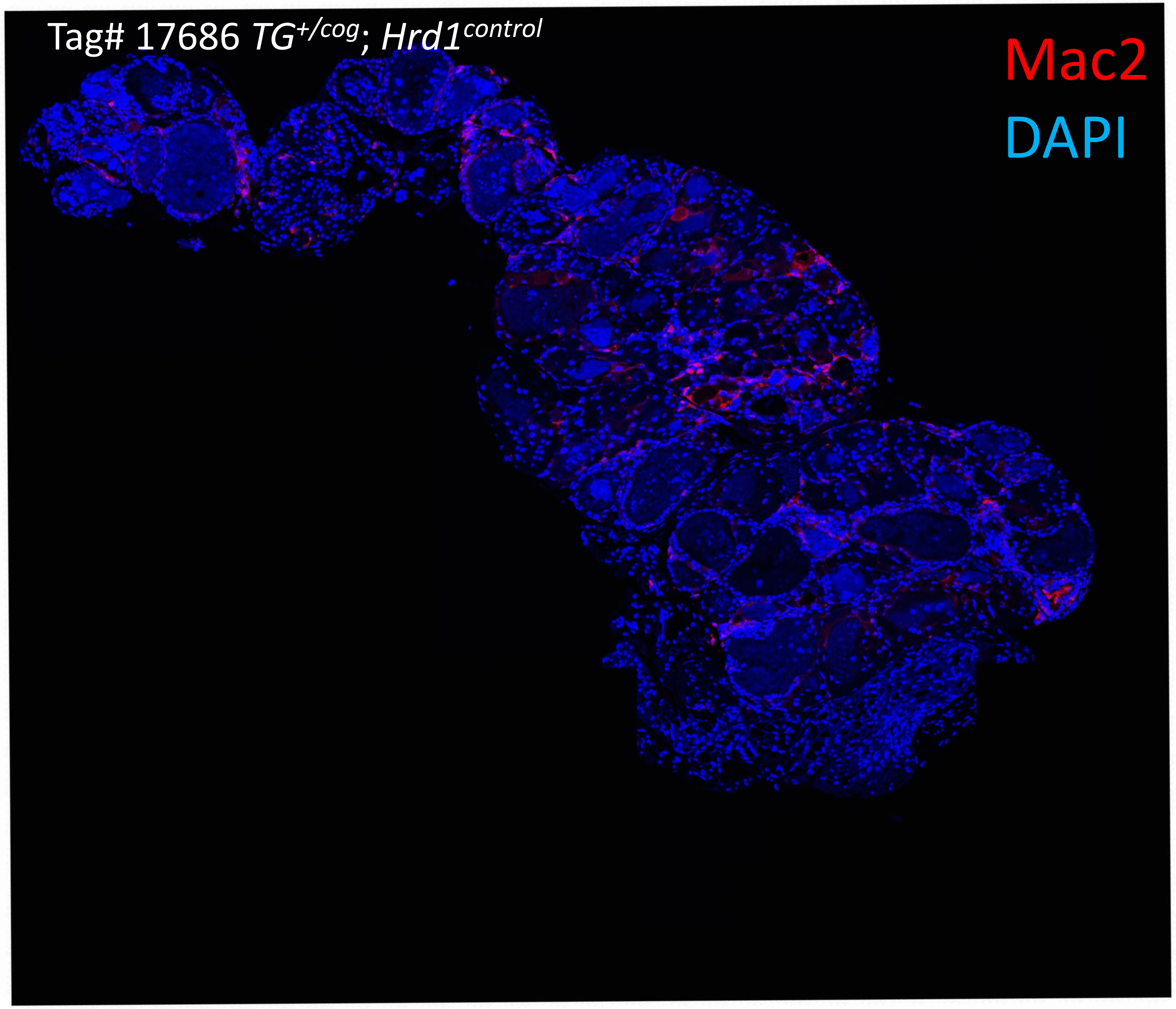

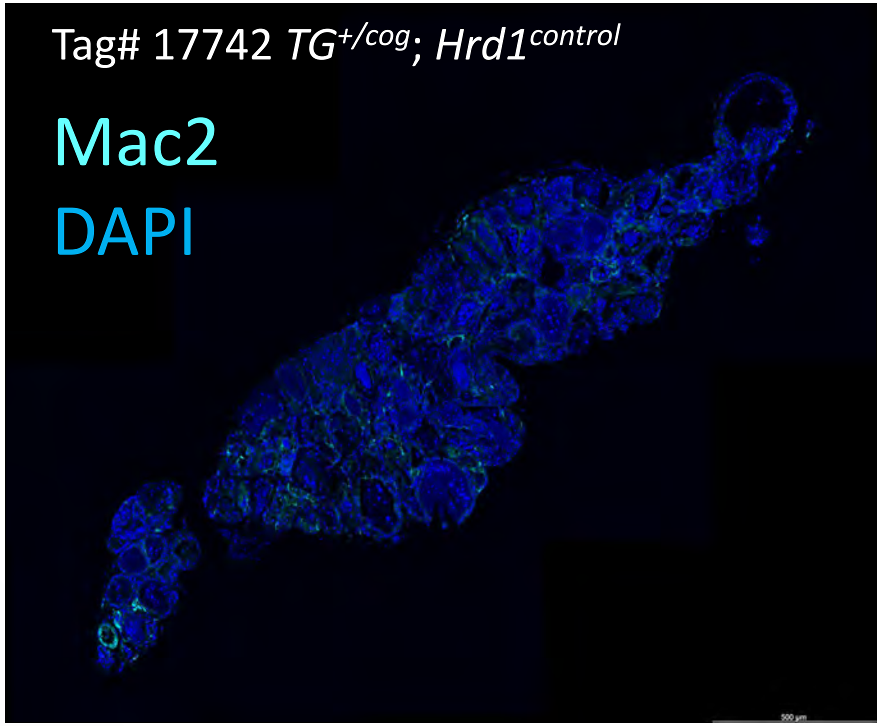

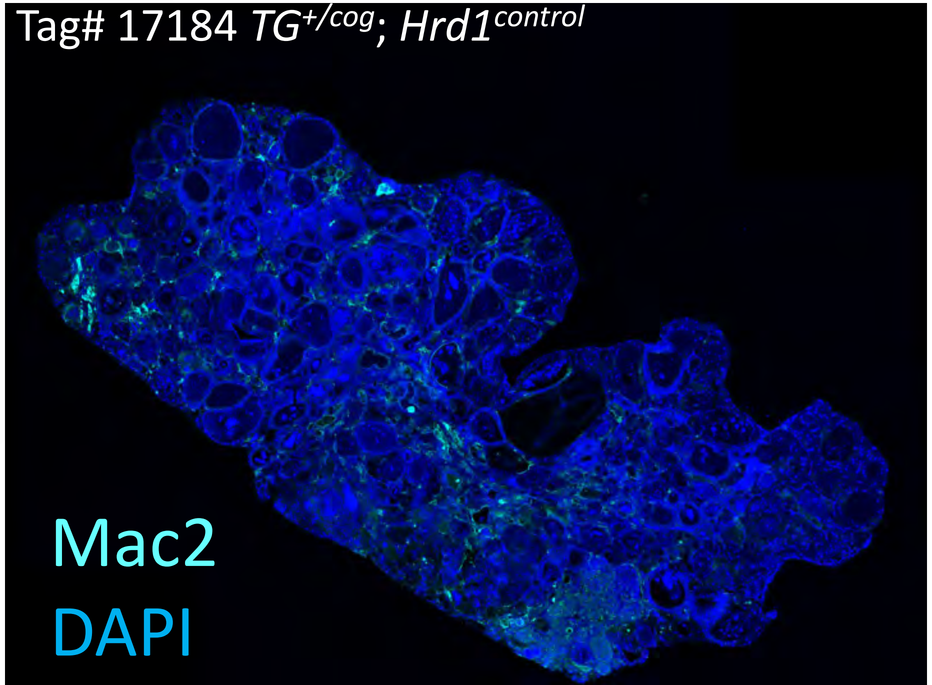

Fig. 8A

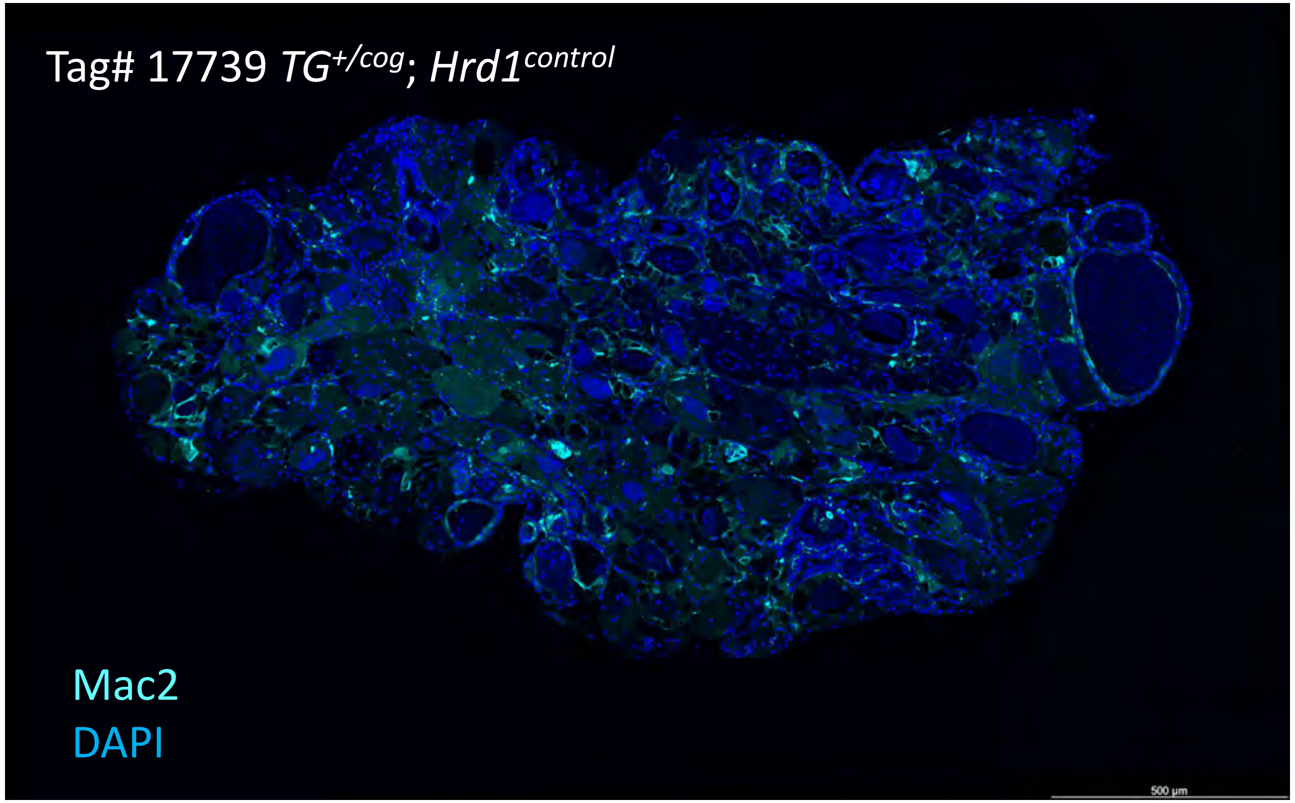
Tag# 17739 TG+/cog; Hrd1control
Mac2
DAPI
500 μm

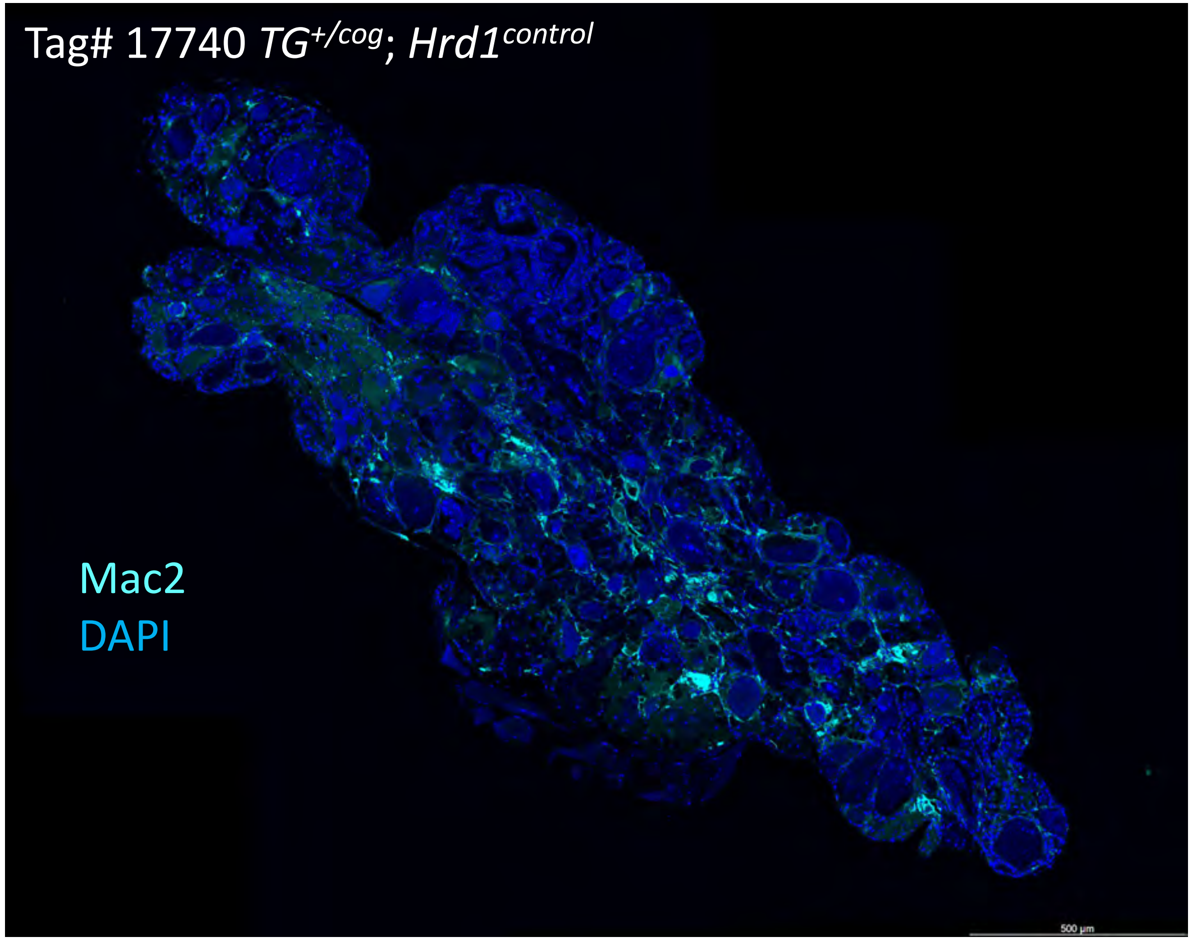
Tag# 17740 TG+/cog; Hrd1control
Mac2
DAPI
500 μm

Fig. 8A

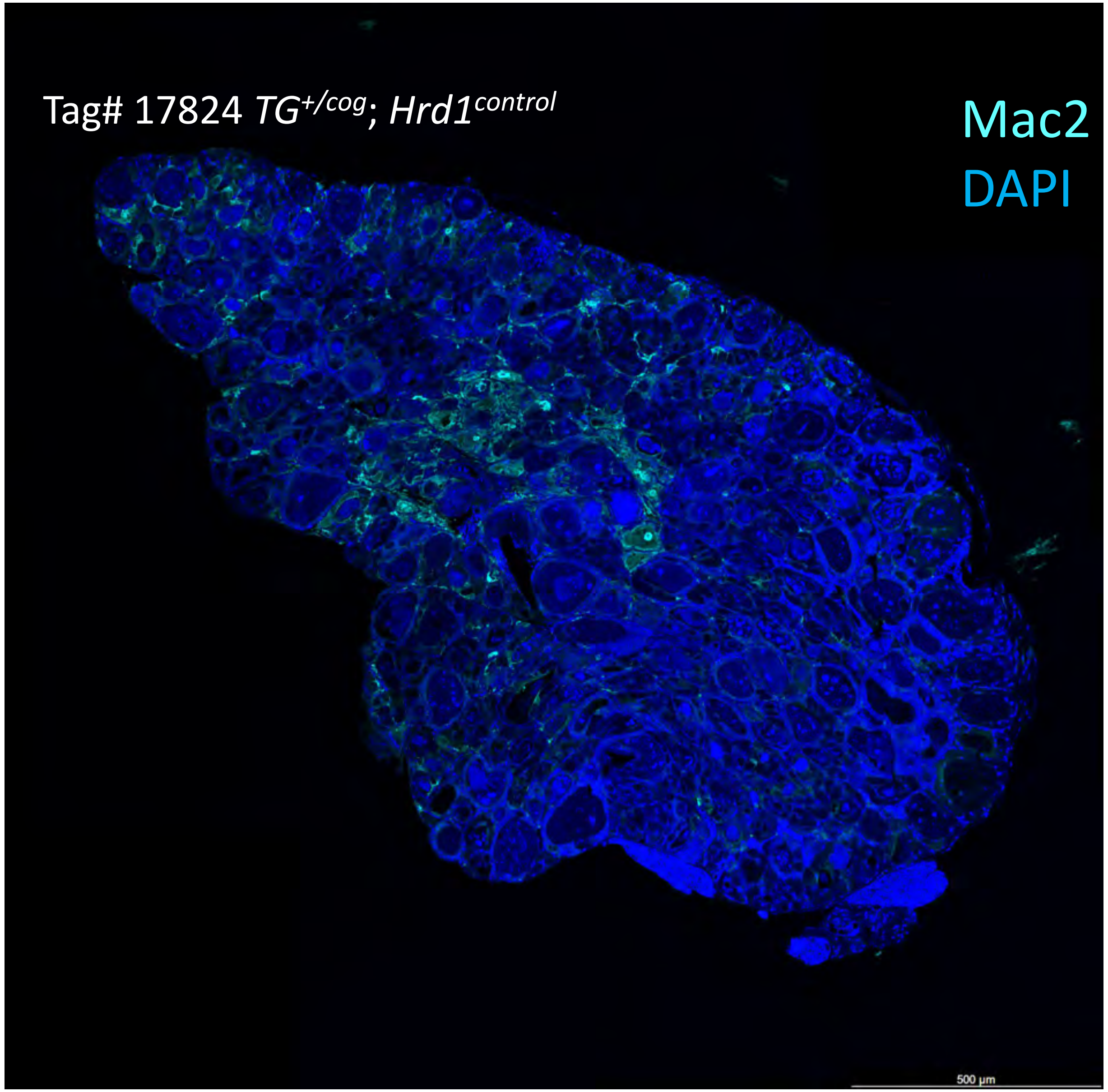
Tag# 17824 TG+/cog; Hrd1control
Mac2
DAPI
500 µm

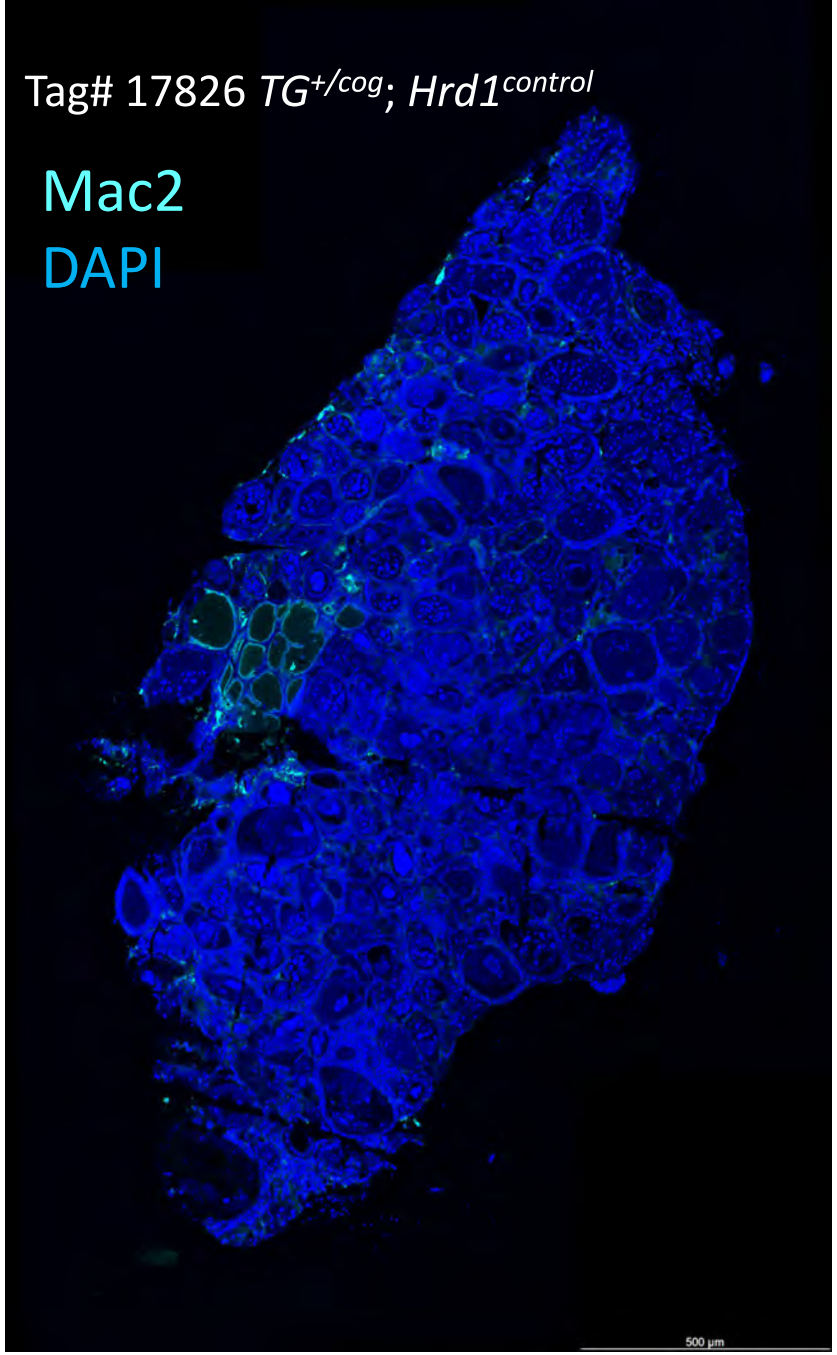
Tag# 17826 TG+/cog; Hrd1control
Mac2
DAPI
500 µm

Fig. 8A

TG$^{+/cog}$; *Hrd1*^{TPO}
Representative image

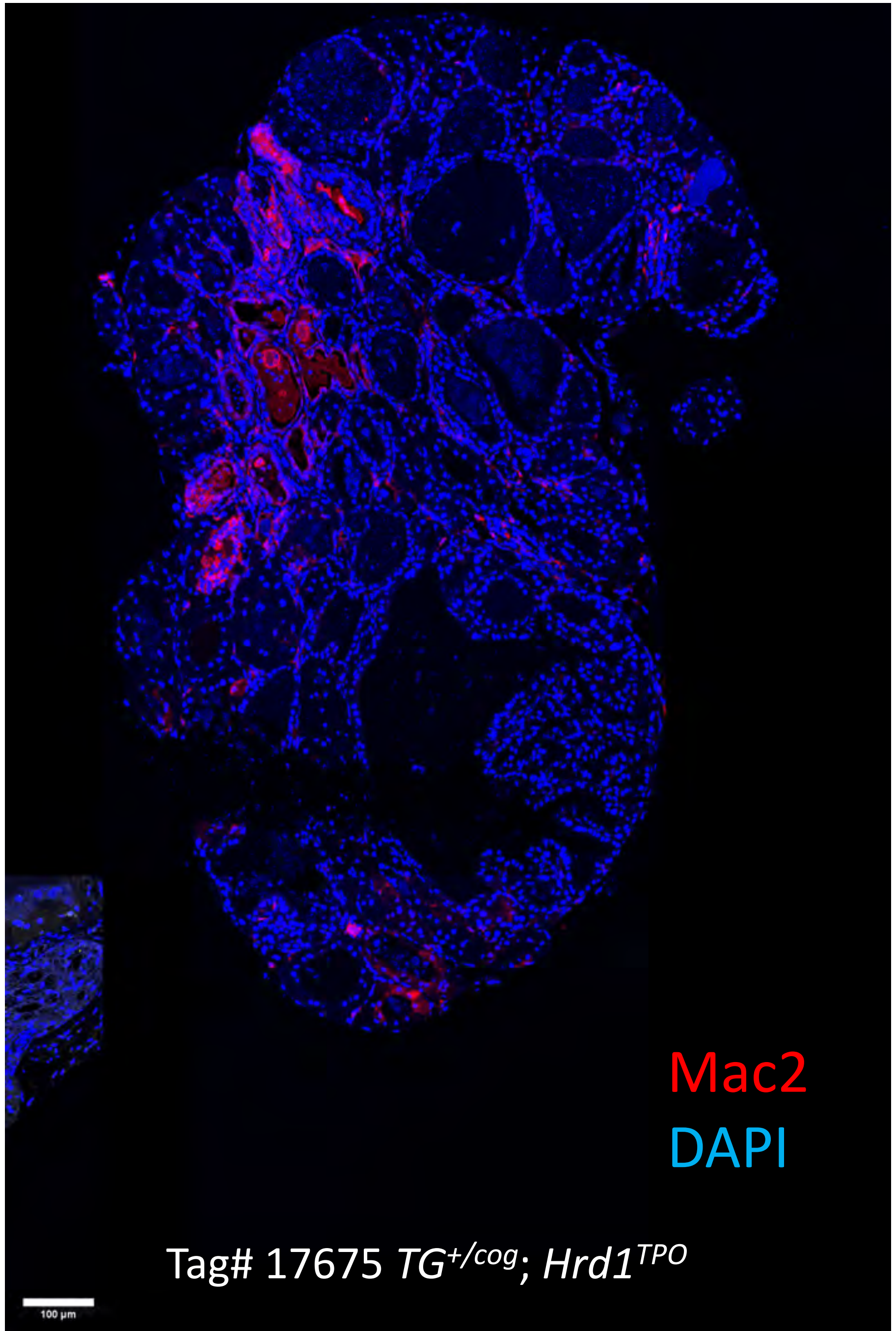

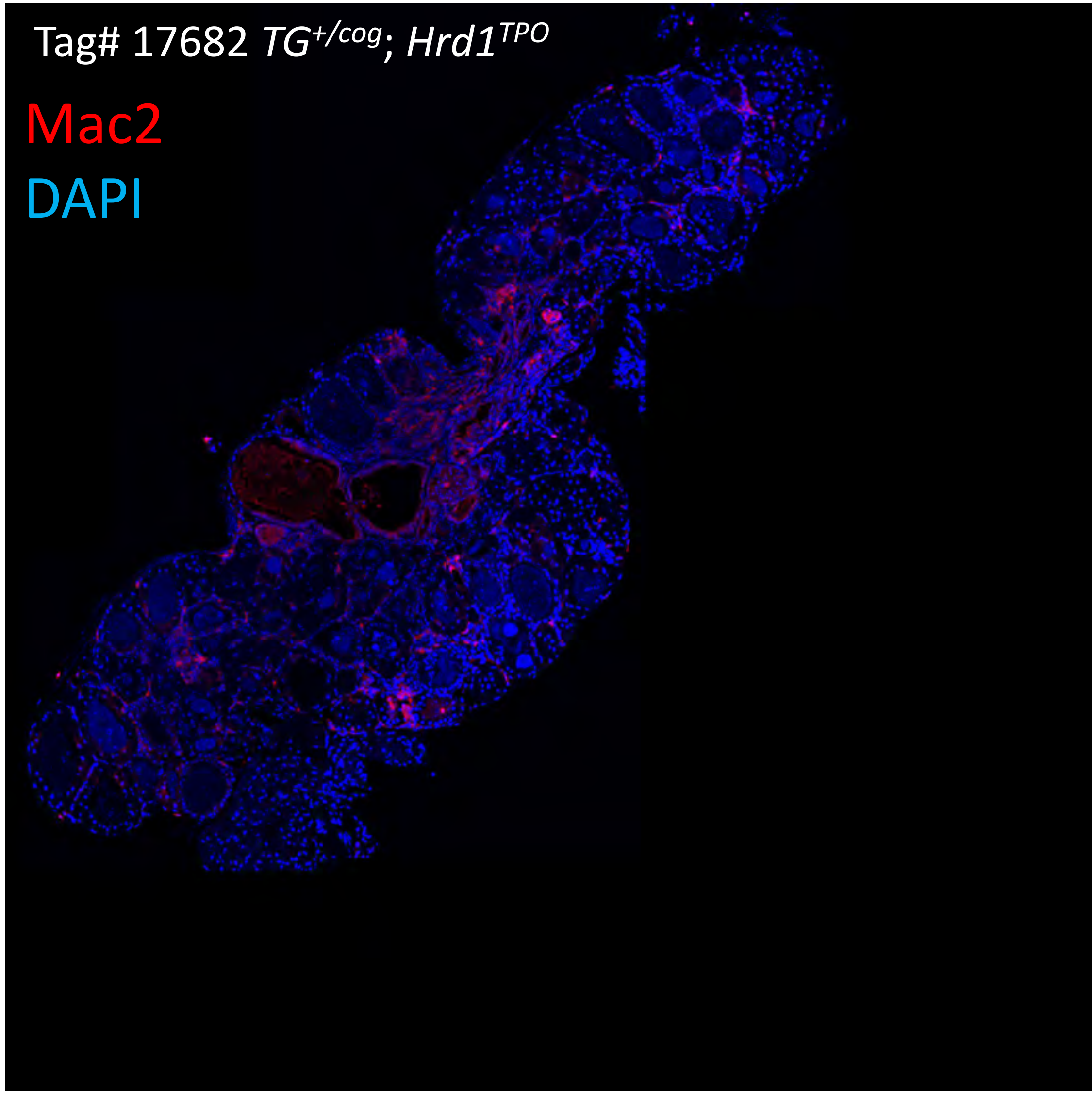

Fig. 8A

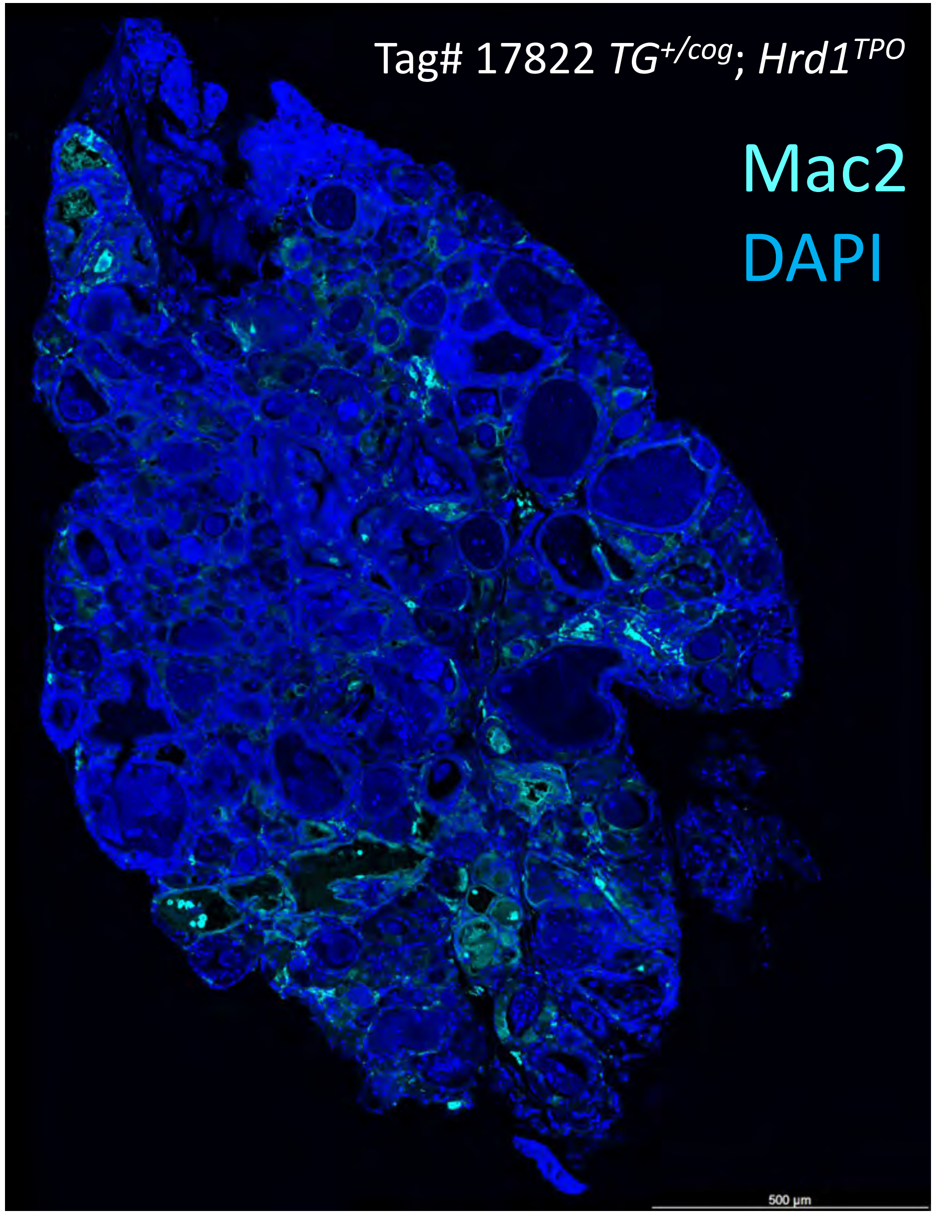
Tag# 17822 $TG^{+/cog}$; $Hrd1^{TPO}$
Mac2
DAPI
500 µm

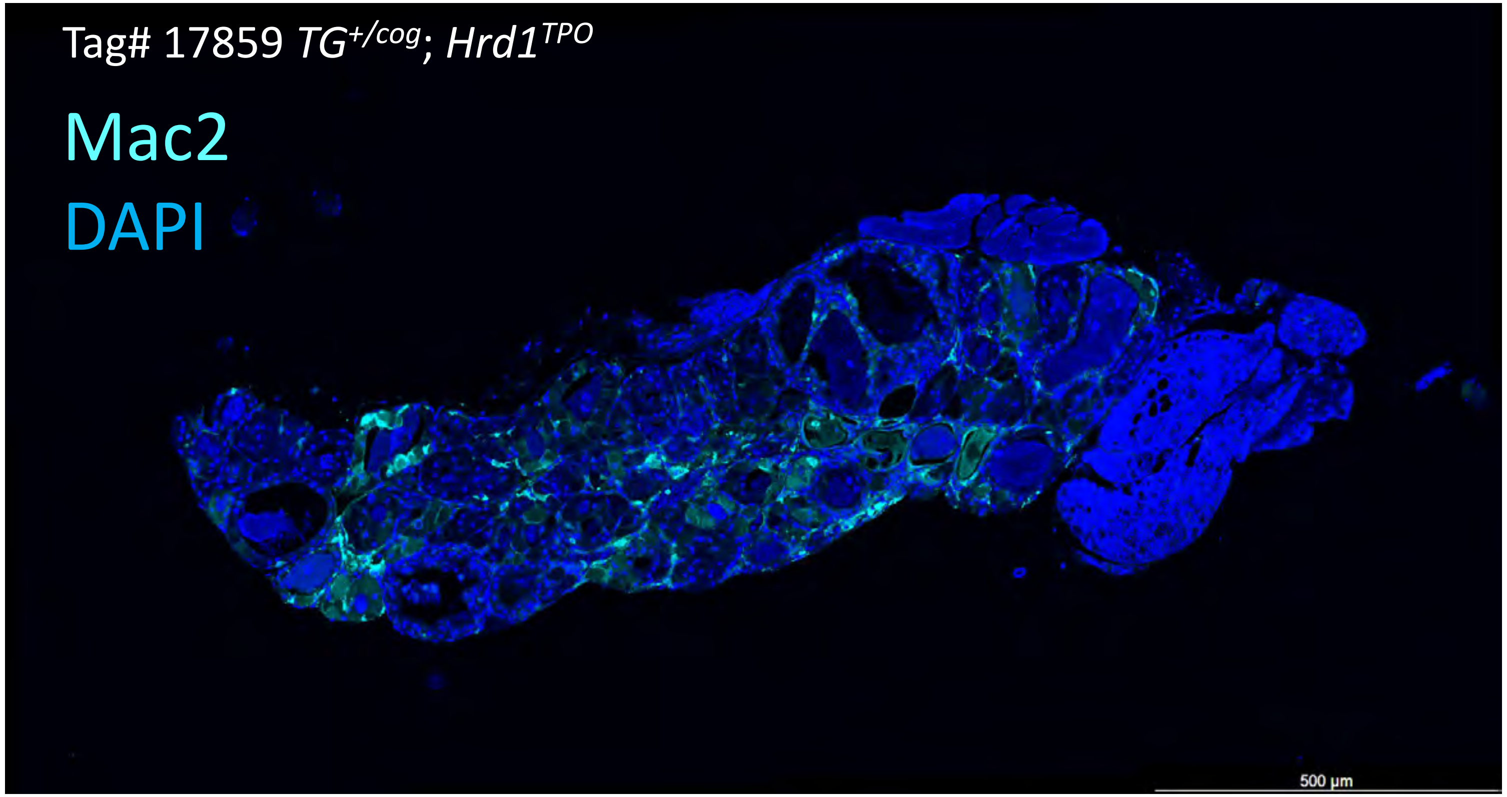
Tag# 17859 $TG^{+/cog}$; $Hrd1^{TPO}$
Mac2
DAPI
500 µm

Fig. 8A

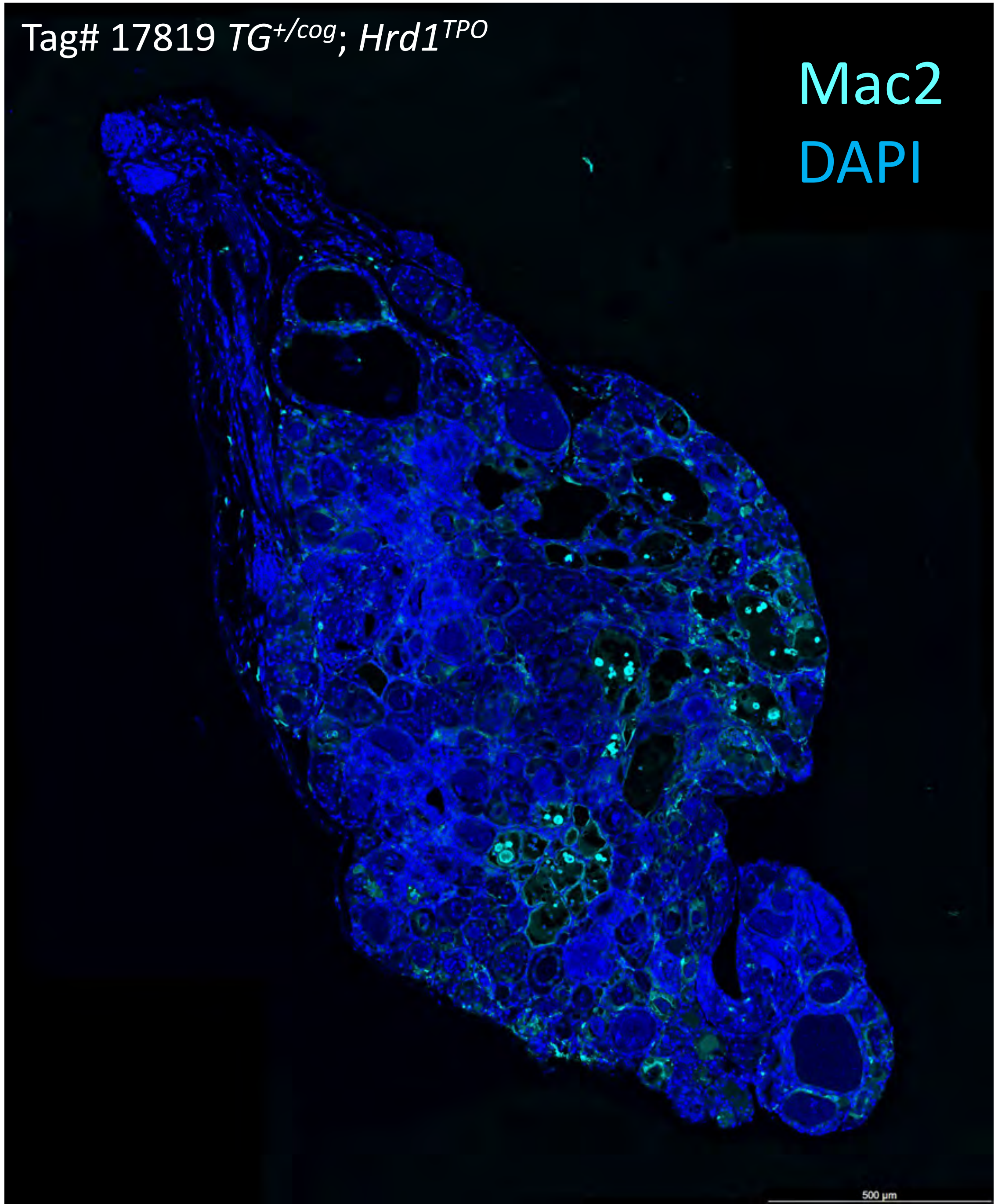

Quantitation

Tag Number	Genotype	Number Cells	# positive cells	% Mac 2 positive
17739	$TG^{+/cog}$; $Hrd1^{control}$	5858	56	0.9559576647
17740	$TG^{+/cog}$; $Hrd1^{control}$	6005	93	1.548709409
17742	$TG^{+/cog}$; $Hrd1^{control}$	4456	28	0.6283662478
17814	$TG^{+/cog}$; $Hrd1^{control}$	5923	43	0.7259834543
17819	$TG^{+/cog}$; $Hrd1^{TPO}$	7874	79	1.003302007
17822	$TG^{+/cog}$; $Hrd1^{TPO}$	6430	127	1.975116641
17824	$TG^{+/cog}$; $Hrd1^{control}$	4619	15	0.3247456159
17826	$TG^{+/cog}$; $Hrd1^{control}$	6432	16	0.2487562189
17859	$TG^{+/cog}$; $Hrd1^{TPO}$	2893	56	1.935706879
17682	$TG^{+/cog}$; $Hrd1^{TPO}$	4050	132	3.259259259
17675	$TG^{+/cog}$; $Hrd1^{TPO}$	5636	217	3.850248403
17686	$TG^{+/cog}$; $Hrd1^{control}$	2944	54	1.83423913

Fig. 8B

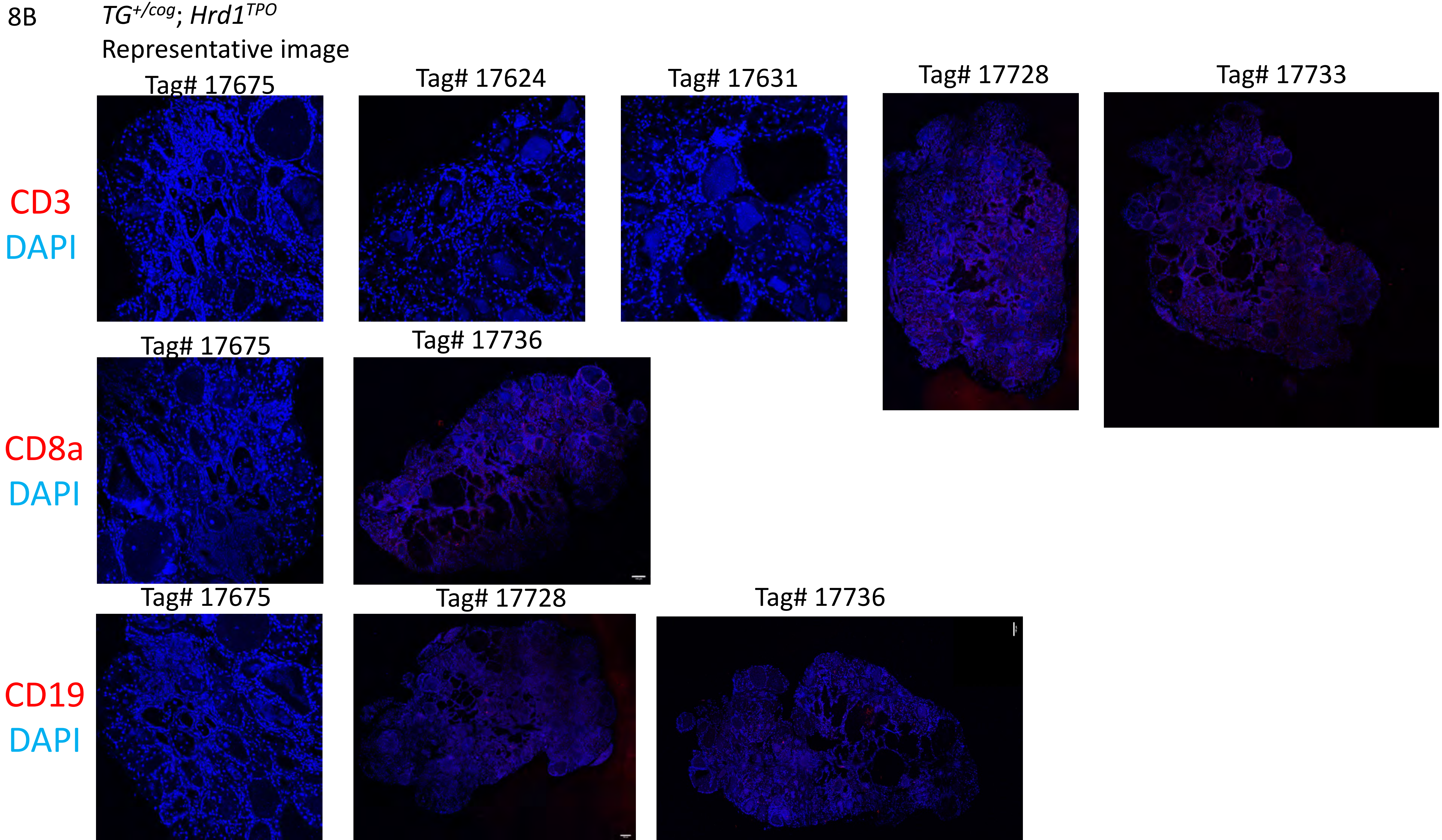
TG+/cog; Hrd1TPO
Representative image
Tag# 17675
Tag# 17624
Tag# 17631
Tag# 17728
Tag# 17733
CD3
DAPI
Tag# 17675
Tag# 17736
CD8a
DAPI
Tag# 17675
Tag# 17728
Tag# 17736
CD19
DAPI

Supplemental Fig. S1

Tag # 17174 *TG*$^{+/+}$

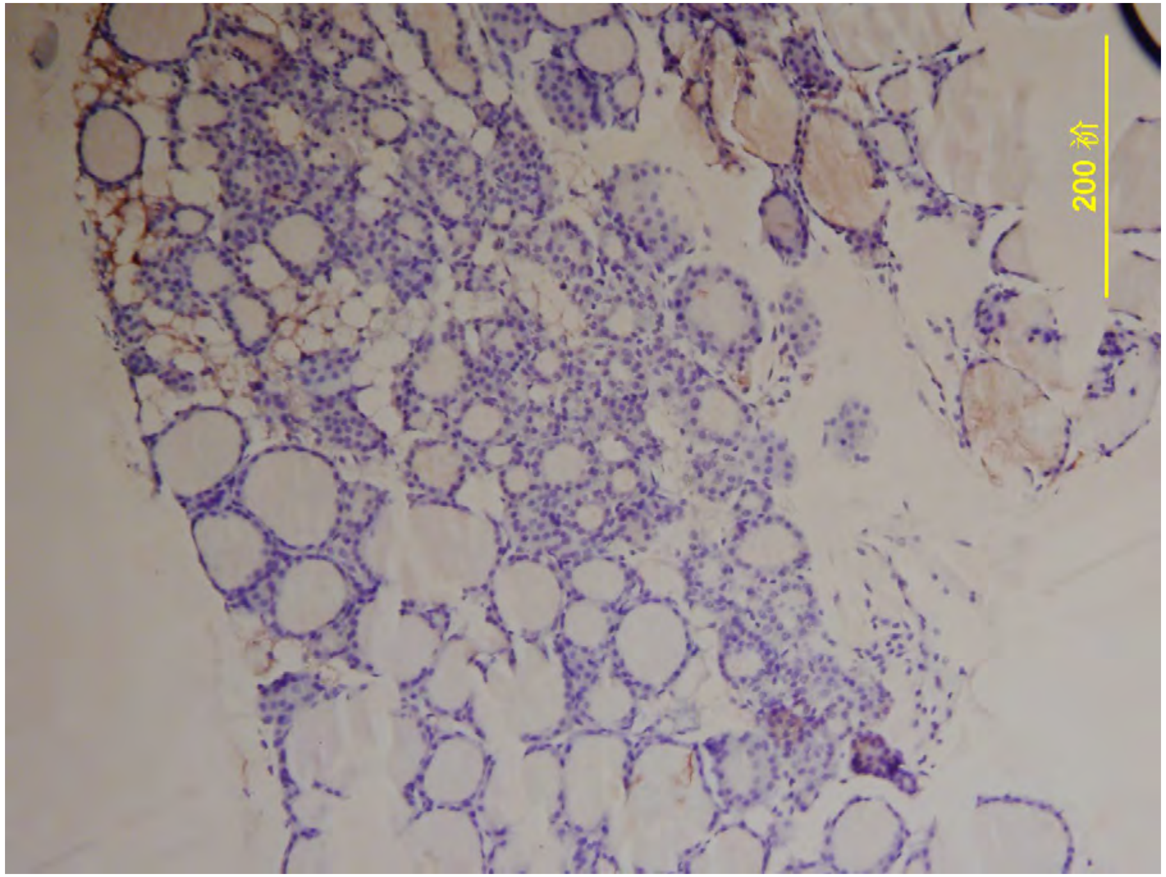

Tag # 17175 *TG*$^{+/+}$

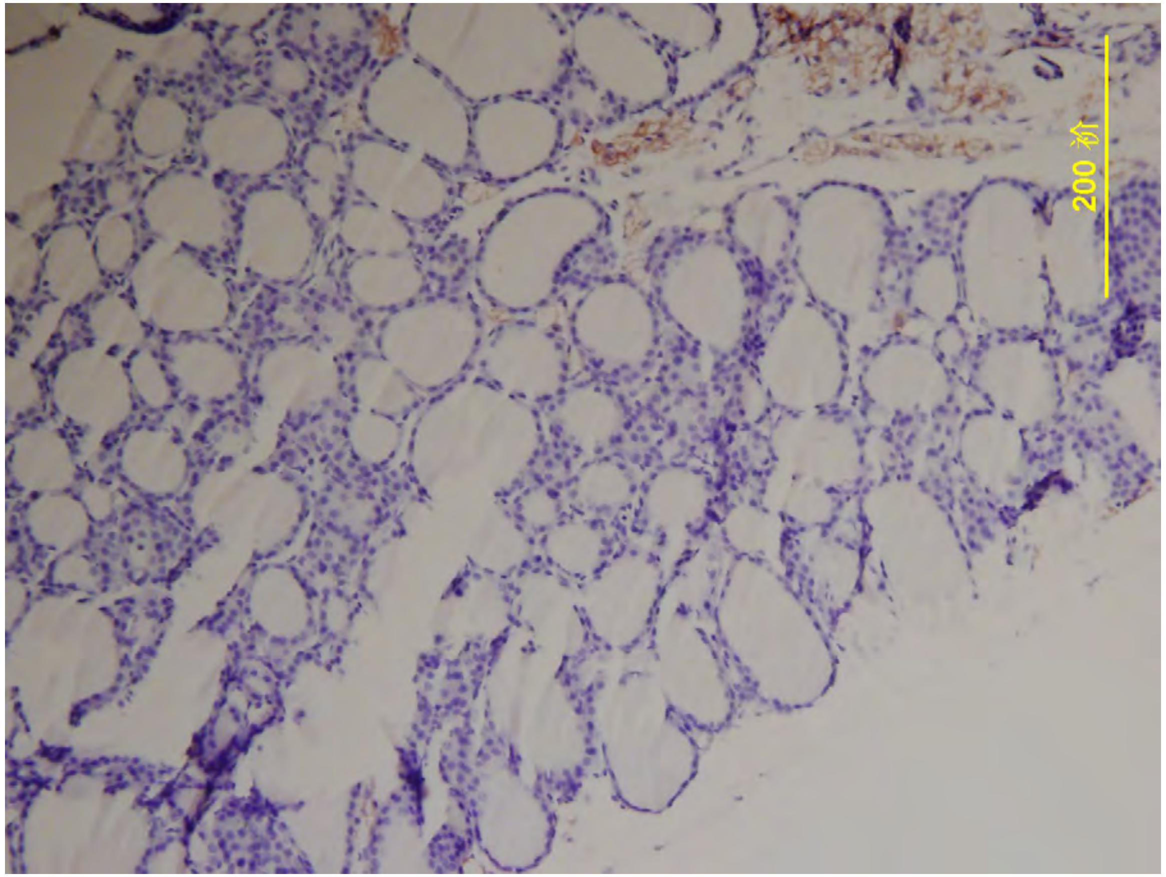

Supplemental Fig. S1

Tag # 17523 *TG*$^{+/cog}$

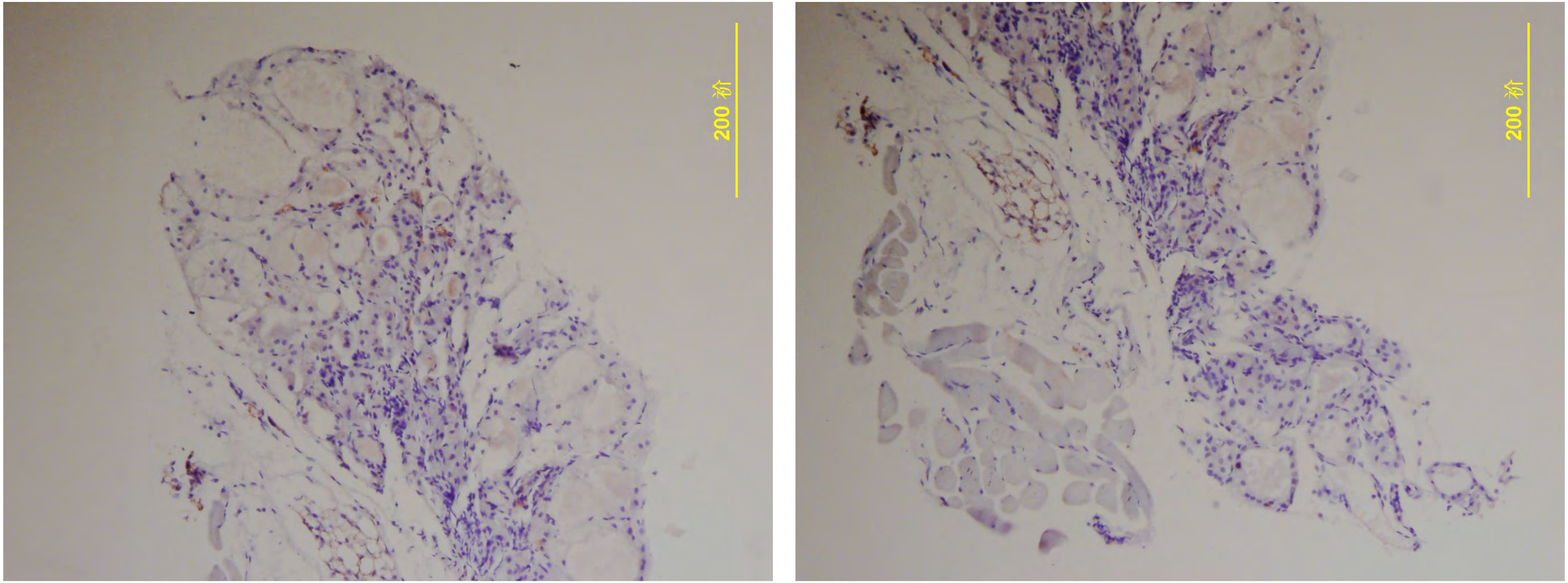

Supplemental Fig. S1

Tag # 17524 *TG*$^{+/cog}$

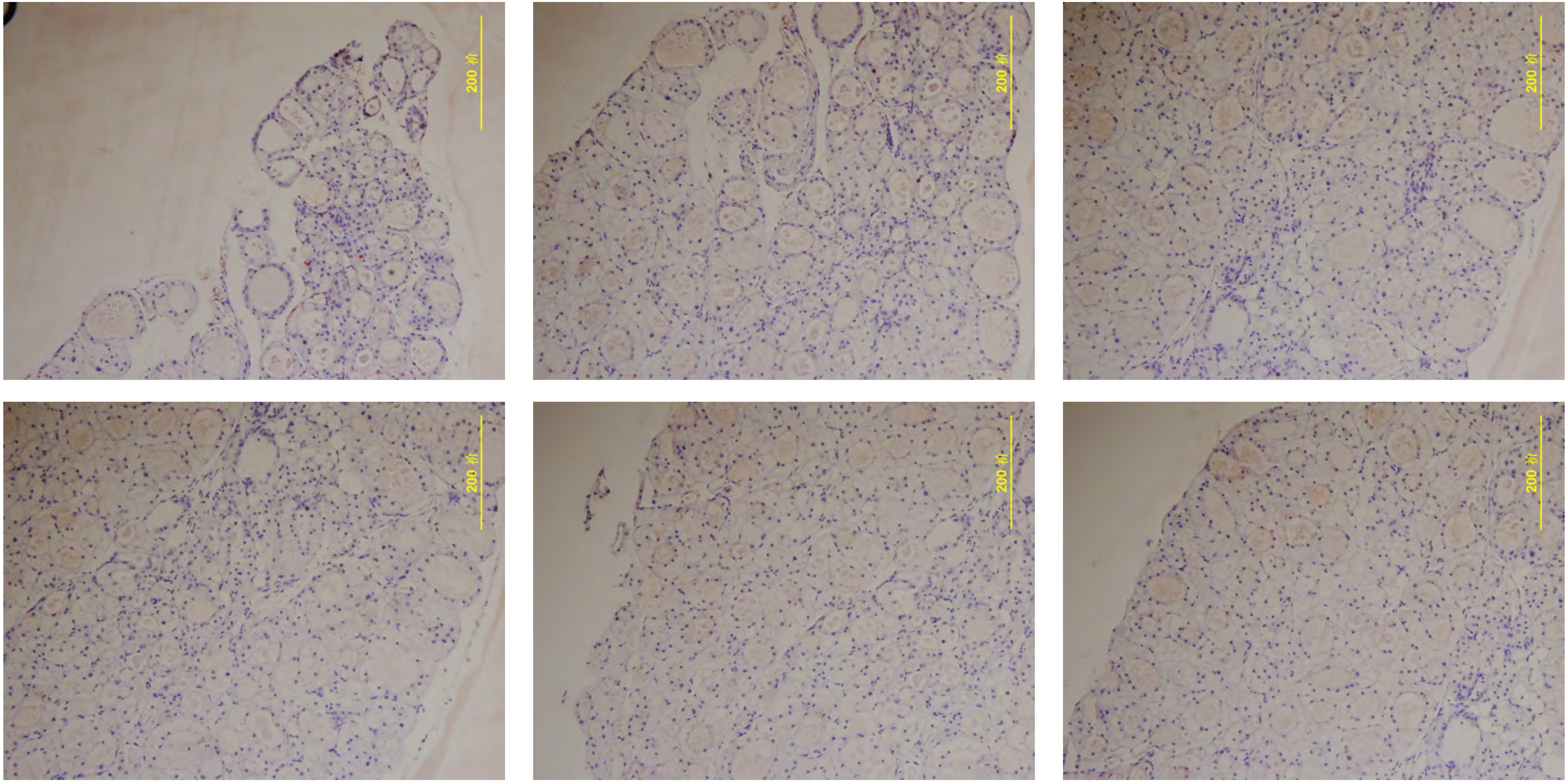

Supplemental Fig. S1

Tag # 17992 $TG^{+/cog}$ + T_4

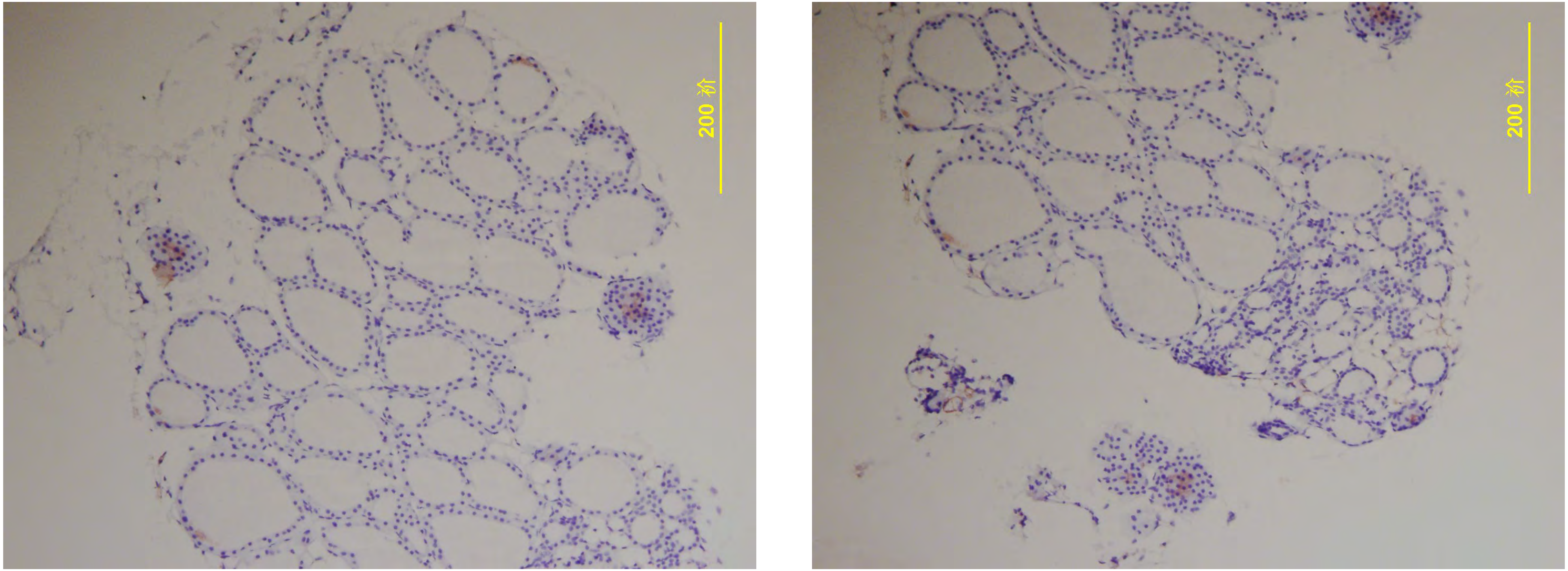

Fig S1

Tag # 17993 $\mathit{TG}^{+/cog}$ + T_4

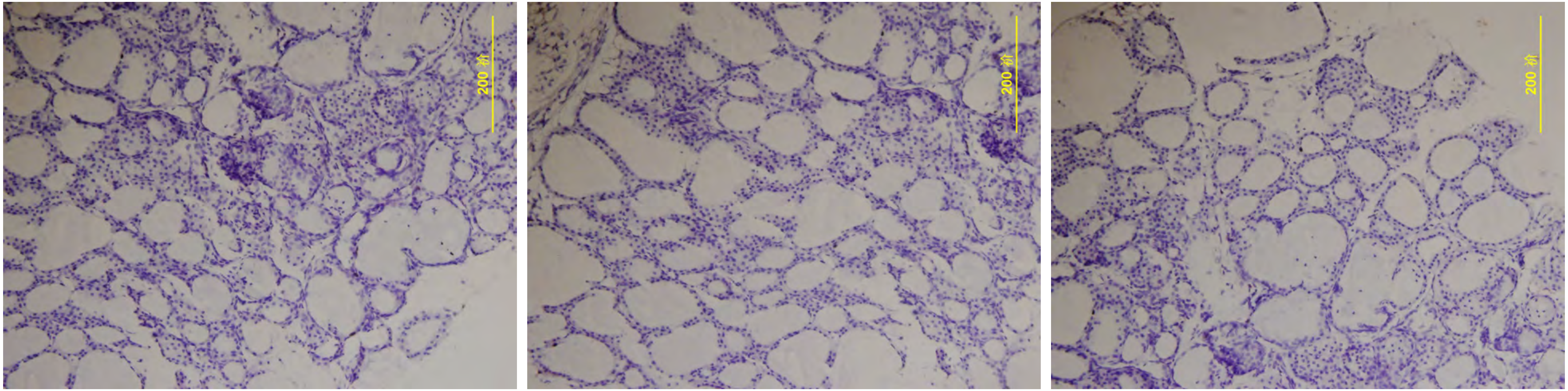

Fig S1

Tag # 17177 $TG^{+/+}$ | Tag # 17869 $TG^{+/+}$

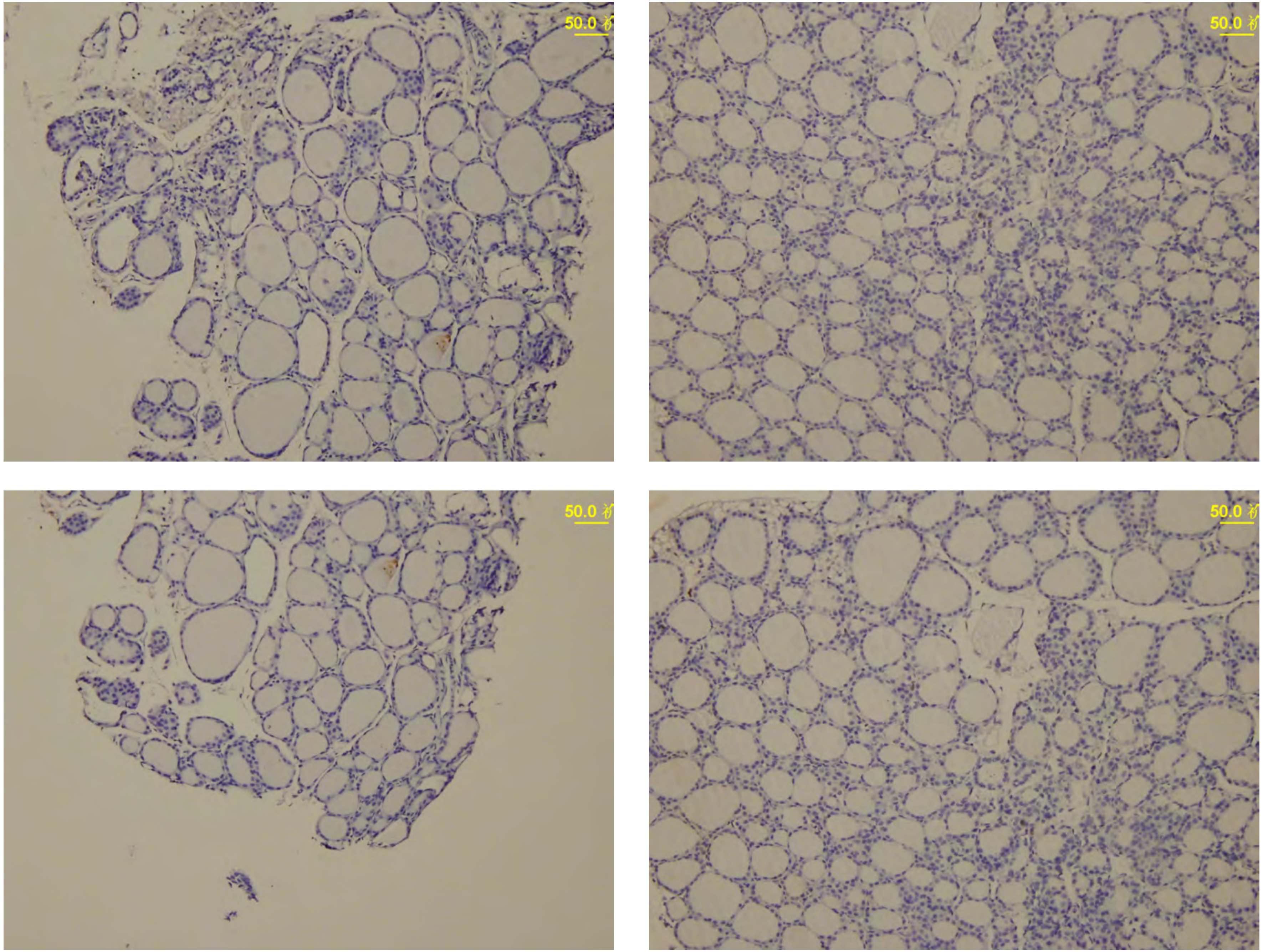

Fig S1

Tag # 17476 *TG*$^{+/cog}$

Tag # 17529 *TG*$^{+/cog}$

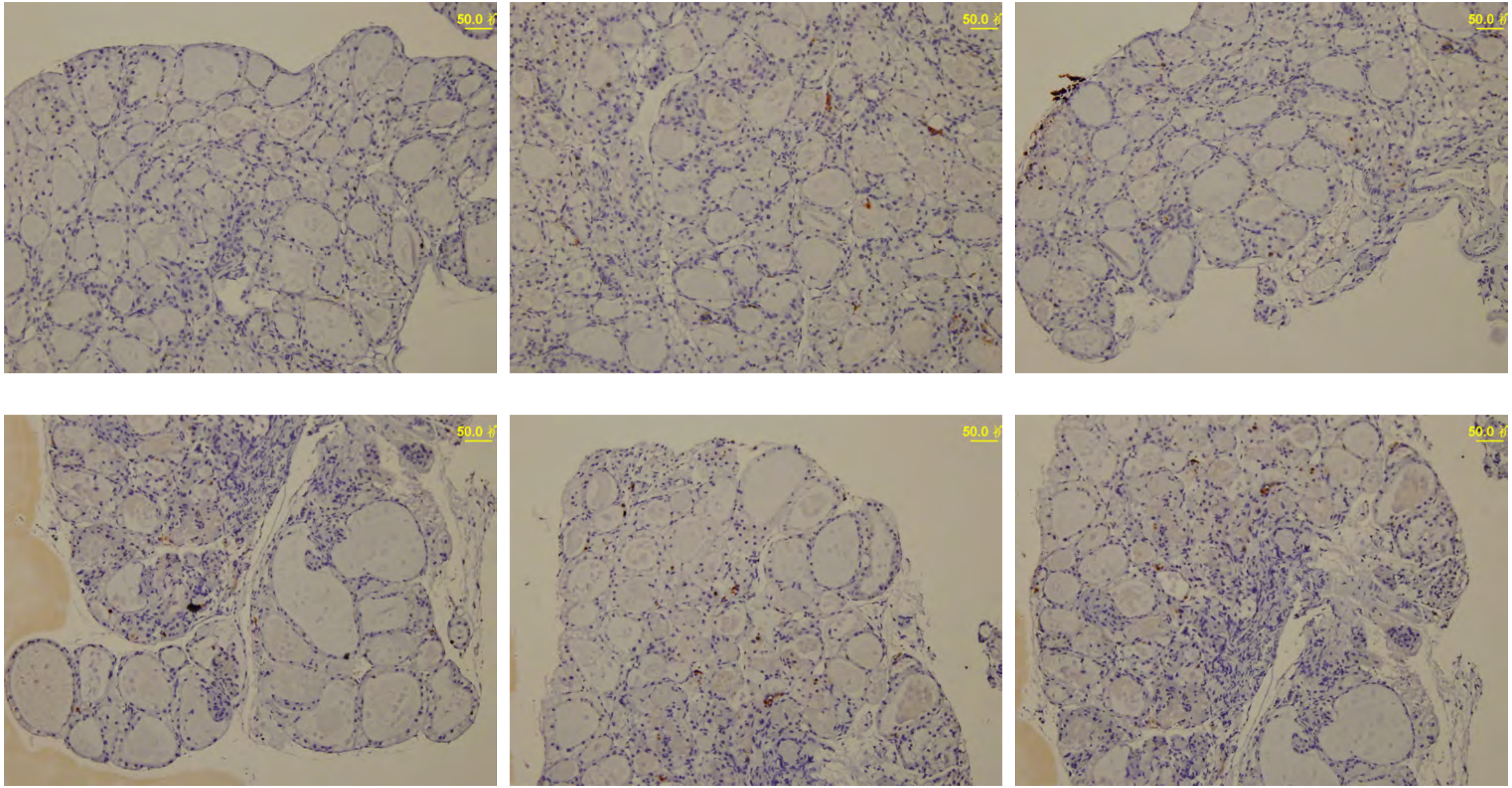

Fig S1

Tag # 17997 *TG*$^{+/cog}$ + T_4

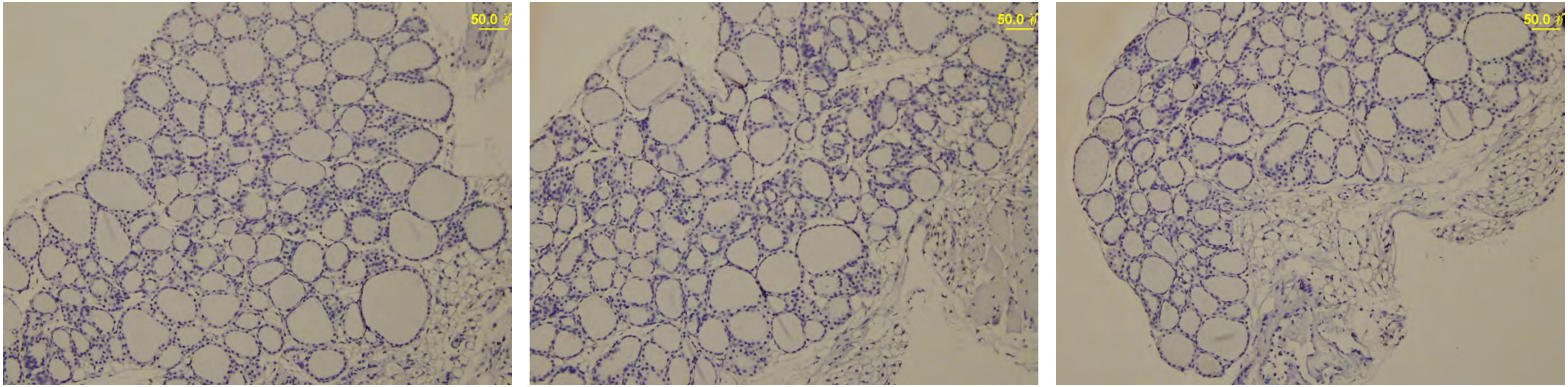

Fig S1

Tag # 17998 $\mathit{TG}^{+/cog}$ + T_4

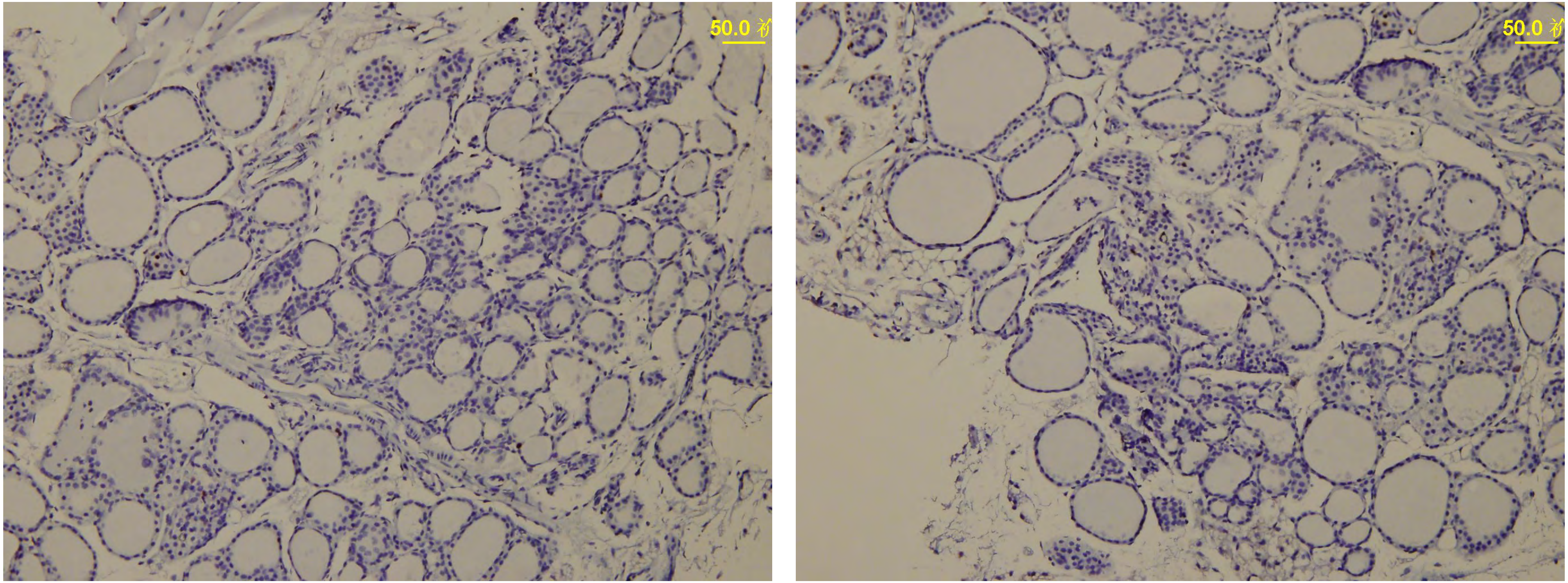

Fig S1

Quantitation

Tag Number	Genotype	x	# nuclei	# Ki67 positive nuclei	% Ki67 positive nuclei
17174	$TG^{+/+}$	1	2030	1	0.049261
17175	$TG^{+/+}$	1	1987	3	0.150981
17523	$TG^{+/cog}$	1	1513	6	0.396563
	$TG^{+/cog}$	2	1854	6	0.323625
17524	$TG^{+/cog}$	1	1430	3	0.20979
	$TG^{+/cog}$	2	1173	2	0.170503
	$TG^{+/cog}$	3	1267	6	0.47356
	$TG^{+/cog}$	4	1349	6	0.444774
	$TG^{+/cog}$	5	1391	9	0.647017
	$TG^{+/cog}$	6	875	6	0.685714
17992	$TG^{+/cog}$ + T_4	1	1197	0	0
	$TG^{+/cog}$ + T_4	2	1181	3	0.254022
17993	$TG^{+/cog}$ + T_4	1	2146	0	0
	$TG^{+/cog}$ + T_4	2	2344	4	0.170648
	$TG^{+/cog}$ + T_4	3	2215	0	0
17177	$TG^{+/+}$	1	1896	1	0.05274261603
	$TG^{+/+}$	2	1068	0	0
17869	$TG^{+/+}$	1	2406	4	0.1662510391
	$TG^{+/+}$	2	2356	5	0.2122241087
17476	$TG^{+/cog}$	1	1458	18	1.234567901
	$TG^{+/cog}$	2	1795	19	1.058495822
	$TG^{+/cog}$	3	1390	16	1.151079137
17529	$TG^{+/cog}$	1	1472	8	0.5434782609
	$TG^{+/cog}$	2	1233	14	1.135442011
	$TG^{+/cog}$	3	1772	12	0.6772009029
17997	$TG^{+/cog}$ + T_4	1	2212	1	0.0452079566
	$TG^{+/cog}$ + T_4	2	2275	1	0.04395604396
	$TG^{+/cog}$ + T_4	3	1785	1	0.05602240896
17998	$TG^{+/cog}$ + T_4	1	2465	5	0.2028397566
	$TG^{+/cog}$ + T_4	2	2246	4	0.17809439

Supplemental Fig. S2

Experiment #4 #5 shown in the figure:

Lane 3,7: vehicle
Lane 4,8: CB5083

Lane: 1 2 3 4 5 6 7 8

Tg

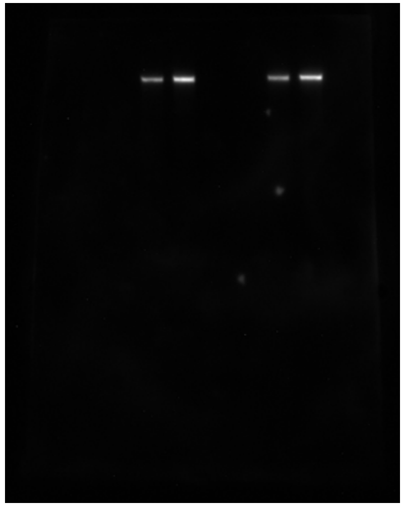

Brightfield image

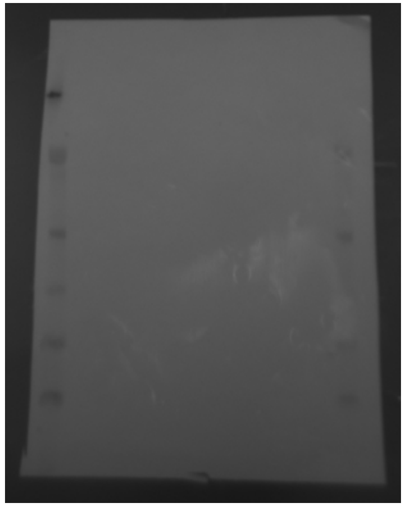

Lane: 1 2 3 4 5 6 7 8

Actin

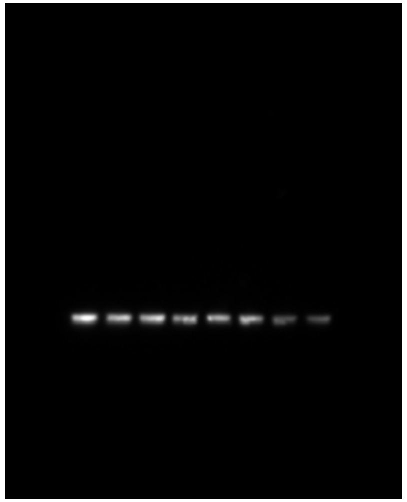

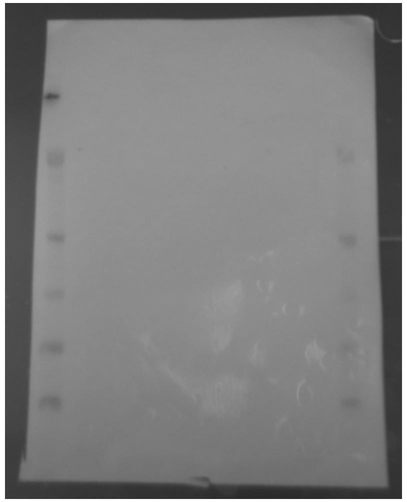

Quantification:

Exp#1				Tg/Actin	Fold change
	Tg	vehicle	17338642	1.064996	1
		cb5083	35988264	2.093706	1.965929
	actin	vehicle	16280480		
		cb5083	17188784		
Exp#2				Tg/Actin	Fold change
	Tg	vehicle	26085524	1.14713	1
		cb5083	44745251	1.979971	1.726022
	actin	vehicle	22739824		
		cb5083	22598944		
Exp#3				Tg/Actin	Fold change
	Tg	vehicle	6346736	0.293385	1
		cb5083	6508688	0.287188	0.978877
	actin	vehicle	21632784		
		cb5083	22663520		
Exp#4				Tg/Actin	Fold change
	Tg	vehicle	8930864	0.408222	1
		cb5083	15600896	0.912386	2.235024
	Actin	vehicle	21877472		
		cb5083	17099008		
Exp#5				Tg/Actin	Fold change
	Tg	vehicle	9777632	1.086892	1
		cb5083	16128400	2.153775	1.98159
	Actin	vehicle	8995952		
		cb5083	7488432		

Lane 1: vehicle
Lane 2: CB5083

Experiment #1:

Lane: 1 2

Tg

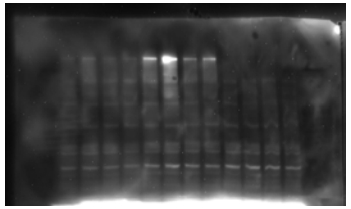

Brightfield image

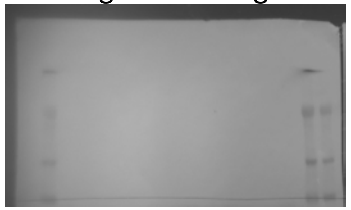

Lane: 1 2

Actin

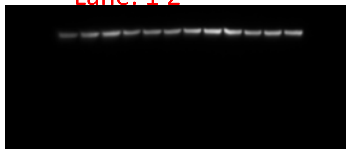

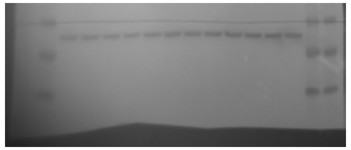

Experiment #2:

Lane: 1 2

Tg

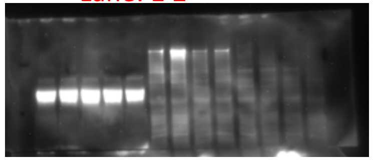

Brightfield image

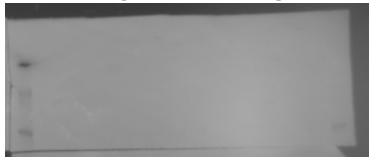

Lane: 1 2

Actin

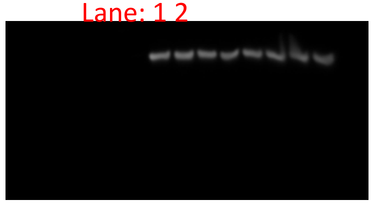

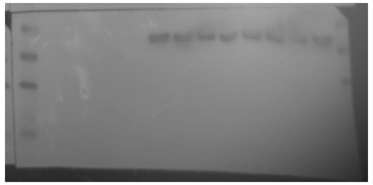

Experiment #3:

Lane: 1 2

Tg

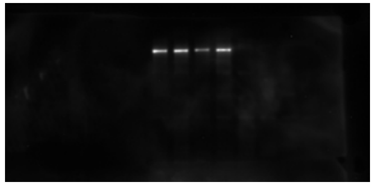

Brightfield image

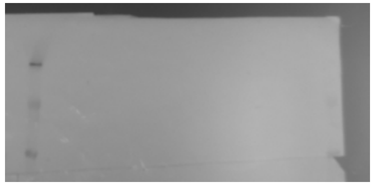

Lane: 1 2

Actin

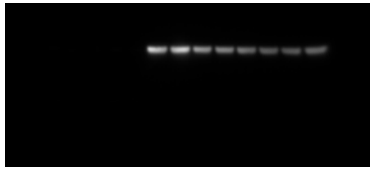

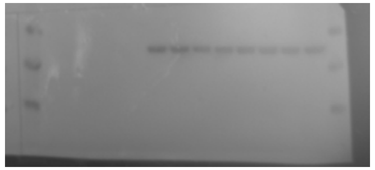

Supplemental Fig. S3

Tag# 17739 $TG^{+/cog}$; $Hrd1^{control}$

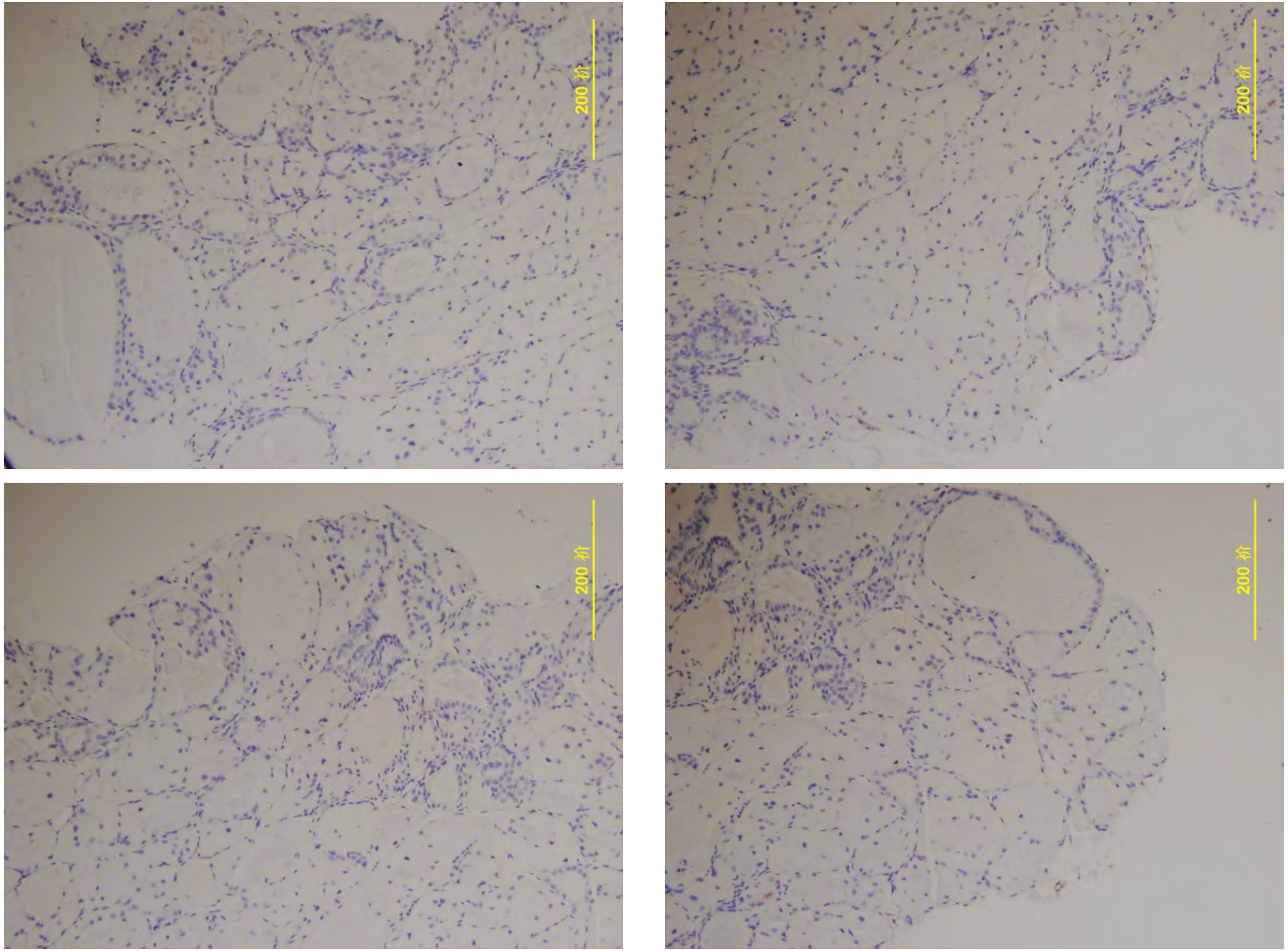

Supplemental Fig. S3

Tag# 17740 $TG^{+/cog}$; $Hrd1^{control}$

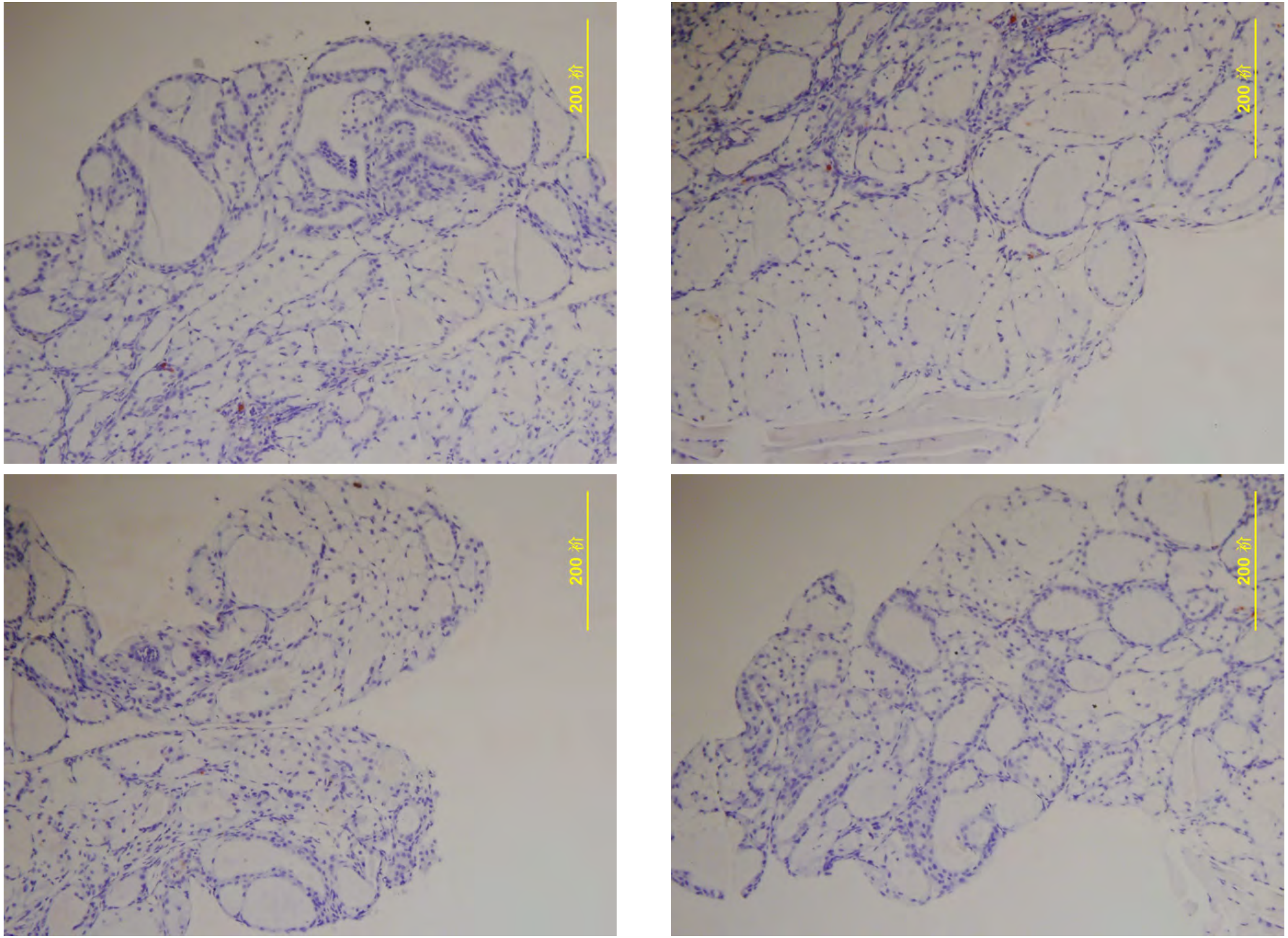

Supplemental Fig. S3

Tag# 17742 $TG^{+/cog}$; $Hrd1^{control}$

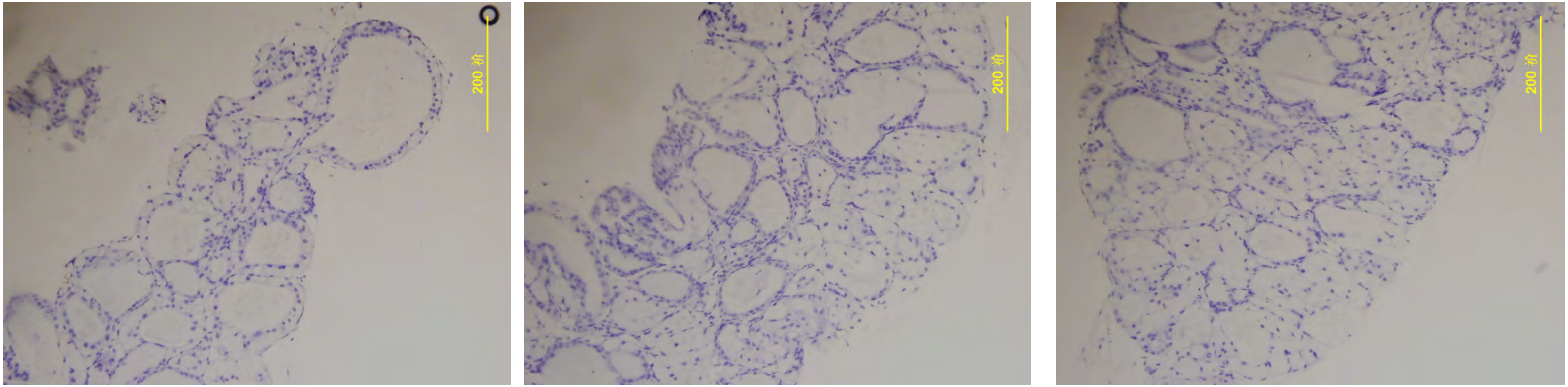

Supplemental Fig. S3

Tag# 17745 $TG^{+/cog}$; $Hrd1^{TPO}$

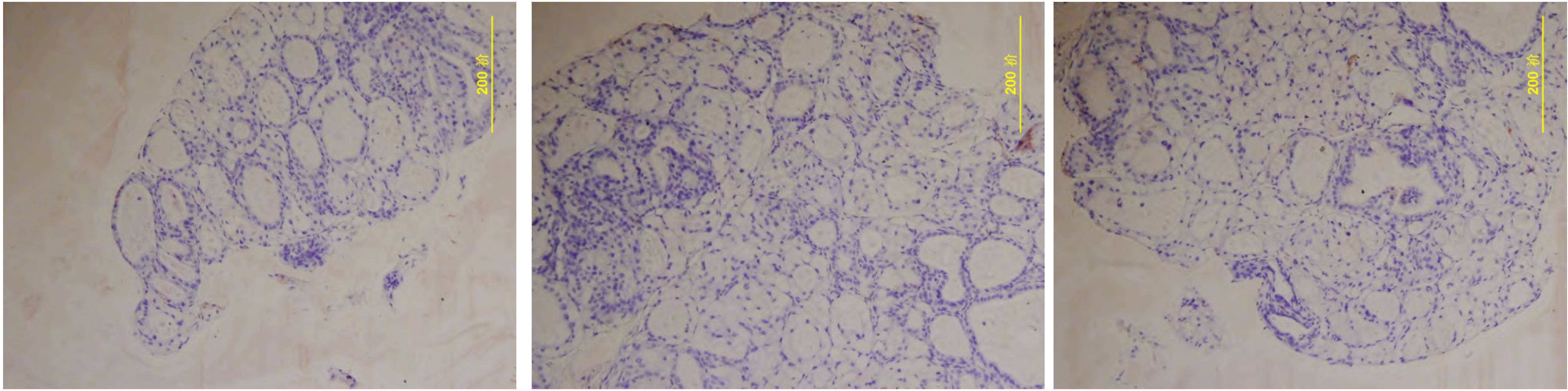

Supplemental Fig. S3

Tag# 17760 $TG^{+/cog}$; $Hrd1^{TPO}$

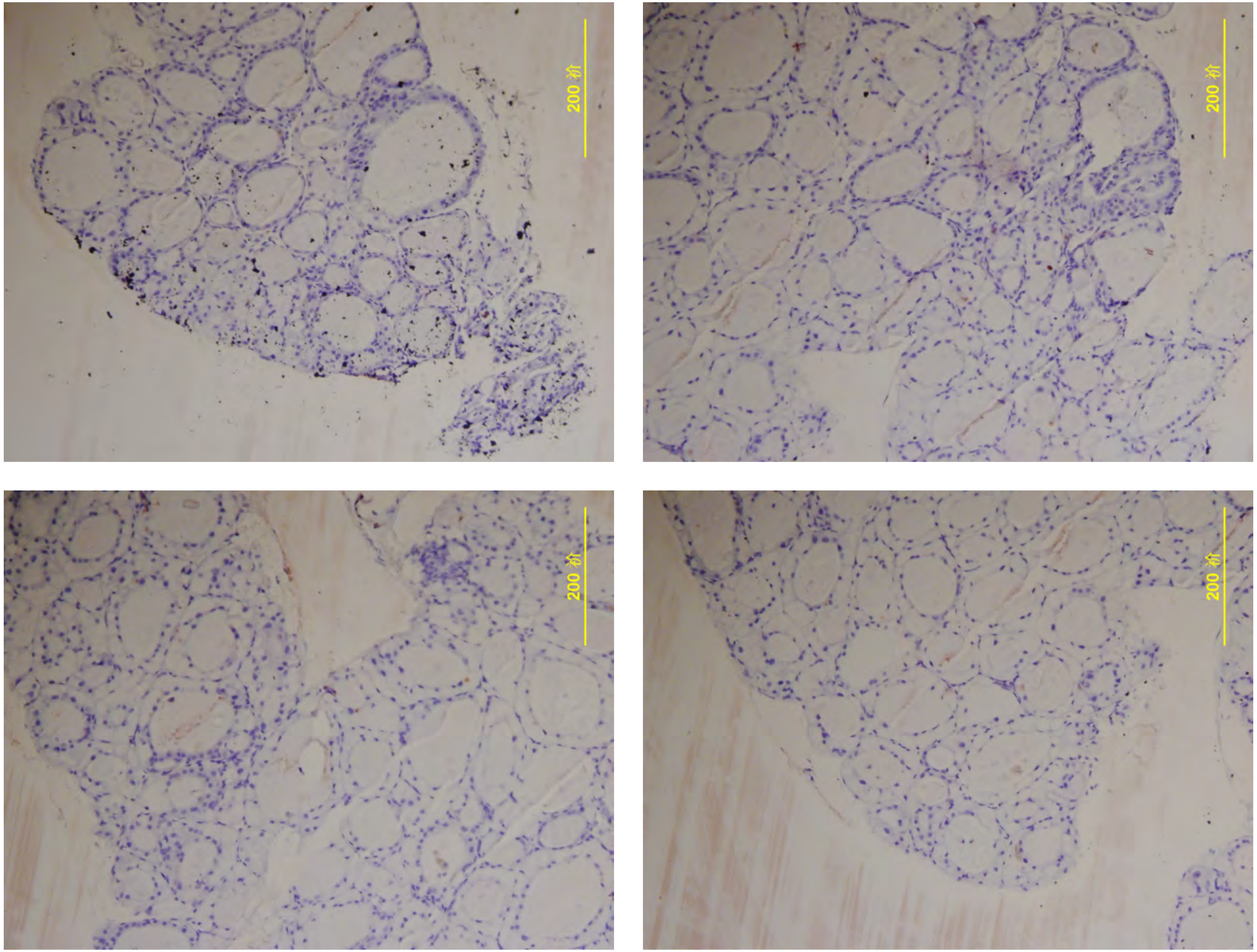

Supplemental Fig. S3

Tag# 17772 *$TG^{+/cog}$; $Hrd1^{TPO}$*

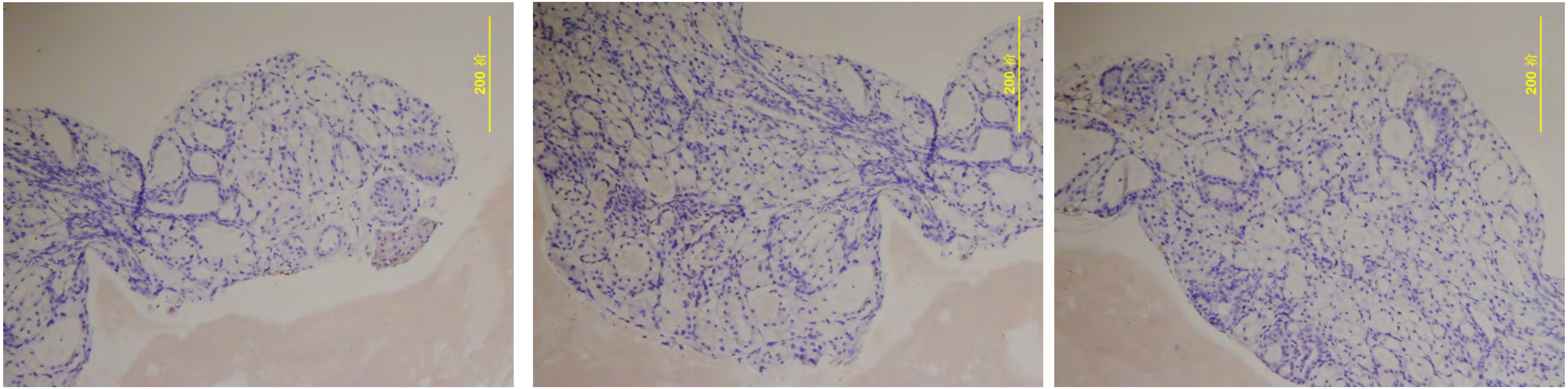

Fig S3

Quantitation

Tag Number	Genotype	x	# nuclei	# Ki67 positive nuclei	% Ki67 positive nuclei
17739	$TG^{+/cog}$; $Hrd1^{control}$	1	1225	3	0.244898
	$TG^{+/cog}$; $Hrd1^{control}$	2	1078	3	0.278293
	$TG^{+/cog}$; $Hrd1^{control}$	3	1206	5	0.414594
	$TG^{+/cog}$; $Hrd1^{control}$	4	1020	4	0.392157
17740	$TG^{+/cog}$; $Hrd1^{control}$	1	1150	8	0.695652
	$TG^{+/cog}$; $Hrd1^{control}$	2	1513	10	0.660939
	$TG^{+/cog}$; $Hrd1^{control}$	3	1257	13	1.034208
	$TG^{+/cog}$; $Hrd1^{control}$	4	1149	7	0.609225
17742	$TG^{+/cog}$; $Hrd1^{control}$	1	703	4	0.56899
	$TG^{+/cog}$; $Hrd1^{control}$	2	1047	4	0.382044
	$TG^{+/cog}$; $Hrd1^{control}$	3	954	3	0.314465
17745	$TG^{+/cog}$; $Hrd1^{TPO}$	1	878	5	0.569476
	$TG^{+/cog}$; $Hrd1^{TPO}$	2	1743	8	0.458979
	$TG^{+/cog}$; $Hrd1^{TPO}$	3	1406	6	0.426743
17760	$TG^{+/cog}$; $Hrd1^{TPO}$	1	1180	4	0.338983
	$TG^{+/cog}$; $Hrd1^{TPO}$	2	1348	9	0.667656
	$TG^{+/cog}$; $Hrd1^{TPO}$	3	1166	6	0.51458
	$TG^{+/cog}$; $Hrd1^{TPO}$	4	753	4	0.531208
17772	$TG^{+/cog}$; $Hrd1^{TPO}$	1	1043	6	0.575264
	$TG^{+/cog}$; $Hrd1^{TPO}$	2	1684	9	0.534442
	$TG^{+/cog}$; $Hrd1^{TPO}$	3	1543	8	0.518471

Supplemental Fig. S6

Spleen (Positive control for Main Fig. 8)

CD3 DAPI | CD8a DAPI | CD19 DAPI

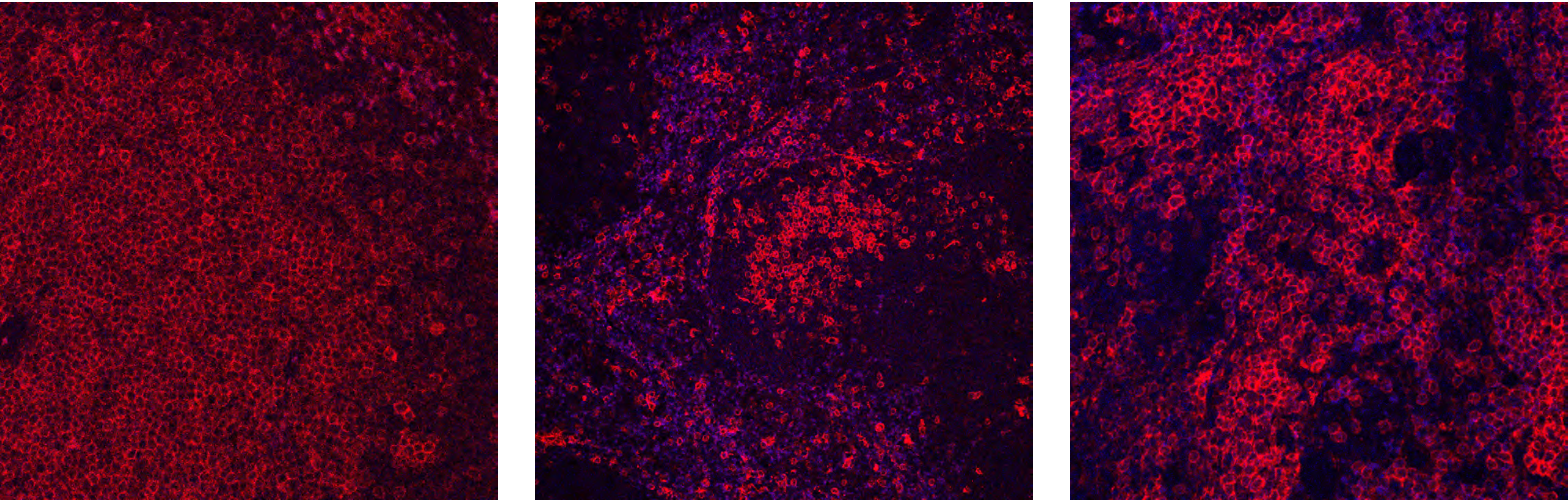

Supplemental Fig. S5

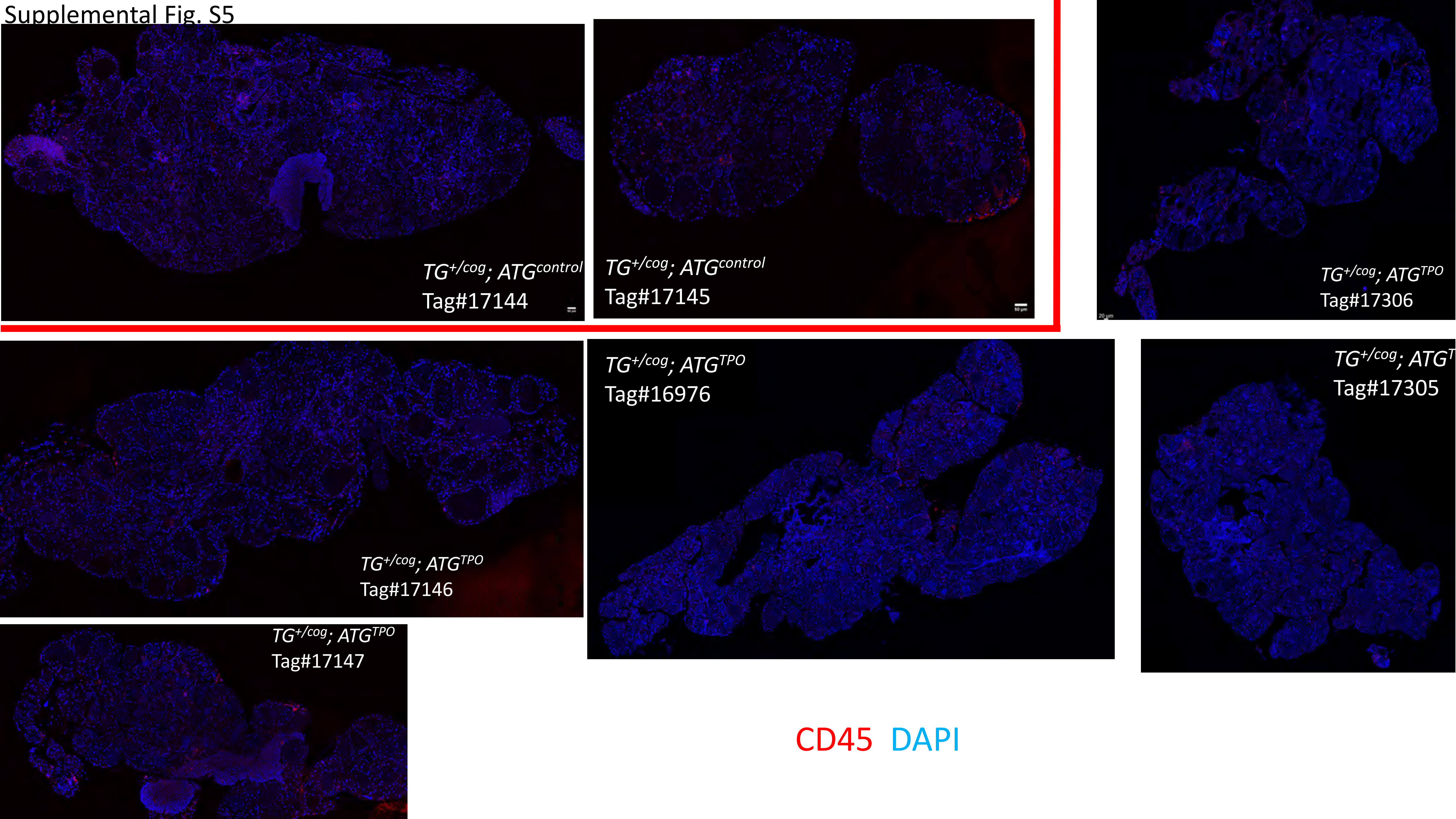

Supplemental Fig. S7

Tag#	*genotype*	Tg autoantibodies (absorbance)			
17234	$TG^{+/+}$	0.21			
17175	$TG^{+/+}$	0.123			
17177	$TG^{+/+}$	0.146			
17870	$TG^{+/+}$	0.0925			
17176	$TG^{+/+}$	0.096			
17869	$TG^{+/+}$	0.0565			
17868	$TG^{+/+}$	0.062			
18009	$TG^{+/+}$	0.0885			
18007	$TG^{+/+}$	0.2725			
17174	$TG^{+/+}$	0.0625			
18008	$TG^{+/+}$	0.0705			
18010	$TG^{+/+}$	0.05			
18011	$TG^{+/+}$	0.039			
17476	$TG^{+/cog}$	0.0745			
17475	$TG^{+/cog}$	0.0805			
17571	$TG^{+/cog}$	0.09			
17558	$TG^{+/cog}$	0.114			
17557	$TG^{+/cog}$	0.1685			
17556	$TG^{+/cog}$	0.11			
17829	$TG^{+/cog}$	0.2145			
17828	$TG^{+/cog}$	0.1005			
17472	$TG^{+/cog}$	0.0825			
17827	$TG^{+/cog}$	0.074			
17833	$TG^{+/cog}$	0.0765			
17684	$TG^{+/cog}$; $Hrd1^{control}$	0.2075			
17759	$TG^{+/cog}$; $Hrd1^{control}$	0.1555			
17826	$TG^{+/cog}$; $Hrd1^{control}$	0.1455			
17824	$TG^{+/cog}$; $Hrd1^{control}$	0.236			
17908	$TG^{+/cog}$; $Hrd1^{control}$	0.109			
17627	$TG^{+/cog}$; $Hrd1^{control}$	0.111			
17690	$TG^{+/cog}$; $Hrd1^{control}$	0.104			
17814	$TG^{+/cog}$; $Hrd1^{control}$	1.3775	Outlier by Q test		accept outlier
17920	$TG^{+/cog}$; $Hrd1^{control}$	0.126		N=15	Q95=0.384
17734	$TG^{+/cog}$; $Hrd1^{control}$	0.1195		gap:	1.125
17958	$TG^{+/cog}$; $Hrd1^{control}$	0.1535		range:	1.3055
18028	$TG^{+/cog}$; $Hrd1^{control}$	0.2525		gap/range:	0.8617388
17686	$TG^{+/cog}$; $Hrd1^{control}$	0.107			
17955	$TG^{+/cog}$; $Hrd1^{control}$	0.072			
18024	$TG^{+/cog}$; $Hrd1^{control}$	0.191			
17760	$TG^{+/cog}$; $Hrd1^{TPO}$	0.083			
17822	$TG^{+/cog}$; $Hrd1^{TPO}$	0.109			
17859	$TG^{+/cog}$; $Hrd1^{TPO}$	0.0475			
17816	$TG^{+/cog}$; $Hrd1^{TPO}$	0.075			
17682	$TG^{+/cog}$; $Hrd1^{TPO}$	0.0815			
17631	$TG^{+/cog}$; $Hrd1^{TPO}$	0.207			
17692	$TG^{+/cog}$; $Hrd1^{TPO}$	0.148			
17736	$TG^{+/cog}$; $Hrd1^{TPO}$	0.067			
17738	$TG^{+/cog}$; $Hrd1^{TPO}$	0.1195			
17728	$TG^{+/cog}$; $Hrd1^{TPO}$	0.193			
17987	$TG^{+/cog}$; $Hrd1^{TPO}$	0.1175			
17733	$TG^{+/cog}$; $Hrd1^{TPO}$	0.068			
17819	$TG^{+/cog}$; $Hrd1^{TPO}$	0.088			
17624	$TG^{+/cog}$; $Hrd1^{TPO}$	0.0705			
17925	$TG^{+/cog}$; $Hrd1^{TPO}$	0.11			
17675	$TG^{+/cog}$; $Hrd1^{TPO}$	0.224			
17983	$TG^{+/cog}$; $Hrd1^{TPO}$	0.1205			
NaI +	*pos CTR*	3.42			
